# Electrochemical selenium- and iodonium-initiated cyclisation of hydroxy-functionalised 1,4-dienes

**DOI:** 10.3762/bjoc.11.18

**Published:** 2015-01-28

**Authors:** Philipp Röse, Steffen Emge, Jun-ichi Yoshida, Gerhard Hilt

**Affiliations:** 1 Fachbereich Chemie, Philipps-Universität Marburg, Hans-Meerwein-Str. 4, 35043 Marburg, Germany; 2 Department of Synthetic Chemistry and Biological Chemistry, Graduate School of Engineering, Kyoto University, Nishikyo-ku, Kyoto 615-8510, Japan

**Keywords:** cyclic ethers, cyclisation, 1,4-dienes, electrochemistry, iodonium, selenium

## Abstract

The cobalt(I)-catalysed 1,4-hydrovinylation reaction of allyloxytrimethylsilane and allyl alcohol with substituted 1,3-dienes leads to hydroxy-functionalised 1,4-dienes in excellent regio- and diastereoselective fashion. Those 1,4-dienols can be converted into tetrahydrofuran and pyran derivatives under indirect electrochemical conditions generating selenium or iodonium cations. The reactions proceed in good yields and regioselectivities for the formation of single diastereomers.

## Introduction

The reaction of terminal alkenes with 1,3-dienes under cobalt catalysis results in 1,4-dienes in a 1,4-hydrovinylation reaction. Besides cobalt, also other transition metals were described to undergo such transformations [1–4]. However, only for the cobalt-catalysed reactions a regiodiverse reaction has been described where the carbon–carbon bond formation, either at the terminal carbon of the double bond (C1) or on C2 was formed, depending on the ligand system applied [5–6]. Besides the ozonolysis of the 1,4-dienes for the generation of 1,3-dicarbonyl derivatives [7–9], these 1,4-dienes are in turn potential substrates for the synthesis of functionalised heterocycles. Particularly, we were interested in the synthesis of tetrahydrofuran and pyran derivatives. Those heterocycles are prevalent substructures in many natural compounds, pesticides and drugs with antifungal and antibacterial properties [10–13]. For this purpose, we investigated a protocol for the straight forward synthesis of 1,4-dienols which should be cyclised into the corresponding tetrahydrofuran or pyran derivatives. With our sight set on efficient and atom economic organic reactions electrochemistry seems to be a powerful tool for the transformation of those 1,4-dienols. Although it seems that all possible functional groups have been investigated in organic electrochemistry, reports on electrochemical transformations of 1,4-dienes are rare [14–17]. First attempts of a direct electrochemical conversion were not very successful, so that we turned our attention towards indirect electrochemical methods [18–19]. Among these we became interested in the electrochemical generation of reactive cations inducing a transformation of the 1,4-diene moiety. As a starting point we put our interest in electrochemical reactions using selenium cations. Several methods applying electrochemically generated selenium cations with alkenes or alkynes including seleno-etherification and lactonisation, epoxidation and oxoselenylation sequences have been reported in the last decades [20–27]. The reactions often proceed in a regio- and stereoselective fashion and tolerate a wide range of functionalities.

Next to selenium cation induced reactions we put our focus on using halonium cations. The advantage of this type of reaction is its lower toxicity and the easy access towards the halonium source. Several reactions using bromonium- and iodonium cations such as iodo-etherification, lactonisation or Friedel–Crafts alkylation reactions can be found in literature. However, these procedures often use expensive or toxic halonium sources like molecular bromine [10,28–29] or organic trihalide salts [30], *N*-bromosuccinimide or *N*-iodosuccinimide and its derivatives [31–36], or more specialised reagents such as bis(pyridinium)iodonium(I) tetrafluoroborate [37–39]. Next to those, the in situ oxidation of halogenide ions with strong oxidants such as oxone, Pb(IV), mCPBA, FeCl_3_ or H_2_O_2_ have been reported [40–45]. A more efficient and versatile method is the electrochemical generation of halonium ions. Thereby, it is possible to accumulate the halonium ions in solution and to add those to a substrate in a separated process (“pool” method) [46–51] or to consume the halonium ions in situ in follow-up reactions inside the cell [52].

Accordingly, we envisaged the generation of suitable starting materials via a cobalt-catalysed hydrovinylation reaction and investigated their in situ conversion via electrochemically generated selenium- or iodonium cations.

## Results and Discussion

### Cobalt-catalysed 1,4-hydrovinylation of allylic alcohols

**Scheme 1:**
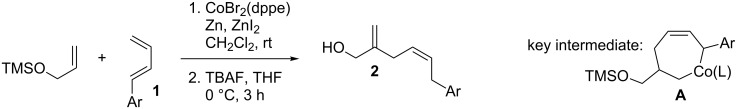
Cobalt-catalysed 1,4-hydrovinylation.

For the successful application of 1,4-dienes in the electrochemical reactions, 1,4-dienes with additional internal nucleophiles, such as an alcohol group, were envisaged and those 1,4-dienols could be generated from simple 1,3-dienes, such as 1,3-butadiene or 1-aryl-substituted 1,3-dienes **1**, and TMS-protected allylic alcohol (Scheme 1) for the synthesis of 1,4-dienols of type **2**.

The cobalt-catalysed hydrovinylation reaction is highly regiospecific for the carbon–carbon bond formation which takes place exclusively at the internal carbon of the double bond of the terminal alkene (C2) and C4 of the 1-aryl-substituted 1,3-diene. The key intermediate **A** in the reaction mechanism is proposed to be a cobaltacycle which only allows the double bond generated from the 1,3-diene component to adopt a *Z*-configuration. Accordingly, the products of type **2** are formed in high selectivity in terms of regio- and diastereomeric control.

The starting materials of type **1** were generated from the aromatic aldehydes and allyltriphenylphosphonium bromide in a Wittig reaction following a known protocol [53]. The synthesis of the 1,4-dienes was then accomplished utilising the cobalt-catalyst precursor and reducing conditions in the presence of zinc iodide for abstracting the bromide anions at room temperature. The TMS-protected allylic alcohol was applied in the cobalt-catalysed 1,4-hydrovinylation process with aryl-substituted 1,3-dienes **1a–k** because the use of allylic alcohol itself led to significant lower yields (up to 30%). Only in case of buta-1,3-diene, 2,3-dimethyl-1,3-butadiene and isoprene allyl alcohol could be used directly without decreasing the yield (Table 1, entries 12–14). The results of the 1,4-dienol syntheses are summarised in Table 1.

**Table 1:** Results of the cobalt-catalysed 1,4-hydrovinylation reaction of TMS-protected allylic alcohol with 1,3-dienes of type **1**.

Entry	1,3-Diene (**1**)	1,4-Dienol (**2**)	Yield^a^

1	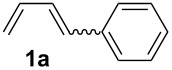	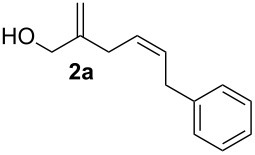	60%
2	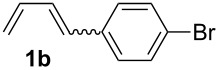	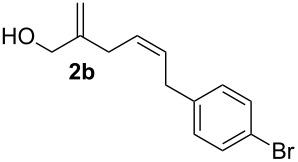	71%
3	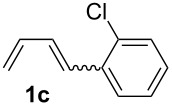	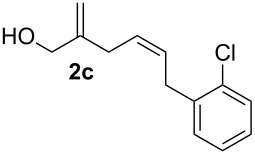	81%
4	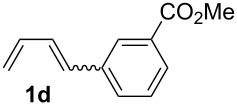	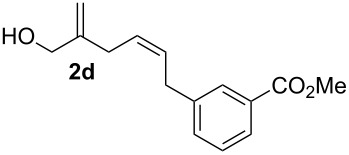	51%
5	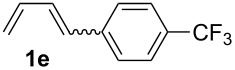	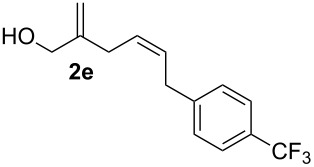	69%
6	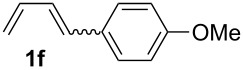	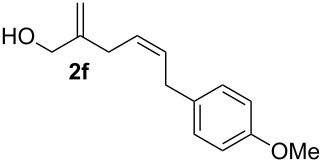	87%
7	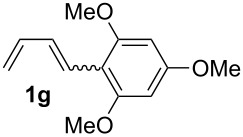	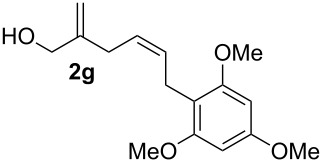	64%
8	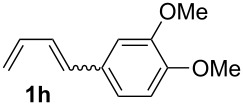	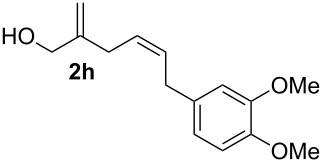	67%
9	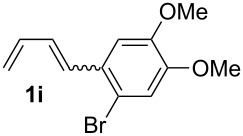	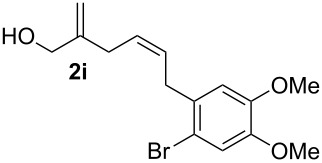	60%
10	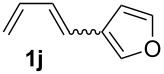	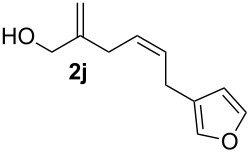	59%
11	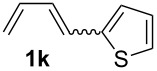	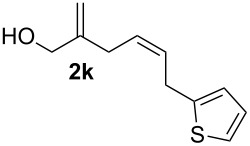	87%
12	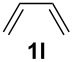	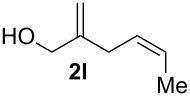	92%^b^
13	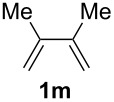	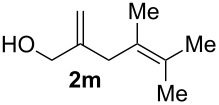	90%^b^
14	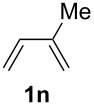	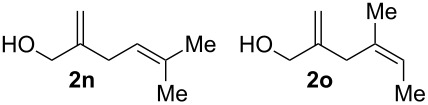	89% (**2n**:**2o** = 79:21)

^a^Reaction conditions: (i) 1,3-diene (1.0 equiv), allyloxytrimethylsilane (1.2 equiv), CoBr_2_(dppe) (5–10 mol %), Zn powder (10–20 mol %), ZnI_2_ (10–20 mol %), CH_2_Cl_2_ (1 mL/mmol), rt, 12–16 h (ii) TBAF (1.1 equiv), THF, 0 °C, 3 h. ^b^Reaction conditions: 1,3-diene (1.0 equiv), allyl alcohol (1.2–2.0 equiv), CoBr_2_(dppe) (5–10 mol %), Zn powder (10–20 mol %), ZnI_2_ (10–20 mol %), CH_2_Cl_2_ (1 mL/mmol), rt, 12–16 h.

The cobalt-catalysed hydrovinylation tolerates halide, ether, ester, trifluoromethyl and heterocyclic substituents and gave the desired products in acceptable to good yields over a two-step reaction sequence of hydrovinylation and deprotection. Electron-withdrawing and electron-donating groups as well as sterically hindered aryl substituents are also accepted (see Table 1, entries 7 and 9). The use of buta-1,3-diene and 2,3-dimethyl-1,3-butadiene gave the 1,4-dienols in excellent yields (Table 1, entries 12 and 13). When isoprene was used, the regioisomeric products **2n** and **2o** were formed in good yields and acceptable regioselectivity, with the carbon–carbon bond formation taking place predominantly at the lower substituted end of the 1,3-diene moiety. These results demonstrate that the cobalt-catalysed hydrovinylation reaction is a powerful tool for the straightforward synthesis of various 1,4-dienols of type **2** and the mild reaction conditions made it possible to generate and isolate the products without isomerisation of the double bonds towards undesired side-products.

### Transformation of 1,4-dienols via electro-generated selenium cations

**Scheme 2:**
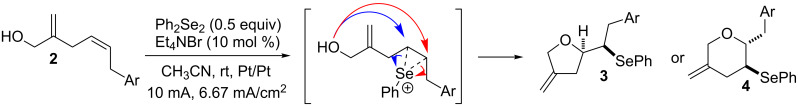
Electrochemical selenoalkoxylation of **2**.

The 1,4-dienols were transformed into cyclic phenylselenoethers by intramolecular cyclisation using selenium cations generated by indirect electrolysis. The reaction was carried out by electrolysing a mixture of the 1,4-dienol, diphenyl diselenide and tetraethylammonium bromide in CH_3_CN at room temperature in an undivided cell, using platinum foil electrodes (constant current 10 mA). In this investigation only diphenyl diselenide was used as selenium source. These reaction conditions led to the formation of products of type **3** as exclusive diastereomers (Scheme 2).

The cyclisation of **2** could lead to a number of products. The PhSe^+^ cation could interact with the 1,1-disubstituted double bond and nucleophilic attack could lead to oxiran- or oxetan-type products. On the other hand, the interaction of the PhSe^+^ ion with the 1,2-disubstituted double bond would lead to the furan-type products **3** or alternatively to pyran-type product **4**. The results of the electrochemical selenoalkoxylation of the 1,4-dienols are summarised in Table 2.

**Table 2:** Electrochemical selenoalkoxylation of 1,4-dienols **2**^a^.

Entry	1,4-Dienol (**2**)	Furan (**3**) or pyran (**4**)	Yield^b^

1	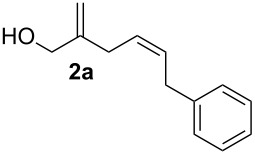	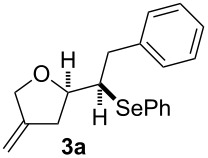	50%
2	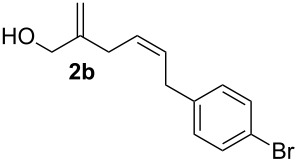	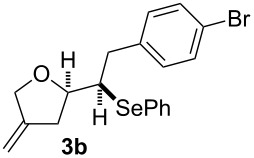	82%
3	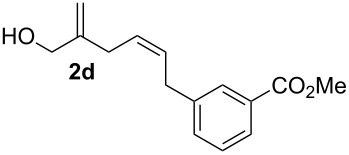	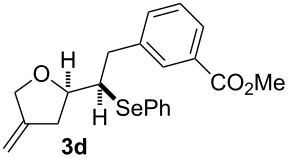	58%
4	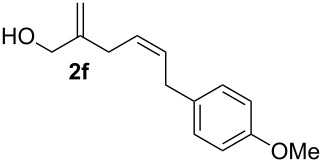	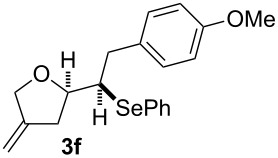	68%
5	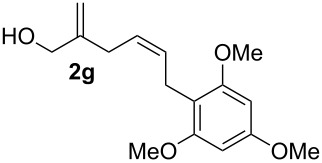	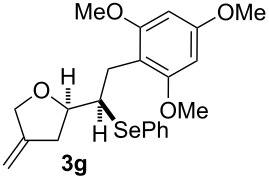	42%
6	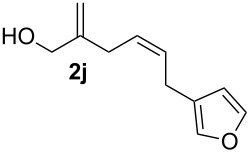	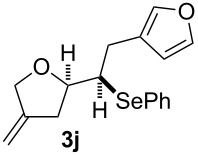	90%
7	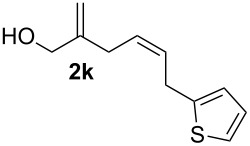	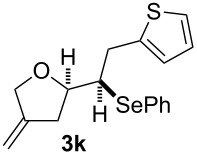	62%
8	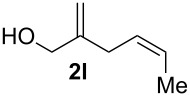	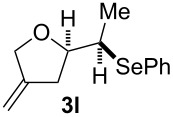	86%
9	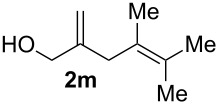	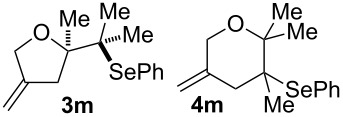	46% (**3m**) 47% (**4m**)
10	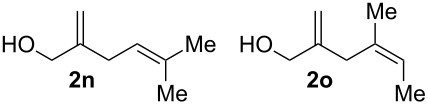	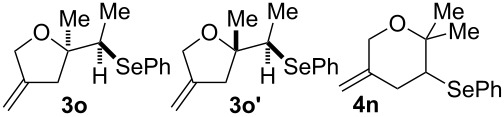	10% (**3o**+**3o’**) 58% (**4n**)
11	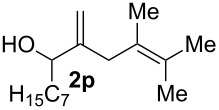	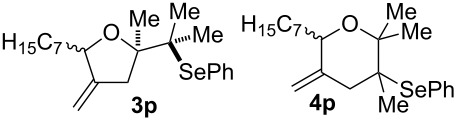	75% (**3p**:**4p** = 1:2.5)
12	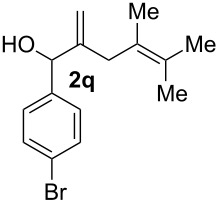	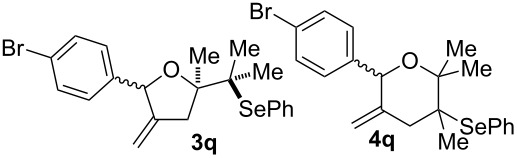	53% (**3q**:**4q** = 1:1.4)

^a^Reaction conditions: 1,4-dienol (0.5 mmol, 1.0 equiv), Ph_2_Se_2_ (0.5 equiv), Et_4_NBr (0.1 equiv), CH_3_CN (10 mL), rt, undivided cell, Pt/Pt, 10 mA, 6.67 mA/cm^2^. ^b^Oxidation of the alcohol could be observed in all reactions (less than 10%).

The electrochemical selenoalkoxylation of the aryl-substituted derivatives of type **2** (Table 2, entries 1–7) led exclusively to the tetrahydrofuran derivatives **3** via a 5-*exo-tet*-type cyclisation in moderate to good yields. The products were generated in diastereoselective fashion and the configuration of the formed diastereomer could be identified by ^1^H NMR experiments comparing a mixture of both diastereomers, synthesised by conventional selenoalkoxylation, with the electrochemically generated selenoether (see Supporting Information File 1). As a side reaction the oxidation of the alcohol could be observed in all reactions (less than 10%). Moreover, under electrochemical conditions the simple methyl-substituted derivative **2l** led to the tetrahydrofuran-type product **3l** in 86% yield (Table 2, entry 8) while the reaction using PhSeBr under conventional methods gave the product in 76% yield and a diastereoselectivity of 73:27 (*threo*:*erythro*). When alkyl-substituted 1,4-dienols are used, the formation of tetrahydrofuran or pyran derivatives can be observed (Table 2, entries 9 and 10). Depending on the substitution grade of the internal double bond, the alcohol functionality attacks at the less-substituted carbon of the internal double bond based on better stabilisation of the cationic intermediate. The triple methyl-substituted starting material **2m** gave a 1:1 mixture of tetrahydrofuran and pyran products **3m** and **4m**, the latter product is formed via a 6-*endo-tet*-type cyclisation, in an excellent combined yield of 93%. When the mixture of the 1,4-dienols (**2n** and **2o**) was applied, the major regioisomer **2n** gave the pyran exclusively in good yields, while the minor 1,4-dienol **2o** resulted in the formation of two tetrahydrofuran diastereomers which could be separated easily by column chromatography (Table 2, entry 10). Using higher substituted 1,4-dienols (**2p** and **2q)** a preferred formation of the pyran products was observed (Table 2, entries 11 and 12). However, under no circumstances the previously discussed strained epoxy-derivatives could be detected.

### Transformation of 1,4-dienols via electro-generated iodonium cations

**Scheme 3:**
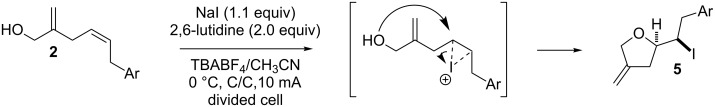
Electrochemical iodoalkoxylation of **2**.

In a similar approach we investigated the cyclisation of the 1,4-dienols **2** with in situ electrochemically generated iodonium ions for the desired synthesis of iodoalkoxylated products of type **5** (Scheme 3).

The electrolysis was carried out in an H-type divided cell (4G glass filter) equipped with carbon fiber electrodes (see Supporting Information File 1). Each chamber was charged with 2,6-lutidine and TBABF_4_ in CH_3_CN (0.3 M) and additionally the 1,4-dienol and sodium iodide were placed in the anode chamber. The reaction was performed at constant current electrolysis (10 mA) at 0 °C. It is considerable that the presence of 2,6-lutidine is crucial for a successful reaction. In the absence of 2,6-lutidine only traces of the product can be observed and oxidation of the alcohol functionality takes place. It is mentionable that under the reaction conditions no aromatic iodination could be observed. Next to sodium iodide other iodide sources such as KI, I_2_ or Bu_4_NI can be used, whereas applying other halogenides such as NaBr, Et_4_NBr or Bu_4_NCl led to a complex mixture of products.

In this series of experiments we focussed our attention on the aryl-substituted 1,4-dienes to avoid undesired mixtures of tetrahydrofuran and pyran derivatives as obtained in the selenoalkoxylation for alkyl-substituted dienols. The results of the electrochemical iodoalkoxylation reactions are summarised in Table 3.

**Table 3:** Electrochemical iodoalkoxylation of 1,4-dienols **2**.

Entry	1,4-Dienol **2**	Iodofuran **5**	Yield^a^

1	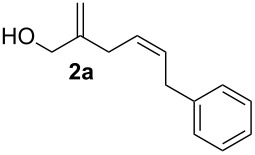	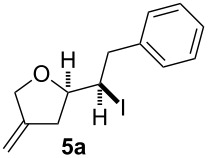	66%
2	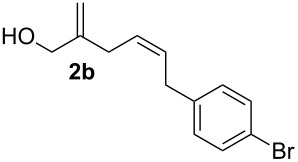	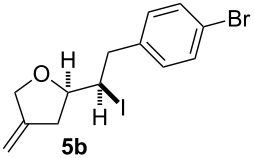	60%
3	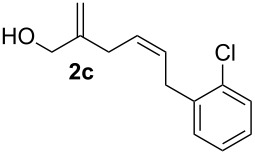	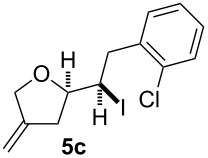	40%
4	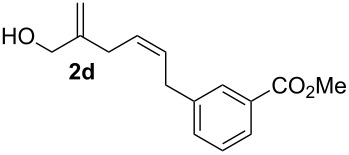	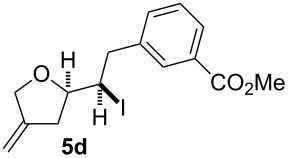	68%
5	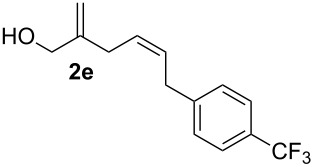	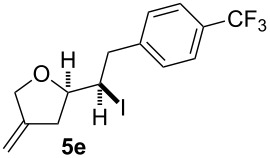	46%
6	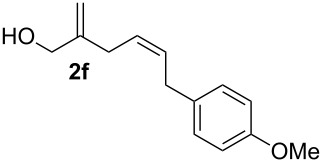	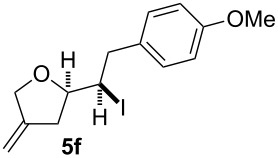	68%
7	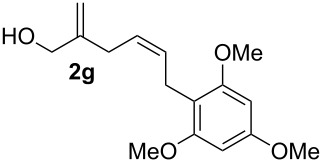	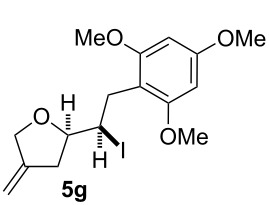	70%
8	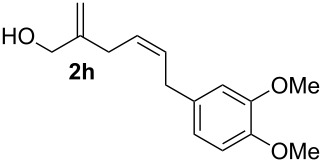	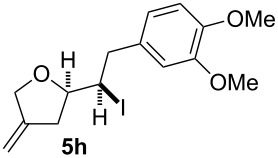	67%
9	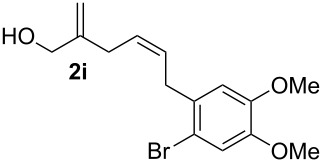	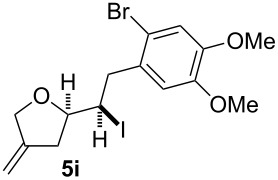	46%
10	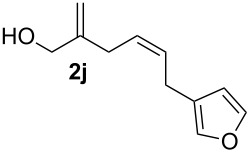	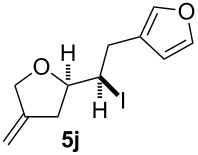	82%
11	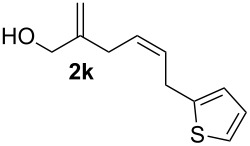	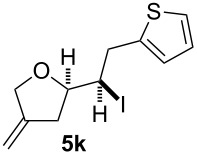	66%

^a^Reaction conditions: H-type cell, anodic chamber: 1,4-dienol (0.5 mmol, 1.0 equiv), NaI (1.1 equiv), 2,6-lutidine (2.0 equiv), TBABF_4_/CH_3_CN (0.3 M, 10 mL); cathode chamber: 2,6-lutidine (2.0 equiv), TBABF_4_/CH_3_CN (0.3 M, 10 mL); 0 °C, C/C, 10 mA.

The electrochemical iodoalkoxylation of the aryl-substituted 1,4-dienols **2** led to the formation of the tetrahydrofuran derivatives **5** as single regio- and diastereomers. 1,4-Dienols with electron-donating and electron-withdrawing substituents as well as heterocyclic compounds gave the product of type **5** in good yields. It is noteworthy, that under these reaction conditions the electron-rich 1,4-dienols (**2g** and **2h**) gave higher yields than in the selenoalkoxylation (Table 3, entries 7 and 8). For compound **5g** the structure could be verified by X-ray analysis (see Supporting Information File 1). It is also considerable that the 1,4-dienols **2c**, **2e** and **2i** which did not gave the selenoether **3** led to a product formation in at least moderate yields of 40% to 46% (Table 3, entries 3, 5 and 9).

The products of the selenoalkoxylation as well as of the iodoalkoxylation are interesting building blocks for further transformations which are under current investigation.

## Conclusion

In conclusion, we have developed the alkoxylation of 1,4-dienols by electrochemically generated selenium and iodonium cations. First, the synthesis of 1,4-dienols via a cobalt-catalysed 1,4-hydrovinylation of substituted 1,3-dienes with allyloxy-trimethylsilane or allyl alcohol has been elaborated. Those 1,4-dienols have been transformed into tetrahydrofuran or pyran derivatives by constant current electrolysis of suitable selenium and iodonium precursors. The reactions proceed in acceptable to good yields in regio- and diasterioselective fashion and tolerate a range of functionalities.

## Experimental

### General procedure for the cobalt-catalysed 1,4-hydrovinylation of aryl-substituted buta-1,3-dienes with allyloxytrimethylsilane and subsequent desilylation with TBAF

Cobalt dibromo(1,3-bis(diphenylphosphino)ethane) (5–10 mol %), zinc powder (10–20 mol %) and zinc iodide (10–20 mol %) were suspended in dichloromethane and stirred at room temperature for 20 min. Then the 1,3-butadiene (1.0 equiv) and the allyloxytrimethylsilane (1.2–2.0 equiv) were added and the mixture was stirred at room temperature until complete conversion was detected by TLC and GC–MS analysis. *n*-Pentane was added, the mixture was filtered through a short pad of silica and concentrated under reduced pressure. The crude material was dissolved in 5 mL tetrahydrofuran, TBAF (1 M in THF, 1.1 equiv) was added and the mixture was stirred at 0 °C for 3 h. Upon completion of the reaction 15 mL water were added, the mixture was extracted with diethyl ether (three times 15 mL), dried over Na_2_SO_4_, filtered and concentrated under reduced pressure. The product was obtained after column chromatography (*n*-pentane/diethyl ether).

### General procedure for the cobalt-catalysed 1,4-hydrovinylation of buta-1,3-dienes with allyl alcohol

Cobalt dibromo(1,3-bis(diphenylphosphino)ethane) (5 mol %), zinc powder (10 mol %) and zinc iodide (10 mol %) were suspended in dichloromethane and stirred at room temperature for 20 min. Then the 1,3-butadiene (1.0 equiv) and allyl alcohol (1.2–1.5 equiv) were added and stirred at room temperature until complete conversion was detected by TLC and GC–MS analysis. Pentane was added and the mixture was filtered through a short pad of silica. The solvent was evaporated and the crude product was purified by column chromatography to give the desired 1,4-diene.

### General procedure for the electrochemical seleno-alkoxylation of 1,4-dienols

An undivided electrolysis cell was charged with diphenyl diselenide (78 mg, 0.25 mmol, 0.5 equiv), tetraethylammonium bromide (11 mg, 0.05 mmol, 0.1 equiv), the 1,4-diene (0.5 mmol, 1.0 equiv) and 10 mL acetonitrile. Then, the cell was equipped with a platinum plate anode and cathode and electrolysed under a constant current (10 mA, 6.67 mA/cm^2^) at 20 °C until completion was detected by TLC and GC–MS analysis. Then, 15 mL water were added, the mixture was extracted with diethyl ether (three times 15 mL), dried over Na_2_SO_4_ and the solvent was removed under reduced pressure. The product was obtained after column chromatography (*n*-pentane/diethyl ether).

### General procedure for the electrochemical iodonium-induced alkoxylation of 1,4-dienols

An H-type divided cell (4G glass filter) was equipped with a carbon fiber anode and carbon fiber cathode. Each chamber was charged with 10 mL TBABF_4_ solution (0.3 M in acetonitrile) and 2,6-lutidine (2.0 equiv). The anodic chamber was charged with the 1,4-diene (1.0 equiv) and sodium iodide (1.1 equiv). Constant current electrolysis (10 mA) was carried out at 0 °C until completion was detected by TLC and GC–MS analysis. Then, 15 mL Na_2_S_2_O_3_ solution (10% in water) were added to both chambers, the mixture was extracted with diethyl ether (three times 15 mL), dried over MgSO_4_ and the solvent was removed under reduced pressure. The product was obtained after column chromatography (*n*-pentane/diethyl ether).

## Supporting Information

Experimental details and detailed spectroscopic data of all compounds are available as Supporting Information. Single crystal data for compound **5g** (CCDC 1030546) has been deposited in the Cambridge Crystallographic Data Center.

File 1 Experimental details and detailed spectroscopic data.
