# Supplementary material for: Electrochemical selenium- and iodonium-initiated cyclisation of hydroxy-functionalised 1,4-dienes
Source: Beilstein J Org Chem. 2015 Jan 28;11:174–83. doi: 10.3762/bjoc.11.18 (PMC4361995; doi:10.3762/bjoc.11.18)

Supporting Information

for

Electrochemical selenium- and iodonium-initiated cyclisation of hydroxy-functionalised 1,4-dienes

Philipp Röse[1], Steffen Emge[1], Jun-ichi Yoshida[2], Gerhard Hilt[1]*

Address: [1]Fachbereich Chemie, Philipps-Universität Marburg, Hans-Meerwein-Str. 4, 35043 Marburg, Germany and [2]Department of Synthetic Chemistry and Biological Chemistry, Graduate School of Engineering, Kyoto University, Nishikyo-ku, Kyoto 615-8510, Japan

Email: hilt@chemie.uni-marburg.de

*Corresponding author

Table of contents **Pages**

Schematic construction of the undivided electrolysis cell 2

Schematic construction of the divided electrolysis cell 3

Comparison of the diastereo selectivity in conventional and electrochemical selenoetherfication 4

Crystal structure of *threo*-2-(1-iodo-2-(2,4,6-trimethoxyphenyl)ethyl)-4-methylene-tetrahydrofuran 5

Experimental 7

- General procedure for the preparation of aryl substituted 1,3-butadienes (GP1) 8
- General procedure for the cobalt-catalyzed 1,4-hydrovinylation of aryl-substituted buta-1,3-dienes with allyloxytrimethylsilane and subsequent desilylation with TBAF (GP2) 14
- General procedure for the cobalt-catalyzed 1,4-hydrovinylation of alkenes with buta-1,3-dienes (GP3) 21
- General procedure for the synthesis of 1,4-dienols containing secondary alcohol functions (GP4) 23
- General procedure for the electrochemical selenoalkoxylation of 1,4-dienols (GP5) 25
- General procedure for the electrochemical iodonium induced alkoxylation of 1,4-dienols (GP6) 34

^{1}H and ^{13}C NMR spectra 42

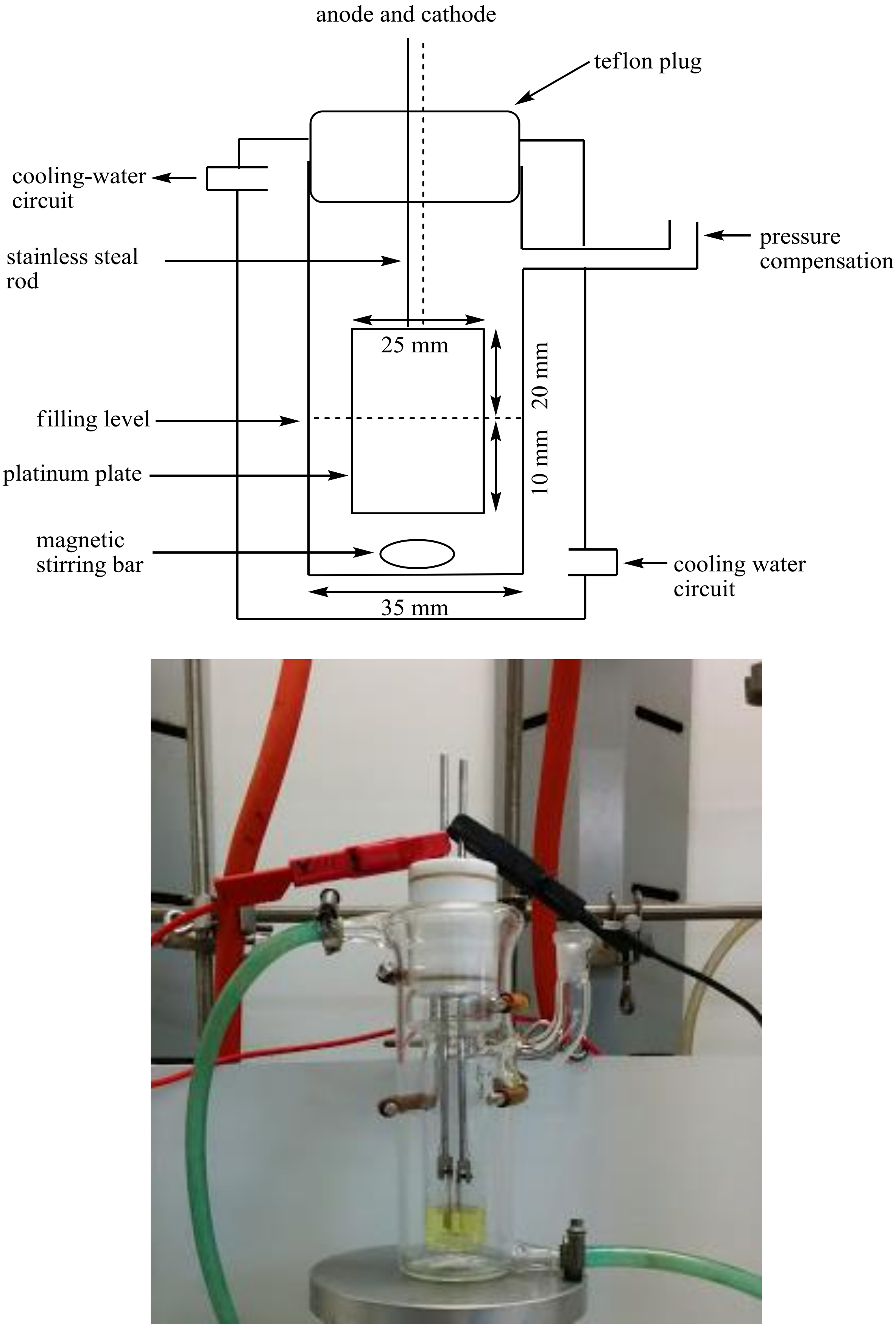

Figure 1: Schematic construction of the undivided electrolysis cell (top), photo of the undivided electrolysis cell (bottom).

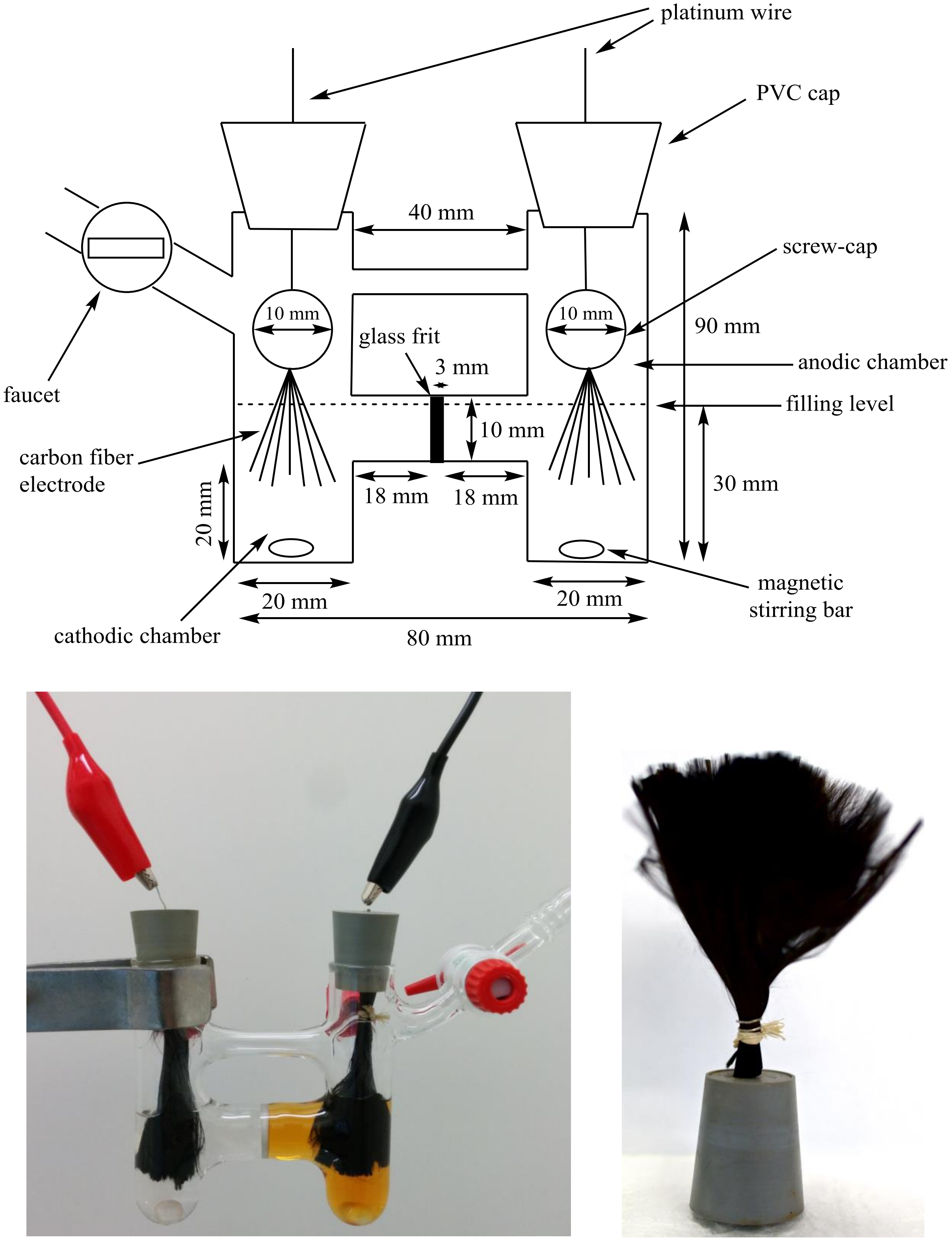

Figure 2: Schematic construction of the divided electrolysis cell (top), photo of the divided electrolysis cell (left) and photo of the carbon fiber electrode (right).

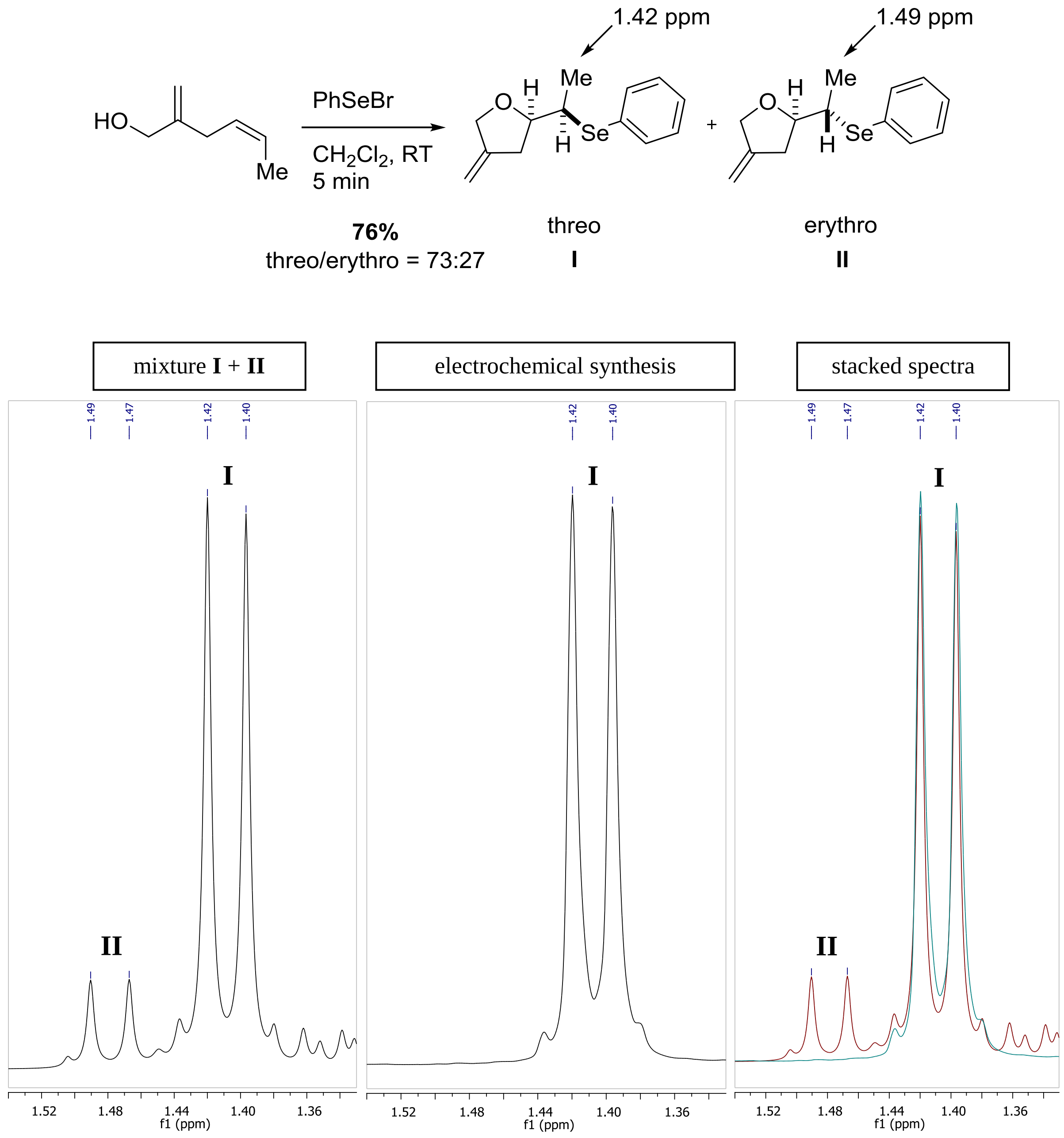

Figure 3: Comparison of the diastereoselectivity in conventional[1] and electrochemical selenoetherfication of 1,4-dienol (2l).

[1] V. M. Divac, Z. M. Burgarčić, *Synthesis* **2009**, *21*, 3684-3688.

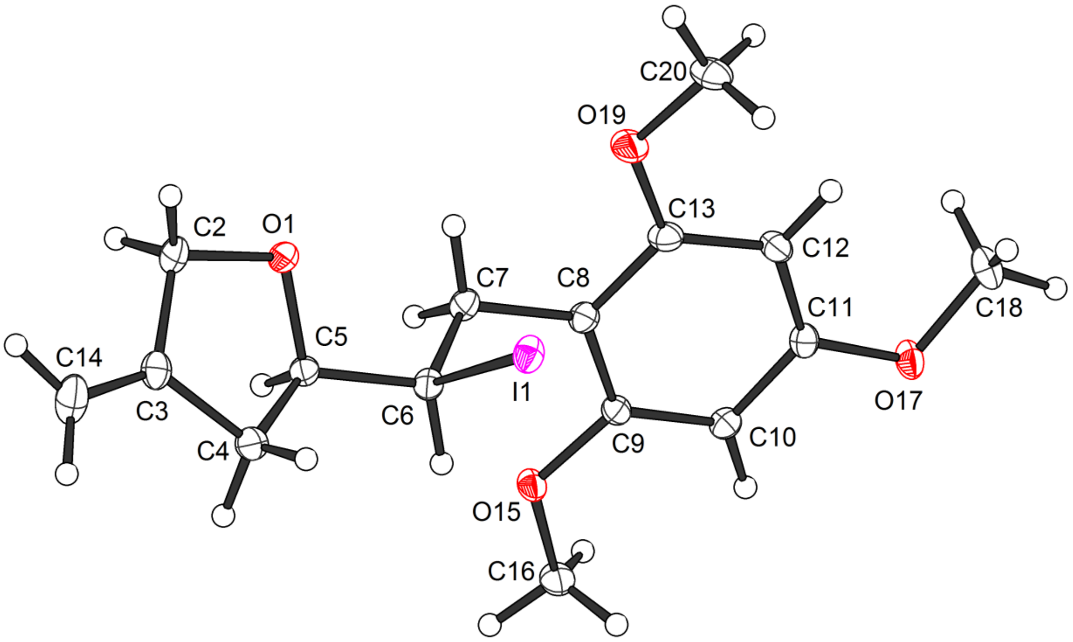

Figure 4: Crystal structure of *threo*-2-(1-iodo-2-(2,4,6-trimethoxyphenyl)ethyl)-4-methylenetetrahydrofuran (5g).

Crystal data of **(5g):**

Habitus, colour	plate-needle, colorless	
Crystal size	0.300 x 0.070 x 0.060 mm^3	
Crystal system	Monoclinic	
Space group	P 2_1/c	Z = 4
Unit cell dimensions	a = 7.2029(4) Å	α= 90°.
	b = 15.3185(9) Å	β= 98.549(2)°.
	c = 14.6777(8) Å	γ = 90°.
Volume	1601.51(16) $Å^3$	
Cell determination	9902 peaks with Theta 2.8 to 27.5°	
Empirical formula	$C_{16} H_{21} I O_4$	
Moiety formula	$C_{16} H_{21} I O_4$	
Formula weight	404.23	
Density (calculated)	1.677 Mg/m^3	
Absorption coefficient	2.012 mm^{-1}	
F(000)	808	

Data collection:

Diffractometer type	Bruker D8 QUEST area detector
Wavelength	0.71073 Å
Temperature	110(2) K
Theta range for data collection	2.807 to 27.568°
Index ranges	-9<=h<=9, -19<=k<=19, -19<=l<=19
Data collection software	BRUKER APEX2 2014.1-1
Cell refinement software	SAINT V8.34A (Bruker AXS Inc., 2013)
Data reduction software	SAINT V8.34A (Bruker AXS Inc., 2013)

Solution and refinement:

Reflections collected	140946
Independent reflections	3702 [R(int) = 0.0441]
Completeness to theta = 25.242°	99.9 %
Observed reflections	3524[II > 2(I)]
Reflections used for refinement	3702
Absorption correction	Numerical
Max. and min. transmission	0.89 and 0.59
Largest diff. peak and hole	0.470 and -0.184 e.$Å^{-3}$
Solution	Direct methods
Refinement	Full-matrix least-squares on F^2
Treatment of hydrogen atoms	Calculated positions, constr. ref.
Programs used	SHELXS-97 (Sheldrick, 2008)
	SHELXL-2013 (Sheldrick, 2013)
	DIAMOND (Crystal Impact)
Data / restraints / parameters	3702 / 0 / 193
Goodness-of-fit on F^2	1.107
R index (all data)	wR2 = 0.0384
R index conventional [I>2sigma(I)]	R1 = 0.0152

Experimental

General methods

^{1}H-NMR spectra were recorded on either an AV-300 (300 MHz), DRX-400 (400 MHz), AV-500 (500MHz) or a DRX-500 (500 MHz) all manufactured by Bruker Physics. The spectra were recorded at room temperature utilizing preset pulse programs. The chemical shifts are given relative to tetramethylsilane as an internal standard. The solvent signal was used for calibration (usually δ ($CHCl_3$) = 7.26 ppm).

^{13}C-NMR spectra were recorded on either an AV-300 (75 MHz), DRX-400 (100 MHz), AV-500 (125 MHz) or a DRX-500 (150 MHz) all manufactured by Bruker Physics. The chemical shifts are given relative to tetramethylsilane as an internal standard. The solvent signal was used for calibration (usually δ ($CHCl_3$) = 77.16 ppm).

Infrared spectra (IR) were recorded on an Alpha-P FT-IR-Spectrometer manufactured by Bruker Physics. The absorption bands are given in wave numbers (cm^{-1}).

GC/MS spectra were recorded utilizing an Agilent 6890 GC-System coupled with a Hewlett Packard 5973 Mass Selective Detector. Ionization was accomplished by electron ionization (EI) at energy of 70 eV. The detected ion masses (m/z) are reported in u corresponding to the intensity of the signals as a percentage of the most intense signal.

High-resolution mass spectra (HRMS) were recorded as electron ionization spectra (EI/HRMS) at an energy of 70 eV on a Finnigan MAT 95S Mass Spectrometer or as electron spray ionization (ESI/HRMS) on a Micromass VG AutoSpec Mass Spectrometer. The detected ion masses (m/z) are given in u.

Thin layer chromatography (TLC) was carried out on prefabricated plates (silica gel 60, F254 with fluorescence indicator) manufactured by Merck. Detection was carried out by fluorescence quenching with a UV lamp (υ= 254 nm) and development of the plates by immersion in either potassium permanganate staining solution ($NaHCO_3$) (12.5 g), $KMnO_4$ (3.8 g), H_2O (1000 mL) or cerium sulphate staining solution ($Ce(SO_4)_2$ (2.00 g), concentrated sulphuric acid (50 mL), ammonium molybdate (50.0 g), H_2O (400 mL)) followed by gentle heating.

Flash chromatography (FC) was carried out on silica gel 60 (40-64 μm, 230-400 mesh ASTM) purchased from either Merck or Macherey-Nagel at room temperature and with 0.2-0.5 overpressure (N_2). The utilized solvents were dried using common procedures.

Solvents: DCM was dried over P_4O_{10}, CH_3CN was dried over CaH_2, while THF and diethyl ether were dried over sodium/benzophenone and distilled under a nitrogen atmosphere. Other solvents were dried using common procedures.

Reactions requiring water- or air-sensitive compounds were carried out in vacuum- and flame-dried flasks utilizing *Schlenk* techniques under an argon atmosphere kept at a slight overpressure. Reaction progress was monitored by TLC- or GC/MS-analysis.

The carbon fiber electrode material was purchased from R&G Faserverbundwerkstoffe GmbH Germany (item description: Carbon roving PyrofilTM TR50S 6k /400 tex). Before use, the carbon fibers were bound together and fixed with a platinum wire (see SI, figure 2).

General procedure for the preparation of aryl-substituted 1,3-butadienes (GP1)[2]

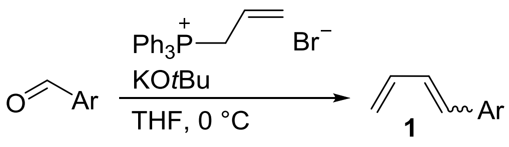

Allyltriphenylphosphonium bromide (1.1 equiv) and potassium *tert*-butoxide (1.2 equiv) were dissolved in THF and stirred at ambient temperature for 20 min. The suspension was cooled to 0 °C, the aldehyde (1.0 equiv) was added and the mixture was stirred until complete conversion was detected by TLC and GC-MS analysis. Then, saturated ammonium chloride solution was added followed by extraction with 3x 15 mL diethyl ether. The organic layers were combined, dried over Na_2SO_4 and concentrated under reduced pressure. The crude product was adsorbed on silica and was purified by column chromatography to give a mixture of *E*/*Z* isomers. The *E*/*Z* ratios were determined by integration of suitable ^{1}H NMR signals and GC-Analysis.

Buta-1,3-dienylbenzene (1a)

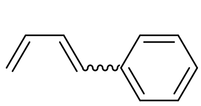

The title compound was synthesized according to GP1 using allyltriphenylphosphonium bromide (4.22 g, 11.0 mmol, 1.1 equiv), potassium *tert*-butoxide (1.35 g, 12.0 mmol, 1.2 equiv) and benzaldehyde (1.06 g, 10.0 mmol, 1.0 equiv) and was obtained after column

[2] G. Hilt, M. Danz, *Synthesis* **2008**, *14*, 2257-2263.

chromatography (eluent: *n*-pentane) as colorless liquid (937 mg, 7.20 mmol, 72%, *E*/*Z* ratio 60:40). **^{1}H NMR** (300 MHz, $CDCl_3$) δ 7.51 – 7.21 (m, 10H), 7.04 – 6.73 (m, 2H), 6.69 – 6.42 (m, 3H), 6.30 (t, *J* = 11.3 Hz, 1H), 5.40 (ddd, *J* = 16.8, 7.1, 6.2 Hz, 2H), 5.31 – 5.15 (m, 2H). **^{13}C NMR** (75 MHz, $CDCl_3$) δ 137.5, 137.33, 137.29, 133.4, 133.0, 130.9, 130.6, 129.8, 129.2, 128.8, 128.4, 127.8, 127.2, 126.6, 119.8, 117.7.

The analytical data are in accordance with the literature.[3]

1-Bromo-4-(buta-1,3-dienyl)benzene (1b)

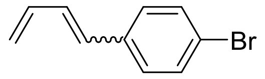

The title compound was synthesized according to GP1 using allyltriphenylphosphonium bromide (4.22 g, 11.0 mmol, 1.1 equiv), potassium *tert*-butoxide (1.35 g, 12.0 mmol, 1.2 equiv) and 4-bromobenzaldehyde (1.85 g, 10.0 mmol, 1.0 equiv) and was obtained after column chromatography (eluent: *n*-pentane) as colorless liquid (1.49 g, 7.13 mmol, 71%, *E*/*Z* ratio 61:39). **^{1}H NMR** (300 MHz, $CDCl_3$) δ 7.61 – 7.36 (m, 4H), 7.26 (dd, *J* = 10.8, 8.0 Hz, 2H), 7.19 (d, *J* = 8.4 Hz, 2H), 6.80 (ddd, *J* = 15.0, 13.6, 7.8 Hz, 2H), 6.64 – 6.14 (m, 4H), 5.50 – 5.31 (m, 2H), 5.31 – 5.13 (m, 2H). **^{13}C NMR** (75 MHz, $CDCl_3$) δ 137.0, 136.4, 136.2, 132.9, 131.9, 131.7, 131.6, 131.5, 130.7, 130.5, 129.2, 128.0, 121.5, 121.1, 120.5, 118.5.

The analytical data are in accordance with the literature.[4]

1-(Buta-1,3-dienyl)-2-chlorobenzene (1c)

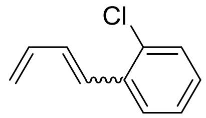

The title compound was synthesized according to GP1 using allyltriphenylphosphonium bromide (4.22 g, 11.0 mmol, 1.1 equiv), potassium *tert*-butoxide (1.35 g, 12.0 mmol, 1.2 equiv) and 2-chlorobenzaldehyde (1.41 g, 10.0 mmol, 1.0 equiv) and was obtained after column chromatography (eluent: *n*-pentane) as colorless liquid (1.17 g, 7.11 mmol, 71%, *E*/*Z* ratio 52:48). **^{1}H NMR** (300 MHz, $CDCl_3$) δ 7.69 – 7.30 (m, 4H), 7.30 – 7.13 (m, 4H), 7.10 – 6.26 (m, 6H), 5.38 (dddd, *J* = 16.9, 16.0, 5.5, 4.6 Hz, 2H), 5.32 – 5.14 (m, 2H). **^{13}C NMR** (75 MHz, $CDCl_3$) δ 137.2, 135.6, 133.8, 133.4, 133.0, 132.2, 132.1, 131.3, 130.0, 129.6,

---

[3] A. Lishchynskyi, K. Muñiz, *Chem. Eur. J.* **2012**, *18*, 2212-2216.
[4] D. A. Mundal, K. E. Lutz, R. J. Thomson, *Org. Lett.* **2009**, *11*, 465-468.

128.8, 128.7, 128.6, 127.7, 127.2, 127.0, 126.5, 126.4, 120.5, 118.9. **IR** (ATR) 3060, 2918, 1815, 1684, 1592, 1468, 1434, 1124, 1042, 1003, 967, 908, 749, 699. **MS** (EI+) *m/z* = 164 ($[M]^+$, 11), 129 (100), 149 (5), 102 (13), 77 (10), 51 (17). **HRMS** (EI+) *m*/*z* calculated for $C_{10}H_9Cl$ ($[M]^+$): 164.0393; found: 164.0376.

Methyl 3-(buta-1,3-dienyl)benzoate (1d)

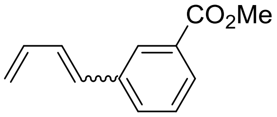

The title compound was synthesized according to GP1 using allyltriphenylphosphonium bromide (4.22 g, 11.0 mmol, 1.1 equiv), potassium *tert*-butoxide (1.35 g, 12.0 mmol, 1.2 equiv) and methyl 3-formylbenzoate (1.64 g, 10.0 mmol, 1.0 equiv) and was obtained after column chromatography (eluent: *n*-pentane/Et_2O 1:1) as colorless liquid (1.27 g, 6.75 mmol, 68%, *E*/*Z* ratio 70:30). **^{1}H NMR** (300 MHz, $CDCl_3$) δ 8.15 – 7.97 (m, 2H), 7.97 – 7.85 (m, 2H), 7.66 – 7.47 (m, 2H), 7.47 – 7.33 (m, 2H), 6.96 – 6.74 (m, 2H), 6.65 – 6.22 (m, 4H), 5.41 (ddd, *J* = 16.9, 6.0, 5.1 Hz, 2H), 5.33 – 5.15 (m, 2H), 3.93 (2x s, 6H, CH_3-*E*/*Z*). **^{13}C NMR** (75 MHz, $CDCl_3$) δ 166.99, 166.97, 137.6 , 137.5, 136.8, 133.3, 132.7, 131.8, 131.7, 130.8, 130.7, 130.6, 130.3, 130.0, 129.2, 128.7, 128.5, 128.3, 128.1, 127.4, 120.6, 118.6, 52.2 (2C). **IR** (ATR) 3008, 2952, 1720, 1598, 1437, 1280, 1195, 1108, 997, 908, 752, 719, 690. **MS** (EI+) *m/z* = 188 ($[M]^+$, 18), 157 (10),129 (100), 128 (79), 102 (4), 77 (9), 129 (100), 149 (5), 102 (13), 77 (10), 51 (17). **HRMS** (EI+) *m*/*z* calculated for $C_{12}H_{12}O_2$ ($[M]^+$): 188.0837; found: 188.0839.

1-(Buta-1,3-dienyl)-4-(trifluoromethyl)benzene (1e)

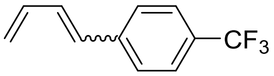

The title compound was synthesized according to GP1 using allyltriphenylphosphonium bromide (4.22 g, 11.0 mmol, 1.1 equiv), potassium *tert*-butoxide (1.35 g, 12.0 mmol, 1.2 equiv) and 4-(trifluoromethyl)benzaldehyde (1.74 g, 10.0 mmol, 1.0 equiv) and was obtained after column chromatography (eluent: *n*-pentane) as colorless liquid (1.33 g, 6.71 mmol, 67%, *E*/*Z* ratio 56:44). **^{1}H NMR** (300 MHz, $CDCl_3$) δ 7.36 – 7.24 (m, 4H), 6.95 – 6.82 (m, 4H), 6.80 – 6.54 (m, 2H), 6.53 – 6.11 (m, 4H), 5.42 – 5.23 (m, 2H), 5.23 – 5.06 (m, 2H). **^{13}C NMR** (75 MHz, $CDCl_3$) δ 141.0 (d, *J* = 21.6 Hz), 140.9 (d, *J* = 21.6 Hz), 136.8, 132.8, 132.7, 132.1, 131.4, 129.8, 129.7, 129.3, 128.9, 126.6, 125.7 (q, *J* = 3.9 Hz), 125.3 (q,

J = 3.8 Hz), 124.4 (q, J = 272.7 Hz), 121.3, 119.5. **^{19}F NMR** (282 MHz, $CDCl_3$) δ −62.53, −62.54.

The ^1J-CF3 coupling of the minor isomer cannot be identified in the ^{13}C NMR spectra. The analytical data are in accordance with the literature.[3]

1-(Buta-1,3-dienyl)-4-methoxybenzene (1f)

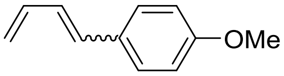

The title compound was synthesized according to GP1 using allyltriphenylphosphonium bromide (4.22 g, 11.0 mmol, 1.1 equiv), potassium *tert*-butoxide (1.35 g, 12.0 mmol, 1.2 equiv) and 4-methoxybenzaldehyde (1.85 g, 10.0 mmol, 1.0 equiv) and was obtained after column chromatography (eluent: *n*-pentane/Et_2O 20:1) as yellow oil (1.43 g, 8.93 mmol, 89%, *E*/*Z* ratio 73:27). **^{1}H NMR** (300 MHz, $CDCl_3$) δ 7.41 – 7.23 (m, 4H), 6.98 – 6.81 (m, 4H), 6.75 – 6.58 (m, 1H), 6.58 – 6.33 (m, 3H), 6.19 (t, J = 11.3 Hz, 1H), 5.41 – 5.24 (m, 2H), 5.24 – 5.05 (m, 2H), 3.82 (2x s, 6H, CH_3-*E*/*Z*). **^{13}C NMR** (75 MHz, $CDCl_3$) δ 159.5, 158.9, 137.5, 133.5, 132.5, 130.4, 130.2, 130.1, 129.5, 128.1, 127.82, 127.80, 119.1, 116.6, 114.2, 113.8, 55.43, 55.41.

The analytical data are in accordance with the literature.[3]

2-(Buta-1,3-dienyl)-1,3,5-trimethoxybenzene (1g)

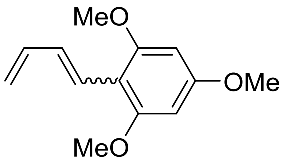

The title compound was synthesized according to GP1 using allyltriphenylphosphonium bromide (2.95 g, 7.70 mmol, 1.1 equiv), potassium *tert*-butoxide (943 mg, 8.40 mmol, 1.2 equiv) and 2,4,6-trimethoxybenzaldehyde (1.37 g, 7.00 mmol, 1.0 equiv) and was obtained after column chromatography (eluent: *n*-pentane/Et_2O 5:1) as yellow oil (1.23 g, 5.57 mmol, 80%, *E*/*Z* ratio 68:32). **Smp**. 41-43 °C. **^{1}H NMR** (300 MHz, $CDCl_3$) δ 7.32 – 7.06 (m, 1H), 6.84 (d, J = 16.0 Hz, 1H), 6.64 – 6.22 (m, 2H), 6.21 – 6.09 (m, 4H), 5.24 (dd, J = 16.4, 10.4 Hz, 2H), 5.05 (dd, J = 8.6, 7.9 Hz, 2H), 4.12 – 3.55 (4x s, 18H, *E*/*Z*-OCH_3). ^{13}C

[3] G. Hilt, M. Danz, *Synthesis* **2008**, *14*, 2257-2263.
[3] G. Hilt, M. Danz, *Synthesis* **2008**, *14*, 2257-2263.

NMR (75 MHz, $CDCl_3$) δ 160.9, 160.4, 159.6, 158.6, 155.8, 140.1, 135.5, 131.7, 131.5, 124.2, 122.1, 117.0, 115.0, 108.1, 90.9, 90.8, 56.0, 55.9, 55.5, 55.4.

The analytical data are in accordance with the literature.[5]

4-(Buta-1,3-dienyl)-1,2-dimethoxybenzene (1h)

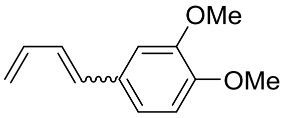

The title compound was synthesized according to GP1 using allyltriphenylphosphonium bromide (8.05 g, 21.0 mmol, 1.1 equiv), potassium *tert*-butoxide (2.47 g, 22.0 mmol, 1.2 equiv) and 3,4-dimethoxybenzaldehyde (3.32 g, 20.0 mmol, 1.0 equiv) and was obtained after column chromatography (eluent: *n*-pentane/Et_2O 15:1) as colorless oil (2.75 g, 14.5 mmol, 73%, *E*/*Z* ratio 58:42). **^{1}H NMR** (300 MHz, $CDCl_3$) δ 6.94 – 6.53 (m, 8H), 6.49 – 6.06 (m, 4H), 5.36 – 4.99 (m, 4H), 3.85 (4x br s, 12H, *E*/*Z*-OCH_3). **^{13}C NMR** (75 MHz, $CDCl_3$) δ 149.3, 149.1, 148.8, 148.5, 137.4, 133.4, 132.8, 130.5, 130.4, 130.3, 129.8, 128.0, 121.9, 120.0, 119.2, 116.7, 112.4, 111.4, 111.2, 109.0, 56.0 (2C).

The analytical data are in accordance with the literature.[6]

1-Bromo-2-(buta-1,3-dienyl)-4,5-dimethoxybenzene (1i)

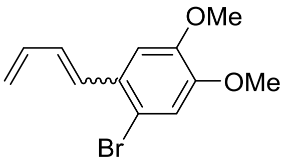

The title compound was synthesized according to GP1 using allyltriphenylphosphonium bromide (4.22 g, 11.0 mmol, 1.1 equiv), potassium *tert*-butoxide (1.35 g, 12.0 mmol, 1.2 equiv) and 2-bromo-4,5-dimethoxybenzaldehyde (2.45 g, 10.0 mmol, 1.0 equiv) and was obtained after column chromatography (eluent: *n*-pentane/Et_2O 3:1) as yellow solid (2.10 g, 7.81 mmol, 78%, *E*/*Z* ratio 73:27). **Smp.** 69-72 °C. **^{1}H NMR** (300 MHz, $CDCl_3$) δ 7.10 – 6.98 (m, 2H), 6.87 (s, 2H), 6.83 – 6.23 (m, 6H), 5.49 – 5.15 (m, 4H), 3.88 (2x br s, 12H, CH_3-*E*/*Z*). **^{13}C NMR** (75 MHz, $CDCl_3$) δ 149.2, 149.1, 148.1, 147.3, 137.3, 133.0, 132.2, 131.3, 131.1, 130.5, 129.9, 129.6, 120.1, 118.0, 115.7, 115.6, 114.5, 114.0, 108.8, 56.4, 56.3, 56.3, 56.2. **IR** (ATR) 3085, 3005, 2934, 2908, 2838, 1597, 1500, 1463, 1430, 1383, 1261, 1210, 1163,

[5] S. B. Bharate, R. Mudududdla, R. Sharma, R. A. Vishwakarma, *Tetrahedron Lett.* **2013**, *54*, 2913-2915.
[6] B. P. Joshi, N. P. Singh, A. Sharma, A. K. Sinha, *Chem. Nat. Comp.* **2005**, *41*, 370-373.

1031, 909, 868, 838. **MS** (EI+) *m/z* = 268 ([M]$^+$, 33), 189 (60), 174 (57), 159 (100), 145 (24), 131 (35), 115 (45), 103 (37), 77 (33). **HRMS** (EI+) *m/z* calculated for $C_{12}H_{13}O_2Br$ ([M]$^+$): 268.0099; found: 268.0095. 1C in the ^{13}C NMR spectra cannot be identified.

3-(Buta-1,3-dienyl)furan (1j)

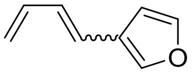

The title compound was synthesized according to GP1 using allyltriphenylphosphonium bromide (2.95 g, 7.70 mmol, 1.1 equiv), potassium *tert*-butoxide (943 mg, 8.40 mmol, 1.2 equiv) and furan-3-carbaldehyde (841 g, 7.00 mmol, 1.0 equiv) and was obtained after column chromatography (eluent: *n*-pentane) as colorless oil (679 mg, 5.65 mmol, 81%, *E*/*Z* ratio 61:39). **^{1}H NMR** (300 MHz, $CDCl_3$) δ 7.61 – 7.34 (m, 4H), 7.01 – 6.79 (m, 1H), 6.65 – 6.36 (m, 4H), 6.25 – 6.08 (m, 3H), 5.43 – 5.07 (m, 4H). **^{13}C NMR** (75 MHz, $CDCl_3$) δ 143.8, 143.2, 141.7, 141.0, 137.2, 133.4, 129.9, 129.7, 124.5, 122.6, 122.6, 120.1, 119.1, 116.8, 111.0, 107.6. **IR** (ATR) 3432, 2923, 2839, 2727, 1759, 1720, 1670, 1633, 1567, 1159, 1072, 1006, 971, 921, 872, 706. **MS** (EI+) *m/z* = 120 ([M]$^+$, 10), 105 (100), 94 (52), 84 (40), 77 (73), 65 (20). **HRMS** (EI+) *m/z* calculated for C_8H_8O ([M]$^+$): 120.0575; found: 120.0578.

2-(Buta-1,3-dienyl)thiophene (1k)

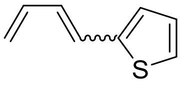

The title compound was synthesized according to GP1 using allyltriphenylphosphonium bromide (4.22 g, 11.0 mmol, 1.1 equiv), potassium *tert*-butoxide (1.35 g, 12.0 mmol, 1.2 equiv) and thiophene-2-carbaldehyde (1.12 g, 10.0 mmol, 1.0 equiv) and was obtained after column chromatography (eluent: *n*-pentane/Et_2O 50:1) as yellow oil (1.02 g, 7.46 mmol, 75%, *E*/*Z* ratio 54:46). **^{1}H NMR** (300 MHz, $CDCl_3$) δ 7.62 – 7.52 (m, 1H), 7.51 – 7.37 (m, 2H), 7.37 – 7.20 (m, 4H), 7.10 – 6.87 (m, 2H), 6.87 – 6.63 (m, 2H), 6.42 (t, *J* = 11.5 Hz, 1H), 5.70 (d, *J* = 16.6 Hz, 1H), 5.65 – 5.52 (m, 2H), 5.44 (d, *J* = 9.9 Hz, 1H). **^{13}C NMR** (75 MHz, $CDCl_3$) δ 142.6, 140.4, 136.8, 133.3, 129.5, 128.6, 128.5, 127.7, 127.1, 126.2, 126.1, 125.8, 124.6, 122.6, 120.2, 117.6.

The analytical data are in accordance with the literature.[7]

[7] E. A. Braude, J. S. Fawcett, D. D. E. Newman, *J. Chem Soc.* **1952**, 4155-4158.

General procedure for the cobalt-catalyzed 1,4-hydrovinylation of aryl-substituted buta-1,3-dienes with allyloxytrimethylsilane and subsequent desilylation with TBAF (GP2)

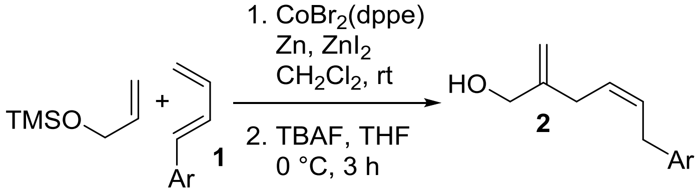

Cobaltdibromo(1,3-bis(diphenylphosphino)ethane) (5-10 mol%), zinc powder (10-20 mol%) and zinc iodide (10-20 mol%) were suspended in CH_2Cl_2 and stirred at r.t. for 20 min. Then the buta-1,3-diene (1.0 equiv) and the alkene (1.2-2.0 equiv) were added and stirred at r.t. until complete conversion was detected by TLC and GC–MS analysis. *n*-Pentane was added, the mixture was filtered through a short pad of silica and concentrated under reduced pressure. The crude material was dissolved in 5 mL THF, TBAF (1 M in THF, 1.1 equiv) was added and the mixture was stirred at 0 °C for 3 h. Upon completion of the reaction 15 mL H_2O were added, the mixture was extracted with 3x 15 mL Et_2O, dried over Na_2SO_4, filtered and concentrated under reduced pressure. The product was obtained after column chromatography (*n*-pentane/Et_2O).

(*Z*)-2-Methylene-6-phenylhex-4-en-1-ol (2a)

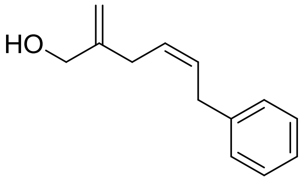

The title compound was prepared according to GP2 using $CoBr_2$(dppe) (185 mg, 0.30 mmol, 10 mol%), zinc powder (39 mg, 0.60 mmol, 20 mol%), zinc iodide (192 mg, 0.60 mmol, 20 mol%), buta-1,3-dienylbenzene (391 mg, 3.00 mmol, 1.0 equiv) and allyloxytrimethylsilane (782 mg, 6.00 mmol, 2.0 equiv). Deprotection was performed using TBAF (3.3 mL, 3.30 mmol, 1.1 equiv, 1 M in THF). The product was obtained after column chromatography (eluent: *n*-pentane/Et_2O 5:1→3:1) as colorless oil (339 mg, 1.80 mmol, 60 %). **^{1}H NMR** (300 MHz, $CDCl_3$) δ 7.30 (ddd, *J* = 8.2, 4.4, 2.6 Hz, 2H), 7.24 – 7.15 (m, 3H), 5.72 (dtt, *J* = 9.9, 7.1, 1.3 Hz, 1H), 5.58 (dtt, *J* = 10.5, 7.4, 1.5 Hz, 1H), 5.08 (d, *J* = 1.4 Hz, 1H), 4.97 (dd, *J* = 2.4, 1.0 Hz, 1H), 4.12 (s, 2H), 3.44 (d, *J* = 7.2 Hz, 2H), 2.95 (d, *J* = 7.4 Hz, 2H), 1.52 (s, 1H). **^{13}C NMR** (75 MHz, $CDCl_3$) δ 147.7, 140.9, 130.3, 128.6, 128.5, 127.3, 126.1, 110.4, 66.1, 33.5, 31.1. **IR** (ATR) 3331, 3064, 3021, 2912, 1650, 1601, 1493, 1447,

1025, 900,737, 697. **MS** (EI+) *m/z* = 188 ([M]$^+$, 7), 170 (18), 157 (27), 155 (32), 142 (14), 129 (47), 117 (70), 91 (100), 79 (32), 77 (18). **HRMS** (EI+) *m*/*z* calculated for $C_{13}H_{16}O$ ([M]$^+$): 188.1201; found: 188.1205.

(*Z*)-6-(4-Bromophenyl)-2-methylenehex-4-en-1-ol (2b)

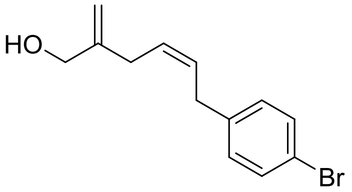

The title compound was prepared according to GP2 using $CoBr_2$(dppe) (185 mg, 0.30 mmol, 10 mol%), zinc powder (39 mg, 0.60 mmol, 20 mol%), zinc iodide (192 mg, 0.60 mmol, 20 mol%), 1-bromo-4-(buta-1,3-dienyl)benzene (621 mg, 3.00 mmol, 1.0 equiv) and allyloxytrimethylsilane (782 mg, 6.00 mmol, 2.0 equiv). Deprotection was performed using TBAF (3.3 mL, 3.30 mmol, 1.1 equiv, 1 M in THF). The product was obtained after column chromatography (eluent: *n*-pentane/Et_2O 5:1→3:1) as colorless oil (568 mg, 2.13 mmol, 71 %). **^{1}H NMR** (300 MHz, $CDCl_3$) δ 7.46 – 7.35 (m, 2H), 7.15 – 6.97 (m, 2H), 5.74 – 5.51 (m, 2H), 5.07 (d, *J* = 1.3 Hz, 1H), 4.94 (d, *J* = 1.3 Hz, 1H), 4.10 (s, 2H), 3.37 (d, *J* = 6.6 Hz, 2H), 2.92 (d, *J* = 6.7 Hz, 2H), 1.50 (s, 1H). **^{13}C NMR** (75 MHz, $CDCl_3$) δ 147.5, 139.8, 131.6, 130.2, 129.6, 127.8, 119.9, 110.5, 66.1, 32.9, 31.0. **IR** (ATR) 3317, 3080, 3015, 2908, 1650, 1484, 1431, 1401, 1315, 1191, 1067, 1012, 901, 831, 799. **MS** (EI+) *m/z* = 266 ([M]$^+$, 7), 250 (11), 235 (19), 195 (19), 169 (40), 154 (28), 141 (29), 129 (47), 116 (100), 90 (29), 79 (31), 71 (13). **HRMS** (EI+) *m*/*z* calculated for $C_{13}H_{15}OBr$ ([M]$^+$): 266.0306; found: 266.0293.

(*Z*)-6-(2-Chlorophenyl)-2-methylenehex-4-en-1-ol (2c)

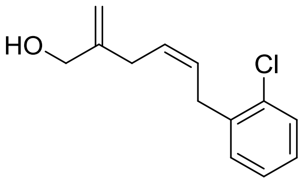

The title compound was prepared according to GP2 using $CoBr_2$(dppe) (185 mg, 0.30 mmol, 10 mol%), zinc powder (39 mg, 0.60 mmol, 20 mol%), zinc iodide (192 mg, 0.60 mmol, 20 mol%), 1-(buta-1,3-dienyl)-2-chlorobenzene (494 mg, 3.00 mmol, 1.0 equiv) and allyloxytrimethylsilane (469 mg, 3.60 mmol, 1.2 equiv). Deprotection was performed using TBAF (3.3 mL, 3.30 mmol, 1.1 equiv, 1 M in THF). The product was obtained after column chromatography (eluent: *n*-pentane/Et_2O 5:1) as colorless oil (541 mg, 2.43 mmol, 81 %).

^{1}H NMR (300 MHz, $CDCl_3$) δ 7.35 (dd, *J* = 7.5, 1.6 Hz, 1H), 7.26 – 7.10 (m, 3H), 5.81 – 5.50 (m, 2H), 5.07 (d, *J* = 1.3 Hz, 1H), 4.95 (d, *J* = 1.3 Hz, 1H), 4.11 (s, 2H), 3.52 (d, *J* = 6.4 Hz, 2H), 2.95 (d, *J* = 6.6 Hz, 2H), 1.50 (s, 1H). **^{13}C NMR** (75 MHz, $CDCl_3$) δ 147.6, 138.4, 134.1, 130.2, 129.6, 128.5, 128.2, 127.6, 127.0, 110.4, 66.1, 31.3, 31.2. **IR** (ATR) 3329, 3068, 3018, 2925, 2866, 1652, 1470, 1440, 1043, 900, 744, 680. **MS** (EI+) *m/z* = 222 ($[M]^+$, 3), 204 (7), 191 (11), 169 (15), 153 (7), 142 (100), 129 (21), 125 (27), 115 (15), 100 (42), 79 (34). **HRMS** (EI+) *m/z* calculated for $C_{13}H_{15}ClO$ ($[M]^+$): 222.0811; found: 222.0802.

(*Z*)-Methyl 3-(5-(hydroxymethyl)hexa-2,5-dienyl)benzoate (2d)

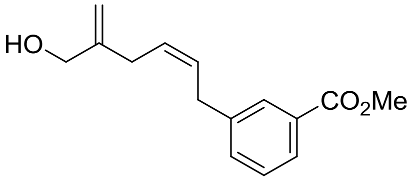

The title compound was prepared according to GP2 using $CoBr_2$(dppe) (179 mg, 0.29 mmol, 10 mol%), zinc powder (37 mg, 0.57 mmol, 20 mol%), zinc iodide (182 mg, 0.57 mmol, 20 mol%), methyl 3-(buta-1,3-dienyl)benzoate (536 mg, 2.85 mmol, 1.0 equiv) and allyloxytrimethylsilane (445 mg, 3.42 mmol, 1.2 equiv). Deprotection was performed using TBAF (3.1 mL, 3.14 mmol, 1.1 equiv, 1 M in THF). The product was obtained after column chromatography (eluent: *n*-pentane/Et_2O 3:1) as yellow oil (354 mg, 1.44 mmol, 51%). **^{1}H NMR** (300 MHz, $CDCl_3$) δ 7.86 (dd, *J* = 6.0, 1.7 Hz, 2H), 7.43 – 7.29 (m, 2H), 5.78 – 5.65 (m, 1H), 5.65 – 5.52 (m, 1H), 5.08 (d, *J* = 1.3 Hz, 1H), 4.97 (t, *J* = 8.9 Hz, 1H), 4.13 (d, *J* = 9.8 Hz, 2H), 3.91 (s, 3H), 3.47 (d, *J* = 6.9 Hz, 2H), 2.95 (d, *J* = 7.1 Hz, 2H), 1.69 (s, 1H). **^{13}C NMR** (75 MHz, $CDCl_3$) δ 167.4, 147.6, 141.3, 133.1, 130.4, 129.7, 129.6, 128.6, 128.0, 127.4, 110.6, 66.1, 52.2, 33.3, 31.1. **IR** (ATR) 3433, 3016, 2951, 2910, 1716, 1438, 1279, 1196, 1106, 1079, 1028, 987, 899, 807, 746, 690. **MS** (EI+) *m/z* = 246 ($[M]^+$, 1), 228 (32), 214 (100), 200 (5), 186 (18), 175 (23), 169 (50), 157 (15), 149 (50), 141 (17), 129 (81), 115 (47), 91 (51), 79 (52), 59 (22). **HRMS** (EI+) *m/z* calculated for $C_{15}H_{18}O_3$ ($[M]^+$): 246.1256; found: 246.1257.

(*Z*)-2-Methylene-6-(4-(trifluoromethyl)phenyl)hex-4-en-1-ol (2e)

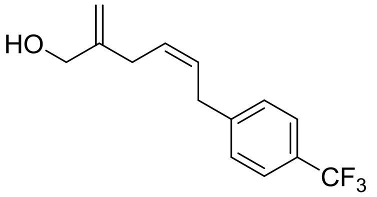

The title compound was prepared according to GP2 using $CoBr_2$(dppe) (37 mg, 0.06 mmol, 5 mol%), zinc powder (8 mg, 0.12 mmol, 10 mol%), zinc iodide (38 mg, 0.12 mmol, 10 mol%), 1-(buta-1,3-dienyl)-4-(trifluoromethyl)benzene (231 mg, 1.17 mmol, 1.0 equiv) and allyloxytrimethylsilane (182 mg, 1.40 mmol, 1.2 equiv). Deprotection was performed using TBAF (2.0 mL, 2.00 mmol, 1.7 equiv, 1 M in THF). The product was obtained after column chromatography (eluent: *n*-pentane/Et_2O 3:1) as colorless oil (208 mg, 0.81 mmol, 69 %). **^{1}H NMR** (300 MHz, $CDCl_3$) δ 7.39 (d, *J* = 8.1 Hz, 2H), 7.21 – 7.04 (m, 2H), 5.72 – 5.32 (m, 2H), 4.93 (d, *J* = 1.1 Hz, 1H), 4.80 (d, *J* = 1.2 Hz, 1H), 3.96 (s, 2H), 3.33 (d, *J* = 6.4 Hz, 2H), 2.78 (d, *J* = 6.5 Hz, 2H), 1.35 (s, 1H). **^{13}C NMR** (75 MHz, $CDCl_3$) δ 147.4, 145.0 (q, *J* = 1.4 Hz), 129.0, 128.7, 128.4 (q, *J* = 31.3 Hz), 128.2, 125.4 (q, *J* = 3.8 Hz), 124.5 (q, *J* = 270.9 Hz), 110.4, 65.9, 33.3, 30.9. **^{19}F NMR** (282 MHz, $CDCl_3$) δ –62.3. **IR** (ATR) 3363, 3019, 2926, 1617, 1419, 1323, 1162, 1114, 1064, 1019, 974, 902, 842, 818, 736. **MS** (EI+) *m/z* = 256 ($[M]^+$, 4), 238 (29), 223 (24), 210 (13), 197 (17), 177 (26), 165 (34), 159 (54), 141 (17), 129 (65), 115 (36), 109 (26), 79 (100). **HRMS** (EI+) *m/z* calculated for $C_{14}H_{15}OF_3$ ($[M]^+$): 256.1075; found: 256.1074.

(*Z*)-6-(4-Methoxyphenyl)-2-methylenehex-4-en-1-ol (2f)

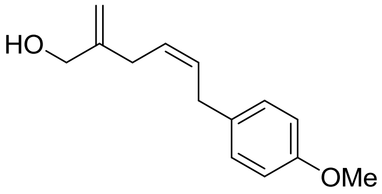

The title compound was prepared according to GP2 using $CoBr_2$(dppe) (185 mg, 0.30 mmol, 10 mol%), zinc powder (39 mg, 0.60 mmol, 20 mol%), zinc iodide (192 mg, 0.60 mmol, 20 mol%), 1-(buta-1,3-dienyl)-4-methoxybenzene (481 mg, 3.00 mmol, 1.0 equiv) and allyloxytrimethylsilane (782 mg, 6.00 mmol, 2.0 equiv). Deprotection was performed using TBAF (3.3 mL, 3.30 mmol, 1.1 equiv, 1 M in THF). The product was obtained after column chromatography (eluent: *n*-pentane/Et_2O 3:1→1:1) as colorless oil (570 mg, 2.61 mmol, 87 %). **^{1}H NMR** (300 MHz, $CDCl_3$) δ 7.10 (d, *J* = 8.6 Hz, 2H), 6.90 – 6.76 (m, 2H), 5.74 –

5.64 (m, 1H), 5.60 – 5.50 (m, 1H), 5.07 (s, 1H), 4.96 (d, J = 1.3 Hz, 1H), 4.11 (d, J = 4.6 Hz, 2H), 3.79 (s, 3H), 3.37 (d, J = 7.1 Hz, 2H), 2.93 (d, J = 7.3 Hz, 2H), 1.48 (t, J = 5.7 Hz, 1H). **^{13}C NMR** (75 MHz, $CDCl_3$) δ 158.1, 147.8, 132.9, 130.7, 129.4, 127.0, 114.1, 110.3, 66.1, 55.4, 32.6, 31.1. **IR** (ATR) 3354, 3008, 2906, 2836, 1650, 1608, 1508, 1457, 1242, 1176, 1033, 899, 815, 772, 690. **MS** (EI+) *m/z* = 218 ([M]$^+$, 8), 200 (4), 187 (29), 172 (8), 159 (19), 147 (100), 134 (8), 121 (42), 108 (24), 91 (15). **HRMS** (ESI+) *m*/*z* calculated for $C_{14}H_{18}O_2H$ ([M+H]$^+$): 219.1381; found: 219.1380.

(*Z*)-2-Methylene-6-(2,4,6-trimethoxyphenyl)hex-4-en-1-ol (2g)

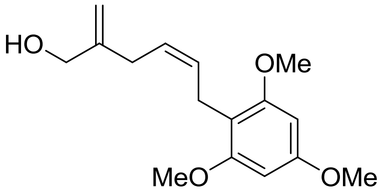

The title compound was prepared according to GP2 using $CoBr_2$(dppe) (161 mg, 0.26 mmol, 5 mol%), zinc powder (33 mg, 0.51 mmol, 10 mol%), zinc iodide (163 mg, 0.51 mmol, 10 mol%), 2-(buta-1,3-dienyl)-1,3,5-trimethoxybenzene (1.12 g, 5.09 mmol, 1.0 equiv) and allyloxytrimethylsilane (796 mg, 6.11 mmol, 1.2 equiv). Deprotection was performed using TBAF (6.1 mL, 6.11 mmol, 1.1 equiv, 1 M in THF). The product was obtained after column chromatography (eluent: *n*-pentane/Et_2O 1:1) as colorless oil (570 mg, 1.22 mmol, 64%), which solidified upon cooling. **Smp**. 55-56 °C. **^{1}H NMR** (300 MHz, $CDCl_3$) δ 6.13 (s, 2H), 5.59 – 5.48 (m, 1H), 5.41 – 5.28 (m, 1H), 5.06 – 5.00 (m, 1H), 4.94 (d, J = 1.3 Hz, 1H), 4.11 (d, J = 4.6 Hz, 2H), 3.80 (2x s, 9H), 3.34 (dd, J = 7.1, 1.3 Hz, 2H), 3.02 (d, J = 7.3 Hz, 2H), 1.68 (s, 1H). **^{13}C NMR** (75 MHz, $CDCl_3$) δ 159.6, 158.7, 148.4, 130.7, 125.8, 109.9, 109.8, 90.9, 66.1, 55.9, 55.5, 31.4, 21.1. **IR** (ATR) 3391, 2999, 2938, 2837, 1595, 1497, 1455, 1437, 1204, 1147, 1121, 1059, 1042, 950, 897, 810. **HRMS** (ESI+) *m*/*z* calculated for $C_{16}H_{22}O_4Na$ ([M+Na]$^+$): 301.1410; found: 301.1410.

(*Z*)-6-(3,4-Dimethoxyphenyl)-2-methylenehex-4-en-1-ol (2h)

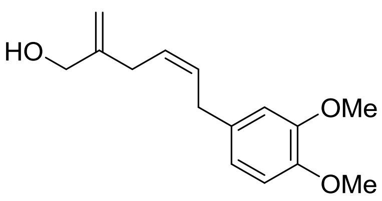

The title compound was prepared according to GP2 using $CoBr_2$(dppe) (309 mg, 0.50 mmol, 5 mol%), zinc powder (65 mg, 1.00 mmol, 10 mol%), zinc iodide (319 mg, 1.00 mmol,

10 mol%), 4-(buta-1,3-dienyl)-1,2-dimethoxybenzene (1.90 g, 10.0 mmol, 1.0 equiv) and allyloxytrimethylsilane (1.56 mg, 12.0 mmol, 1.2 equiv). Deprotection was performed using TBAF (11 mL, 11.0 mmol, 1.1 equiv, 1 M in THF). The product was obtained after column chromatography (eluent: *n*-pentane/Et_2O 1:1) as yellow oil (1.67 mg, 6.72 mmol, 67%). **^{1}H NMR** (300 MHz, $CDCl_3$) δ 6.83 – 6.75 (m, 1H), 6.75 – 6.65 (m, 2H), 5.69 (dtt, *J* = 9.7, 7.0, 1.2 Hz, 1H), 5.56 (dtt, *J* = 10.5, 7.4, 1.4 Hz, 1H), 5.07 (d, *J* = 1.3 Hz, 1H), 5.02 – 4.87 (m, 1H), 4.10 (d, *J* = 6.5 Hz, 2H), 3.85 (s, 3H), 3.84 (s, 3H), 3.35 (t, *J* = 9.5 Hz, 2H), 2.93 (d, *J* = 7.3 Hz, 2H), 1.61 (s, 1H). **^{13}C NMR** (75 MHz, $CDCl_3$) δ 149.1, 147.8, 147.5, 133.5, 130.5, 127.1, 120.3, 112.0, 111.6, 110.3, 66.1, 56.1, 56.0, 33.0, 31.0. **IR** (ATR) 3488, 3006, 2922, 2848, 1651, 1593, 1511, 1458, 1258, 1232, 1142, 1025, 898, 848, 806, 756, 691. **MS** (EI+) *m/z* = 248 ($[M]^+$, 66), 230 (72), 217 (29), 199 (31), 189 (22), 177 (100), 166 (42), 152 (18), 151 (90), 138 (85), 128 (26), 115 (33), 107 (11), 95 (16), 91 (21), 77 (26). **HRMS** (EI+) *m/z* calculated for $C_{15}H_{20}O_3$ ($[M]^+$): 248.1412; found: 248.1397.

(*Z*)-6-(2-Bromo-4,5-dimethoxyphenyl)-2-methylenehex-4-en-1-ol (2i)

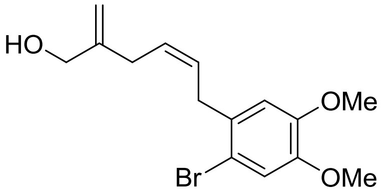

The title compound was prepared according to GP2 using $CoBr_2$(dppe) (80 mg, 0.13 mmol, 5 mol%), zinc powder (16 mg, 0.26 mmol, 10 mol%), zinc iodide (83 mg, 0.26 mmol, 10 mol%), 1-bromo-2-(buta-1,3-dienyl)-4,5-dimethoxybenzene (700 mg, 2.60 mmol, 1.0 equiv) and allyloxytrimethylsilane (594 mg, 3.12 mmol, 1.2 equiv). Deprotection was performed using TBAF (3.1 mL, 3.12 mmol, 1.2 equiv, 1 M in THF). The product was obtained after column chromatography (eluent: *n*-pentane/Et_2O 3:2) as colorless oil (507 mg, 1.55 mmol, 60%). **^{1}H NMR** (300 MHz, $CDCl_3$) δ 6.99 (s, 1H), 6.71 (s, 1H), 5.71 – 5.51 (m, 2H), 5.06 (d, *J* = 1.0 Hz, 1H), 4.94 (d, *J* = 1.1 Hz, 1H), 4.10 (s, *J* = 9.3 Hz, 2H), 3.83 (s, 3H), 3.82 (s, 3H), 3.49 – 3.37 (m, 2H), 2.93 (d, *J* = 5.8 Hz, 2H), 1.76 (s, 1H). **^{13}C NMR** (75 MHz, $CDCl_3$) δ 148.6, 148.1, 147.7, 132.1, 129.0, 127.9, 115.7, 114.1, 112.9, 110.3, 66.1, 56.3, 56.1, 33.5, 31.2. **IR** (ATR) 3511, 3425, 3080, 3008, 2907, 2842, 1651, 1602, 1502, 1440, 1379, 1336, 1255, 1213, 1157, 1028, 964, 899, 852, 801, 758, 693, 610. **MS** (EI+) *m/z* = 326 ($[M]^+$, 16), 231 (13), 216 (12), 176 (52), 151 (11), 115 (8), 98 (11), 73 (36), 61 (37). **HRMS** (EI+) *m/z* calculated for $C_{15}H_{19}BrO_3$ ($[M]^+$): 326.0518; found:326.0508.

(Z)-6-(Furan-2-yl)-2-methylenehex-4-en-1-ol (2j)

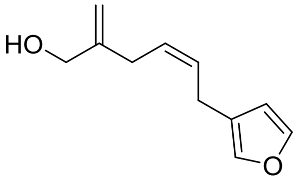

The title compound was prepared according to GP2 using $CoBr_2$(dppe) (228 mg, 0.37 mmol, 5 mol%), zinc powder (48 mg, 0.73 mmol, 10 mol%), zinc iodide (233 mg, 0.73 mmol, 10 mol%), 3-(buta-1,3-dienyl)furan (882 mg, 7.34 mmol, 1.0 equiv) and allyloxytrimethylsilane (1.15 g, 8.81 mmol, 1.2 equiv). Deprotection was performed using TBAF (8.0 mL, 1.09 mmol, 1.1 equiv, 1 M in THF). The product was obtained after column chromatography (eluent: *n*-pentane/Et_2O 3:1→1:1) as yellow oil (772 mg, 4.33 mmol, 59%). (the product decomposes slowly at room temperature!). **^{1}H NMR** (300 MHz, $CDCl_3$) δ 7.35 (s, 1H), 7.24 (d, J = 14.7 Hz, 1H), 6.27 (s, 1H), 5.76 – 5.63 (m, 1H), 5.54 (dt, J = 9.0, 7.4 Hz, 1H), 5.06 (s, 1H), 4.94 (s, 1H), 4.10 (s, 2H), 3.21 (d, J = 7.0 Hz, 2H), 2.90 (d, J = 7.1 Hz, 2H), 1.44 (s, 1H). **^{13}C NMR** (75 MHz, $CDCl_3$) δ 147.6, 143.1, 139.1, 129.4, 127.4, 123.9, 111.1, 110.4, 66.1, 31.0, 23.0. **IR** (ATR) 3319, 3016, 2912, 1650, 1501, 1432, 1155, 1057, 1022, 984, 901, 873, 777. **MS** (EI+) m/z = 178 ($[M]^+$, 3), 160 (15), 147 (23), 131 (25), 117 (36), 107 (27), 91 (100), 79 (85), 77 (69), 67 (18), 57 (15). **HRMS** (EI+) m/z calculated for $C_{11}H_{14}O_2$ ($[M]^+$): 178.0994; found: 178.0990.

(Z)-2-Methylene-6-(thiophen-2-yl)hex-4-en-1-ol (2k)

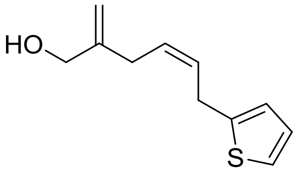

The title compound was prepared according to GP2 using $CoBr_2$(dppe) (228 mg, 0.26 mmol, 5 mol%), zinc powder (48 mg, 0.51 mmol, 10 mol%), zinc iodide (233 mg, 0.51 mmol, 10 mol%), 2-(buta-1,3-dienyl)thiophene (700 mg, 5.14 mmol, 1.0 equiv) and allyloxytrimethylsilane (805 mg, 6.18 mmol, 1.2 equiv). Deprotection was performed using TBAF (5.7 mL, 5.65 mmol, 1.1 equiv, 1 M in THF). The product was obtained after column chromatography (eluent: *n*-pentane/Et_2O 5:1) as orange oil (869 mg, 4.47 mmol, 87 %). **^{1}H NMR** (300 MHz, $CDCl_3$) δ 7.13 (dd, J = 5.1, 1.1 Hz, 1H), 6.93 (dd, J = 5.1, 3.5 Hz, 1H), 6.87 – 6.73 (m, 1H), 5.85 – 5.68 (m, 1H), 5.68 – 5.51 (m, 1H), 5.08 (s, 1H), 4.96 (s, 1H), 4.11 (s, 2H), 3.61 (d, J = 7.2 Hz, 2H), 2.93 (d, J = 7.3 Hz, 2H), 1.50 (s, 1H).**^{13}C NMR** (75 MHz,

$CDCl_3$) δ 147.4, 143.8, 129.4, 127.9, 127.0, 124.4, 123.6, 110.6, 66.1, 31.0, 27.8. **IR** (ATR) 3329, 3076, 3014, 2910, 1651, 1434, 1286, 1237, 1027, 899, 847, 822, 763, 691. **MS** (EI+) *m/z* = 194 ($[M]^+$, 7), 176 (15), 163 (24), 135 (33), 123 (100), 110 (13), 97 (66), 91 (19), 79 (26). **HRMS** (EI+) *m/z* calculated for $C_{11}H_{14}OS$ ($[M]^+$): 194.0765; found: 194,0753.

General procedure for the cobalt-catalyzed 1,4-hydrovinylation of alkenes with buta-1,3-dienes (GP3)[8]

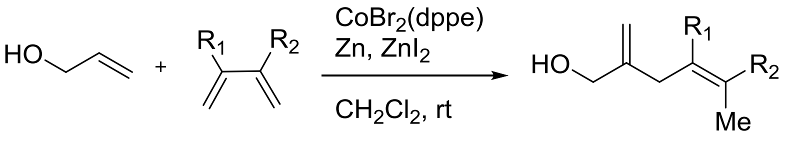

Cobaltdibromo(1,3-bis(diphenylphosphino)ethane) (5 mol%), zinc powder (10 mol%) and zinc iodide (10 mol%) were suspended in CH_2Cl_2 and stirred at r.t. for 20 min. Then, the buta-1,3-diene (1.0 equiv) and allyl alcohol (1.2-1.5 equiv) were added and stirred at r.t. until complete conversion was detected by TLC and GC-MS analysis. *n*-Pentane was added and the mixture was filtered through a short pad of silica. The solvent was evaporated and the crude product was purified by column chromatography to give the desired 1,4-diene.

(*Z*)-2-Methylenehex-4-en-1-ol (2l)

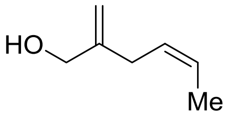

The title compound was prepared according to GP3 using $CoBr_2$(dppe) (154 mg, 0.25 mmol, 5 mol%), zinc powder (30 mg, 0.50 mmol, 10 mol%), zinc iodide (160 mg, 0.50 mmol, 10 mol%), 1,3-butadiene (1.7 mL, 20 wt% in toluene, 5.00 mmol, 1.0 equiv) and allylalcohol (349 mg, 6.00 mmol, 1.2 equiv) in 3 mL CH_2Cl_2. The product was obtained after column chromatography (eluent: *n*-pentane/Et_2O 100:1→10:1) as colorless oil (517 mg, 4.61 mmol, 92%). **^{1}H NMR** (300 MHz, $CDCl_3$) δ 5.66 – 5.50 (m, 1H), 5.50 – 5.35 (m, 1H), 5.02 (d, *J* = 0.9 Hz, 1H), 4.89 (d, *J* = 1.0 Hz, 1H), 4.07 (s, 2H), 2.81 (d, *J* = 7.3 Hz, 2H), 1.84 (br s, 1H), 1.63 (d, *J* = 6.7 Hz, 3H). **^{13}C NMR** (75 MHz, $CDCl_3$) δ 147.9, 127.3, 126.0, 109.9, 66.0, 30.8, 12.8. **IR** (ATR) 3338, 3080, 3017, 2918, 2862, 1651, 1437, 1403, 1026, 898, 693. **MS** (EI+) *m/z* = 111 ($[M]^+$, 3), 94 (82), 81 (29), 79 (100), 77 (71), 67 (38), 57 (56), 55 (65), 41 (77), 39 (70). **HRMS** (EI+) *m/z* calculated for $C_7H_{12}O$ ($[M]^+$): 112.0888; found: 112.0885.

---

[8] M. Arndt, M. Dindaroğlu, H. G. Schmalz, G. Hilt, *Org. Lett.* **2011**, *13*, 6236-6239.

4,5-Dimethyl-2-methylenehex-4-en-1-ol (2m)

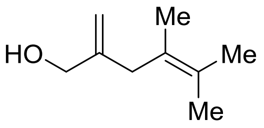

The title compound was prepared according to GP3 using $CoBr_2$(dppe) (463 mg, 0.75 mmol, 5 mol%), zinc powder (98 mg, 1.50 mmol, 10 mol%), zinc iodide (479 mg, 1.50 mmol, 10 mol%), allylalcohol (1.31 g, 22.5 mmol, 1.2 equiv) and 2,3-dimethylbuta-1,3-diene (1.23 g, 15.0 mmol, 1.0 equiv) in 10 mL CH_2Cl_2. The product was obtained after column chromatography (eluent: *n*-pentane/Et_2O 6:1) as colorless oil (1.89 g, 13.5 mmol, 90%). **^{1}H NMR** (300 MHz, $CDCl_3$) δ 5.01 (s, 1H), 4.81 (s, 1H), 4.01 (s, 2H), 2.78 (s, 2H), 1.76 (s, 1H), 1.67 (s, 3H), 1.65 (s, 3H), 1.60 (s, 3H). **^{13}C NMR** (75 MHz, $CDCl_3$) δ 147.2, 126.6, 124.7, 109.9, 66.0, 38.4, 20.7, 20.5, 18.4. **IR** (ATR) 3319, 2987, 2913, 2860, 1650, 1443, 1375, 1317, 1055, 1022, 657. **MS** (EI+) *m/z* = 140 ([M]$^+$, 33), 122 (45), 108 (18), 91 (81), 79 (82), 67 (69), 55 (75), 41 (89). **HRMS** (EI+) *m*/*z* calculated for $C_9H_{16}O$ ([M]$^+$): 140.1201; found: 140.1203.

5-Methyl-2-methylenehex-4-en-1-ol (2n) and (*Z*)-4-methyl-2-methylenehex-4-en-1-ol (2o)

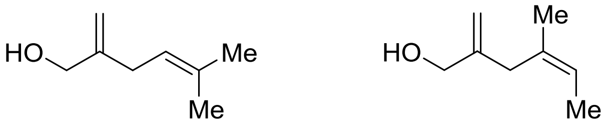

The title compound was prepared according to GP3 using $CoBr_2$(dppe) (216 mg, 0.35 mmol, 5 mol%), zinc powder (46 mg, 0.70 mmol, 10 mol%), zinc iodide (223 mg, 0.70 mmol, 10 mol%), isoprene (477 mg, 7.00 mmol, 1.0 equiv) and Allylalcohol (610 mg, 10.5 mmol, 1.5 equiv) in 5 mL CH_2Cl_2. The product was obtained after column chromatography (eluent: *n*-pentane/Et_2O 5:1→3:1) as colorless oil (786 mg, 6.23 mmol, 89%, ratio **2m**:**2o** 79:21). NMR-data of the major isomer (**2m**): **^{1}H NMR** (300 MHz, $CDCl_3$) δ 5.17 (m, 1H), 5.05 – 4.95 (m, 1H), 4.87 (dd, *J* = 2.4, 1.0 Hz, 1H), 4.06 (d, *J* = 3.6 Hz, 2H), 2.75 (d, *J* = 7.3 Hz, 2H), 1.72 (d, *J* = 1.0 Hz, 3H), 1.62 (s, *J* = 3.5 Hz, 3H), 1.27 – 1.11 (m, 1H). **^{13}C NMR** (75 MHz, $CDCl_3$) δ 148.4, 133.6, 121.2, 109.4, 65.9, 31.9, 25.7, 17.6. **IR** (ATR) 3309, 3080, 2968, 2920, 2863, 1650, 1443, 1377, 1101, 1014, 900, 817. **MS** (EI+) *m/z* = 126 ([M]$^+$, 3), 111 (5), 108 (50), 95 (9), 93 (100), 77 (36), 67 (26), 55(24). **HRMS** (EI+): *m*/*z* calculated for $C_8H_{14}O$ ([M]$^+$): 126.1045; found: 126.1047.

General procedure for the synthesis of 1,4-dienols containing secondary alcohol functions (GP4)

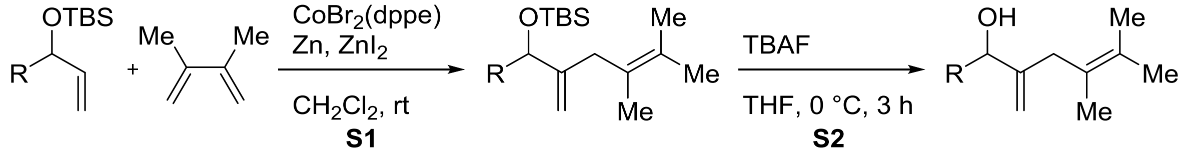

S1: Cobaltdibromo(1,3-bis(diphenylphosphino)ethane) (10 mol%), zinc powder (20 mol%) and zinc iodide (20 mol%) were suspended in CH_2Cl_2 and stirred at r.t. for 20 min. Then the alkene (1.0 equiv) and 2,3-dimethylbuta-1,3-diene (1.2 equiv) were added and stirred at r.t. until complete conversion was detected by TLC and GC−MS analysis. *n*-Pentane was added, the mixture was filtered through a short pad of silica (Et_2O) and concentrated under reduced pressure to give the TBS-protected 1,4-diene. **S2:** The 1,4-diene was dissolved in THF, TBAF (1 M in THF, 1.2 equiv) was added and the mixture was stirred at r.t. for 3 h. Upon completion of the reaction 15 mL H_2O were added, the mixture was extracted with 3x 15 mL Et_2O, dried over Na_2SO_4, filtered and concentrated under reduced pressure. The product was obtained after column chromatography (*n*-pentane/Et_2O).

***tert*-Butyl(2,3-dimethyl-5-methylenetridec-2-en-6-yloxy)dimethylsilane**

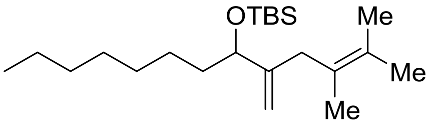

The title compound was prepared according to GP4-S1 using $CoBr_2$(dppe) (124 mg, 0.20 mmol, 10 mol%), zinc powder (26 mg, 0.40 mmol, 20 mol%), zinc iodide (128 mg, 0.40 mmol, 20 mol%), *tert*-butyl(dec-1-en-3-yloxy)dimethylsilane (541 mg, 2.00 mmol, 1.0 equiv) and 2,3-dimethylbuta-1,3-diene (197 mg, 2.40 mmol, 1.2 equiv). The product was obtained after the usual workup as colorless oil (689 mg, 1.95 mmol, 98%). **^{1}H NMR** (300 MHz, $CDCl_3$) δ 4.91 (d, J = 1.2 Hz, 1H), 4.62 (dd, J = 3.7, 1.7 Hz, 1H), 4.04 (t, J = 6.2 Hz, 1H), 2.70 (s, 2H), 1.69 (s, 3H), 1.61 (s, 6H), 1.53 – 1.44 (m, 2H), 1.27 (s, 10H), 0.92 – 0.85 (m, 12H), 0.03 (s, J = 5.0 Hz, 3H), 0.00 (s, J = 3.0 Hz, 3H). **^{13}C NMR** (75 MHz, $CDCl_3$) δ 149.5, 126.3, 125.2, 109.4, 76.5, 36.8, 36.1, 32.0, 29.7, 29.4, 26.0, 25.8, 22.8, 20.6, 20.5, 18.9, 18.4, 14.3, −4.47, −4.86. The analytical data are in accordance with the literature.[9]

[9] H. Menz, S. Kirsch, *Org. Lett.* **2009**, *11*, 5634–5637.

2,3-Dimethyl-5-methylenetridec-2-en-6-ol (2p)

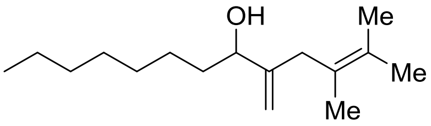

The title compound was prepared according to GP4-S2 using *tert*-butyl(2,3-dimethyl-5-methylenetridec-2-en-6-yloxy)dimethylsilane (624 mg, 1.77 mmol, 1.0 equiv) and TBAF (2.1 mL, 2.12 mmol, 1.2 equiv, 1 M in THF) in 1 mL THF. The product was obtained after column chromatography (eluent: *n*-pentane/Et_2O 6:1) as colorless oil (252 mg, 1.06 mmol, 60%). **^{1}H NMR** (300 MHz, $CDCl_3$) δ 5.00 (s, 1H), 4.73 (s, 1H), 4.06 (dd, *J* = 11.8, 4.9 Hz, 1H), 2.85 – 2.65 (m, 2H), 1.69 (s, 3H), 1.64 (s, 3H), 1.61 (s, 3H), 1.58 – 1.50 (m, 1H), 1.47 (d, *J* = 4.0 Hz, 1H), 1.43 – 1.14 (m, 10H), 0.87 (t, *J* = 5.4 Hz, 3H). **^{13}C NMR** (75 MHz, $CDCl_3$) δ 149.7, 126.7, 124.9, 110.0, 75.6, 36.8, 35.6, 32.0, 29.7, 29.4, 25.9, 22.8, 20.7, 20.5, 18.7, 14.2. **IR** (ATR) 3344, 2921, 2858, 1644, 1453, 1378, 1310, 1227, 1117, 1023, 901, 721, 641, 551. **MS** (EI+) *m/z* = 235 ($[M]^+$, 2), 220 (11), 213 (23), 135 (86), 122 (66), 121 (71), 107 (100), 93 (52), 55 (61), 43 (55), 41 (82). **HRMS** (EI+) *m*/*z* calculated for $C_{16}H_{30}O$ ($[M]^+$): 238.2297; found:238.2306.

(1-(4-Bromophenyl)-4,5-dimethyl-2-methylenehex-4-enyloxy)(*tert*-butyl)dimethylsilane

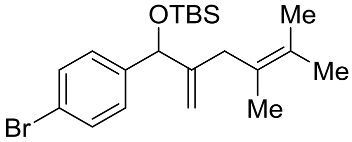

The title compound was prepared according to GP4-S1 using $CoBr_2$(dppe) (62 mg, 0.10 mmol, 5 mol%), zinc powder (13 mg, 0.20 mmol, 10 mol%), zinc iodide (64 mg, 0.20 mmol, 10 mol%), (1-(4-bromophenyl)-4,5-dimethyl-2-methylenehex-4-enyloxy)(*tert*-butyl)-dimethylsilane (654 mg, 2.00 mmol, 1.0 equiv) and 2,3-dimethylbuta-1,3-diene (197 mg, 2.40 mmol, 1.2 equiv). The product was obtained after the usual workup as colorless oil (776 mg, 1.90 mmol, 95%). **^{1}H NMR** (300 MHz, $CDCl_3$) δ 7.42 (d, *J* = 8.4 Hz, 2H), 7.23 (d, *J* = 8.1 Hz, 2H), 5.16 (s, 1H), 5.11 (s, 1H), 4.73 (s, 1H), 2.61 (d, *J* = 16.6 Hz, 1H), 2.41 (d, *J* = 16.6 Hz, 1H), 1.63 (s, 3H), 1.46 (s, 6H), 0.92 (s, 9H), 0.07 (s, 3H), -0.01 (s, 3H).**^{13}C NMR** (75 MHz, $CDCl_3$) δ 149.0, 142.6, 131.1, 128.1, 126.6, 124.6, 120.8, 110.6, 78.0, 35.5, 26.0, 20.6, 20.4, 18.7, 18.5, –4.7, –4.9. **IR** (ATR) 2952, 2861, 1478, 1396, 1254, 1074, 1008, 963, 902, 865, 838, 781, 732, 674, 526. **MS** (EI+) *m/z* = 408 ($[M]^+$, 2), 329 (4), 393 (3), 278 (12),

261 (12), 182 (14), 167 (8), 147 (10), 107 (8), 83 (61), 75 (100), 73 (16), 55 (14), 41 (12). **HRMS** (EI+) *m/z* calculated for $C_{21}H_{33}BrOSi$ ($[M]^+$): 408.1484; found: 408.1484.

1-(4-Bromophenyl)-4,5-dimethyl-2-methylenehex-4-en-1-ol (2q)

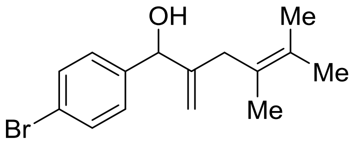

The title compound was prepared according to GP4-S2 using (1-(4-bromophenyl)-4,5-dimethyl-2-methylenehex-4-enyloxy)(*tert*-butyl)dimethylsilane (685 mg, 1.67 mmol, 1.0 equiv) and TBAF (2.0 mL, 2.01 mmol, 1.2 equiv, 1 M in THF) in 1 mL THF. The product was obtained after column chromatography (eluent: *n*-pentane/Et_2O 3:1) as colorless oil (300 mg, 1.02 mmol, 61%). **^{1}H NMR** (300 MHz, $CDCl_3$) δ 7.50 – 7.44 (m, 2H), 7.30 – 7.22 (m, 2H), 5.26 – 5.20 (m, 1H), 5.10 (d, J = 3.8 Hz, 1H), 4.91 (d, J = 1.6 Hz, 1H), 2.60 (q, J = 16.0 Hz, 2H), 1.99 (t, J = 9.1 Hz, 1H), 1.65 (s, 3H), 1.52 (d, J = 0.9 Hz, 3H), 1.50 (s, 3H). **^{13}C NMR** (75 MHz, $CDCl_3$) δ 148.5, 141.2, 131.6, 128.5, 127.1, 124.5, 121.6, 111.4, 76.9, 37.1, 20.7, 20.5, 18.5. **IR** (ATR) 3342, 2984, 2912, 2860, 1644, 1589, 1483, 1441, 1401, 1326, 1228, 1183, 1107, 1066, 1038, 1011, 961, 907, 822, 732, 660, 609, 551, 527, 461. **MS** (EI+) *m/z* = 294 ($[M]^+$, 21), 263 (32), 261 (31), 182 (100), 167 (42), 145 (39), 145 (89), 77 (62). **HRMS** (EI+) *m/z* calculated for $C_{15}H_{19}BrO$ ($[M]^+$): 294,0619; found: 294.0606.

General procedure for the electrochemical selenoalkoxylation of 1,4-dienols (GP5)

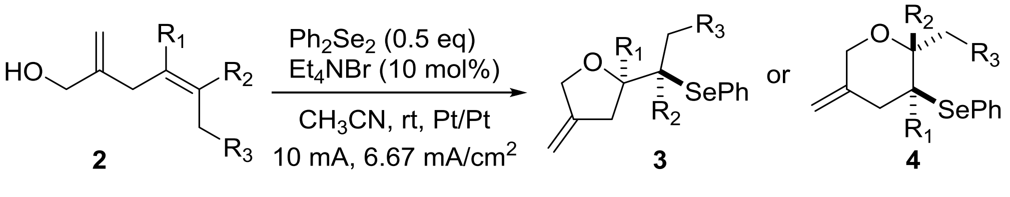

An undivided electrolysis cell was charged with diphenyl diselenide (78 mg, 0.25 mmol, 0.5 equiv), tetraethylammonium bromide (11 mg, 0.05 mmol, 0.1 equiv), the 1,4-diene (0.50 mmol, 1.0 equiv) and 10 mL CH_3CN. Then, the cell was equipped with a platinium plate anode and cathode and electrolyzed under a constant current (10 mA, 6.67 mA/cm^2) at 20 °C until completion was detected by TLC and GC–MS analysis. Then, 15 mL H_2O were added, the mixture was extracted with 3x 15 mL Et_2O, dried over Na_2SO_4 and the solvent was

removed under reduced pressure. The product was obtained after column chromatography (n-pentane/Et_2O).

***threo*-4-Methylene-2-(2-phenyl-1-(phenylselanyl)ethyl)tetrahydrofuran (3a)**

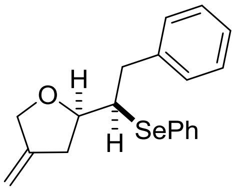

The title compound was prepared according to GP5 using diphenyl diselenide (78 mg, 0.25 mmol, 0.5 equiv), tetraethylammonium bromide (11 mg, 0.05 mmol, 0.1 equiv) and (Z)-2-methylene-6-phenylhex-4-en-1-ol (94 mg 0.50 mmol, 1.0 equiv). The mixture was electrolyzed until 2.34 F/mol have passed. The product was obtained after column chromatography (eluent: n-pentane/Et_2O 30:1) as colorless oil (79 mg, 0.23 mmol, 50%). **^{1}H NMR** (300 MHz, $CDCl_3$) δ 7.44 – 7.28 (m, 2H), 7.23 – 7.04 (m, 8H), 4.91 – 4.85 (m, 1H), 4.85 – 4.79 (m, 1H), 4.39 (d, J = 13.0 Hz, 1H), 4.17 (dd, J = 13.0, 1.5 Hz, 1H), 4.01 (ddd, J = 9.3, 6.4, 3.2 Hz, 1H), 3.36 (td, J = 7.6, 3.2 Hz, 1H), 3.21 – 3.11 (m, 1H), 2.97 (dt, J = 10.9, 5.2 Hz, 1H), 2.73 – 2.60 (m, 1H), 2.46 (ddd, J = 6.3, 5.2, 3.1 Hz, 1H). **^{13}C NMR** (75 MHz, $CDCl_3$) δ 147.9, 139.9, 134.5, 130.1, 129.4, 129.1, 128.5, 127.4, 126.5, 104.5, 80.5, 71.8, 52.7, 39.9, 36.9. **^{77}Se NMR** (95 MHz, $CDCl_3$) δ 340.8. **IR** (ATR) 3064, 3026, 2912, 2846, 1482, 1432, 1053, 1029, 885, 738, 695. **MS** (EI+) m/z = 344 ($[M]^+$, 21), 262 (12), 187 (10), 169 (24), 157 (11), 145 (9), 129 (13), 117 (20), 105 (10), 91 (100), 83 (33), 77 (15). **HRMS** (EI+) m/z calculated for $C_{19}H_{20}OSe$ ($[M]^+$): 344.0679; found: 344.0683.

***threo*-2-(2-(4-Bromophenyl)-1-(phenylselanyl)ethyl)-4-methylenetetrahydrofuran (3b)**

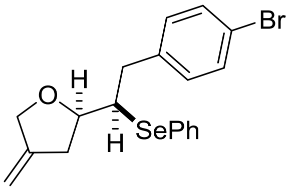

The title compound was prepared according to GP5 using diphenyl diselenide (78 mg, 0.25 mmol, 0.5 equiv), tetraethylammonium bromide (11 mg, 0.05 mmol, 0.1 equiv) and (Z)-6-(4-bromophenyl)-2-methylenehex-4-en-1-ol (134 mg 0.50 mmol, 1.0 equiv). The mixture was electrolyzed until 2.24 F/mol have passed. The product was obtained after column chromatography (eluent: n-pentane/Et_2O 50:1→30:1) as colorless oil (172 mg, 0.41 mmol,

82%). **^{1}H NMR** (300 MHz, $CDCl_3$) δ 7.48 – 7.29 (m, 4H), 7.26 – 7.15 (m, 3H), 7.10 – 6.95 (m, 2H), 5.02 – 4.94 (m, 1H), 4.94 – 4.87 (m, 1H), 4.46 (d, *J* = 12.9 Hz, 1H), 4.24 (dd, *J* = 13.0, 1.5 Hz, 1H), 4.06 (ddd, *J* = 9.3, 6.4, 3.2 Hz, 1H), 3.42 – 3.32 (m, 1H), 3.18 (dd, *J* = 14.0, 7.2 Hz, 1H), 2.95 (dd, *J* = 14.0, 8.1 Hz, 1H), 2.81 – 2.67 (m, 1H), 2.63 – 2.49 (m, 1H). **^{13}C NMR** (75 MHz, $CDCl_3$) δ 147.7, 138.9, 134.6, 131.5, 131.2, 129.8, 129.1, 127.6, 120.3, 104.7, 80.5, 71.9, 52.4, 39.2, 36.8. **^{77}Se NMR** (95 MHz, $CDCl_3$) δ 339.8. **IR** (ATR) 3067, 2911, 2844, 1662, 1579, 1481, 1432, 1405, 1312, 1262, 1066, 1020, 887, 832, 801, 738, 691. **MS** (EI+) *m/z* = 421 ([M]$^+$, 10), 340 (8), 267 (10), 249 (10), 183 (12), 169 (60), 157 (18), 128 (17), 115 (23), 95 (30), 83 (100), 77 (36). **HRMS** (EI+) *m*/*z* calculated for $C_{19}H_{19}BrOSe$ ([M]$^+$): 421.9784; found: 421.9794.

***threo*-Methyl-3-(2-(4-methylenetetrahydrofuran-2-yl)-2-(phenylselanyl)ethyl)benzoate (3d)**

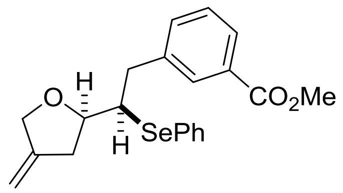

The title compound was prepared according to GP5 using diphenyl diselenide (78 mg, 0.25 mmol, 0.5 equiv), tetraethylammonium bromide (11 mg, 0.05 mmol, 0.1 equiv) and (*Z*)-methyl 3-(5-(hydroxymethyl)hexa-2,5-dienyl)benzoate (123 mg, 0.50 mmol, 1.0 equiv). The mixture was electrolyzed until 5.41 F/mol have passed. The product was obtained after column chromatography (eluent: *n*-pentane/Et_2O 30:1) as yellow oil (117 mg, 0.29 mmol, 58%). **^{1}H NMR** (300 MHz, $CDCl_3$) δ 7.86 (dd, *J* = 7.4, 6.1 Hz, 1H), 7.44 – 7.28 (m, 2H), 7.25 – 7.14 (m, 1H), 5.06 – 4.96 (m, 1H), 4.96 – 4.83 (m, 1H), 4.48 (d, *J* = 13.0 Hz, 1H), 4.27 (d, *J* = 13.0 Hz, 1H), 4.18 – 4.01 (m, 1H), 3.90 (s, 3H), 3.46 (td, *J* = 8.1, 3.4 Hz, 1H), 3.29 (dd, *J* = 14.1, 7.1 Hz, 1H), 3.07 (dd, *J* = 14.1, 8.2 Hz, 1H), 2.77 (dd, *J* = 16.7, 8.3 Hz, 1H), 2.58 (dd, *J* = 15.5, 5.9 Hz, 1H). **^{13}C NMR** (75 MHz, $CDCl_3$) δ 167.3, 147.8, 140.3, 134.6, 134.2, 130.4, 129.8, 129.1, 128.5, 127.8, 127.5, 104.7, 80.7, 71.9, 52.4, 52.2, 39.6, 36.9. **^{77}Se NMR** (95 MHz, $CDCl_3$) δ 338.5. **IR** (ATR) 3060, 2949, 2849, 1720, 1582, 1475, 1437, 1281, 1200, 1105, 1031, 993, 886, 734, 694. **MS** (EI+) *m/z* = 402 ([M]$^+$, 14), 314 (19), 244 (57), 228 (26), 213 (100), 175 (12), 169 (20), 163 (58), 149 (57), 129 (38), 115 (30), 95 (28), 82 (59), 77 (40). **HRMS** (EI+) *m*/*z* calculated for $C_{21}H_{22}O_3Se$ ([M]$^+$): 402.0734; found: 402.0739.

threo-2-(2-(4-Methoxyphenyl)-1-(phenylselanyl)ethyl)-4-methylenetetrahydrofuran (3f)

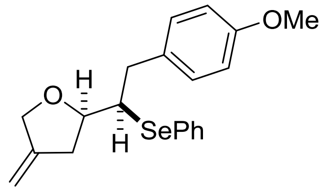

The title compound was prepared according to GP5 using diphenyl diselenide (78 mg 0.25 mmol, 0.5 equiv), tetraethylammonium bromide (11 mg, 0.05 mmol, 0.1 equiv) and (*Z*)-6-(4-methoxyphenyl)-2-methylenehex-4-en-1-ol (109 mg, 0.50 mmol, 1.0 equiv). The mixture was electrolyzed until 2.18 F/mol have passed. The product was obtained after column chromatography (eluent: *n*-pentane/Et_2O 30:1) as slight yellow oil (127 mg, 0.34 mmol, 68%). **^{1}H NMR** (500 MHz, $CDCl_3$) δ 7.47 – 7.42 (m, 2H), 7.25 – 7.19 (m, 3H), 7.12 – 7.06 (m, 2H), 6.84 – 6.78 (m, 2H), 5.03 – 4.86 (m, 2H), 4.47 (d, *J* = 12.9 Hz, 1H), 4.25 (dd, *J* = 13.0, 1.5 Hz, 1H), 4.09 (ddd, *J* = 9.3, 6.4, 3.3 Hz, 1H), 3.78 (s, 3H), 3.41 (td, *J* = 7.6, 3.3 Hz, 1H), 3.08 (ddd, *J* = 91.9, 14.2, 7.7 Hz, 2H), 2.81 – 2.51 (m, 2H). **^{13}C NMR** (126 MHz, $CDCl_3$) δ 158.3, 148.0, 134.5, 132.0, 130.4, 130.2, 129.1, 127.4, 113.9, 104.5, 80.4, 71.9, 55.4, 53.0, 39.0, 37.0. **^{77}Se NMR** (76 MHz, $CDCl_3$) δ 340.1. **IR** (ATR) 3066, 2997, 2941, 2837, 1670, 1609, 1579, 1509, 1437, 1361, 1300, 1243, 1175, 1034, 956, 888, 822, 740, 691, 528, 468, 408. **MS** (EI+): *m/z* = 374 ([M]$^+$,4), 216 (13), 147 (95), 121 (100), 115 (29), 91 (48), 78 (33), 77 (34) 51 (19), 39 (21). **HRMS**: (EI+): *m*/*z* calculated for $C_{20}H_{22}O_2Se$ ([M]$^+$): 374.0785; found: 374.0787.

threo-4-Methylene-2-(1-(phenylselanyl)-2-(2,4,6-trimethoxyphenyl)ethyl)tetrahydro-furan (3g)

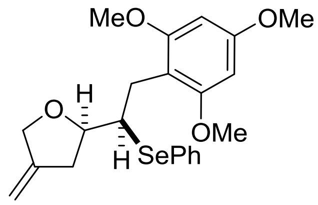

The title compound was prepared according to GP5 using diphenyl diselenide (78 mg, 0.25 mmol, 0.5 equiv), tetraethylammonium bromide (11 mg, 0.05 mmol, 0.1 equiv) and (*Z*)-2-methylene-6-(2,4,6-trimethoxyphenyl)hex-4-en-1-ol (139 mg, 0.50 mmol, 1.0 equiv). The mixture was electrolyzed until 4.85 F/mol have passed. The product was obtained after column chromatography (eluent: *n*-pentane/Et_2O 3:1→1:1) as yellow oil (91 mg, 0.21 mmol, 42%). **^{1}H NMR** (300 MHz, $CDCl_3$) δ 7.51 – 7.39 (m, 2H), 7.21 – 7.08 (m, 3H), 6.00 (s, 2H),

4.89 – 4.82 (m, 1H), 4.83 – 4.76 (m, 1H), 4.38 (d, *J* = 13.0 Hz, 1H), 4.14 (dd, *J* = 13.0, 1.6 Hz, 1H), 3.95 (ddd, *J* = 9.4, 6.3, 3.5 Hz, 1H), 3.72 (s, 3H), 3.65 (s, 6H), 3.55 (ddd, *J* = 8.6, 7.0, 3.5 Hz, 1H), 3.18 (ddd, *J* = 18.4, 17.3, 9.3 Hz, 2H), 3.08 – 2.95 (m, 1H), 2.70 – 2.53 (m, 1H), 2.42 (dd, *J* = 15.7, 6.1 Hz, 1H). **^{13}C NMR** (75 MHz, $CDCl_3$) δ 159.9, 159.4, 148.7, 134.0, 131.3, 128.7, 126.7, 109.3, 104.0, 90.6, 81.1, 71.8, 55.6, 55.4, 50.6, 37.3, 27.5. **^{77}Se NMR** (95 MHz, $CDCl_3$) δ 345.4. **IR** (ATR) 3070, 2928, 2841, 1597, 1495, 1459, 1422, 1261, 1198, 1149, 1117, 1053, 1031, 949, 884, 804, 738, 692, 664. **MS** (EI+): *m/z* = 434 ($[M]^+$, 1), 276 (11), 207 (26), 181 (100), 168 (85), 151 (9), 139 (20), 121 (18), 91 (12), 77 (13). **MS**: (EI+): *m/z* calculated for $C_{22}H_{26}O_4Se$ ($[M]^+$): 434.0996; found: 434.1013.

***threo*-3-(2-(4-Methylenetetrahydrofuran-2-yl)-2-(phenylselanyl)ethyl)furan (3j)**

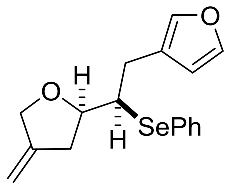

The title compound was prepared according to GP5 using diphenyl diselenide (78 mg, 0.25 mmol, 0.5 equiv), tetraethylammonium bromide (11 mg, 0.05 mmol, 0.1 equiv) and (*Z*)-6-(furan-2-yl)-2-methylenehex-4-en-1-ol (89 mg, 0.50 mmol, 1.0 equiv). The mixture was electrolyzed until 2.51 F/mol have passed. The product was obtained after column chromatography (eluent: *n*-pentane/Et_2O 10:1) as yellow oil (150 mg, 0.45 mmol, 90%). **^{1}H NMR** (500 MHz, $CDCl_3$) δ 7.52 (dd, *J* = 7.2, 1.9 Hz, 2H), 7.37 – 7.31 (m, 1H), 7.31 – 7.22 (m, 4H), 6.27 (s, 1H), 5.03 – 4.97 (m, 1H), 4.93 (s, 1H), 4.47 (d, *J* = 13.0 Hz, 1H), 4.27 (d, *J* = 13.1 Hz, 1H), 4.19 – 4.11 (m, 1H), 3.38 (td, *J* = 7.3, 3.8 Hz, 1H), 3.06 (dd, *J* = 14.9, 7.0 Hz, 1H), 2.88 (dd, *J* = 14.9, 7.5 Hz, 1H), 2.78 – 2.69 (m, 1H), 2.63 (dd, *J* = 15.8, 6.3 Hz, 1H). **^{13}C NMR** (126 MHz, $CDCl_3$) δ 147.8, 142.9, 140.3, 134.5, 129.9, 129.1, 127.5, 122.7, 111.4, 104.7, 80.8, 71.8, 51.2, 36.9, 28.9. **^{77}Se NMR** (95 MHz, $CDCl_3$) δ 344.1. **IR** (ATR) 3070, 2914, 2847, 1725, 1576, 1474, 1433, 1161, 1061, 1025, 845, 785, 735, 690, 597. **MS** (EI+): *m/z* = 334 ($[M]^+$, 15), 314 (10), 234 (7), 177 (42), 157 (26), 131 (19), 119 (12), 109 (21), 91 (41), 81 (100), 77 (49). **HR-MS**: (EI+): *m/z* calculated for $C_{17}H_{18}O_2Se$ ($[M]^+$): 334.0472; found: 334.0473.

***threo*-4-Methylene-2-(1-(phenylselanyl)-2-(thiophen-2-yl)ethyl)tetrahydrofuran (3k)**

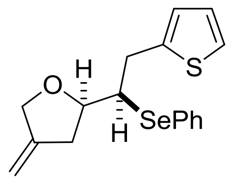

The title compound was prepared according to GP5 using diphenyl diselenide (78 mg, 0.25 mmol, 0.5 equiv), tetraethylammonium bromide (11 mg, 0.05 mmol, 0.1 equiv) and (*Z*)-2-methylene-6-(thiophen-2-yl)hex-4-en-1-ol (97 mg, 0.50 mmol, 1.0 equiv). The mixture was electrolyzed until 4.59 F/mol have passed. The product was obtained after column chromatography (eluent: *n*-pentane/Et_2O 50:1→30:1) as yellow oil (108 mg, 0.31 mmol, 62%). **^{1}H NMR** (300 MHz,) δ 7.58 – 7.41 (m, 2H), 7.23 (dd, *J* = 5.6, 2.5 Hz, 1H), 7.13 (dt, *J* = 4.3, 2.3 Hz, 2H), 7.05 – 6.84 (m, 2H), 6.16 – 5.96 (m, 1H), 5.01 (dt, *J* = 4.4, 2.1 Hz, 1H), 4.97 – 4.86 (m, 1H), 4.59 – 4.37 (m, 2H), 4.37 – 4.18 (m, 1H), 4.13 (ddd, *J* = 9.2, 6.4, 3.2 Hz, 1H), 3.42 (dd, *J* = 11.4, 5.7 Hz, 1H), 3.25 (dd, *J* = 17.4, 9.3 Hz, 1H), 2.83 – 2.64 (m, 1H), 2.53 – 2.34 (m, 1H).**^{13}C NMR** (75 MHz, $CDCl_3$) δ 147.8, 142.4, 141.9, 134.5, 129.2, 127.5, 126.1, 124.6, 124.0, 104.7, 80.0, 71.0, 39.4, 37.0, 34.0. **^{77}Se NMR** (95 MHz, $CDCl_3$) δ 343.7. **IR** (ATR) 3075, 2908, 2843, 1725, 1689, 1431, 1040, 955, 885, 848, 738, 692. **MS** (EI+): *m/z* = 350 ($[M]^+$, 1), 192 (92), 164 (14), 149 (15), 137 (76), 123 (80), 110 (45), 97 (59), 82 (87), 54 (100). **HR-MS**: (EI+): *m/z* calculated for $C_{17}H_{18}OSSe$ ($[M]^+$): 350.0244; found: 350.0241.

***threo*-4-Methylene-2-(1-(phenylselanyl)ethyl)tetrahydrofuran (3l)**

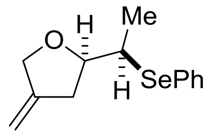

The title compound was prepared according to GP5 using diphenyl diselenide (78 mg, 0.25 mmol, 0.5 equiv), tetraethylammonium bromide (11 mg, 0.05 mmol, 0.1 equiv) and (*Z*)-2-methylenehex-4-en-1-ol (56 mg, 0.50 mmol, 1.0 equiv). The mixture was electrolyzed until 2.19 F/mol have passed. The product was obtained after column chromatography (eluent: *n*-pentane/Et_2O 50:1→25:1) as colorless oil (115 mg, 0.43 mmol, 86 %). **^{1}H NMR** (300 MHz, $CDCl_3$) δ 7.65 – 7.52 (m, 2H), 7.33 – 7.20 (m, 3H), 4.95 (dt, *J* = 21.7, 2.1 Hz, 2H), 4.45 (d, *J* = 13.1 Hz, 1H), 4.27 (dd, *J* = 13.1, 1.0 Hz, 1H), 4.03 (dt, *J* = 8.6, 6.2 Hz, 1H), 3.42 (quin, *J* = 6.9 Hz, 1H), 2.63 (d, *J* = 6.1 Hz, 1H), 2.53 (dd, *J* = 8.6, 1.9 Hz, 1H), 1.41 (d, *J* = 7.0 Hz, 3H). **^{13}C NMR** (75 MHz, $CDCl_3$) δ 147.9, 135.1, 129.1, 127.6 (2C), 104.6, 83.0, 71.8, 43.0,

36.4, 18.3. **^{77}Se NMR** (95 MHz, $CDCl_3$) δ 381.1. **IR** (ATR) 3069, 2957, 2917, 2840, 1665, 1579, 1473, 1436, 1372, 1308, 1242, 1192, 1162, 1106, 1046, 961, 885, 839, 736, 692, 665, 469, 406. **MS** (EI+) *m/z* = 268 ($[M]^+$, 29), 186 (38), 158 (38), 111 (100), 83 (82), 57 (68). **HRMS** (ESI+) *m*/*z* calculated for $C_{13}H_{16}OSeNa$ ($[M+Na]^+$): 292.0259; found: 292.0258.

2-Methyl-4-methylene-2-(2-(phenylselanyl)propan-2-yl)tetrahydrofuran (3m) and 2,2,3-trimethyl-5-methylene-3-(phenylselanyl)tetrahydro-2*H*-pyran (4m)

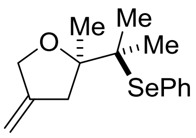

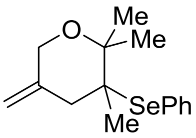

The title compounds were prepared according to GP5 using diphenyl diselenide (78 mg, 0.25 mmol, 0.5 equiv), tetraethylammonium bromide (11 mg, 0.05 mmol, 0.1 equiv) and 4,5-dimethyl-2-methylenehex-4-en-1-ol (70 mg, 0.50 mmol, 1.0 equiv). The mixture was electrolyzed until 2.64 F/mol have passed. After flash chromatography (eluent: *n*-pentane/Et_2O 30:1) the tetrahydrofuran **3m** (68 mg, 0.23 mmol, 46%) was obtained as slight yellow oil and the pyran **4m** (71 mg, 0.24 mmol, 47%) was obtained as colorless oil. Analytical data of the tetrahydrofuran **3m**: **^{1}H NMR** (500 MHz, $CDCl_3$) δ 7.69 – 7.64 (m, 2H), 7.39 – 7.34 (m, 1H), 7.32 – 7.27 (m, 2H), 5.02 – 4.99 (m, 1H), 4.95 – 4.92 (m, 1H), 4.47 (dt, *J* = 7.3, 2.4 Hz, 1H), 4.38 (dq, *J* = 13.1, 1.9 Hz, 1H), 3.16 (d, *J* = 15.6 Hz, 1H), 2.34 (dd, *J* = 15.6, 1.1 Hz, 1H), 1.48 (s, 3H), 1.32 (s, 3H), 1.27 (s, 3H). **^{13}C NMR** (75 MHz, $CDCl_3$) δ 148.6, 139.0, 128.7, 128.6, 128.4, 104.9, 89.0, 71.2, 55.8, 42.2, 26.8, 26.5, 21.8. **^{77}Se NMR** (95 MHz, $CDCl_3$) δ 490.1. **IR** (ATR) 3070, 2963, 2926, 2858, 1666, 1577, 1463, 1433, 1370, 1325, 1280, 1187, 1127, 1100, 1066, 1038, 884, 770, 740, 693. **MS** (EI+) *m/z* = 269 ($[M]^+$, 7), 139 (100), 97 (91), 81 (95), 43 (57). **HRMS** (EI+) *m*/*z* calculated for $C_{15}H_{20}OSe$ ($[M]^+$): 296.0679; found: 296.0681. Analytical data of the pyran **4m**: **^{1}H NMR** (300 MHz, $CDCl_3$) δ = 7.69 – 7.62 (m, 2H), 7.42 – 7.27 (m, 3H), 4.84 (s, 1H), 4.68 (s, 1H), 4.22 (d, *J* = 13.2 Hz, 1H), 4.06 (d, *J* = 13.2 Hz, 1H), 2.54 (dd, *J* = 124.1, 14.3 Hz, 2H), 1.52 (s, 3H), 1.36 (s, 3H), 1.33 (s, 3H). **^{13}C NMR** (75 MHz, $CDCl_3$) δ 142.2, 139.1, 128.9, 128.8, 127.5, 110.3, 77.6, 66.1, 54.4, 43.7, 24.6, 23.9, 23.8. **^{77}Se NMR** (95 MHz, $CDCl_3$) δ 480.7. **IR** (ATR) 3069, 2979, 2933, 2843, 1655, 1577, 1442, 1372, 1297, 1254, 1219, 1149, 1055, 896, 810, 740, 693. **MS** (EI+) *m/z* = 296 ($[M]^+$, 6), 139 (100), 123 (52), 81 (94), 77 (39), 43 (53). **HRMS** (EI+) *m*/*z* calculated for $C_{15}H_{20}OSe$ ($[M]^+$): 296.0679; found: 296.0666.

***threo*/*erythro*-2-Methyl-4-methylene-2-(1-(phenylselanyl)ethyl)tetrahydrofuran (3o and 3o') and 2,2-dimethyl-5-methylene-3-(phenylselanyl)tetrahydro-2*H*-pyran (4n)**

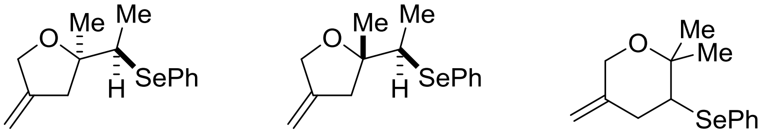

The title compounds were prepared according to GP5 using diphenyl diselenide (98 mg, 0.32 mmol, 0.5 equiv), tetraethylammonium bromide (17 mg, 0.08 mmol, 0.1 equiv) and a mixture of 5-methyl-2-methylenehex-4-en-1-ol and (*Z*)-4-methyl-2-methylenehex-4-en-1-ol (**2n**:**2o** = 79:21) (80 mg, 0.63 mmol, 1.0 equiv). The mixture was electrolyzed until 3.10 F/mol have passed. After chromatography (eluent: *n*-pentane/Et_2O 30:1) the tetrahydrofurans **3o** and **3o'** (17 mg, 0.06, 10%) was obtained as yellow oil and the pyran **4n** (103 mg, 0.37 mmol, 58%) as colorless oil. ^{1}H NMR data of the major isomer of the tetrahydrofuran **3o**: **^{1}H NMR** (300 MHz, $CDCl_3$) δ 7.61 – 7.51 (m, 2H), 7.31 – 7.21 (m, 3H), 4.77 (d, *J* = 0.8 Hz, 1H), 4.70 (s, 1H), 4.19 (d, *J* = 13.1 Hz, 1H), 3.98 (d, *J* = 13.0 Hz, 1H), 3.24 (dd, *J* = 10.9, 5.9 Hz, 1H), 2.69 – 2.54 (m, 2H), 1.42 (s, 3H), 1.37 (s, 3H). ^{1}H NMR data of the minor isomer of the tetrahydrofuran **3o'**: **^{1}H NMR** (300 MHz, $CDCl_3$) δ 7.61 – 7.51 (m, 2H), 7.31 – 7.21 (m, 3H), 5.02 – 4.94 (m, 1H), 4.94 – 4.86 (m, 1H), 4.46 – 4.30 (m, 2H), 3.46 (q, *J* = 7.1 Hz, 1H), 2.77 – 2.69 (m, 1H), 2.46 – 2.35 (m, 1H), 1.39 (s, 3H), 1.32 (s, 3H). Analytical data of the mixture of **3o** and **3o'**: **^{13}C NMR** (75 MHz, $CDCl_3$) δ 148.3, 143.5, 134.9, 134.5, 129.5, 129.1, 128.9, 127.6, 127.3, 109.4, 104.9, 85.9, 75.2, 70.4, 65.9, 52.8, 52.3, 49.5, 43.0, 37.3, 29.0, 22.4, 19.6, 18.7. **^{77}Se NMR** (95 MHz, $CDCl_3$) δ 385.1, 383.7. **IR** (ATR) 3068, 2961, 2919, 2853, 1664, 1577, 1464, 1433, 1384, 1357, 1117, 1053, 1021, 957, 884, 741, 693. **MS** (EI+) *m/z* = 269 ($[M]^+$, 7), 139 (100), 97 (91), 81 (95), 43 (57). **HRMS** (EI+) *m/z* calculated for $C_{14}H_{18}OSe$ ($[M]^+$): 282.0523; found: 282.0531. Analytical data of the pyran **4n**: **^{1}H NMR** (300 MHz, $CDCl_3$) δ 7.70 – 7.61 (m, 2H), 7.41 – 7.25 (m, 4H), 5.01 – 4.95 (m, 1H), 4.95 – 4.87 (m, 1H), 4.46 (d, *J* = 13.0 Hz, 1H), 4.32 – 4.23 (m, 1H), 3.91 (dd, *J* = 8.2, 7.4 Hz, 1H), 2.68 – 2.57 (m, 2H), 1.37 (s, *J* = 4.8 Hz, 3H), 1.33 (s, *J* = 4.7 Hz, 3H). **^{13}C NMR** (75 MHz, $CDCl_3$) δ 148.1, 138.7, 128.8, 127.3, 104.5, 86.6, 77.4, 72.0, 49.4, 35.1, 26.2, 26.1. **^{77}Se NMR** (76 MHz, $CDCl_3$) δ 484.1. **IR** (ATR) 3068, 2975, 2842, 1654, 1577, 1471, 1437, 1372, 1119, 1059, 1021, 903, 873, 823, 739, 691. **MS** (EI+) *m/z* = 269 ($[M]^+$, 7), 139 (100), 97 (91), 81 (95), 43 (57). **HRMS** (EI+) *m/z* calculated for $C_{14}H_{18}OSe$ ($[M]^+$): 282.0523; found: 282.0531.

5-Heptyl-2-methyl-4-methylene-2-(2-(phenylselanyl)propan-2-yl)tetrahydrofuran (3p) and 6-heptyl-2,2,3-trimethyl-5-methylene-3-(phenylselanyl)tetrahydro-2*H*-pyran (4p)

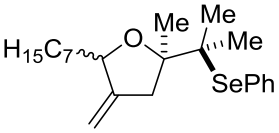

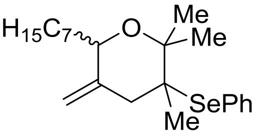

The title compounds were prepared according to GP5 using diphenyl diselenide (100 mg, 0.32 mmol, 0.5 equiv), tetraethylammonium bromide (15 mg, 0.07 mmol, 0.1 equiv) and 2,3-dimethyl-5-methylenetridec-2-en-6-ol (153 mg, 0.64 mmol, 1.0 equiv). The mixture was electrolyzed until 3.46 F/mol have passed. After flash chromatography (eluent: *n*-pentane/Et_2O 100:1→30:1) the tetrahydrofuran **3m** and the pyran **4m** were obtained as slight yellow oil (189 mg, 0.48 mmol, 75%, **3p**:**4p** = 1:2.5). The pyran could be separated from the mixture using flash chromatography (eluent: *n*-pentane/Et_2O 150:1→50:1→30:1) and was obtained as colorless oil (134 mg ,0.34 mmol, 53%). Analytical data of the pyran (**4p**): ^{1}**H NMR** (500 MHz, $CDCl_3$) δ 7.70 – 7.65 (m, 2H), 7.38 – 7.33 (m, 1H), 7.31 – 7.27 (m, 2H), 4.98 (dd, *J* = 3.8, 2.3 Hz, 1H), 4.85 (dt, *J* = 3.0, 1.5 Hz, 1H), 4.38 (ddd, *J* = 6.9, 5.2, 2.0 Hz, 1H), 3.13 (dd, *J* = 15.3, 1.2 Hz, 1H), 2.30 (d, *J* = 15.3 Hz, 1H), 1.65 – 1.57 (m, 2H), 1.49 (s, 3H), 1.36 – 1.24 (m, 12H), 1.21 (s, 3H), 0.87 (t, *J* = 7.0 Hz, 3H). ^{13}**C NMR** (75 MHz, $CDCl_3$) δ 152.5 , 139.0, 128.7, 128.6 , 128.5, 105.0, 87.2, 80.3, 55.1, 42.2, 36.1, 32.0, 29.9, 29.4, 27.0, 26.6, 25.6, 22.8, 21.3, 14.3. ^{77}**Se NMR** (95 MHz, $CDCl_3$) δ 492.9. **IR** (ATR) 3070, 2924, 2855, 1664, 1578, 1461, 1371, 1285, 1066, 1022, 986, 932, 883, 781, 739, 693. **MS** (EI+) *m/z* = 237 (24%), 195 (29%), 85 (17%), 57 (100%), 43 (16%). **HRMS** (EI+) *m*/*z* calculated for $C_{22}H_{34}OSe$ ($[M]^+$): 394,1775; gef.: 394.1784.

Synthesis of 5-(4-bromophenyl)-2-methyl-4-methylene-2-(2-(phenylselanyl)propan-2-yl)tetrahydrofuran (3q) and 6-(4-bromophenyl)-2,2,3-trimethyl-5-methylene-3-(phenylselanyl)tetrahydro-2*H*-pyran (4q)

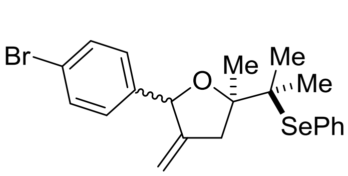

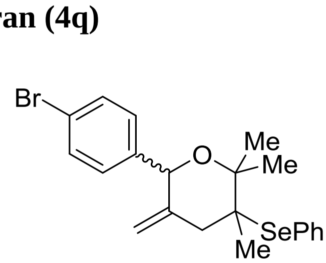

The title compounds were prepared according to GP5 using diphenyl diselenide (78 mg, 0.25 mmol, 0.5 equiv), tetraethylammonium bromide (11 mg, 0.05 mmol, 0.1 equiv) and 1-(4-bromophenyl)-4,5-dimethyl-2-methylenehex-4-en-1-ol (151 mg, 0.51 mmol, 1.0 equiv). The

mixture was electrolyzed until 3.46 F/mol have passed. After flash chromatography (eluent: *n*-pentane/Et_2O 100:1→30:1) the tetrahydrofuran **3q** and the pyran **4q** were obtained as slight yellow oil (121 mg, 0.27 mmol, 53%, **3q**:**4q** = 1:1.4). The pyran could be separated from the mixture using flash chromatography (eluent: *n*-pentane/Et_2O 200:1→100:1→50:1) and was obtained as colorless oil (72 mg ,0.16 mmol, 31%). Analytical data of the pyran (**4q**): **^{1}H NMR** (300 MHz, $CDCl_3$) δ 7.62 – 7.54 (m, 2H), 7.43 – 7.34 (m, 2H), 7.33 – 7.15 (m, 5H), 5.20 (d, *J* = 1.4 Hz, 1H), 4.97 (dd, *J* = 4.3, 2.5 Hz, 1H), 4.57 (dt, *J* = 2.8, 2.0 Hz, 1H), 3.23 (dd, *J* = 15.9, 2.0 Hz, 1H), 2.43 (dd, *J* = 15.9, 0.8 Hz, 1H), 1.46 (s, *J* = 3.6 Hz, 3H), 1.35 (s, *J* = 13.3 Hz, 3H), 1.20 (s, *J* = 9.8 Hz, 3H). **^{13}C NMR** (126 MHz, $CDCl_3$) δ 152.0, 140.6, 139.0, 131.6, 129.5, 128.7, 128.2, 121.9, 108.0, 87.8, 81.8, 55.1, 42.4, 26.9, 26.7, 21.0. **^{77}Se NMR** (95 MHz, $CDCl_3$) δ 493.2. **IR** (ATR) 3065, 2965, 2925, 2859, 1586, 1477, 1372, 1276, 1177, 1103, 1057, 1011, 964, 894, 817, 739, 692, 616. **MS** (EI+) *m/z* = .**HRMS** (ESI+): *m*/*z* ber. für $C_{21}H_{23}BrNaOSe$ ($[M+Na]^+$): 472.9990; gef.: 472.9990.

A aromatic quaternary carbon of **4q** cannot be identified in the ^{13}C NMR spectra.

General procedure for the electrochemical iodonium induced alkoxylation of 1,4-dienols (GP6)

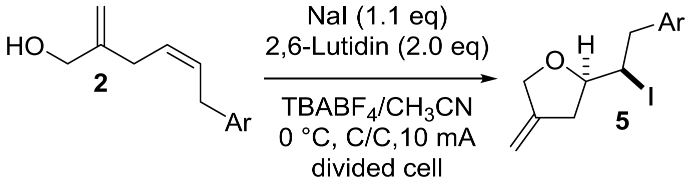

An H-type divided cell (4G glass filter) was equipped with a carbon fiber anode and carbon fiber cathode. Each chamber was charged with 10 mL $TBABF_4$ solution (0.3 M in CH_3CN) and 2,6-lutidine (2.0 equiv). The anodic chamber was charged with the 1,4-diene (1.0 equiv) and sodium iodide (1.1 equiv). Constant current electrolysis (10 mA) was carried out at 0 °C until completion was detected by TLC and GC−MS analysis. Then, 15 mL $Na_2S_2O_3$ solution (10wt% in H_2O) were added to both chambers, the mixture was extracted with 3x 15 mL Et_2O, dried over $MgSO_4$ and the solvent was removed under reduced pressure. The product was obtained after column chromatography (*n*-pentane/Et_2O).

***threo*-2-(1-Iodo-2-phenylethyl)-4-methylenetetrahydrofuran (5a)**

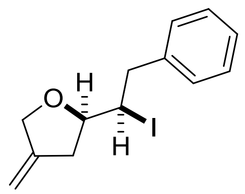

The title compound was prepared according to GP6 using (*Z*)-2-methylene-6-phenylhex-4-en-1-ol (94 mg, 0.50 mmol, 1.0 equiv), sodium iodide (82 mg, 0.55 mmol, 1.1 equiv) and 2,6-lutidine (0.12 mL, 1.00 mmol, 2.0 equiv). The mixture was electrolyzed until 2.56 F/mol have passed. The product was obtained after column chromatography (eluent: *n*-pentane/Et_2O 50:1) as colorless oil (104 mg, 0.33 mmol, 66%). **^{1}H NMR** (300 MHz, $CDCl_3$) δ 7.41 – 7.26 (m, 3H), 7.26 – 7.15 (m, 2H), 4.96 (ddt, *J* = 18.1, 4.5, 2.1 Hz, 2H), 4.55 (d, *J* = 12.8 Hz, 1H), 4.32 (ddd, *J* = 8.8, 6.5, 3.9 Hz, 2H), 3.80 – 3.54 (m, 1H), 3.37 (dd, *J* = 14.2, 6.4 Hz, 1H), 3.24 (dd, *J* = 14.3, 8.9 Hz, 1H), 2.76 – 2.61 (m, 1H), 2.61 – 2.42 (m, 1H). **^{13}C NMR** (75 MHz, $CDCl_3$) δ 147.0, 139.7, 129.2, 128.6, 127.0, 105.1, 80.9, 72.1, 43.3, 41.1, 38.7. **IR** (ATR) 3078, 3027, 2990, 2907, 2835, 1668, 1494, 1429, 1338, 1052, 1032, 886, 745, 697. **MS** (EI+) *m/z* = 314 ($[M]^+$, 1), 187 (45), 169 (21), 159 (6),145 (12), 129 (11), 109 (14), 91 (100), 83 (40), 55 (27). **HRMS** (EI+) *m*/*z* calculated for $C_{13}H_{15}IO$ ($[M]^+$): 314.0168; found: 314.0172.

***threo*-2-(2-(4-Bromophenyl)-1-iodoethyl)-4-methylenetetrahydrofuran (5b)**

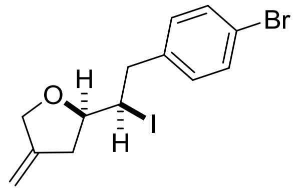

The title compound was prepared according to GP6 (*Z*)-6-(4-bromophenyl)-2-methylenehex-4-en-1-ol (134 mg, 0.50 mmol, 1.0 equiv), sodium iodide (82 mg, 0.55 mg, 1.1 equiv) and 2,6-lutidine (0.12 mL, 1.00 mmol, 2.0 equiv). The mixture was electrolyzed until 3.01 F/mol have passed. The product was obtained after column chromatography (eluent: *n*-pentane/Et_2O 50:1) as colorless oil (118 mg, 0.30 mmol, 60%). **^{1}H NMR** (300 MHz, $CDCl_3$) δ 7.37 (d, *J* = 8.3 Hz, 2H), 7.03 (d, *J* = 8.2 Hz, 2H), 4.93 (s, 1H), 4.87 (s, 1H), 4.47 (d, *J* = 13.0 Hz, 1H), 4.26 (d, *J* = 12.9 Hz, 1H), 4.23 – 4.13 (m, 1H), 3.66 – 3.52 (m, 1H), 3.24 (dd, *J* = 14.4, 6.1 Hz, 1H), 3.10 (dd, *J* = 14.3, 9.2 Hz, 1H), 2.62 (dd, *J* = 15.9, 6.5 Hz, 1H), 2.45 (dd, *J* = 15.8, 8.5 Hz, 1H). **^{13}C NMR** (75 MHz, $CDCl_3$) δ 146.8, 138.7, 131.8, 130.9, 120.9, 105.2, 80.9, 72.2, 42.5, 40.4, 38.6. **IR** (ATR) 3078, 2914, 2845, 1486, 1430, 1310, 1244, 1062, 1009,

887, 832, 798, 723. **MS** (EI+) *m/z* = 394 ($[M]^+$, 1), 265 (31), 247 (10), 223 (5), 169 (100), 144 (8), 129 (10), 109 (12), 90 (13), 83 (77), 77 (11), 55 (33). **HRMS** (EI+) *m/z* calculated for $C_{13}H_{14}BrIO$ ($[M]^+$): 391.9273; found: 391.9260.

***threo*-2-(2-(2-Chlorophenyl)-1-iodoethyl)-4-methylenetetrahydrofuran (5c)**

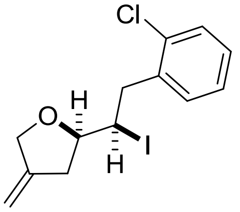

The title compound was prepared according to GP6 using (*Z*)-6-(2-chlorophenyl)-2-methylenehex-4-en-1-ol (111 mg, 0.50 mmol, 1.0 equiv), sodium iodide (82 mg, 0.55 mmol, 1.1 equiv) and 2,6-lutidine (0.12 mL, 1.00 mmol, 2.0 equiv). The mixture was electrolyzed until 2.81 F/mol have passed. The product was obtained after column chromatography (eluent: *n*-pentane/Et_2O 30:1) as colorless oil (69 mg, 0.20 mmol, 40%). **^{1}H NMR** (300 MHz,) δ 7.37 – 7.20 (m, 2H), 7.18 – 7.03 (m, 2H), 4.93 (dt, *J* = 4.4, 2.3 Hz, 1H), 4.90 – 4.82 (m, 1H), 4.57 – 4.36 (m, 2H), 4.35 – 4.17 (m, 1H), 3.53 (ddd, *J* = 8.3, 6.7, 4.0 Hz, 1H), 3.40 (dd, *J* = 14.2, 6.2 Hz, 1H), 3.30 (dd, *J* = 14.2, 8.9 Hz, 1H), 2.72 – 2.55 (m, 1H), 2.55 – 2.41 (m, 1H). **^{13}C NMR** (75 MHz, $CDCl_3$) δ 146.9, 137.0, 134.1, 132.0, 129.7, 128.5, 126.7, 105.0, 81.0, 71.9, 41.4, 38.8, 38.7. **IR** (ATR) 3070, 2944, 2841, 1473, 1436, 1041, 888, 750, 681. **MS** (EI+) *m/z* = 348 ($[M]^+$, 1), 221 (20), 125 (40), 97 (100), 83 (17), 69 (24), 55 (16). **HRMS** (EI+) *m/z* calculated for $C_{13}H_{14}ClIO$ ($[M]^+$): 347.9978; found: 347.9765.

***threo*-Methyl 3-(2-iodo-2-(4-methylenetetrahydrofuran-2-yl)ethyl)benzoate (5d)**

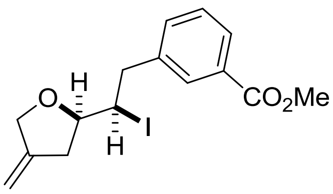

The title compound was prepared according to GP6 using (*Z*)-methyl 3-(5-(hydroxymethyl)hexa-2,5-dienyl)benzoate (123 mg, 0.50 mmol, 1.0 equiv), sodium iodide (82 mg, 0.55 mmol, 1.1 equiv) and 2,6-lutidine (0.12 mL, 1.00 mmol, 2.0 equiv). The mixture was electrolyzed until 4.33 F/mol have passed. The product was obtained after column chromatography (eluent: *n*-pentane/Et_2O 25:1) as orange oil (125 mg, 0.34 mmol, 68%). **^{1}H NMR** (300 MHz, $CDCl_3$) δ 7.94 (dt, *J* = 7.0, 1.8 Hz, 1H), 7.90 (s, 1H), 7.47 – 7.35 (m,

2H), 5.04 – 4.98 (m, 1H), 4.94 (dt, *J* = 4.4, 2.1 Hz, 1H), 4.55 (d, *J* = 12.9 Hz, 1H), 4.40 – 4.27 (m, 2H), 3.92 (s, 3H), 3.70 (ddd, *J* = 8.2, 6.7, 3.8 Hz, 1H), 3.40 (dd, *J* = 14.4, 5.9 Hz, 1H), 3.26 (dd, *J* = 14.4, 9.4 Hz, 1H), 2.76 – 2.65 (m, 1H), 2.60 – 2.47 (m, 1H). **^{13}C NMR** (75 MHz, $CDCl_3$) δ 167.1, 146.9, 140.1, 133.9, 130.6, 130.2, 128.7, 128.3, 105.2, 81.1, 72.2, 52.3, 42.8, 40.5, 38.6. **IR** (ATR) 2950, 2901, 2849, 1719, 1436, 1281, 1199, 1103, 1037, 991, 885, 803, 749, 701. **MS** (EI+) *m/z* = 372 ($[M]^+$, 1), 245 (16), 227 (5), 213 (100), 163 (11), 149 (41), 131 (11), 115 (7), 95 (11), 83 (39), 55 (13). **HRMS** (EI+) *m*/*z* calculated for $C_{15}H_{17}IO_3$ ($[M]^+$): 372.0222; found: 372.0217.

***threo*-2-(1-Iodo-2-(4-(trifluoromethyl)phenyl)ethyl)-4-methylenetetrahydrofuran (5e)**

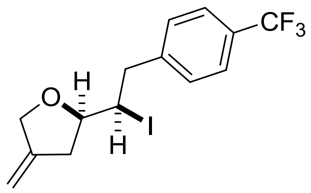

The title compound was prepared according to GP6 using (*Z*)-2-methylene-6-(4-(trifluoromethyl)phenyl)hex-4-en-1-ol (128 mg, 0.50 mmol, 1.0 equiv), sodium iodide (82 mg, 0.55 mmol, 1.1 equiv) and 2,6-lutidine (0.12 mL, 1.00 mmol, 2.0 equiv). The mixture was electrolyzed until 5.00 F/mol have passed. The product was obtained after column chromatography (eluent: *n*-pentane/Et_2O 50:1) as orange oil (86 mg, 0.23 mmol, 46%). **^{1}H NMR** (300 MHz, $CDCl_3$) δ 7.57 (d, *J* = 8.0 Hz, 2H), 7.34 (d, *J* = 7.9 Hz, 2H), 5.06 – 4.98 (m, 1H), 4.95 (d, *J* = 2.0 Hz, 1H), 4.56 (d, *J* = 12.9 Hz, 1H), 4.42 – 4.22 (m, 2H), 3.68 (ddd, *J* = 8.2, 6.7, 3.6 Hz, 1H), 3.42 (dd, *J* = 14.3, 5.8 Hz, 1H), 3.27 (dd, *J* = 14.3, 9.4 Hz, 1H), 2.78 – 2.63 (m, 1H), 2.54 (ddd, *J* = 10.6, 7.6, 2.3 Hz, 1H). **^{13}C NMR** (75 MHz, $CDCl_3$) δ 146.7, 143.6, 129.5 (2C), 129.4 (q, *J* = 32.0 Hz), 124.3 (q, *J* = 272.6 Hz), 125.6 (q, *J* = 3.8 Hz), 105.3, 81.0, 72.2, 42.7, 39.6, 38.5. **IR** (ATR) 3079, 2948, 2848, 1617, 1424, 1321, 1163, 1118, 1061, 1022, 890, 846, 820, 596. **MS** (EI+) *m/z* = 382 ($[M]^+$, 7), 255 (100), 237 (17), 211 (11), 197 (6), 185 (11), 173 (12), 159 (56), 109 (9), 95 (11), 83 (61), 55 (21). **HRMS** (EI+) *m*/*z* calculated for $C_{14}H_{14}F_3IO$ ($[M]^+$): 382.0041; found: 382.0035.

***threo*-2-(1-Iodo-2-(4-methoxyphenyl)ethyl)-4-methylenetetrahydrofuran (5f)**

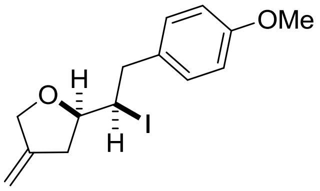

The title compound was prepared according to GP6 using (*Z*)-6-(4-methoxyphenyl)-2-methylenehex-4-en-1-ol (109 mg, 0.50 mmol, 1.0 equiv), sodium iodide (82 mg, 0.55 mmol, 1.1 equiv) and 2,6-lutidine (0.12 mL, 1.00 mmol, 2.0 equiv). The mixture was electrolyzed until 2.98 F/mol have passed. The product was obtained after column chromatography (eluent: *n*-pentane/Et_2O 10:1) as colorless oil (117 mg, 0.34 mmol, 68%). **^{1}H NMR** (300 MHz, $CDCl_3$) δ 7.17 – 7.11 (m, 2H), 6.89 – 6.82 (m, 2H), 5.02 – 4.96 (m, 1H), 4.96 – 4.90 (m, 1H), 4.55 (d, *J* = 12.9 Hz, 1H), 4.39 – 4.24 (m, 2H), 3.80 (s, 3H), 3.65 (ddd, *J* = 8.3, 6.6, 3.8 Hz, 1H), 3.24 (ddd, *J* = 23.1, 14.4, 7.7 Hz, 2H), 2.72 – 2.46 (m, 2H). **^{13}C NMR** (75 MHz, $CDCl_3$) δ 158.6, 147.1, 131.8, 130.2, 114.0, 105.0, 80.8, 72.1, 55.4, 42.5, 42.0, 38.8. **IR** (ATR) 2995, 2951, 2835, 1664, 1610, 1510, 1244, 1176, 1033, 891, 819. **MS** (EI+) *m/z* = 344 ($[M]^+$, 1), 217 (18), 121 (100), 109 (12), 91 (10), 83 (13), 77 (11), 55 (14). **HRMS** (EI+) *m*/*z* calculated for $C_{14}H_{17}IO_2$ ($[M]^+$): 344,0273; found: 344.0269.

***threo*-2-(1-Iodo-2-(2,4,6-trimethoxyphenyl)ethyl)-4-methylenetetrahydrofuran (5g)**

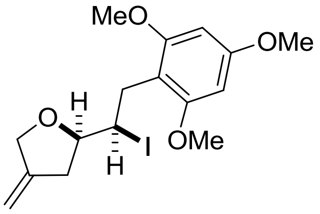

The title compound was prepared according to GP6 using (*Z*)-6-(4-methoxyphenyl)-2-methylenehex-4-en-1-ol (139 mg, 0.50 mmol, 1.0 equiv), sodium iodide (82 mg, 0.55 mmol, 1.1 equiv) and 2,6-lutidine (0.12 mL, 1.00 mmol, 2.0 equiv). The mixture was electrolyzed until 2.80 F/mol have passed. The product was obtained after column chromatography (eluent: *n*-pentane/Et_2O 3:1) as white solid (141 mg, 0.35 mmol, 70%). **Smp**. 95-97 °C . **^{1}H NMR** (300 MHz, $CDCl_3$) δ 6.11 (s, 2H), 4.99 – 4.91 (m, 1H), 4.91 – 4.85 (m, 1H), 4.58 – 4.50 (m, 1H), 4.48 (dd, *J* = 4.9, 3.5 Hz, 1H), 4.29 (dd, *J* = 13.1, 1.5 Hz, 1H), 3.81 (s, 3H), 3.80 (s, 6H), 3.68 (ddd, *J* = 8.6, 6.2, 5.3 Hz, 1H), 3.35 (dd, *J* = 13.9, 8.5 Hz, 1H), 3.15 (dd, *J* = 13.9, 6.6 Hz, 1H), 2.73 – 2.53 (m, 1H), 2.53 – 2.33 (m, 1H). **^{13}C NMR** (75 MHz, $CDCl_3$) δ 160.2, 159.2, 147.9, 109.3, 104.6, 90.6, 82.1, 71.6, 55.8, 55.4, 40.1, 38.6, 30.8. **IR** (ATR)

2939, 2878, 2838, 1594, 1458, 1420, 1201, 1142, 1058, 1031, 889, 815. **MS** (EI+) *m/z* = 404 ($[M]^+$, 1), 312 (3), 277 (4), 181 (100), 168 (3), 151 (6), 136 (8), 121 (14), 109 (22). **HRMS** (EI+) *m/z* calculated for $C_{16}H_{21}IO_4$ ($[M]^+$): 404.0485; found: 404.0476.

***threo*-2-(2-(3,4-Dimethoxyphenyl)-1-iodoethyl)-4-methylenetetrahydrofuran (5h)**

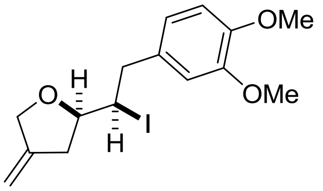

The title compound was prepared according to GP6 using (*Z*)-6-(3,4-dimethoxyphenyl)-2-methylenehex-4-en-1-ol (124 mg, 0.50 mmol, 1.0 equiv), sodium iodide (82 mg, 0.55 mmol, 1.1 equiv) and 2,6-lutidine (0.12 mL, 1.00 mmol, 2.0 equiv). The mixture was electrolyzed until 2.66 F/mol have passed. The product was obtained after column chromatography (eluent: *n*-pentane/Et_2O 3:1) as white solid (125 mg, 0.34 mmol, 67%). **Smp**. 83-85 °C . **^{1}H NMR** (300 MHz, $CDCl_3$) δ 6.85 – 6.75 (m, 2H), 6.74 (s, 1H), 5.01 – 4.96 (m, 1H), 4.95 – 4.90 (m, 1H), 4.54 (d, *J* = 12.9 Hz, 1H), 4.37 – 4.25 (m, 2H), 3.87 (s, *J* = 4.1 Hz, 3H), 3.86 (s, 3H), 3.64 (ddd, *J* = 8.3, 6.6, 3.7 Hz, 1H), 3.30 (dd, *J* = 14.3, 6.6 Hz, 1H), 3.18 (dd, *J* = 14.3, 8.7 Hz, 1H), 2.73 – 2.59 (m, 1H), 2.59 – 2.44 (m, 1H). **^{13}C NMR** (75 MHz, $CDCl_3$) δ 149.1, 148.1, 147.0, 132.3, 121.3, 112.5, 111.4, 105.0, 80.7, 72.1, 56.0, 56.0, 43.0, 41.6, 38.7. **IR** (ATR) 3076, 2995, 2931, 2837, 1593, 1514, 1459, 1336, 1263, 1239, 1148, 1029, 893, 808. **MS** (EI+) *m/z* = 374 ($[M]^+$, 9), 246 (21), 229 (10), 151 (100), 109 (18), 109 (48). **HRMS** (EI+) *m/z* calculated for $C_{15}H_{19}IO_3$ ($[M]^+$): 374.0379; found: 374.0394.

***threo*-2-(2-(2-Bromo-4,5-dimethoxyphenyl)-1-iodoethyl)-4-methylenetetrahydrofuran (5i)**

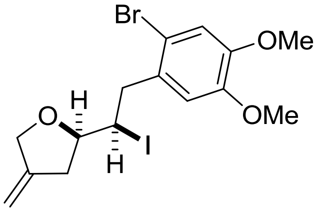

The title compound was prepared according to GP6 using (*Z*)-6-(2-bromo-4,5-dimethoxyphenyl)-2-methylenehex-4-en-1-ol (164 mg, 0.50 mmol, 1.0 equiv), sodium iodide (82 mg, 0.55 mmol, 1.1 equiv) and 2,6-lutidine (0.12 mL, 1.00 mmol, 2.0 equiv). The mixture was electrolyzed until 2.11 F/mol have passed. The product was obtained after column

chromatography (eluent: *n*-pentane/Et_2O 3:1) as white solid (105 mg, 0.23 mmol, 46%). **Smp**. 65-67 °C. **^{1}H NMR** (300 MHz, $CDCl_3$) δ 6.99 (s, 1H), 6.84 (s, 1H), 5.07 – 4.96 (m, 1H), 4.96 – 4.86 (m, 1H), 4.55 (d, *J* = 12.9 Hz, 1H), 4.48 (ddd, *J* = 8.7, 6.7, 3.7 Hz, 1H), 4.32 (dd, *J* = 13.0, 1.4 Hz, 1H), 3.86 (s, 3H), 3.85 (s, 3H), 3.55 (ddd, *J* = 8.3, 6.6, 3.7 Hz, 1H), 3.38 (dd, *J* = 14.3, 6.7 Hz, 1H), 3.30 (dd, *J* = 14.3, 8.7 Hz, 1H), 2.75 – 2.61 (m, 1H), 2.61 – 2.45 (m, 1H). **^{13}C NMR** (75 MHz, $CDCl_3$) δ 148.8, 148.3, 147.0, 130.9, 115.7, 114.9, 114.5, 105.1, 80.8, 72.0, 56.3, 56.3, 43.6, 39.6, 39.1. **IR** (ATR)2930, 2839, 1662, 1596, 1500, 1457, 1436, 1380, 1335, 1256, 1213, 1156, 1062, 1021, 952, 881, 852, 831 794. **MS** (EI+) *m/z* = 451 ($[M]^+$, 5), 277 (10), 245 (20), 229 (100), 151 (15), 127 (8), 109 (17), 83 (31). **HRMS** (EI+) *m*/*z* calculated for $C_{15}H_{18}BrIO_3$ ($[M]^+$): 451.9484; found: 451.9498.

***threo*-3-(2-Iodo-2-(4-methylenetetrahydrofuran-2-yl)ethyl)furan (5j)**

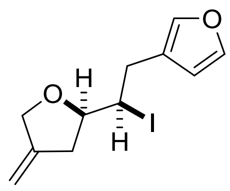

The title compound was prepared according to GP6 using (*Z*)-6-(furan-3-yl)-2-methylenehex-4-en-1-ol (89 mg, 0.50 mmol, 1.0 equiv), sodium iodide (82 mg, 0.55 mmol, 1.1 equiv) and 2,6-lutidine (0.12 mL, 1.00 mmol, 2.0 equiv). The mixture was electrolyzed until 4.12 F/mol have passed. The product was obtained after column chromatography (eluent: *n*-pentane/Et_2O 5:1) as yellow oil (125 mg, 0.41 mmol, 82%). **^{1}H NMR** (300 MHz, $CDCl_3$) δ 7.37 (t, *J* = 1.6 Hz, 1H), 7.34 (d, *J* = 0.7 Hz, 1H), 6.34 (s, *J* = 0.8 Hz, 1H), 5.06 – 4.97 (m, 1H), 4.97 – 4.87 (m, 1H), 4.53 (d, *J* = 13.0 Hz, 1H), 4.33 (dd, *J* = 13.0, 1.4 Hz, 1H), 4.23 (ddd, *J* = 8.4, 6.3, 4.1 Hz, 1H), 3.73 (ddd, *J* = 8.3, 6.6, 4.1 Hz, 1H), 3.24 – 3.02 (m, 2H), 2.79 – 2.61 (m, 1H), 2.61 – 2.42 (m, 1H). **^{13}C NMR** (75 MHz, $CDCl_3$) δ 147.0, 143.1, 140.4, 122.8, 111.0, 105.1, 81.2, 72.1, 39.8, 38.5, 32.7. **IR** (ATR) 3135, 3079, 2912, 2844, 1668, 1500, 1429, 1205, 1059, 1024, 877, 785, 733, 627, 597. **MS** (EI+) *m/z* = 304 ($[M]^+$, 1), 177 (70), 159 (11), 149 (5), 135 (14), 120 (8), 109 (89), 95 (37), 81 (100), 55 (43). **HRMS** (EI+) *m*/*z* calculated for $C_{11}H_{13}IO_2$ ($[M]^+$): 303.9960; found: 303.9953.

threo-2-(1-Iodo-2-(thiophen-2-yl)ethyl)-4-methylenetetrahydrofuran (5k)

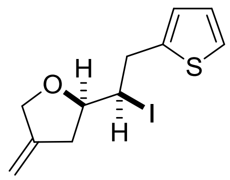

The title compound was prepared according to GP6 using (*Z*)-2-methylene-6-(thiophen-3-yl)hex-4-en-1-ol (97 mg, 0.50 mmol, 1.0 equiv), sodium iodide (82 mg, 0.55 mmol, 1.1 equiv) and 2,6-lutidine (0.12 mL, 1.00 mmol, 2.0 equiv). The mixture was electrolyzed until 5.04 F/mol have passed. The product was obtained after column chromatography (eluent: *n*-pentane/Et_2O 5:1) as slight yellow oil (106 mg, 0.33 mmol, 66%). **^{1}H NMR** (300 MHz, $CDCl_3$) δ 7.19 (dd, *J* = 5.1, 1.2 Hz, 1H), 6.98 – 6.93 (m, 1H), 6.91 (dd, *J* = 3.4, 0.9 Hz, 1H), 5.03 – 4.97 (m, 1H), 4.97 – 4.91 (m, 1H), 4.54 (d, *J* = 13.0 Hz, 1H), 4.39 – 4.28 (m, 2H), 3.68 (ddd, *J* = 8.3, 6.6, 3.8 Hz, 1H), 3.61 – 3.44 (m, 2H), 2.78 – 2.60 (m, 1H), 2.60 – 2.41 (m, 1H). **^{13}C NMR** (75 MHz, $CDCl_3$) δ 146.9, 142.0, 127.0, 126.5, 124.4, 105.2, 80.6, 72.1, 40.5, 38.7, 37.7. **IR** (ATR) 3076, 2918, 2848, 1667, 1430, 1249, 1038, 896, 847, 826, 729, 694. **MS** (EI+) *m/z* = 320 ($[M]^+$, 1), 193 (38), 175 (20), 123 (6), 109 (48), 97 (100), 83 (27), 79 (15), 55 (15). **HRMS** (EI+) *m/z* calculated for $C_{11}H_{13}IOS$ ($[M]^+$): 319.9732; found: 319.9725.

^{1}H and ^{13}C NMR spectra

Buta-1,3-dienylbenzene (1a)

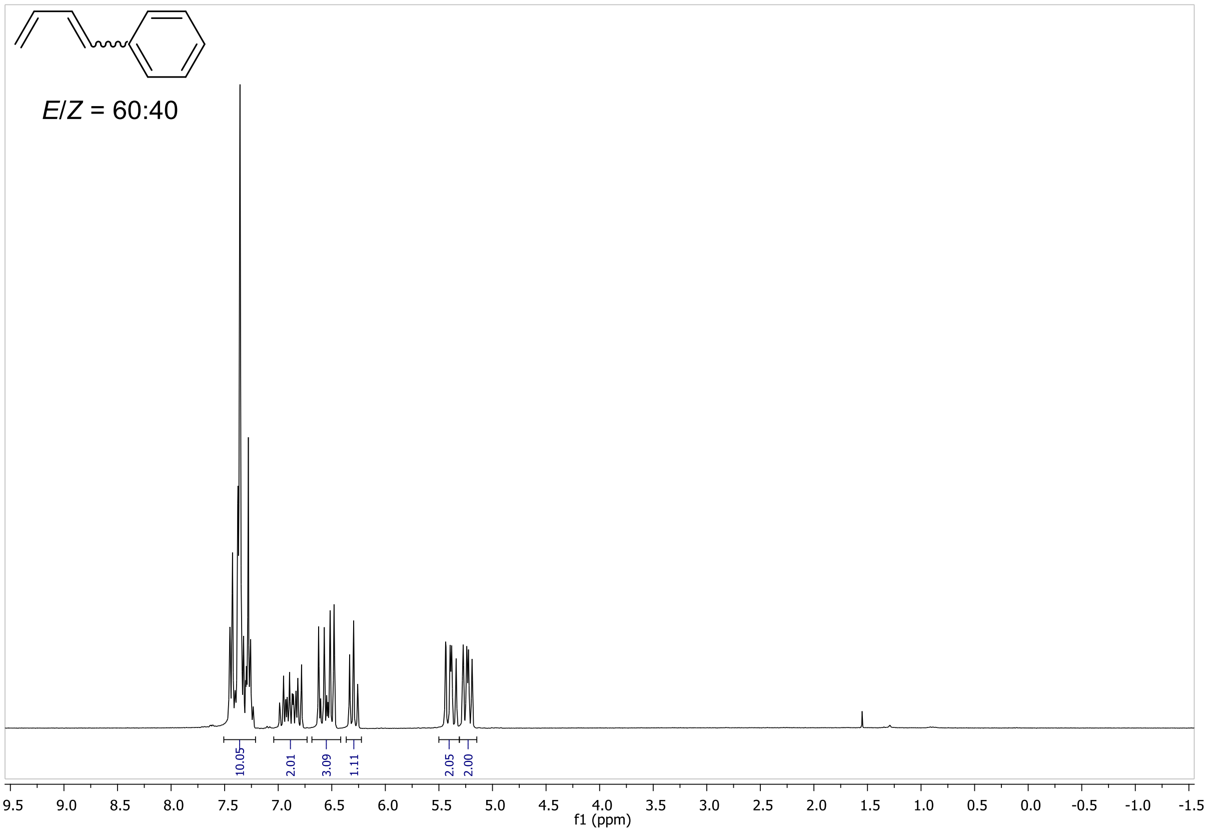

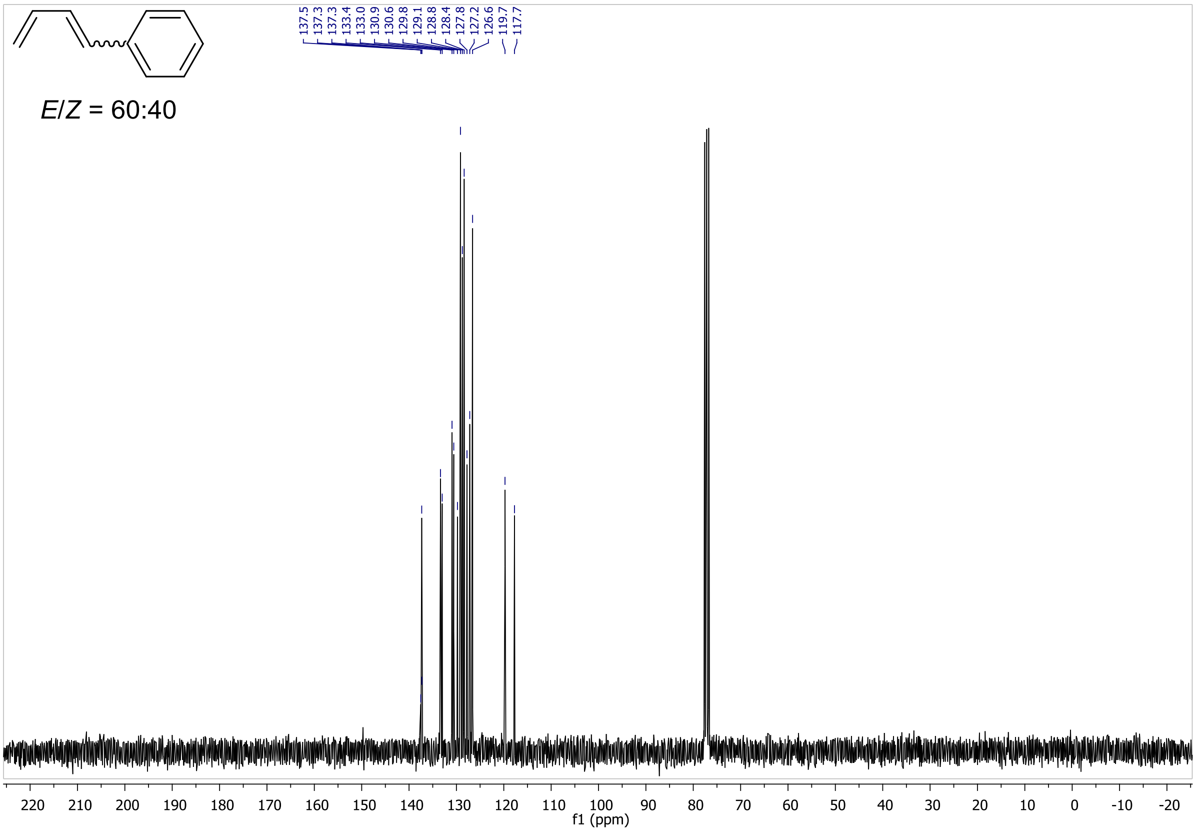

1-Bromo-4-(buta-1,3-dienyl)benzene (1b)

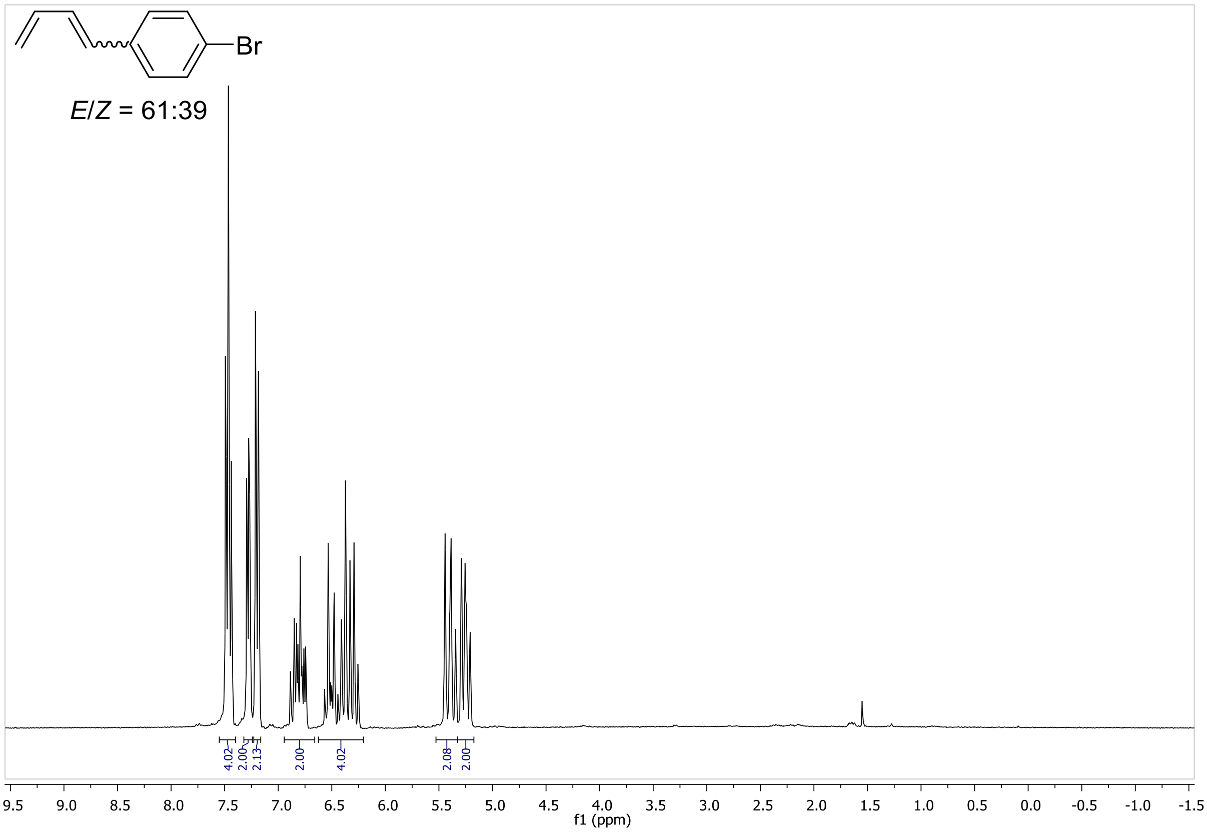

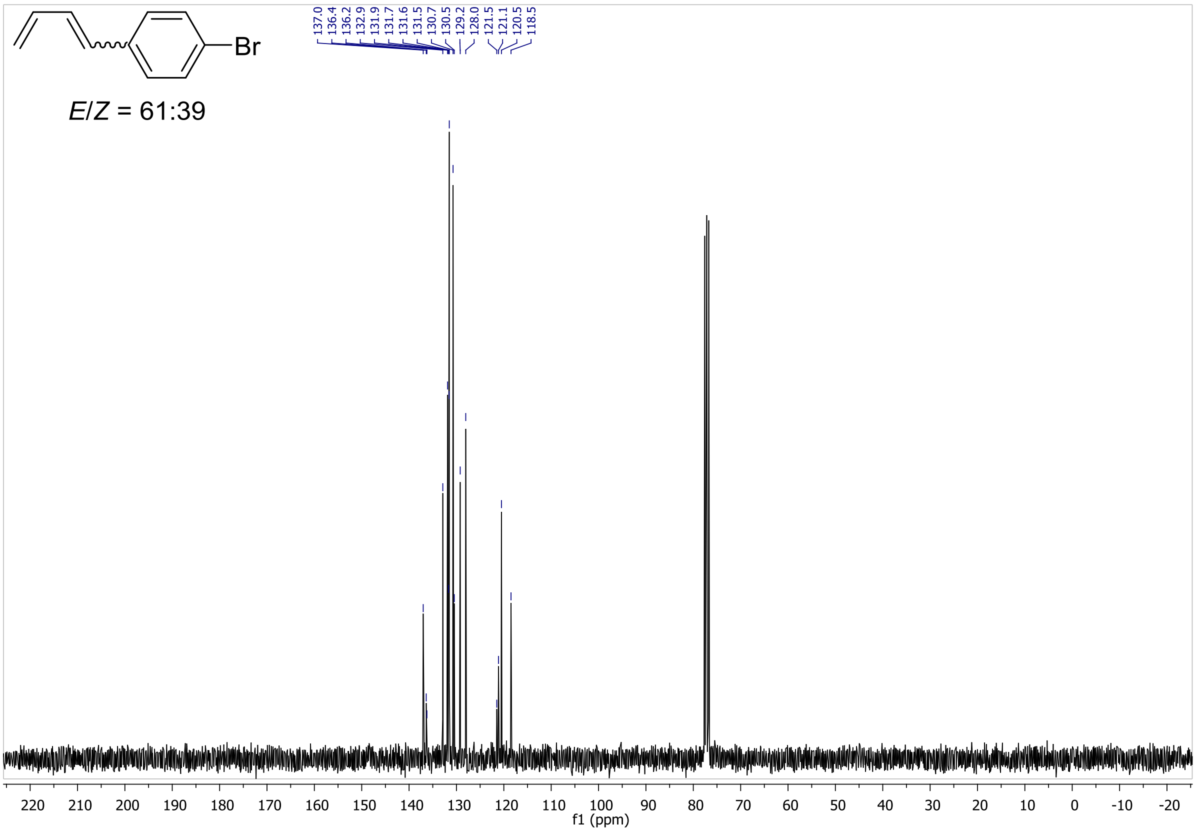

1-(Buta-1,3-dienyl)-2-chlorobenzene (1c)

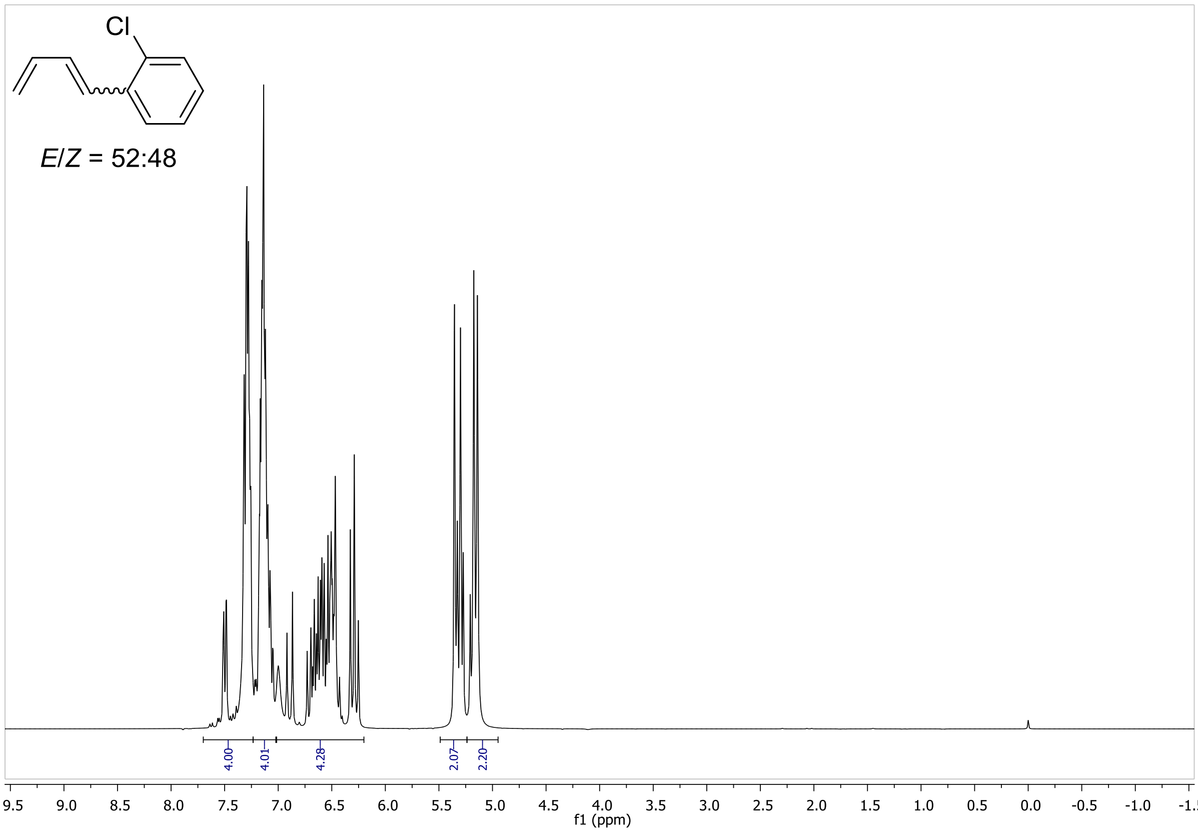

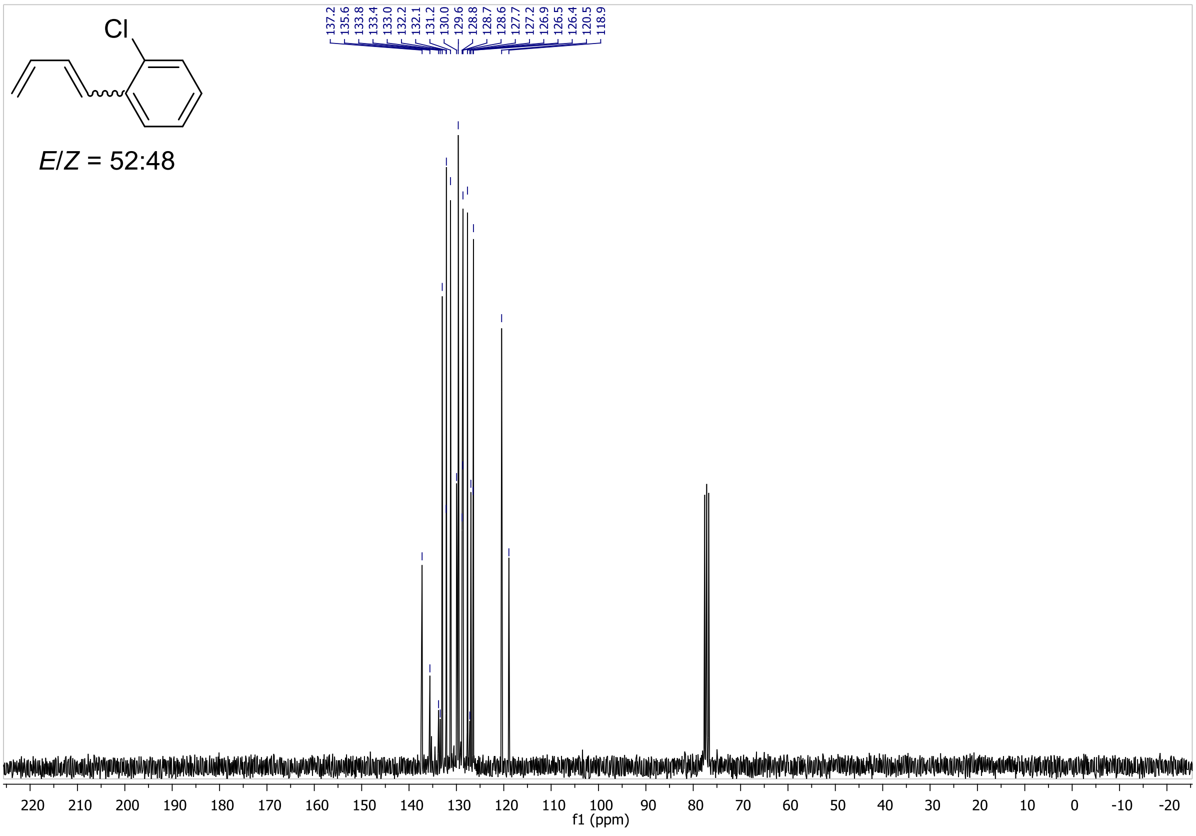

Methyl 3-(buta-1,3-dienyl)benzoate (1d)

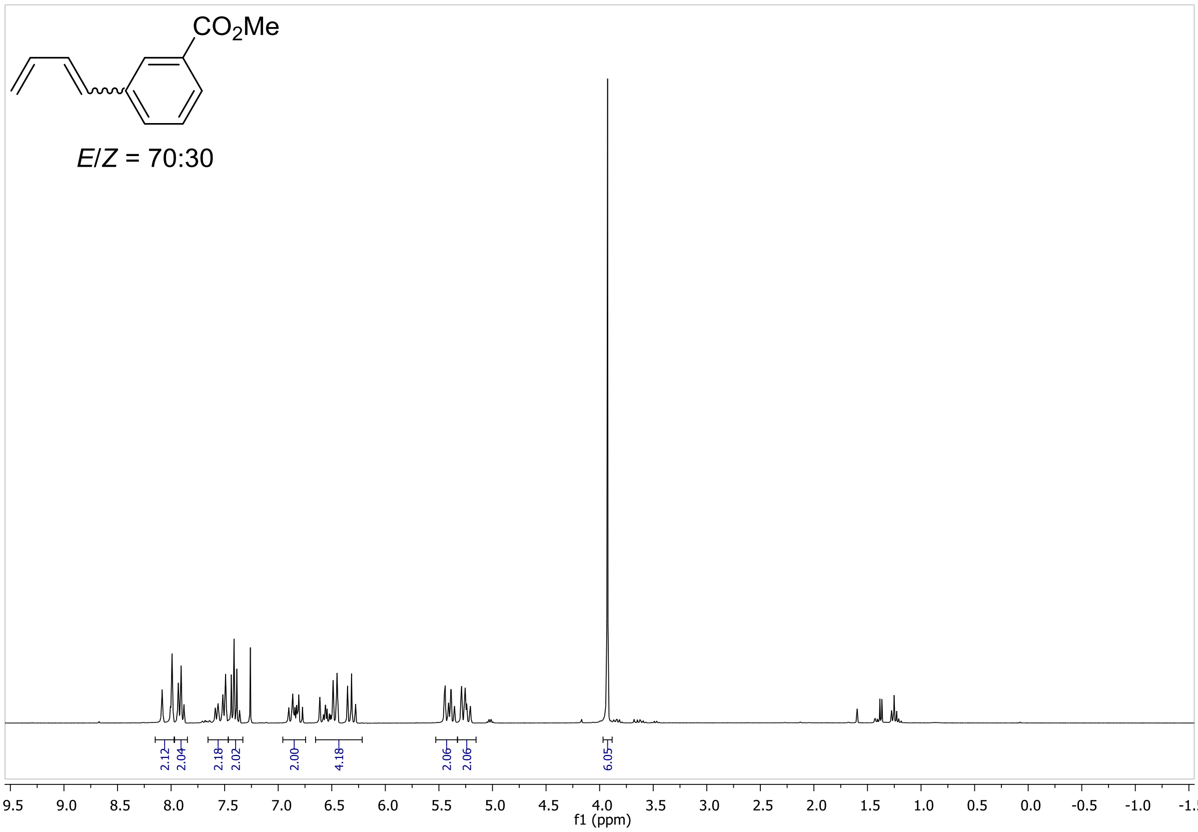

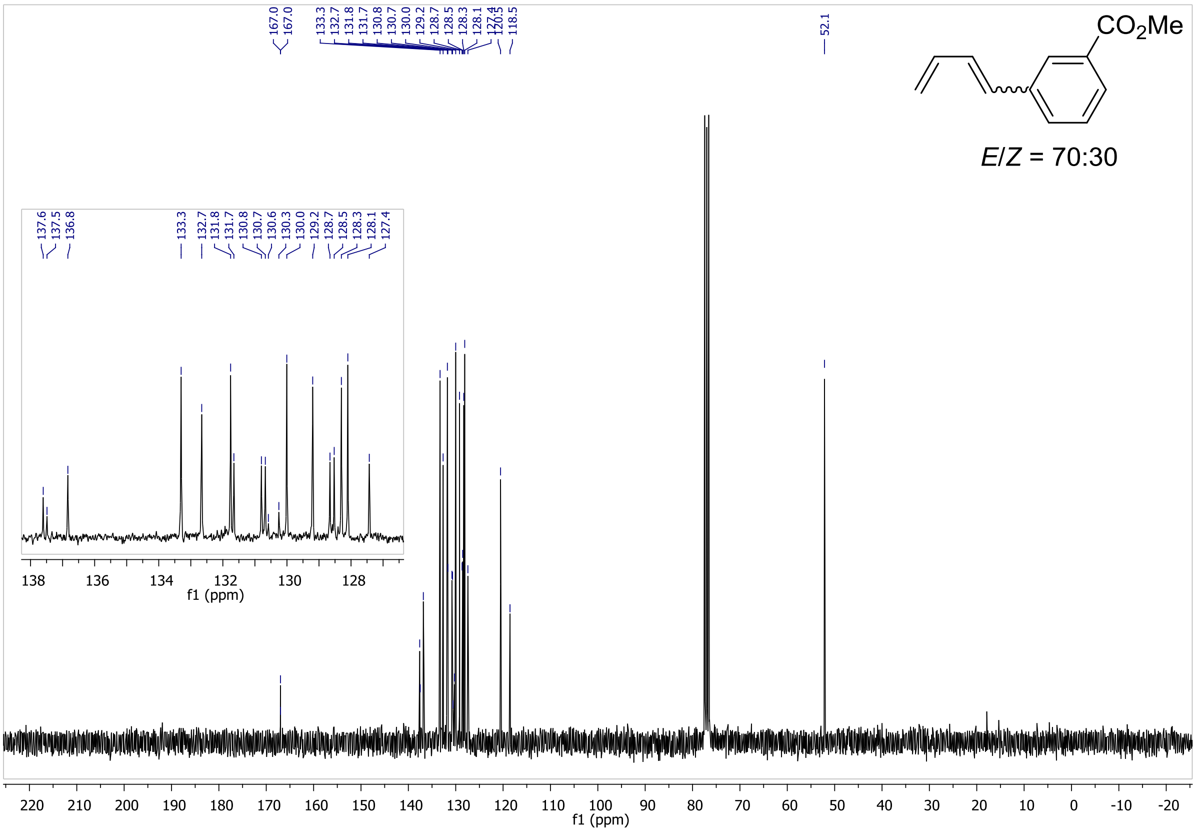

1-(Buta-1,3-dienyl)-4-(trifluoromethyl)benzene (1e)

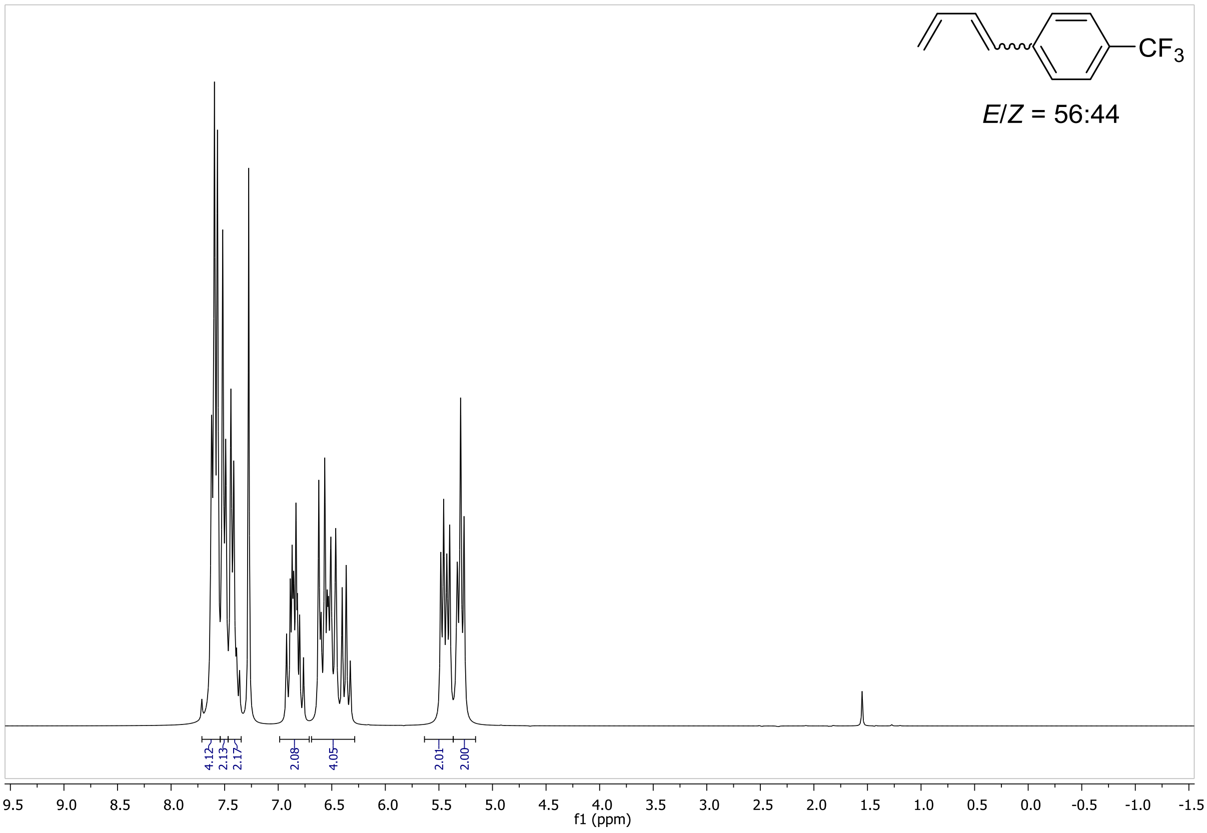

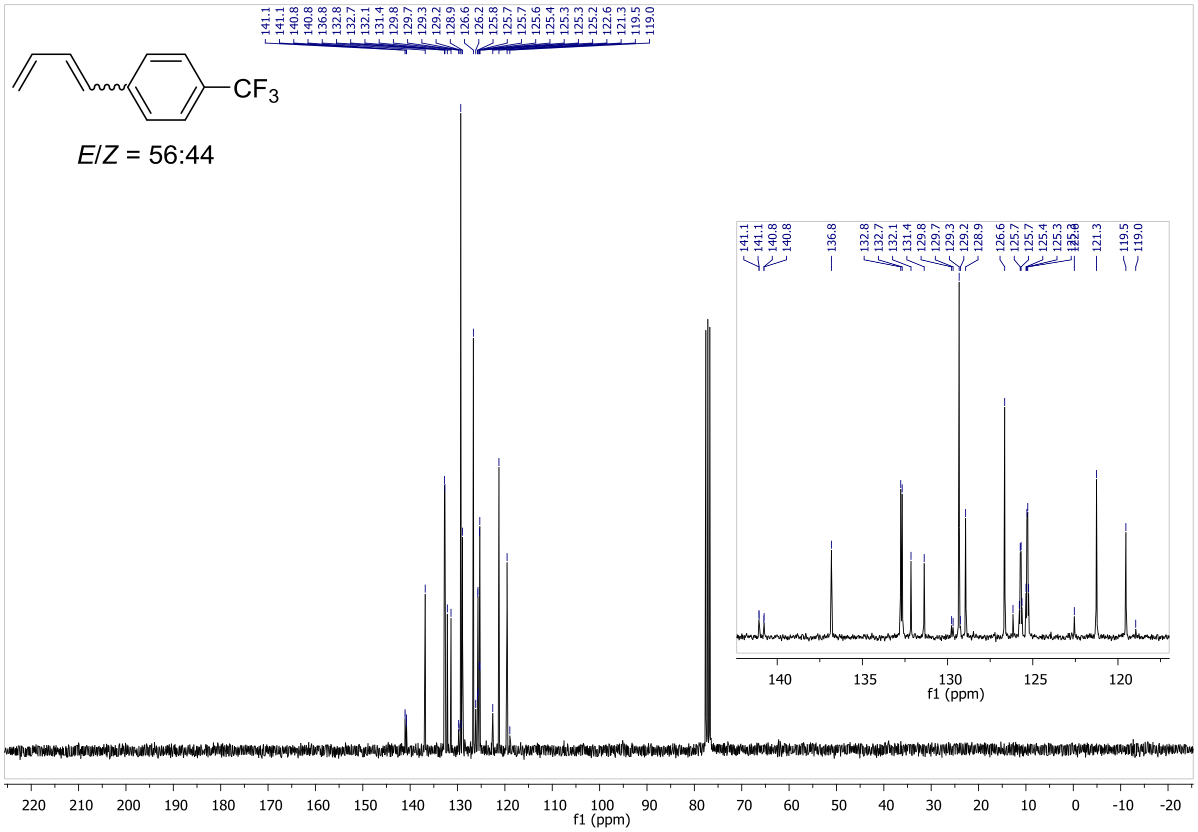

1-(Buta-1,3-dienyl)-4-methoxybenzene (1f)

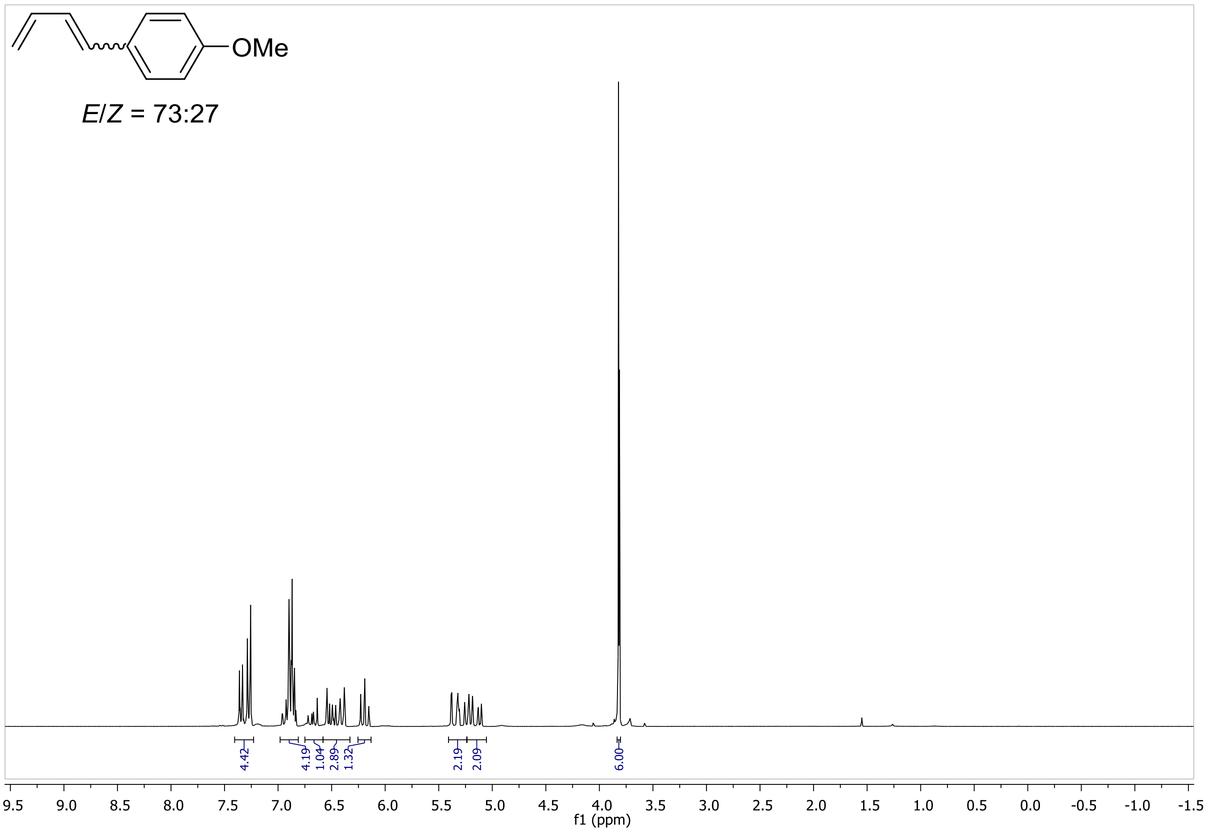

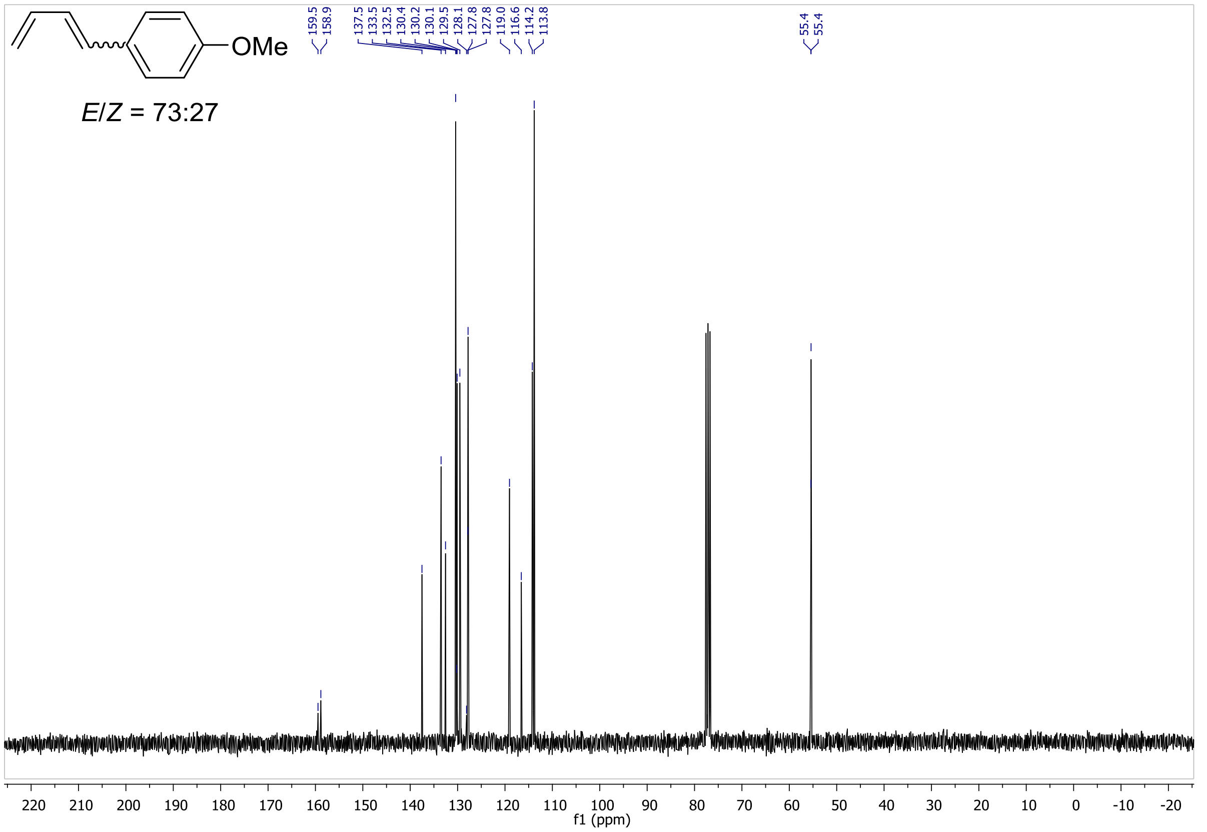

2-(Buta-1,3-dienyl)-1,3,5-trimethoxybenzene (1g)

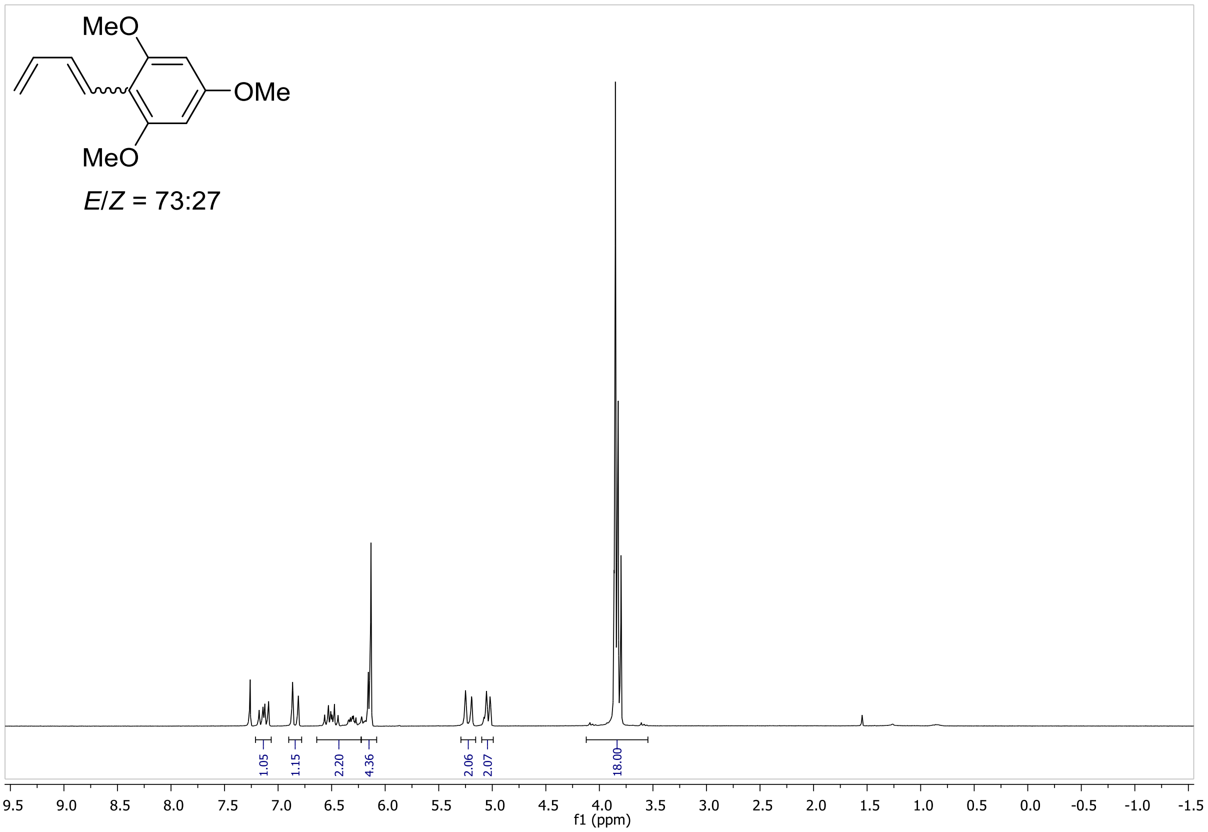

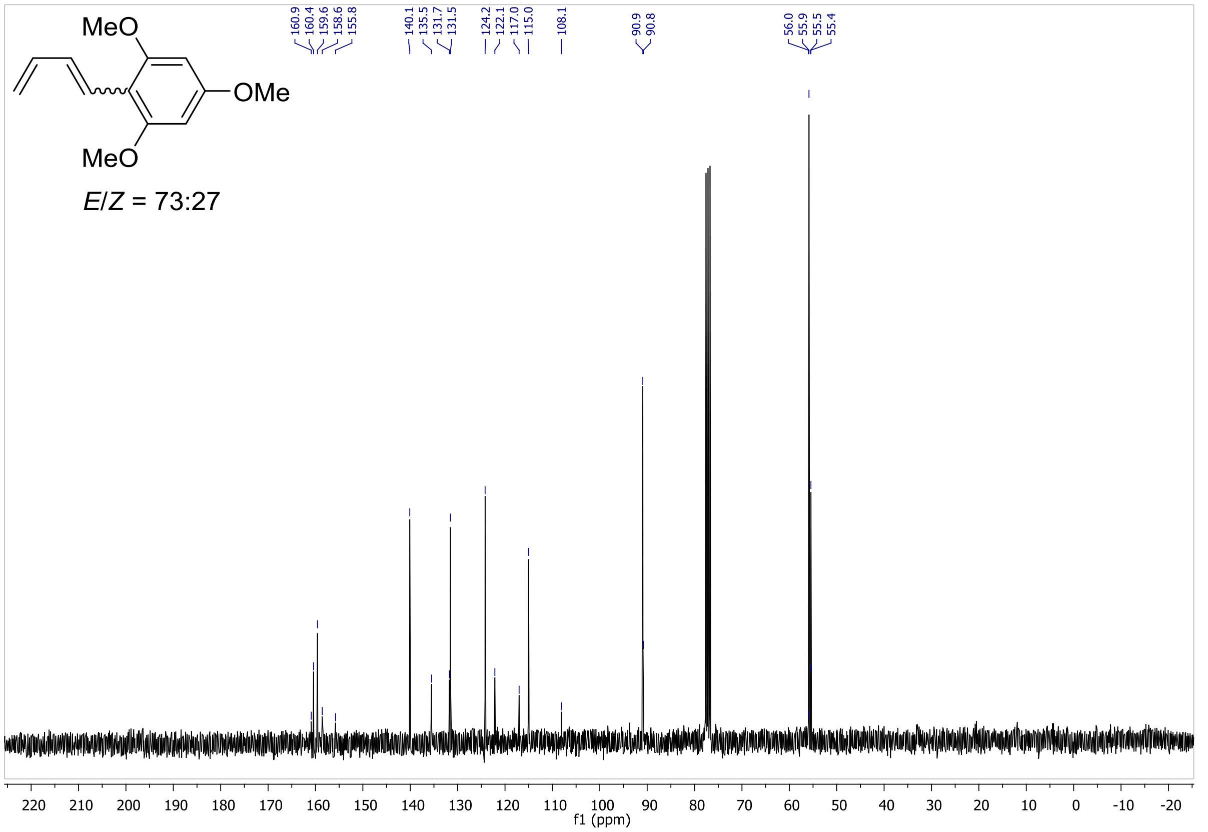

4-(Buta-1,3-dienyl)-1,2-dimethoxybenzene (1h)

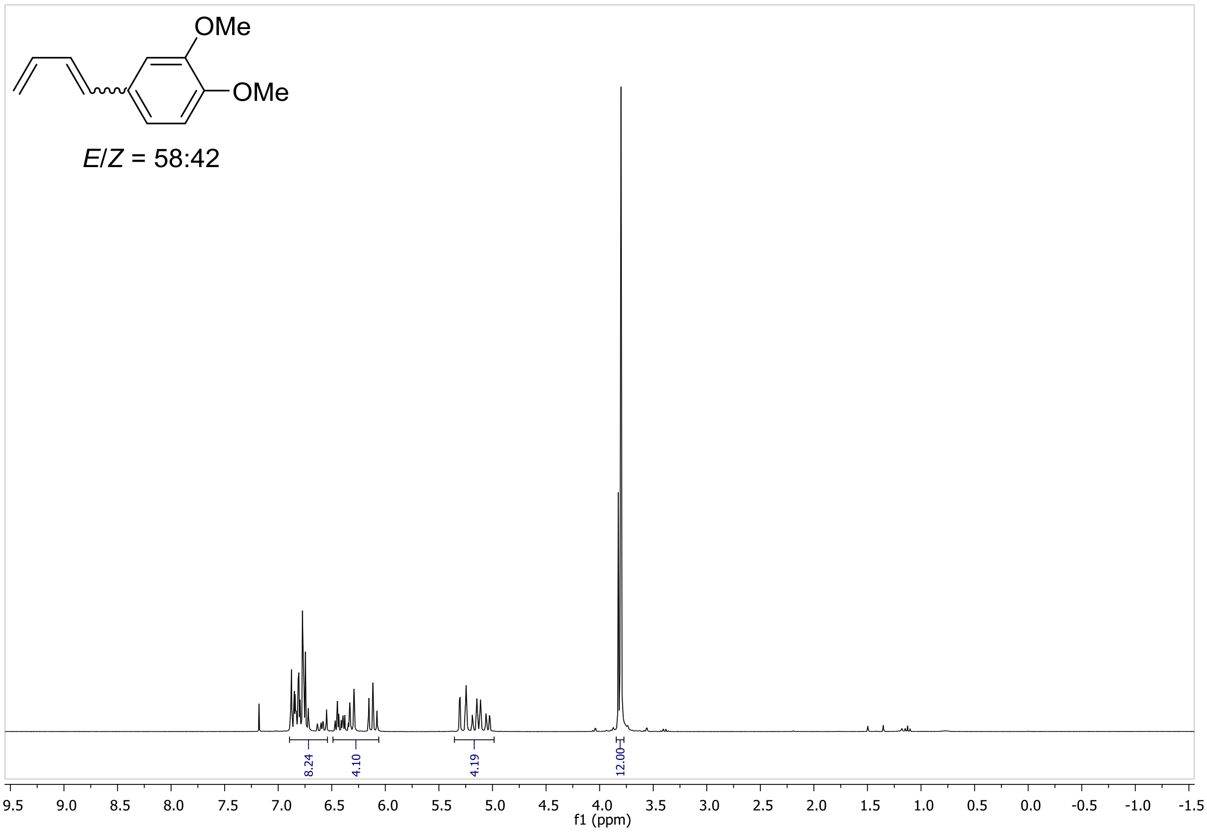

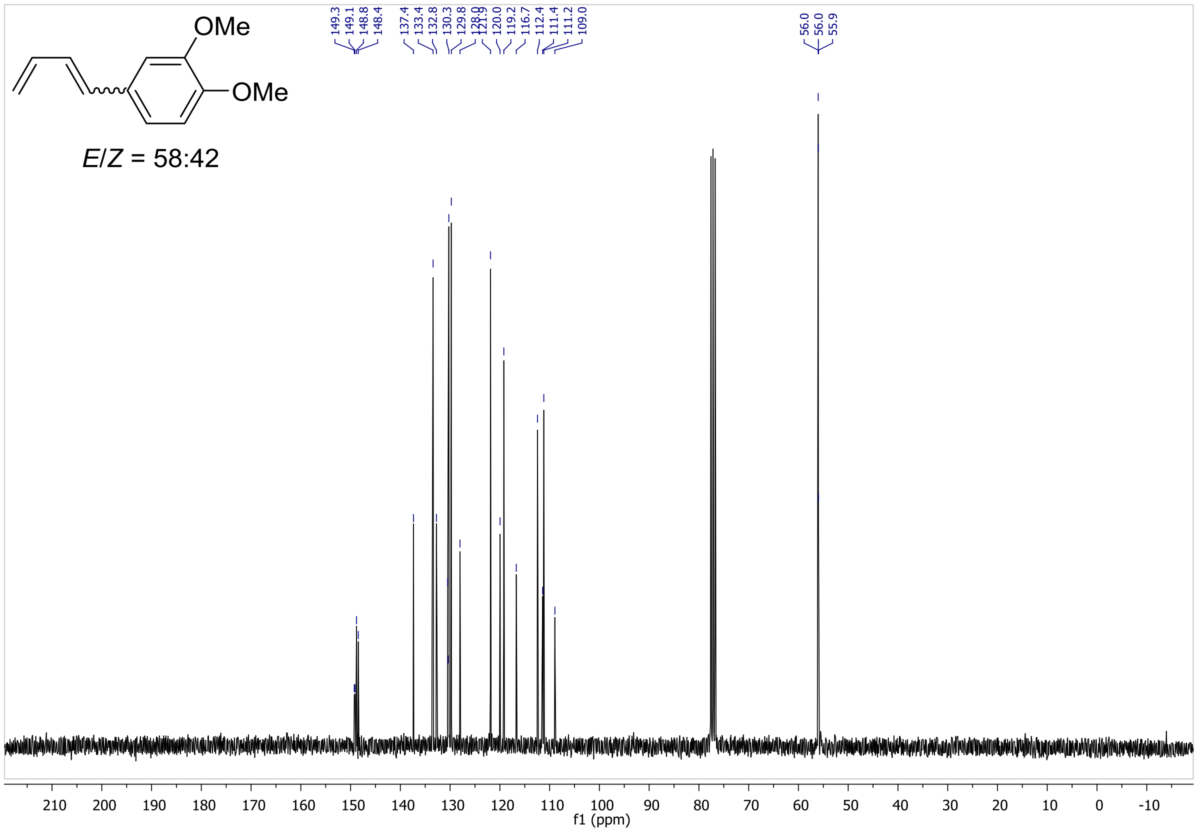

1-Bromo-2-(buta-1,3-dienyl)-4,5-dimethoxybenzene (1i)

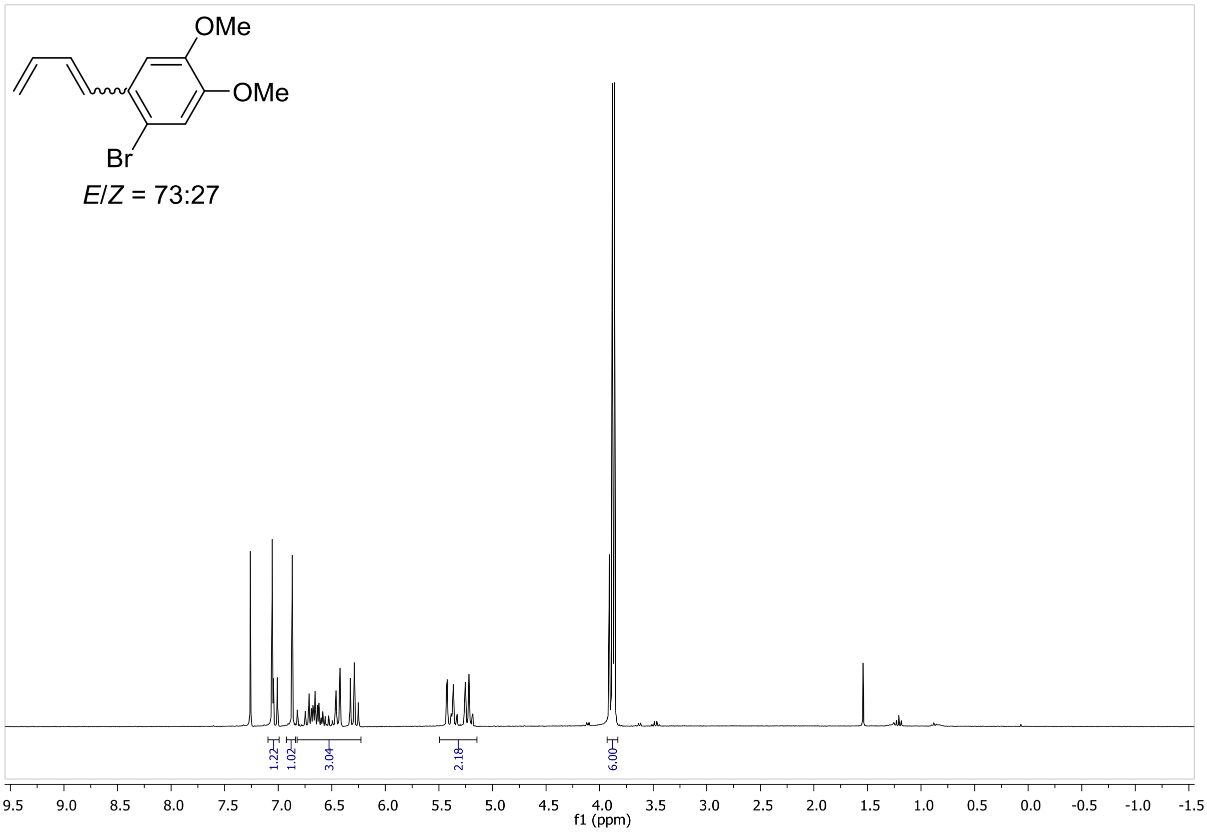

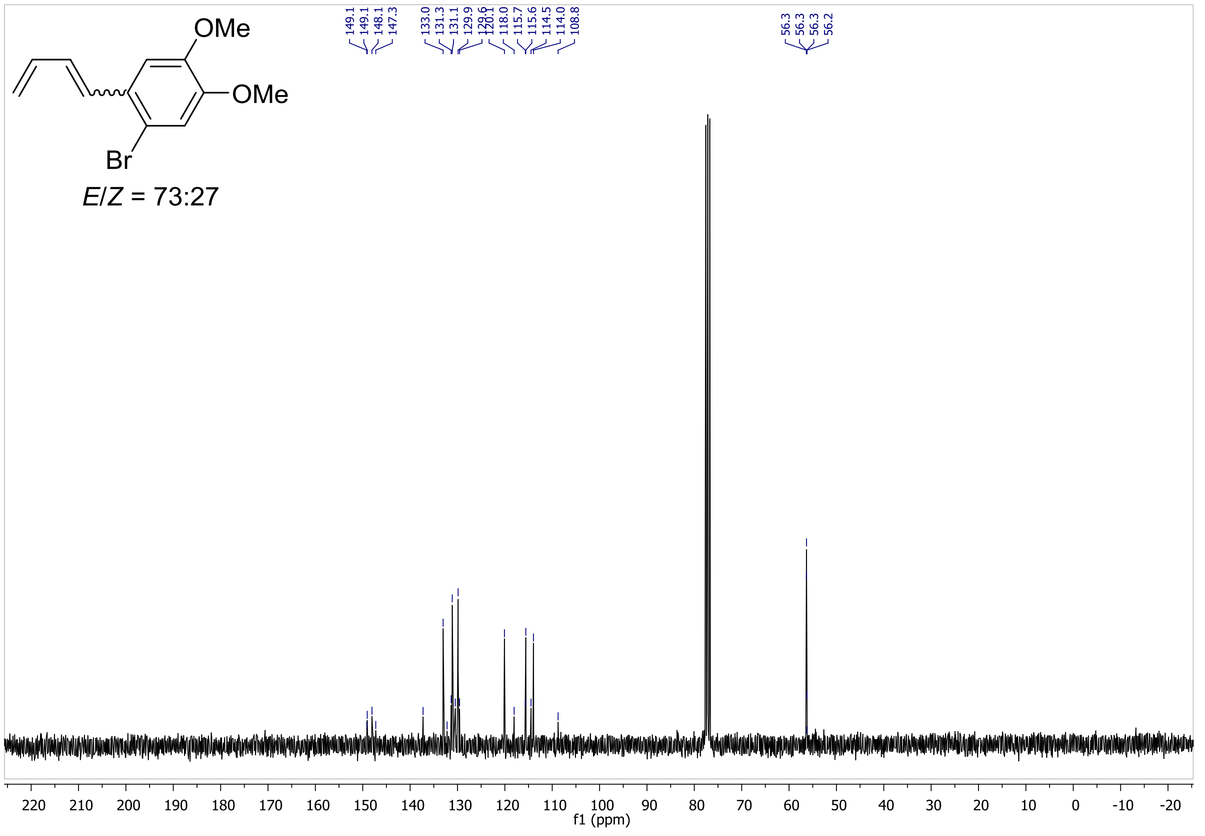

3-(Buta-1,3-dienyl)furan (1j)

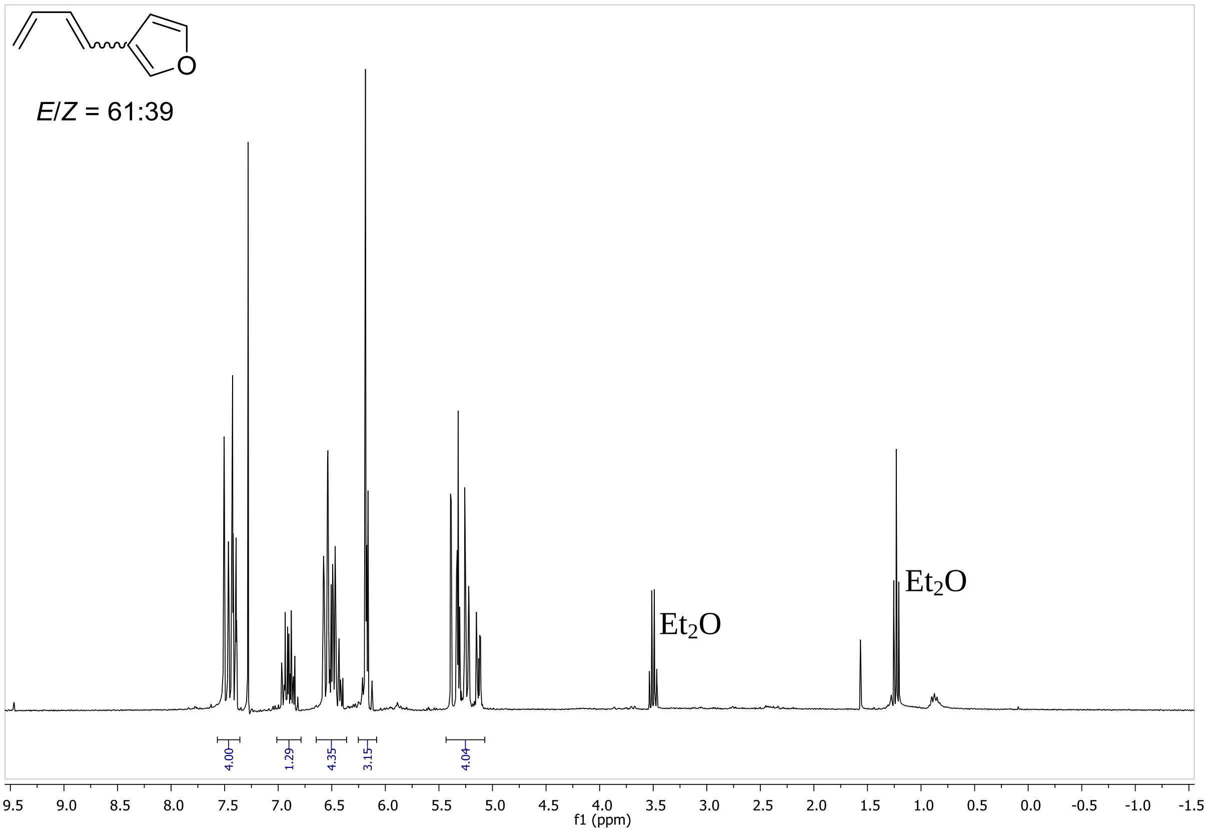

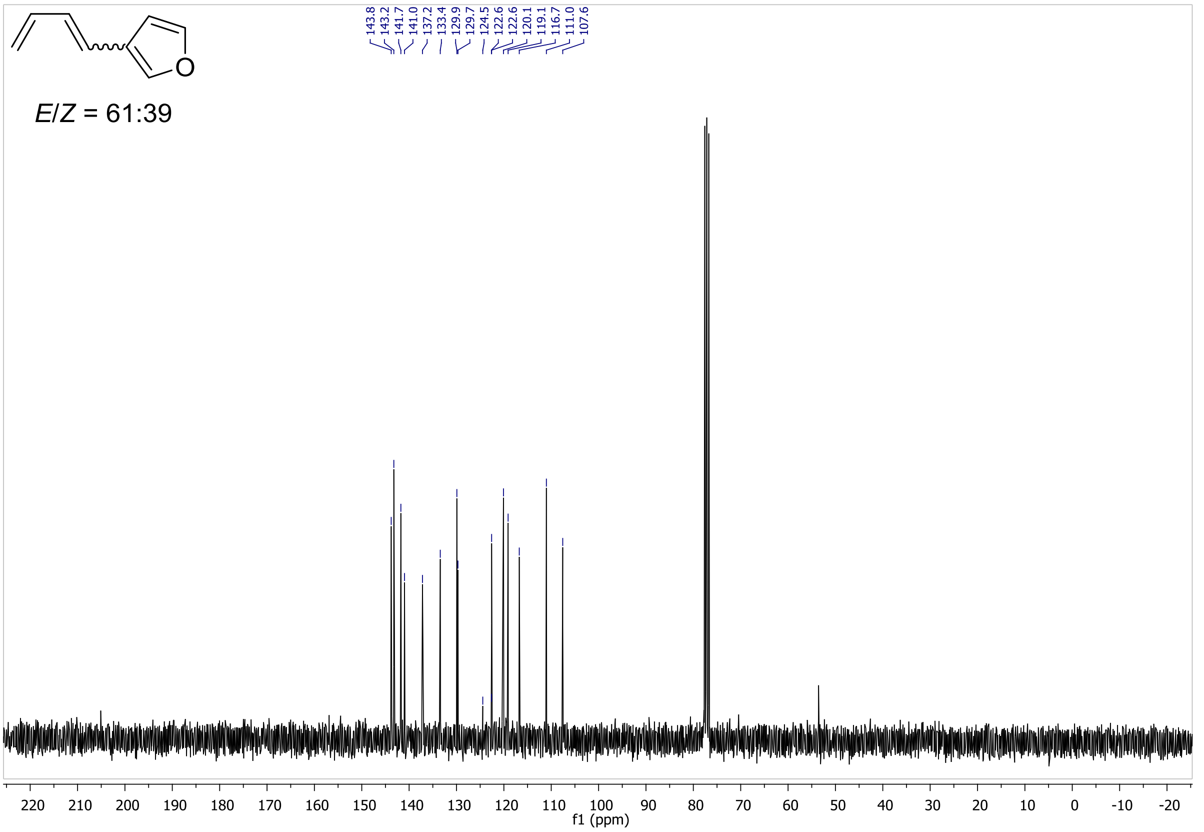

2-(Buta-1,3-dienyl)thiophene (1k)

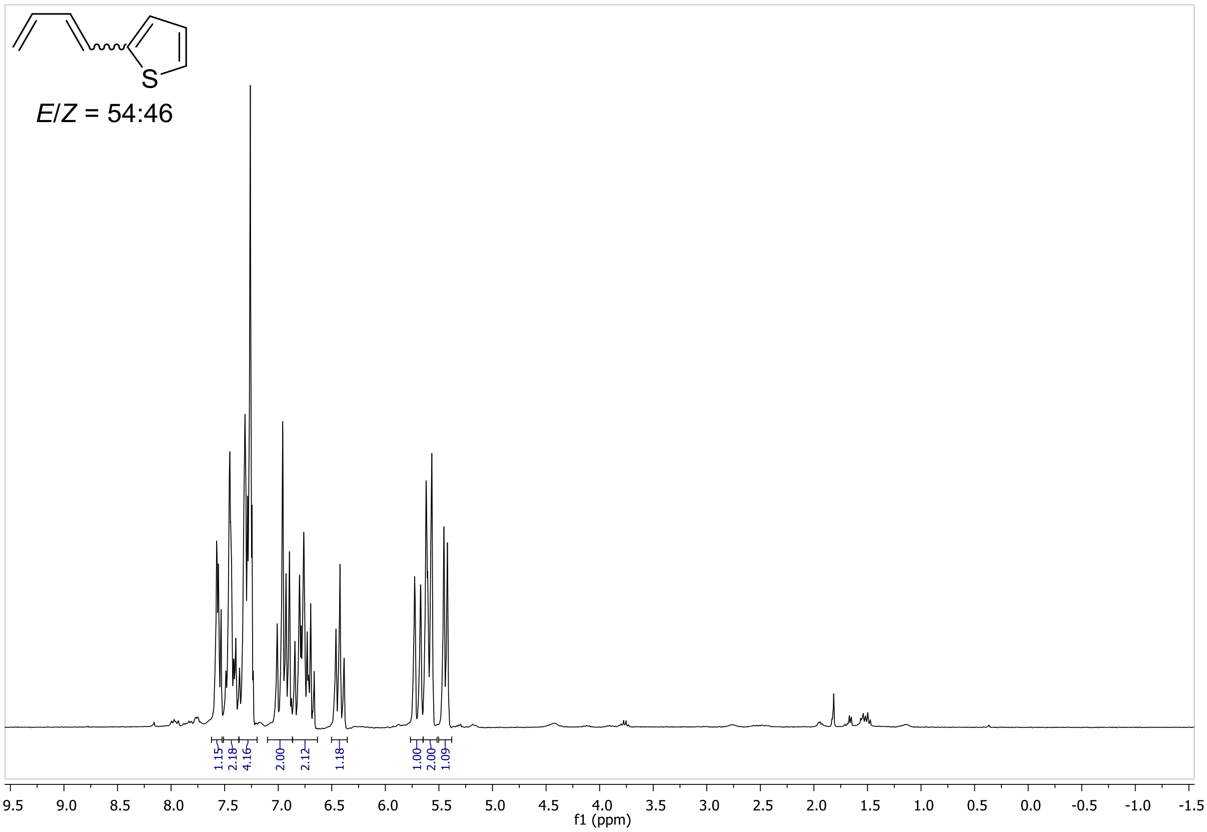

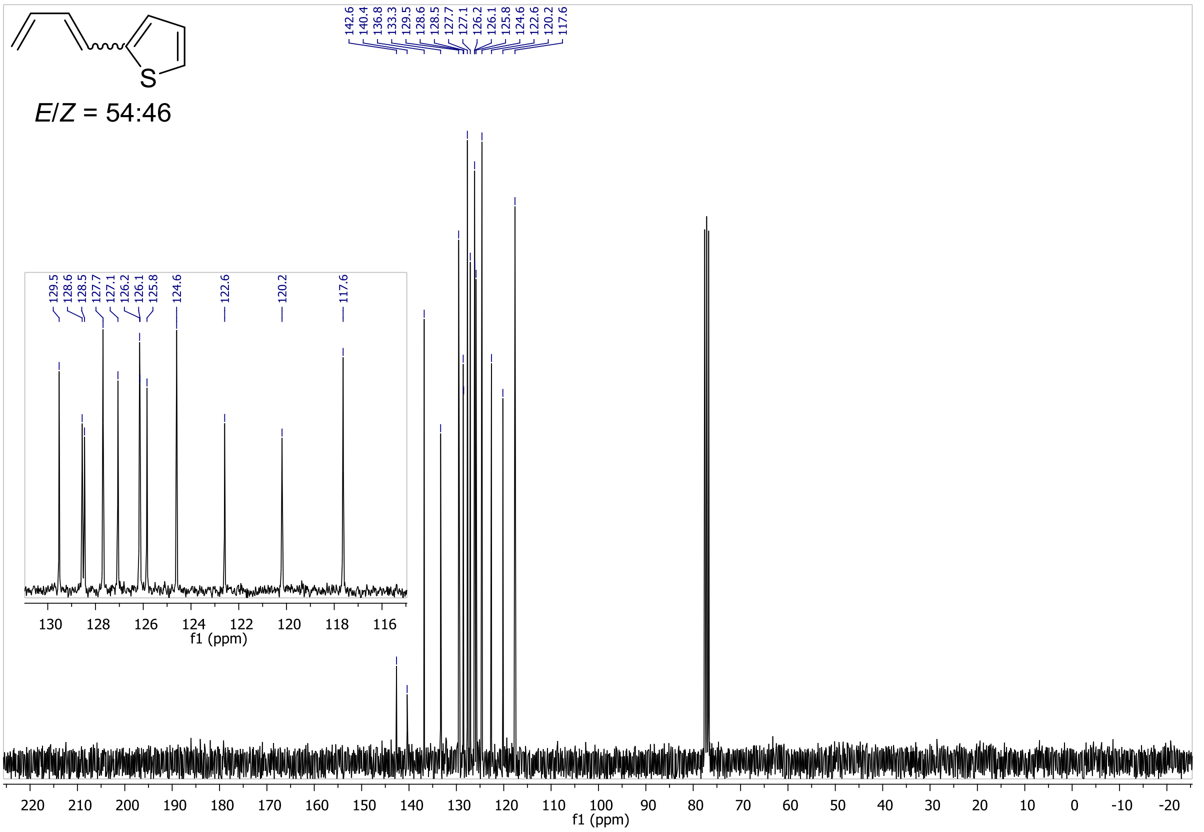

(*Z*)-2-Methylene-6-phenylhex-4-en-1-ol (2a)

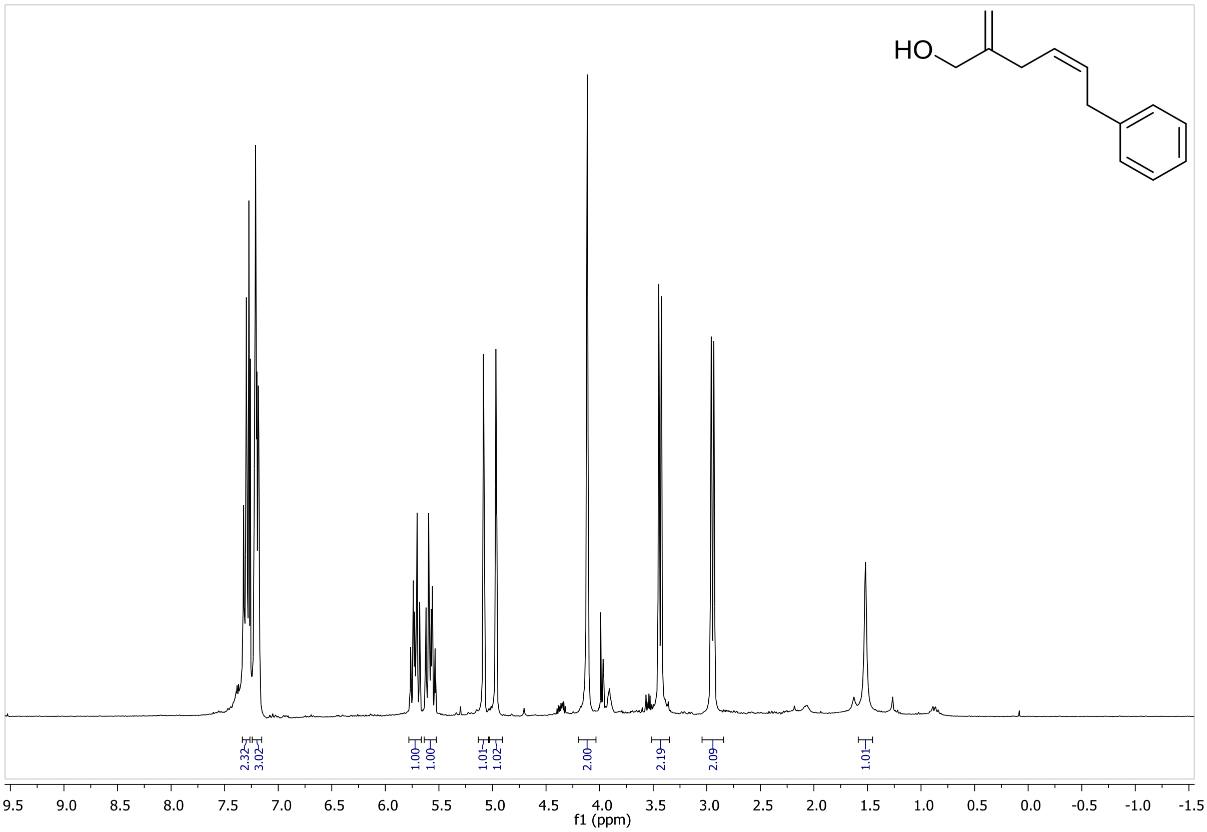

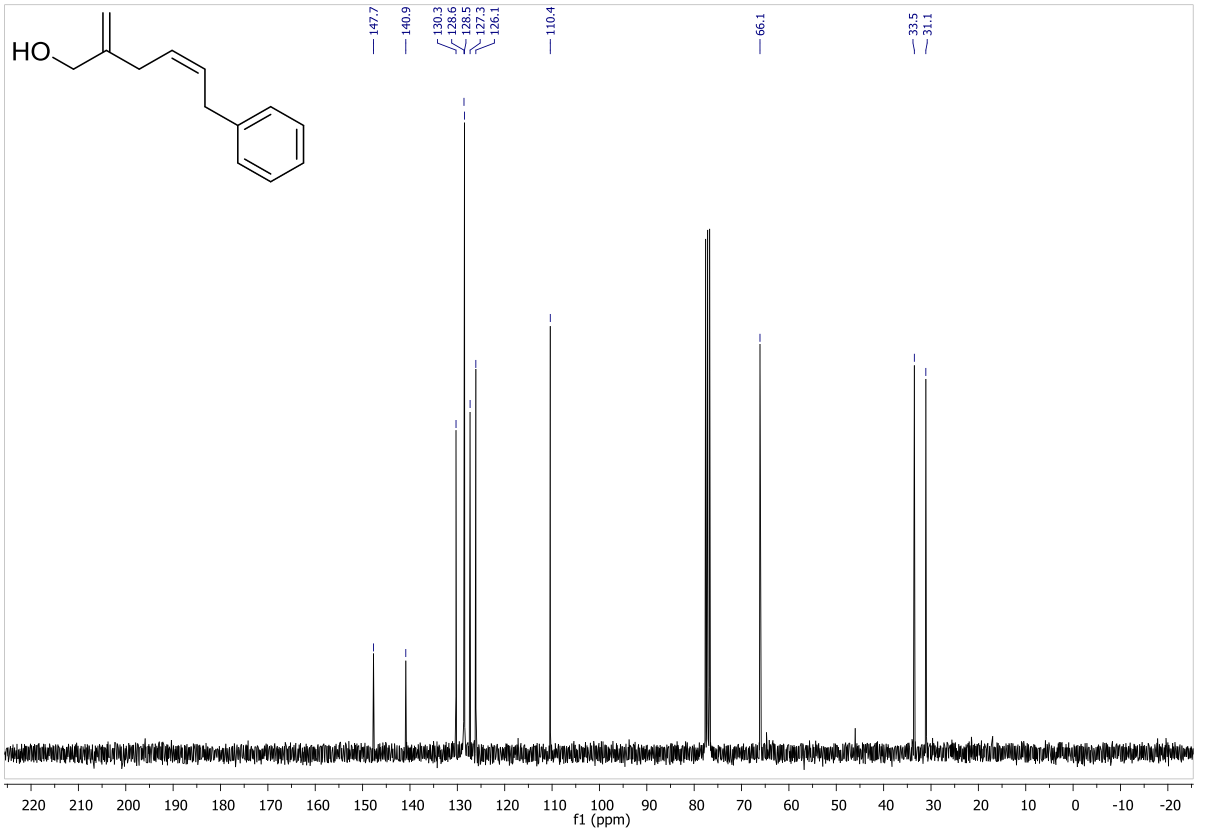

(*Z*)-6-(4-Bromophenyl)-2-methylenehex-4-en-1-ol (2b)

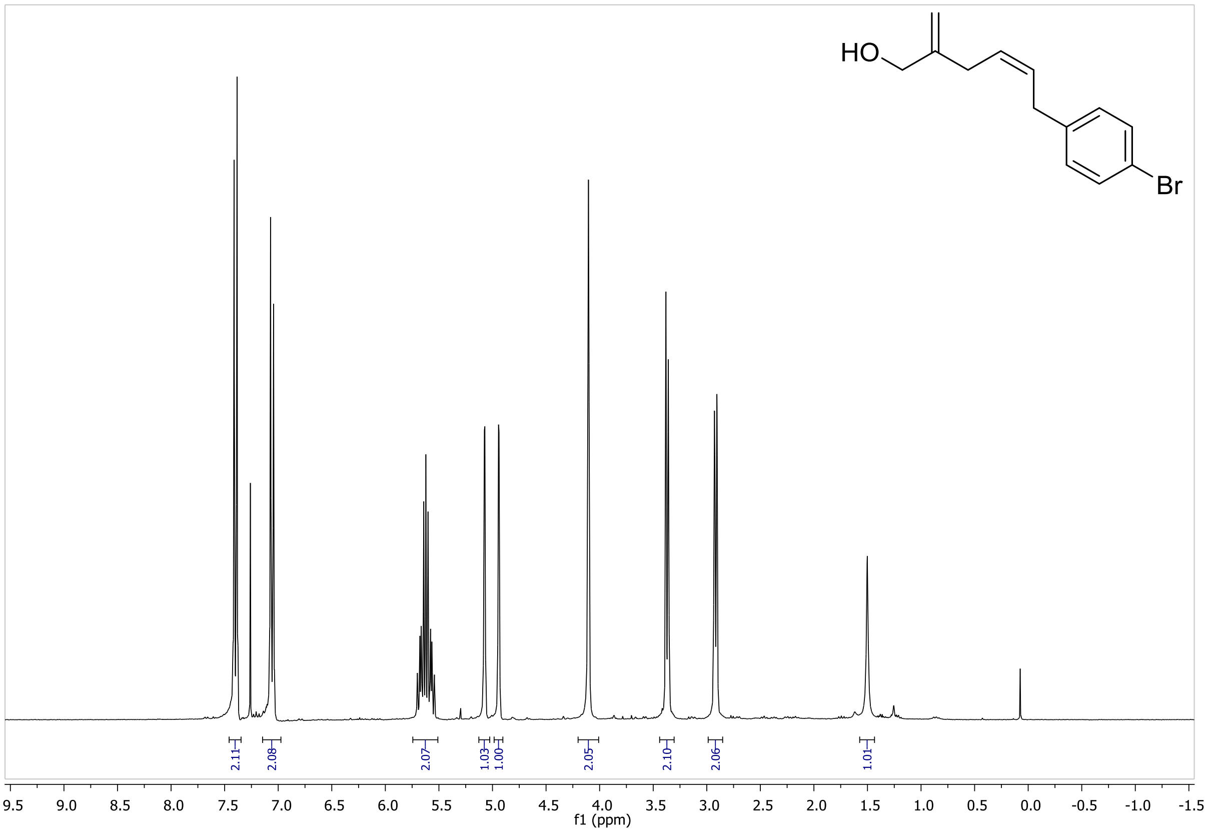

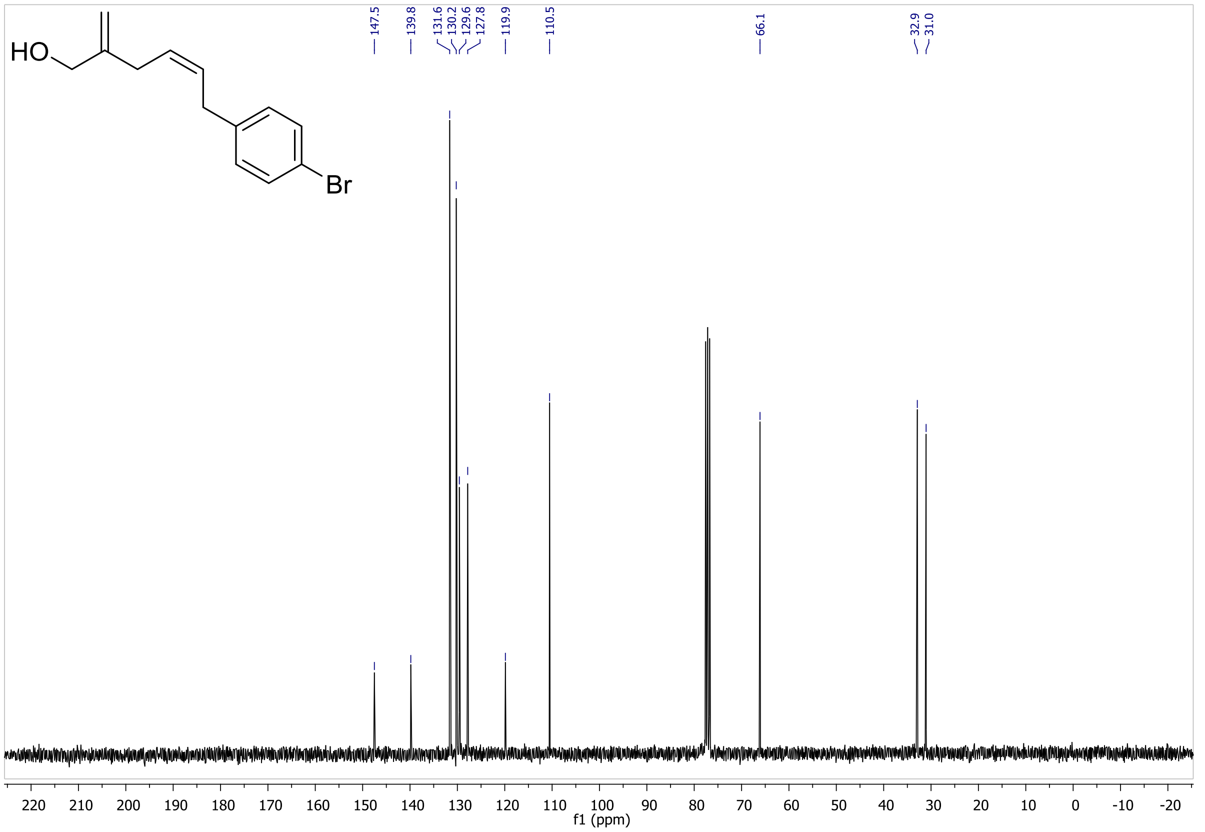

(*Z*)-6-(2-Chlorophenyl)-2-methylenehex-4-en-1-ol (2c)

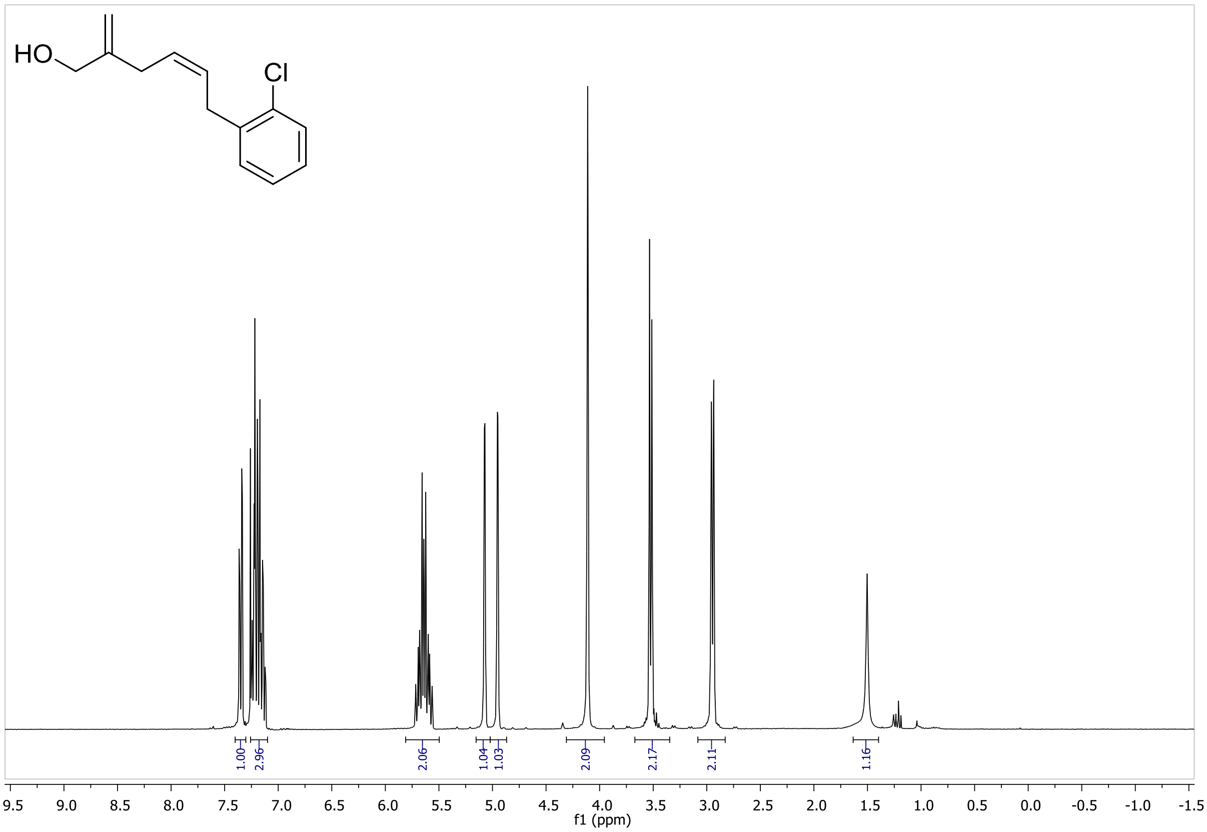

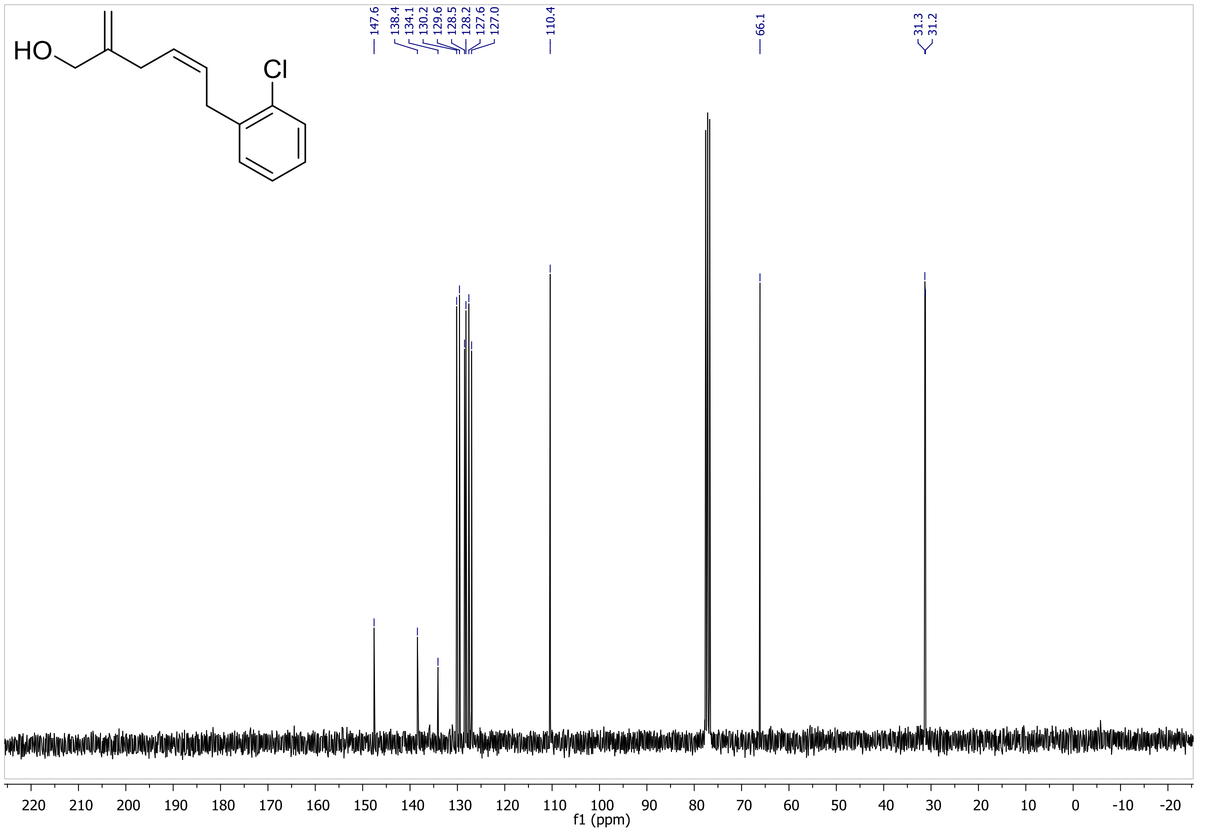

(*Z*)-Methyl 3-(5-(hydroxymethyl)hexa-2,5-dienyl)benzoate (2d)

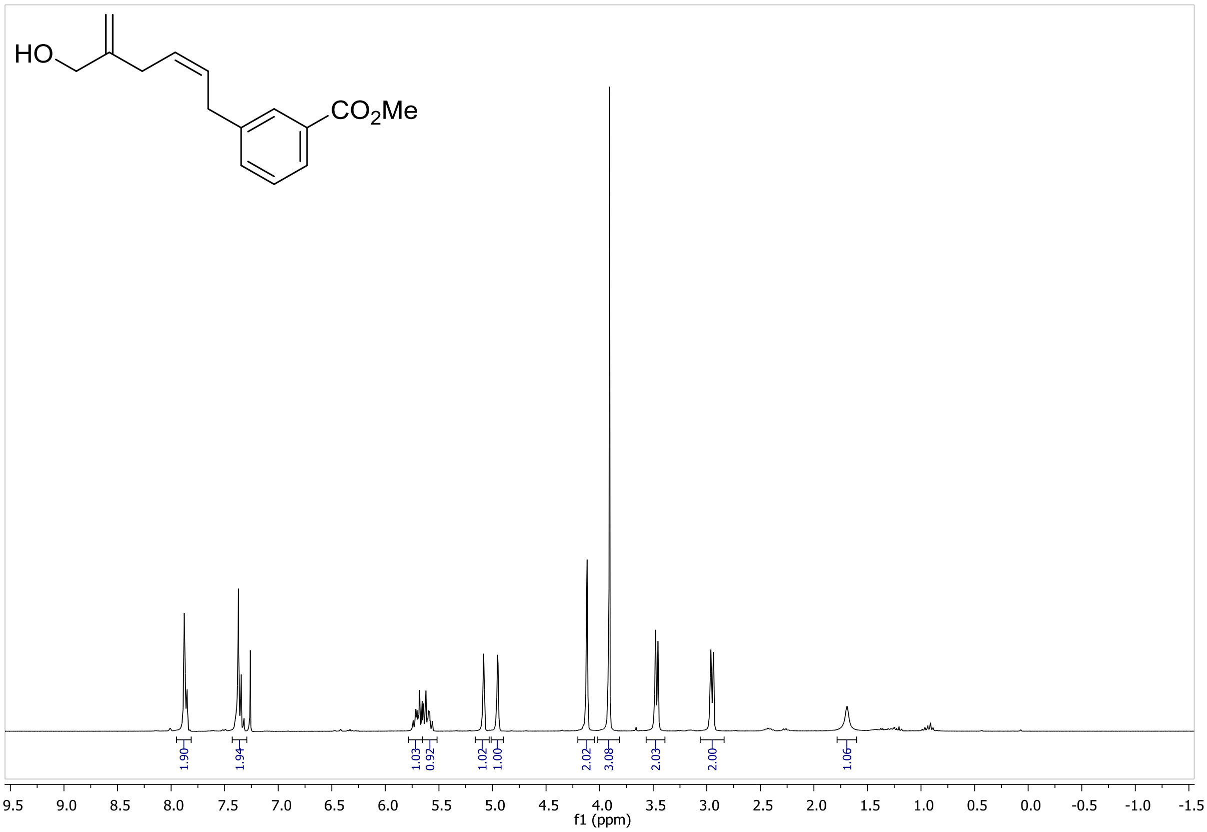

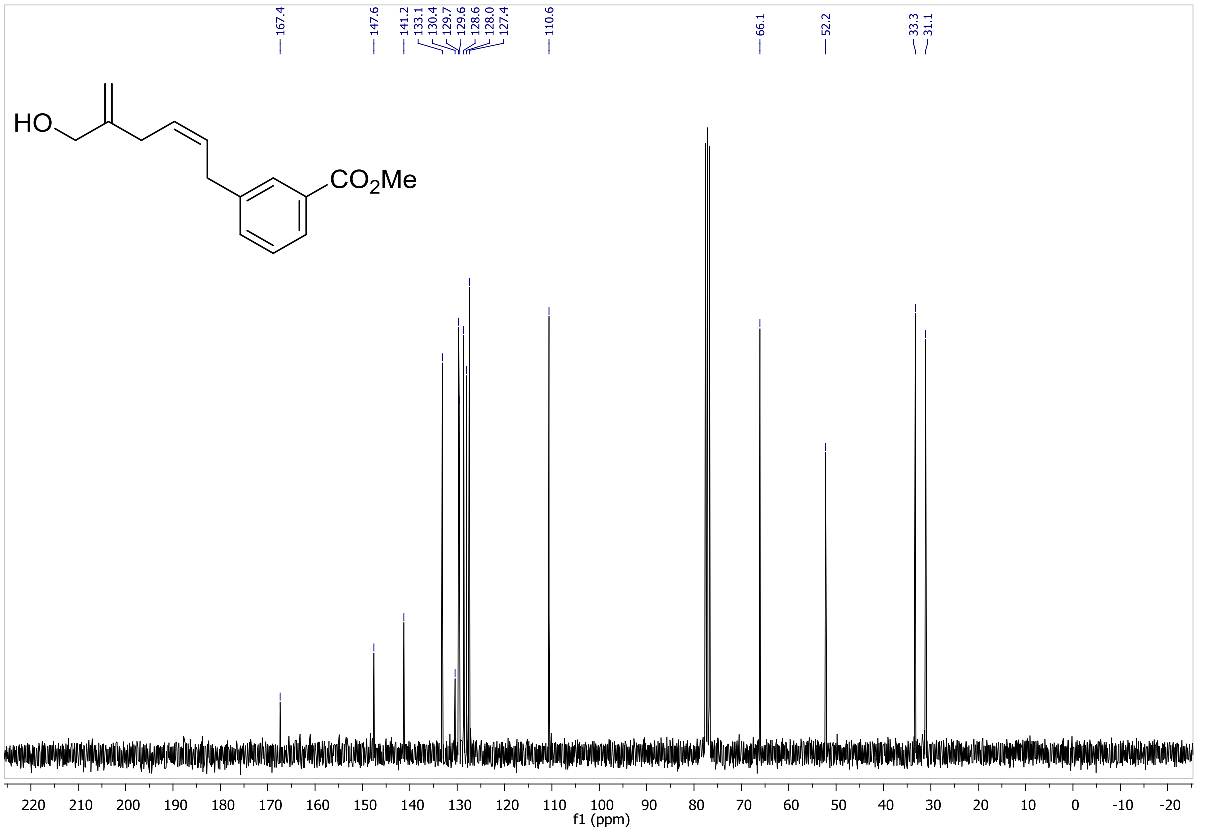

(*Z*)-2-Methylene-6-(4-(trifluoromethyl)phenyl)hex-4-en-1-ol (2e)

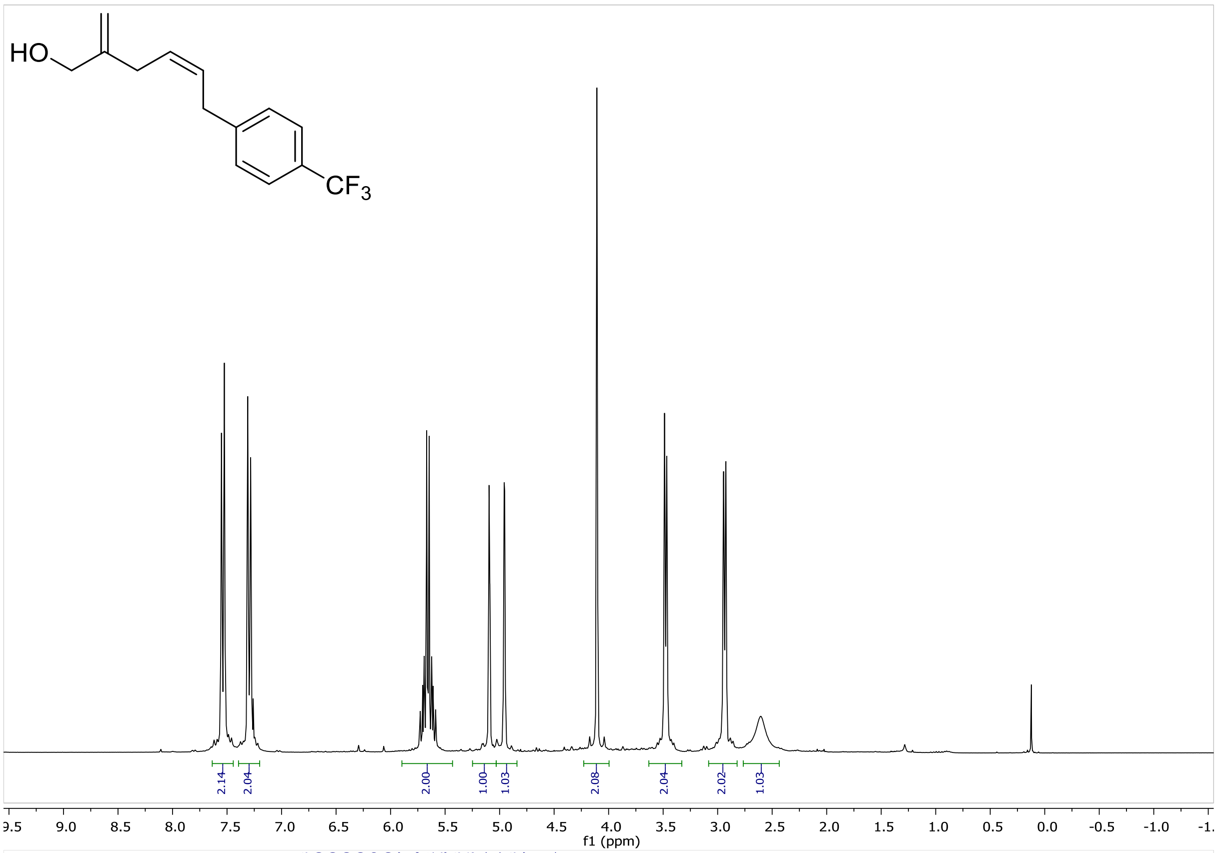

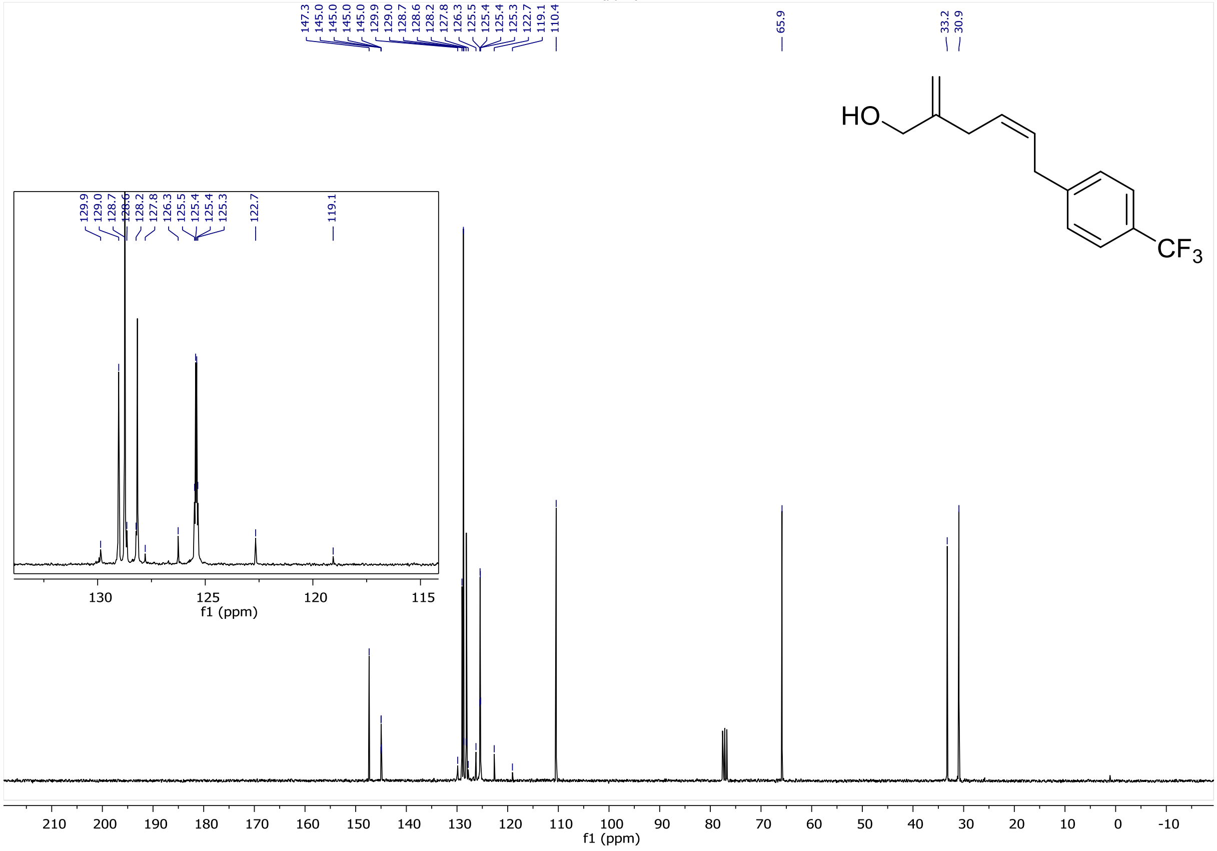

(*Z*)-6-(4-Methoxyphenyl)-2-methylenehex-4-en-1-ol (2f)

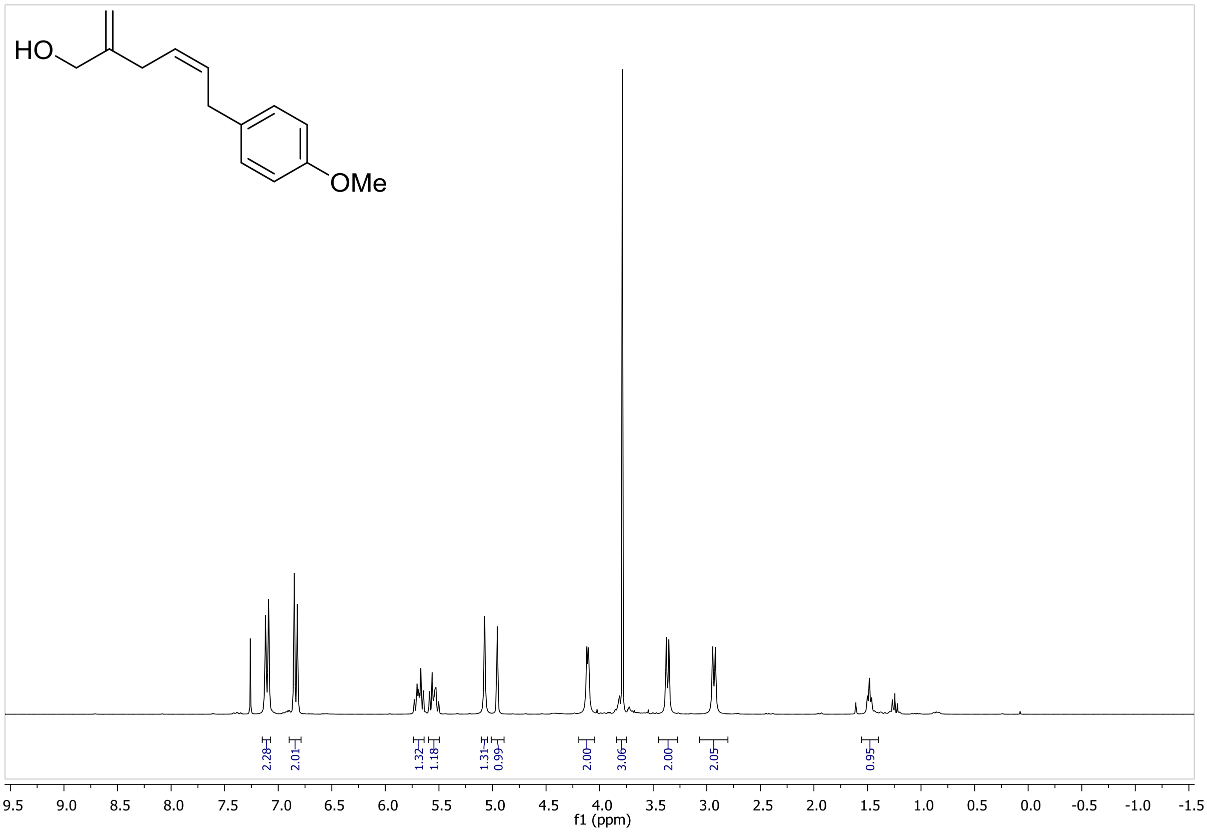

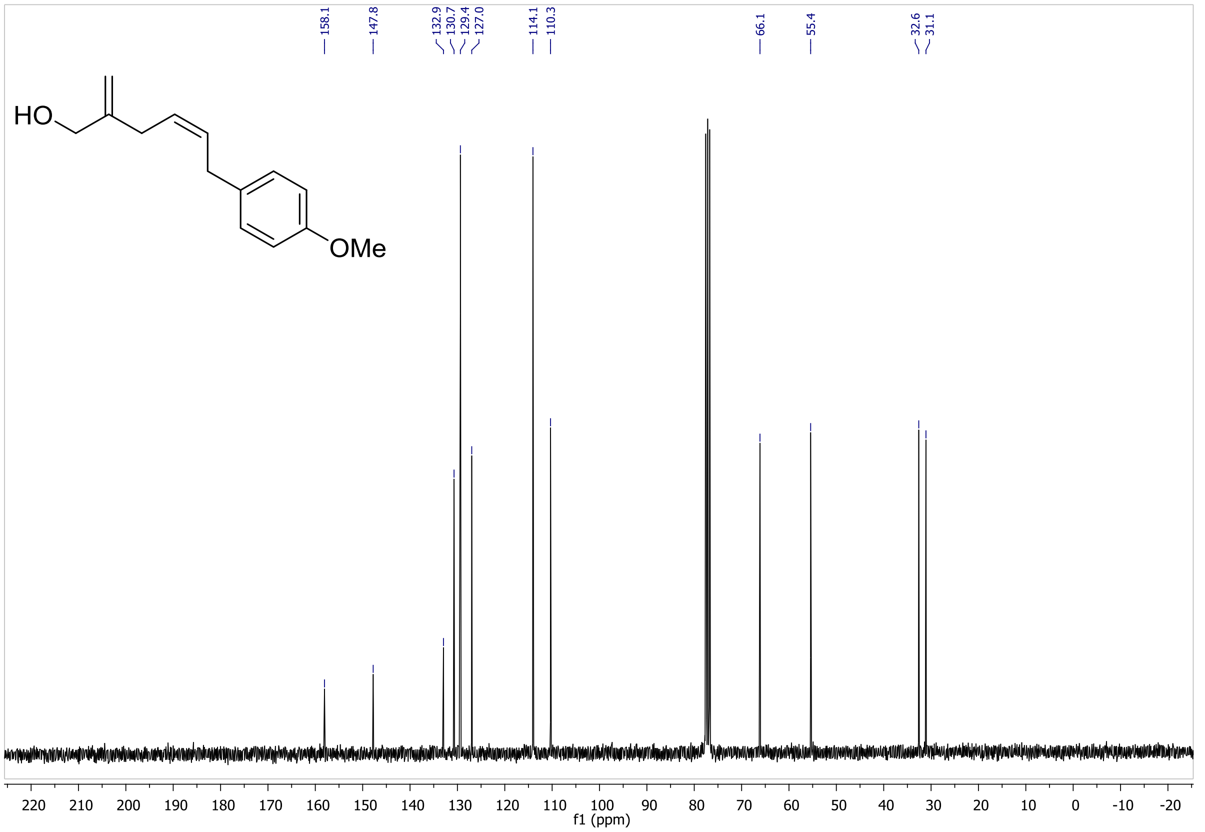

(Z)-2-Methylene-6-(2,4,6-trimethoxyphenyl)hex-4-en-1-ol (2g)

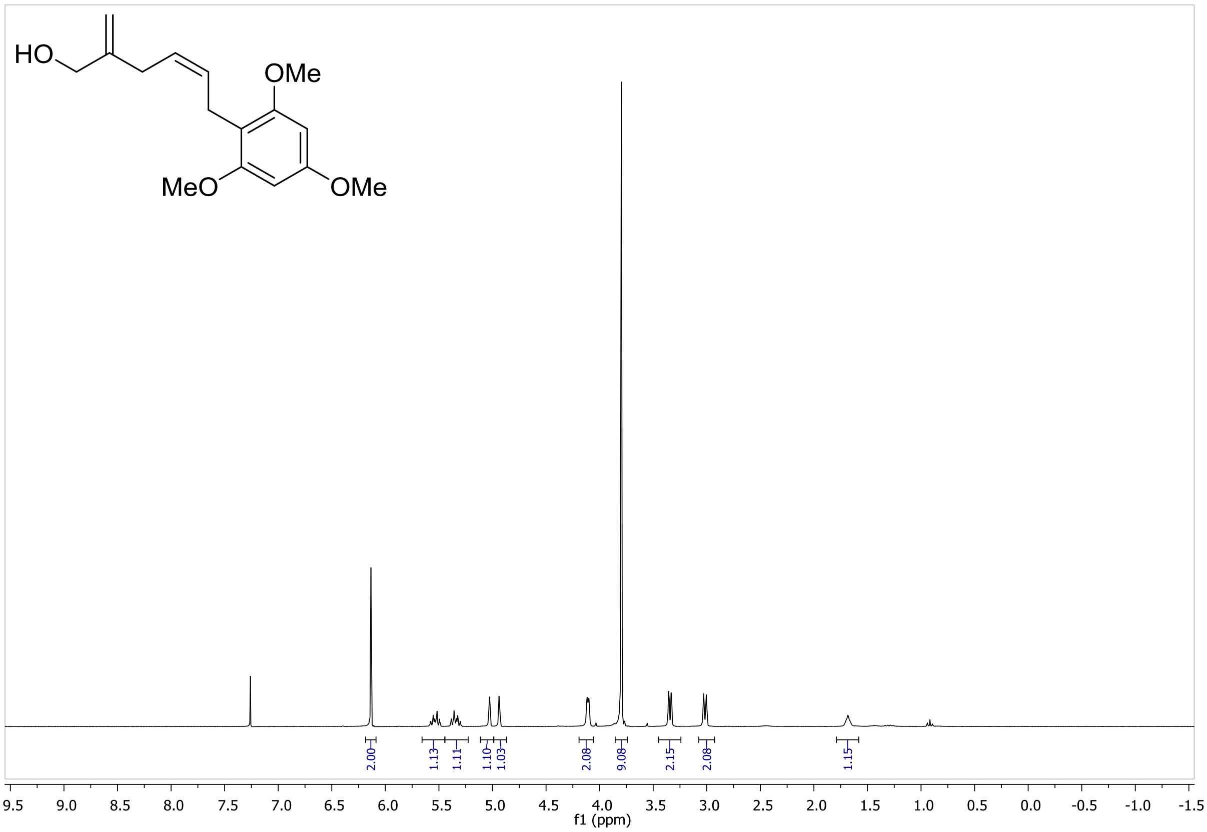

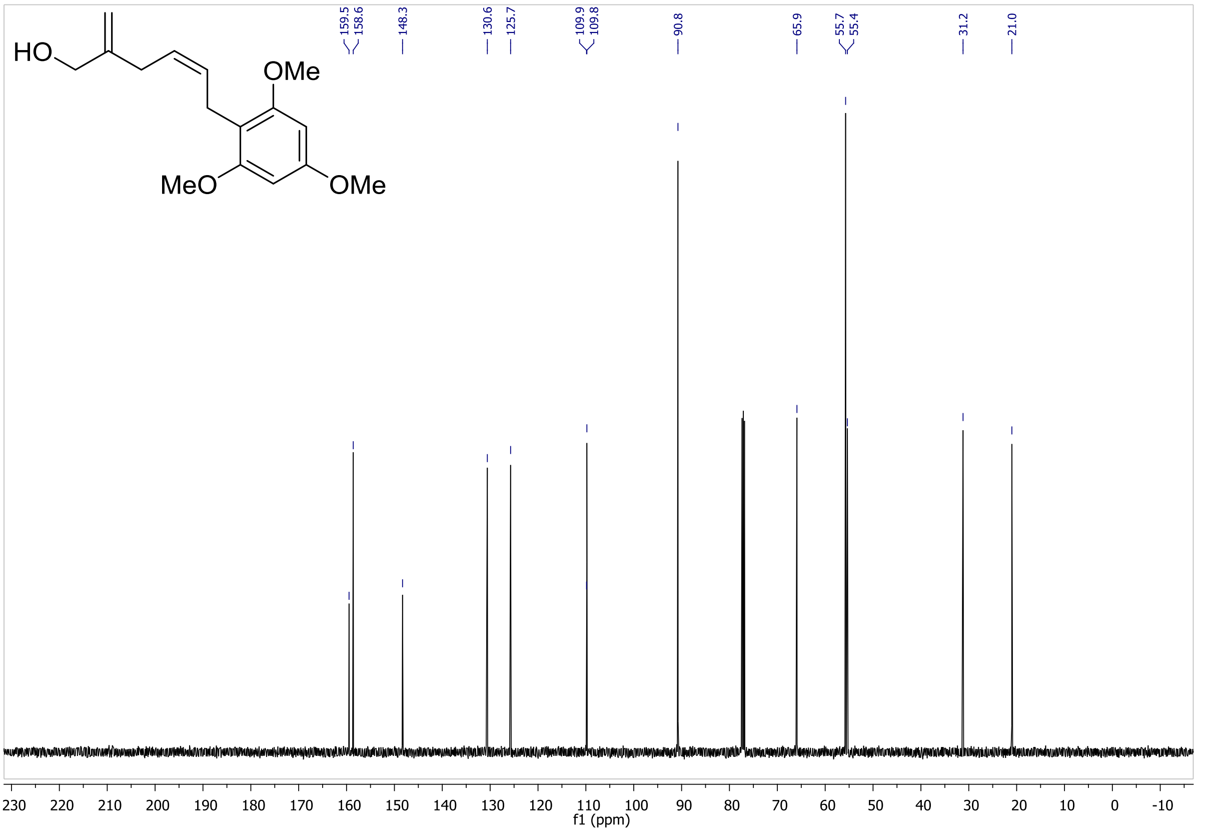

(*Z*)-6-(3,4-Dimethoxyphenyl)-2-methylenehex-4-en-1-ol (2h)

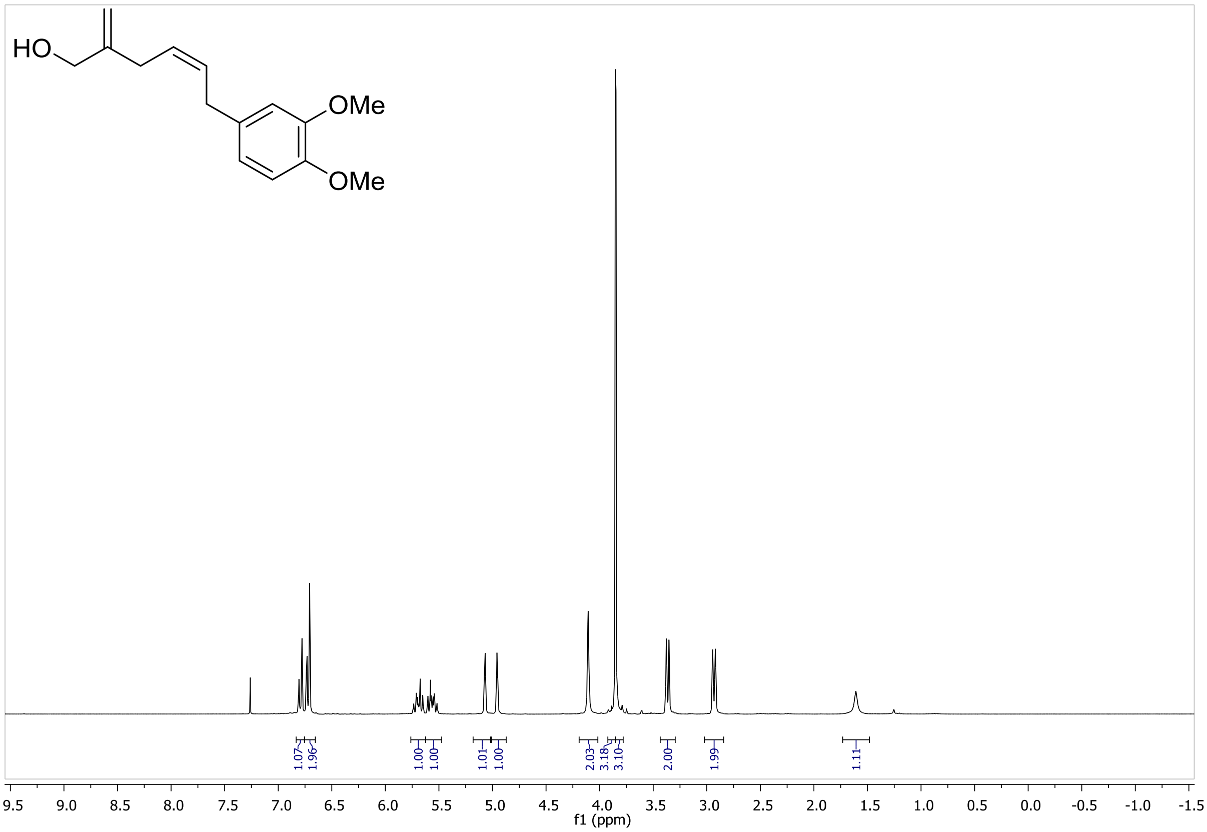

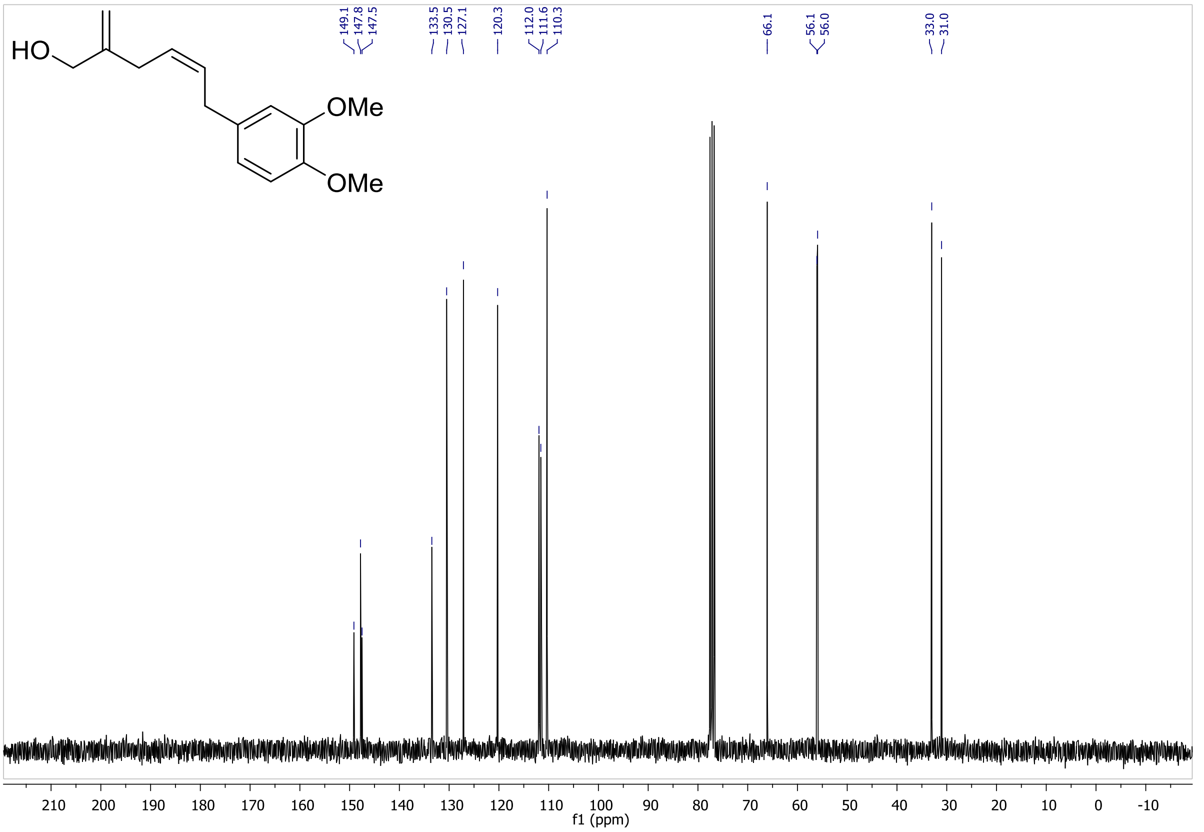

(*Z*)-6-(2-Bromo-4,5-dimethoxyphenyl)-2-methylenehex-4-en-1-ol (2i)

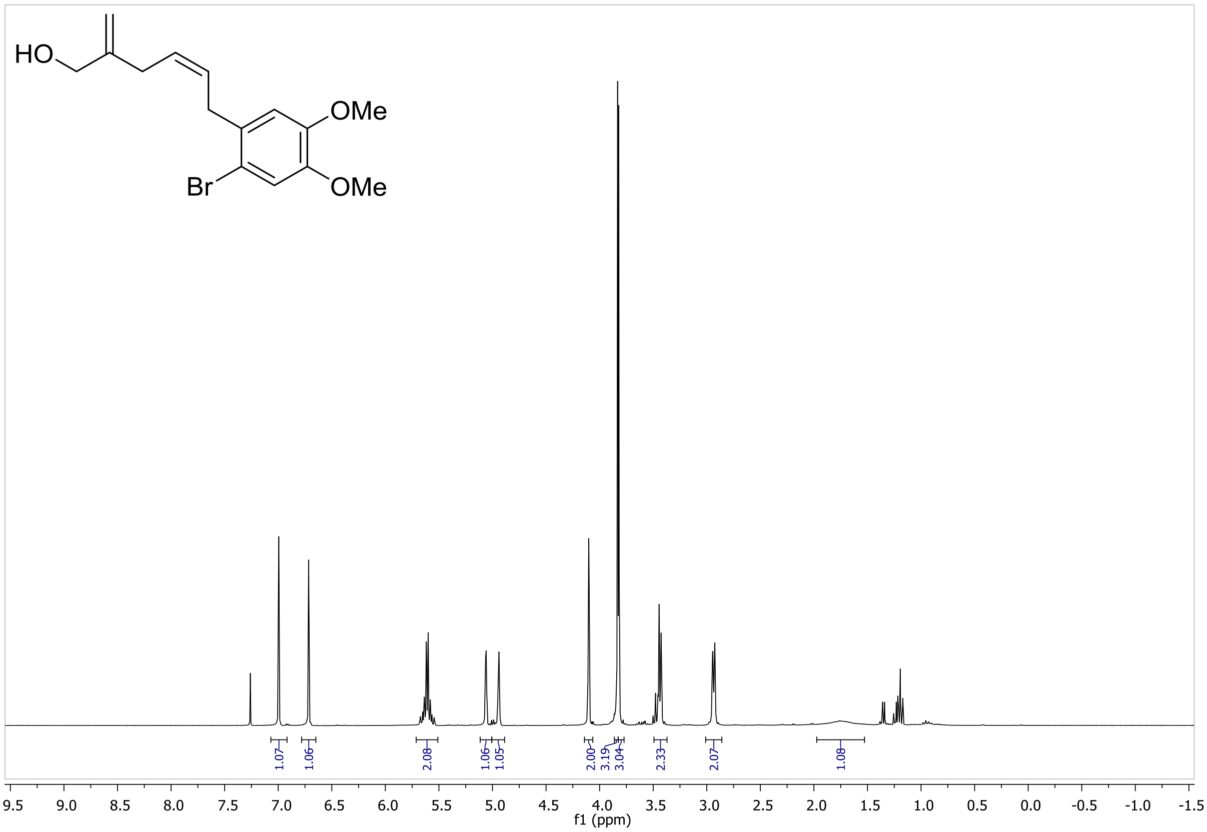

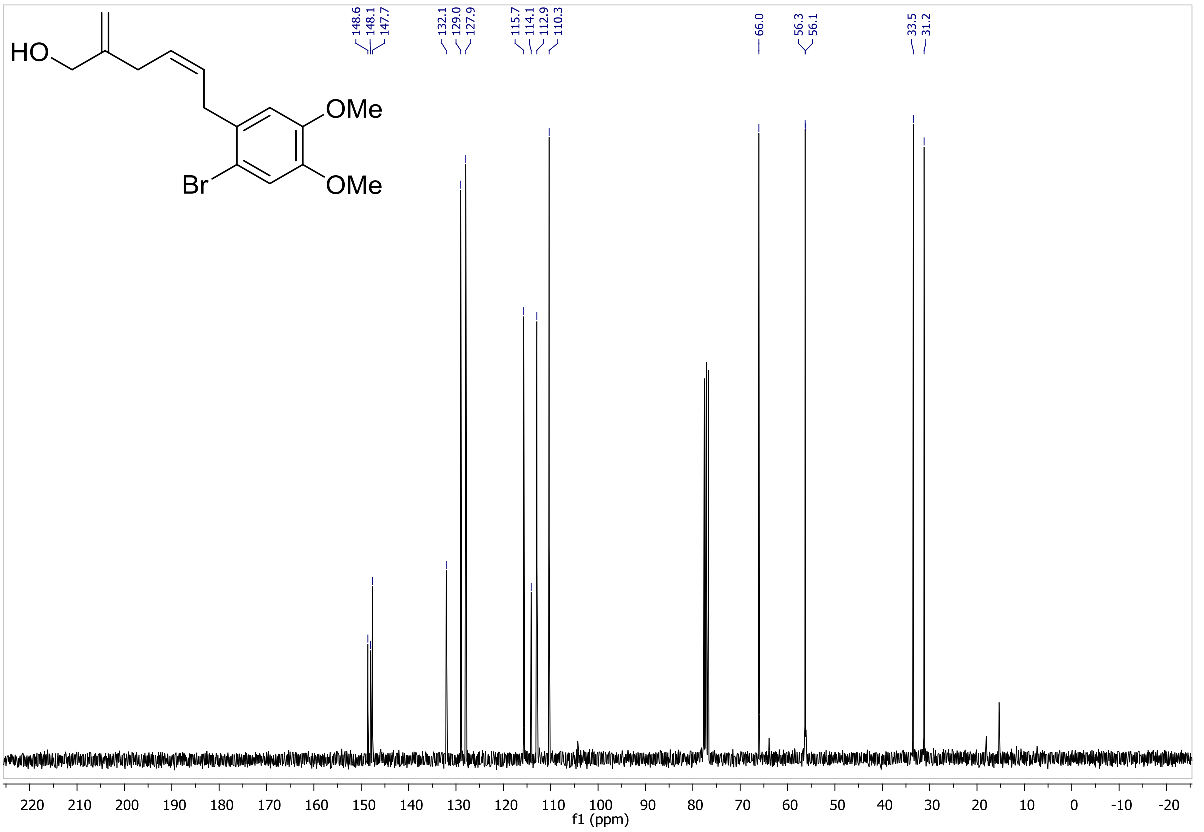

(*Z*)-6-(Furan-2-yl)-2-methylenehex-4-en-1-ol (2j)

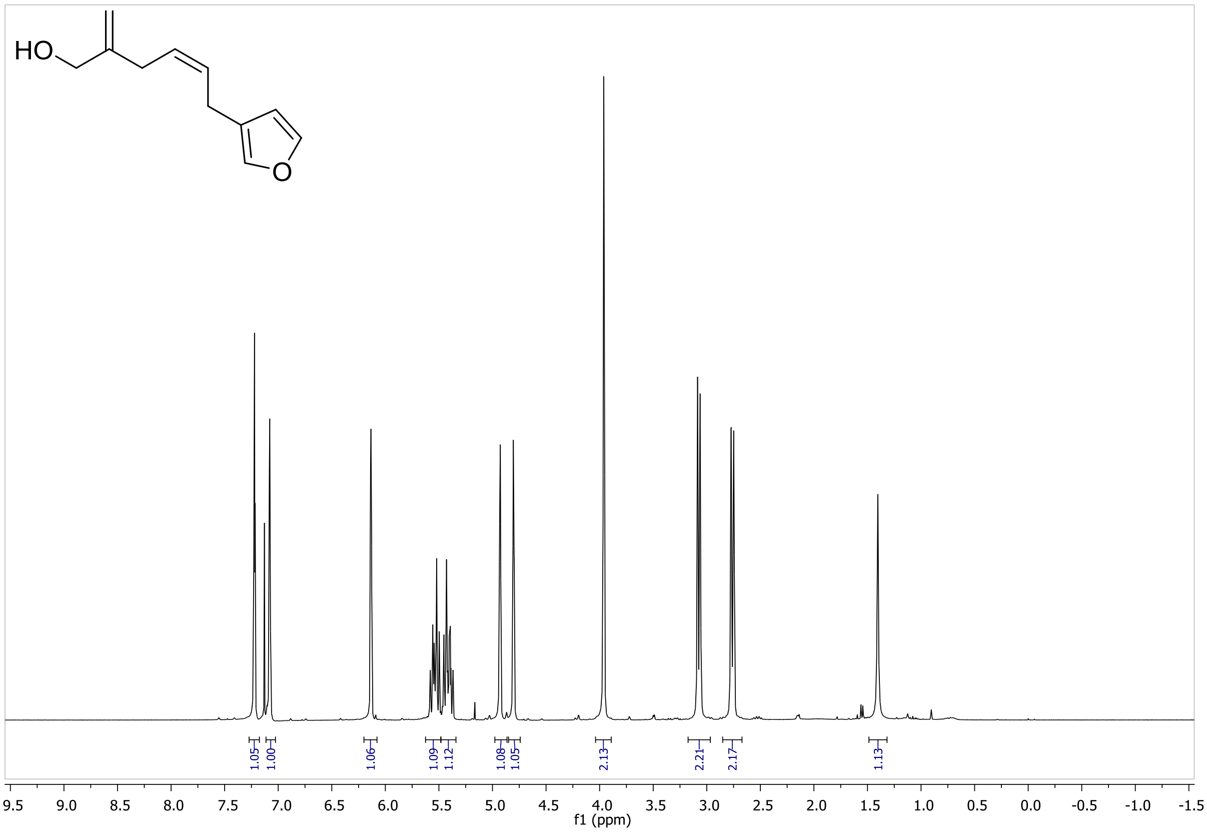

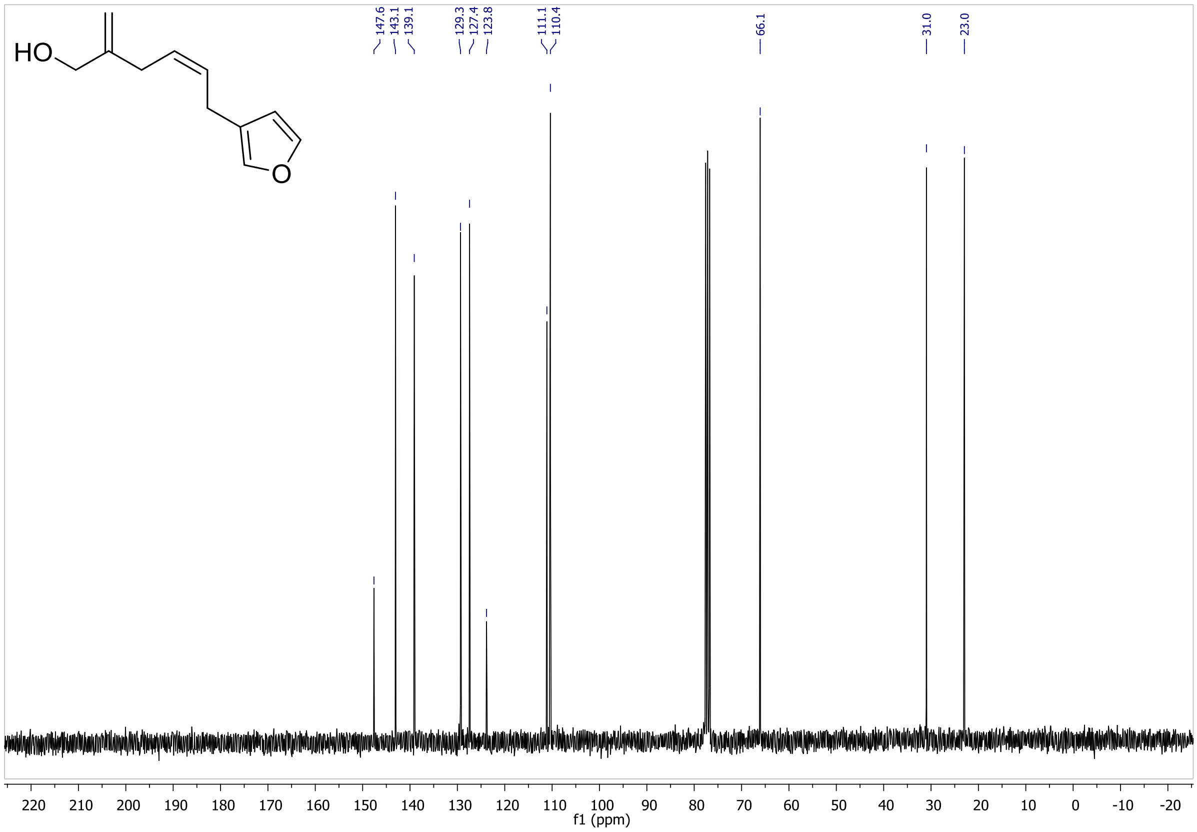

(*Z*)-2-Methylene-6-(thiophen-2-yl)hex-4-en-1-ol (2k)

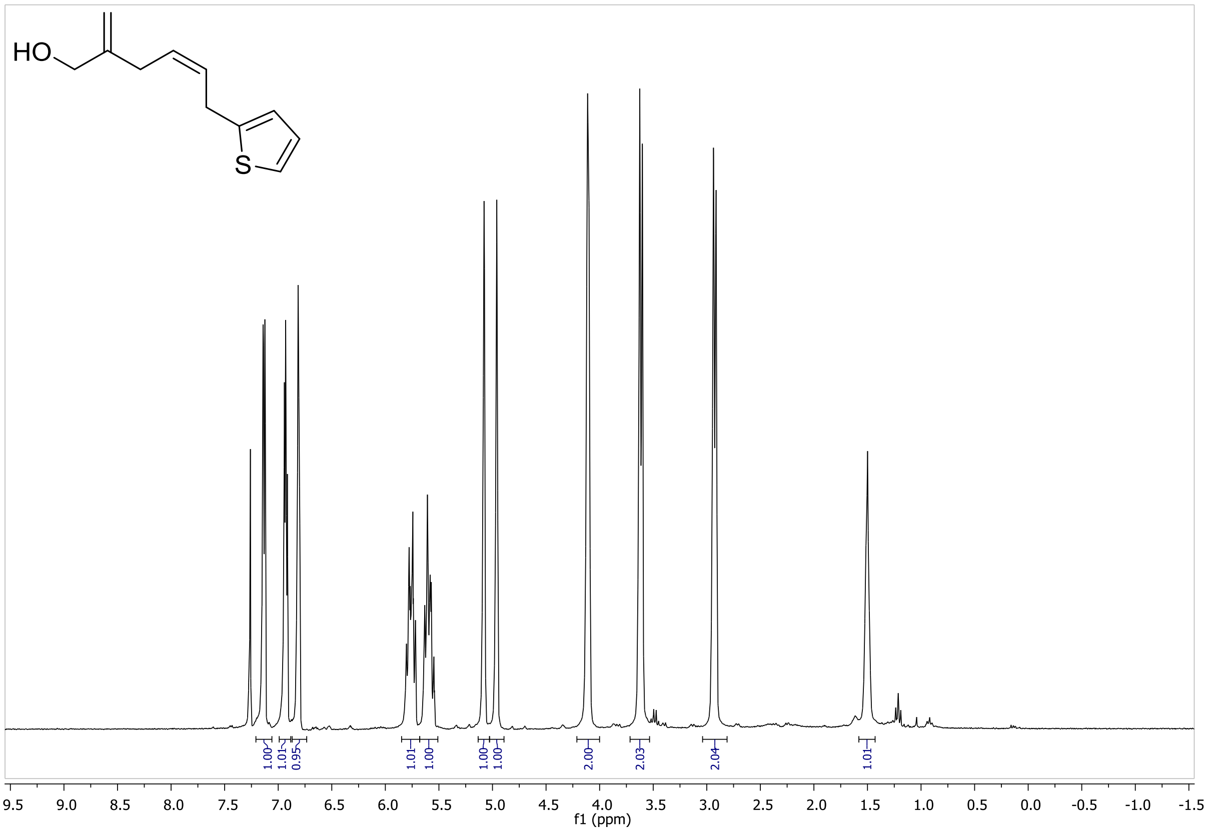

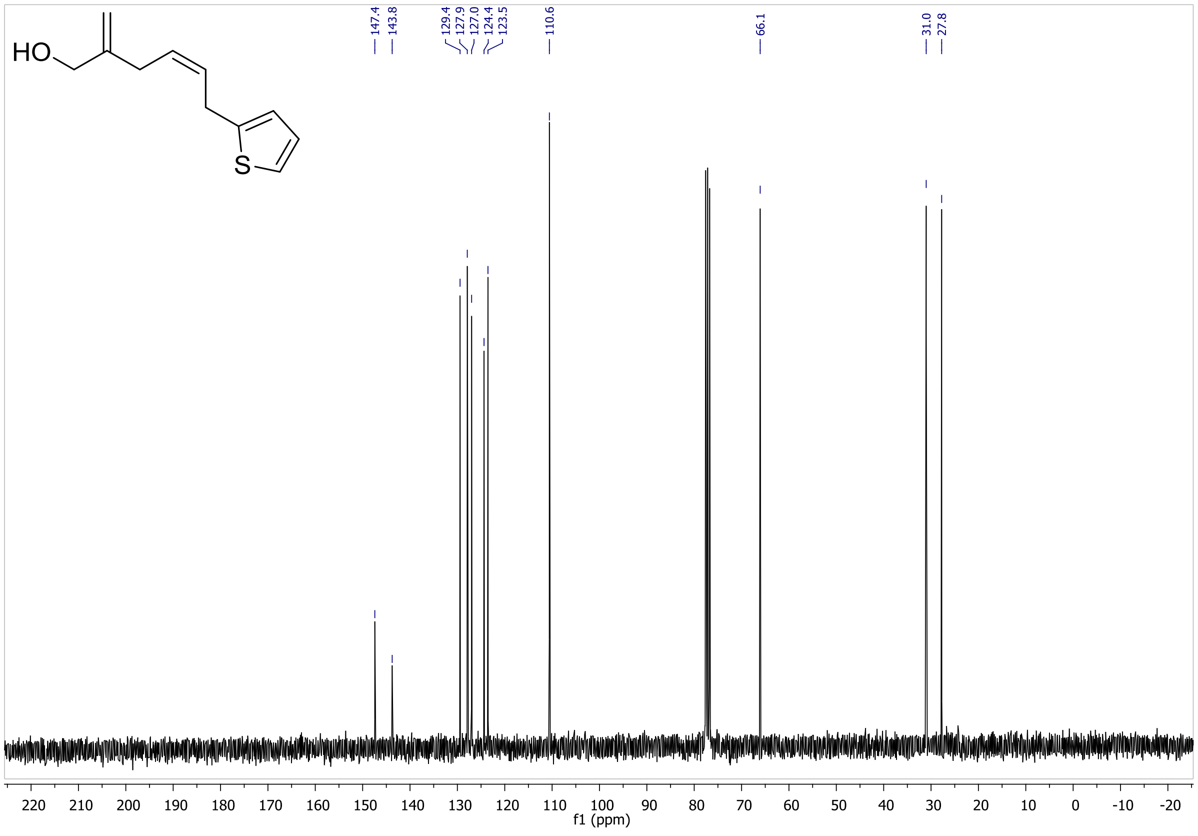

(*Z*)-2-Methylenehex-4-en-1-ol (2l)

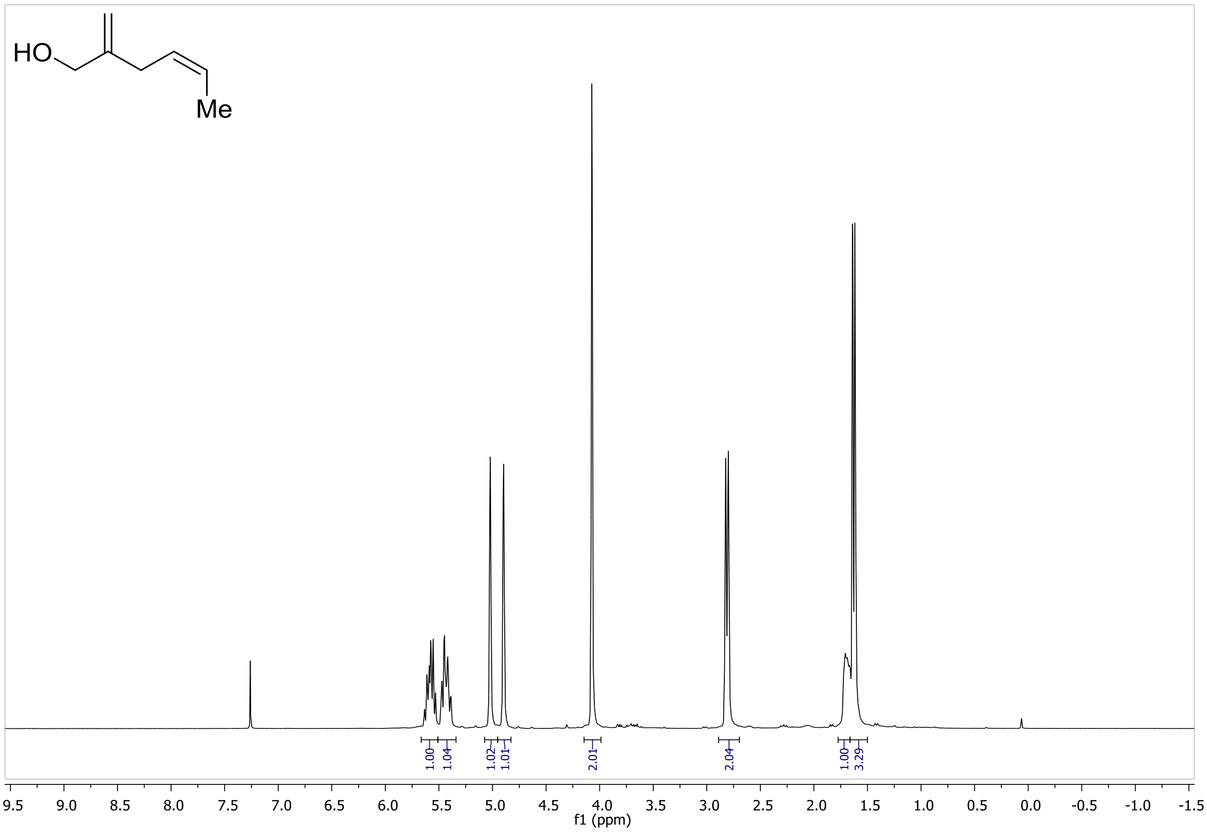

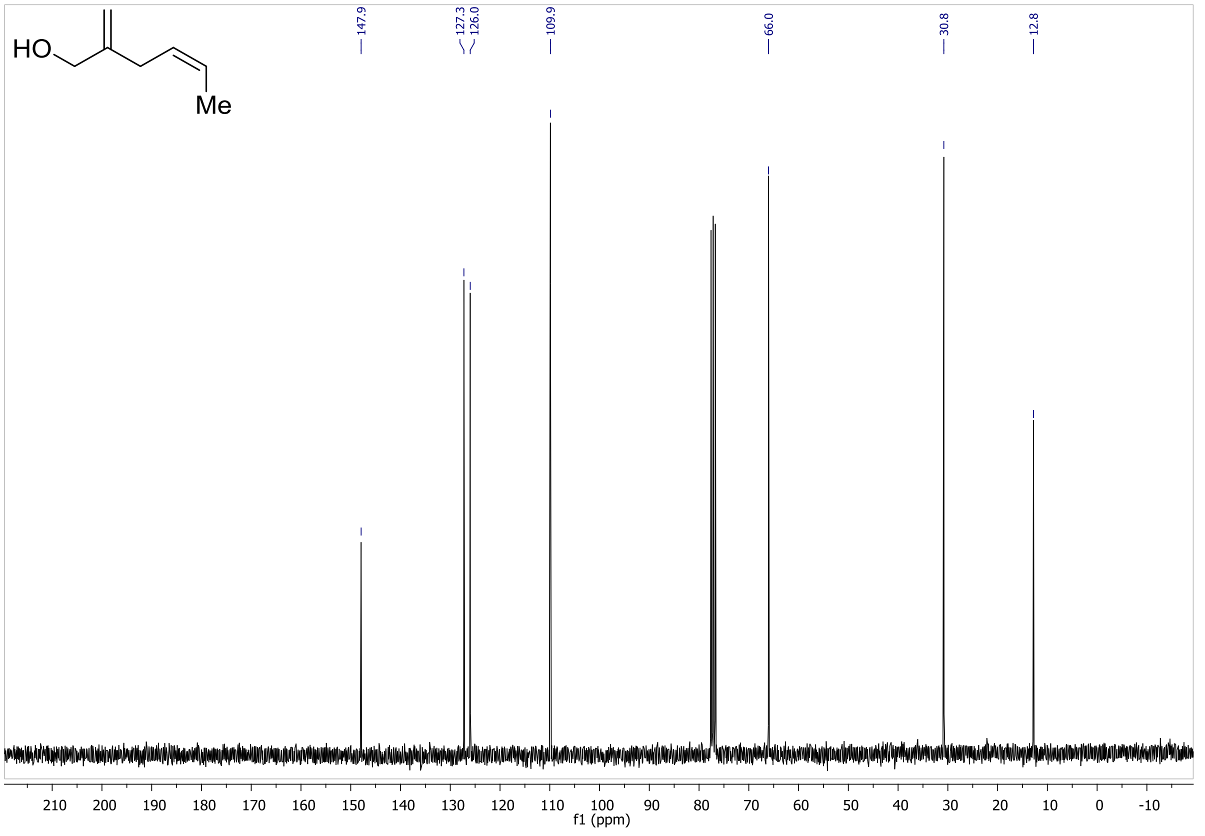

4,5-Dimethyl-2-methylenehex-4-en-1-ol (2m)

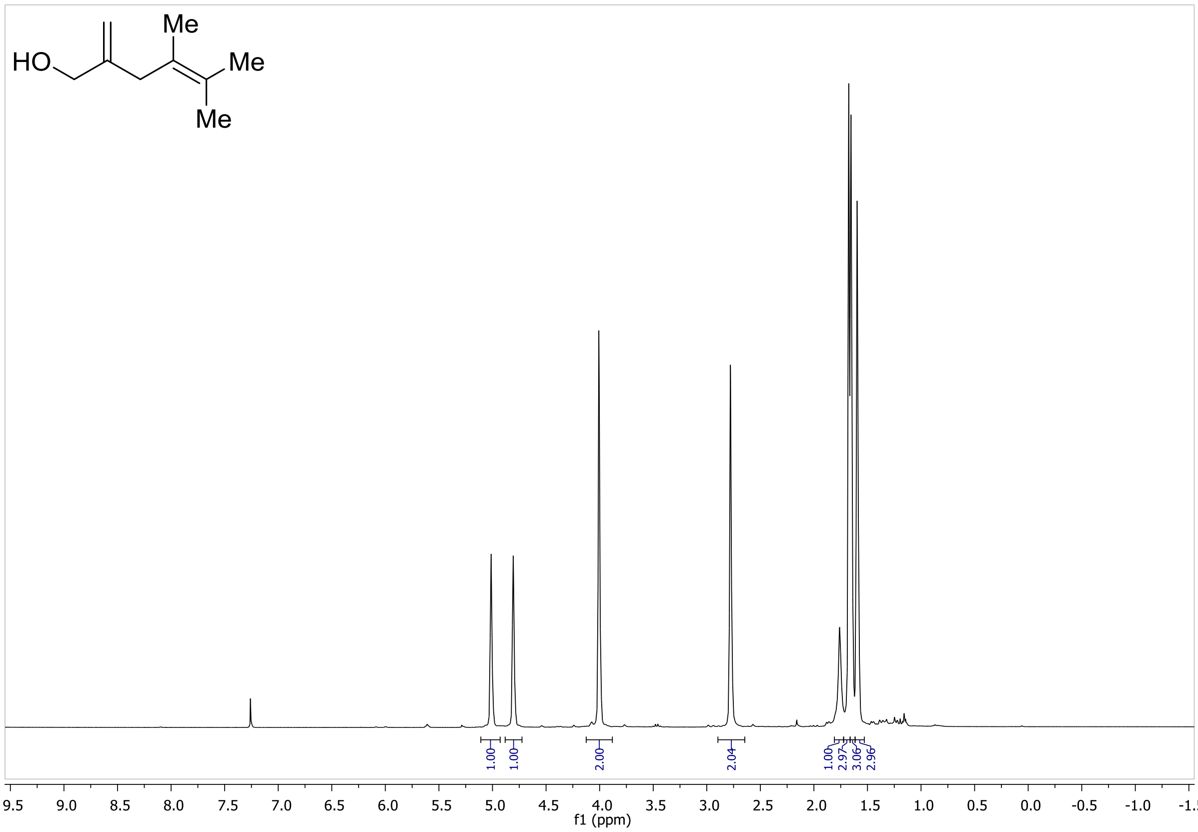

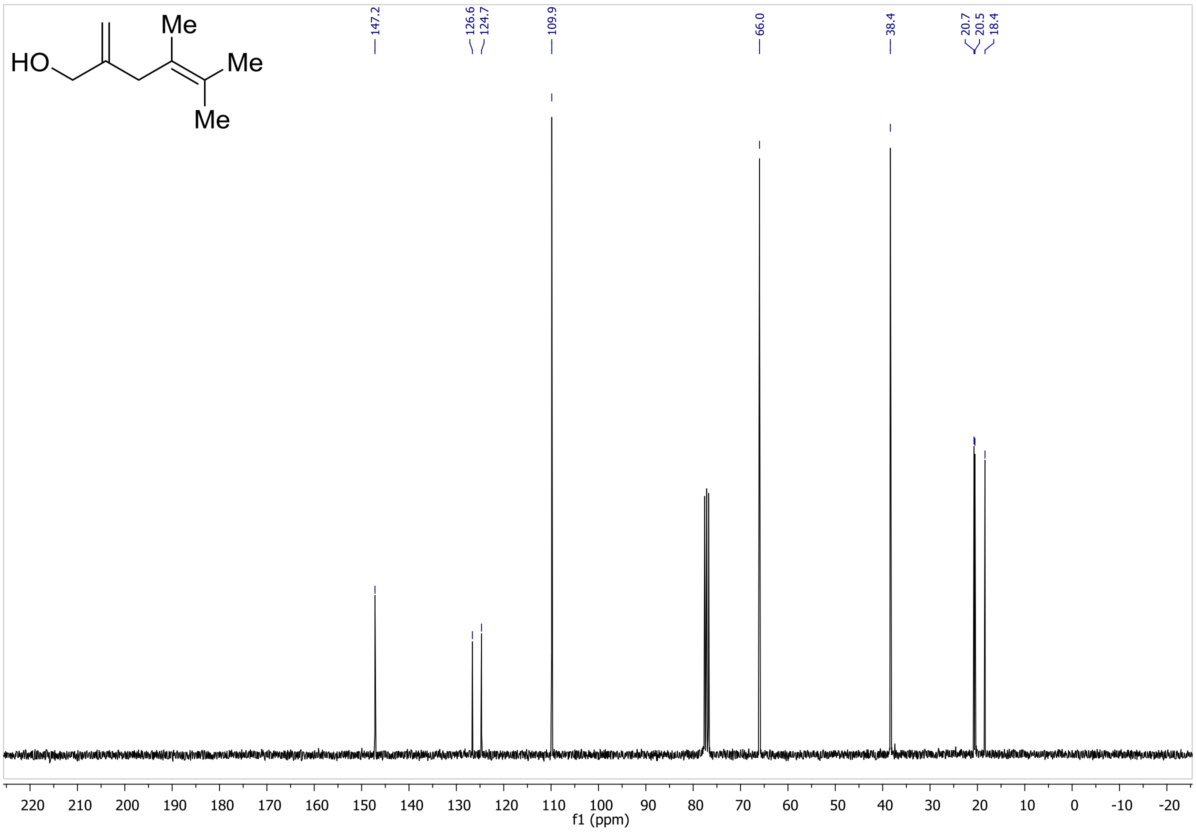

5-Methyl-2-methylenehex-4-en-1-ol (2n) and (*Z*) -4-methyl-2-methylenehex-4-en-1-ol (2o)

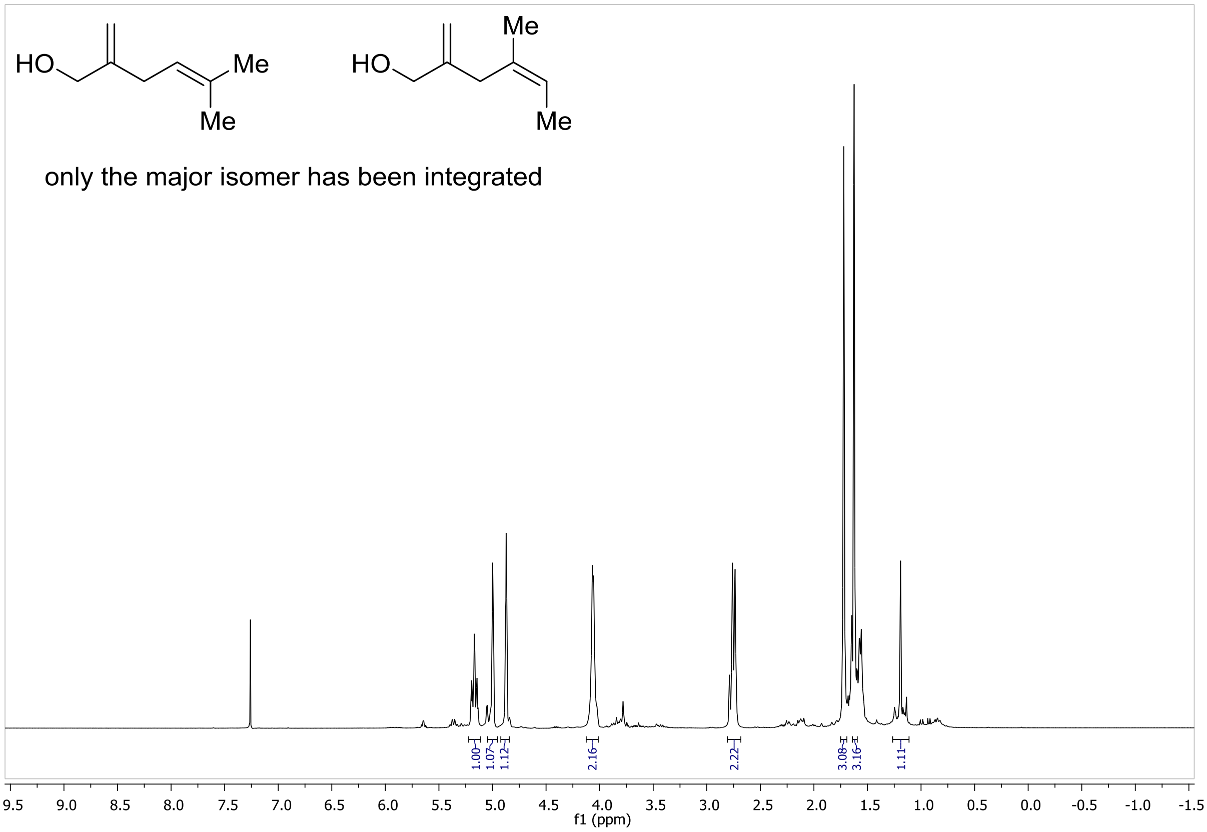

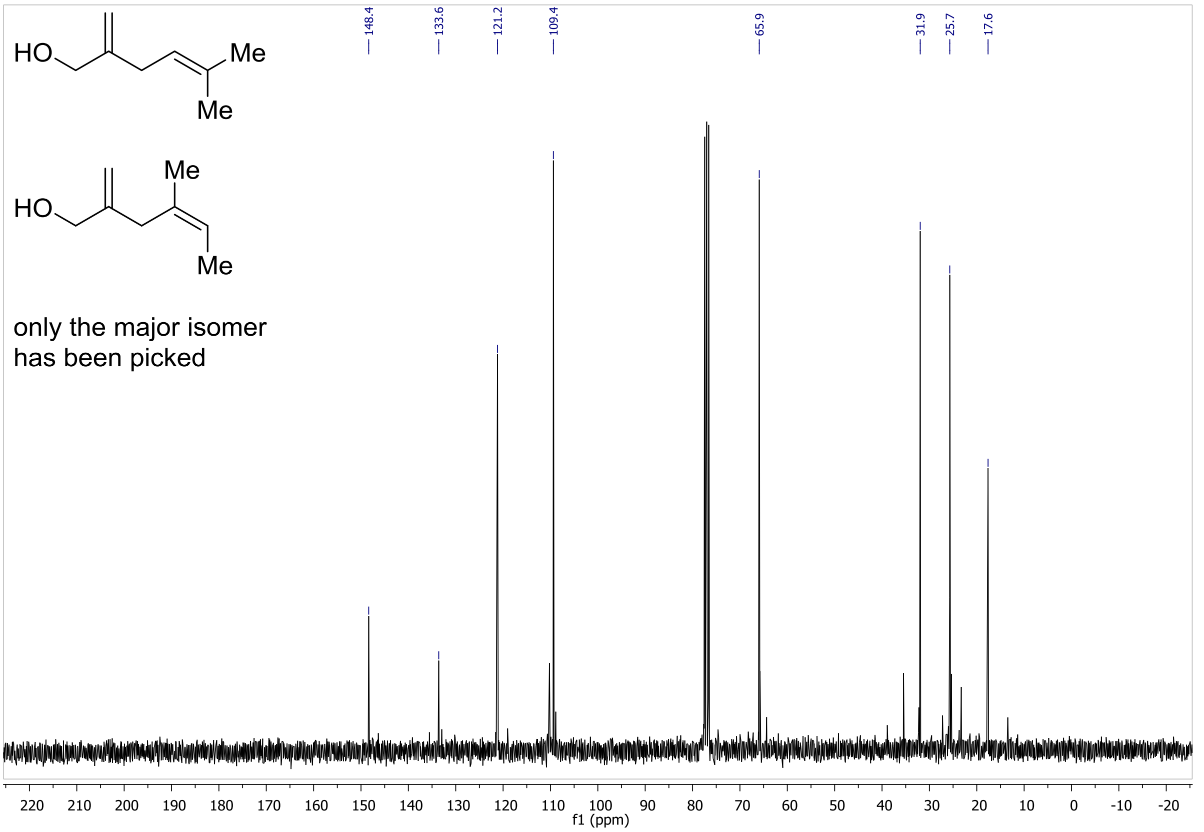

***tert*-Butyl(2,3-dimethyl-5-methylenetridec-2-en-6-yloxy)dimethylsilane**

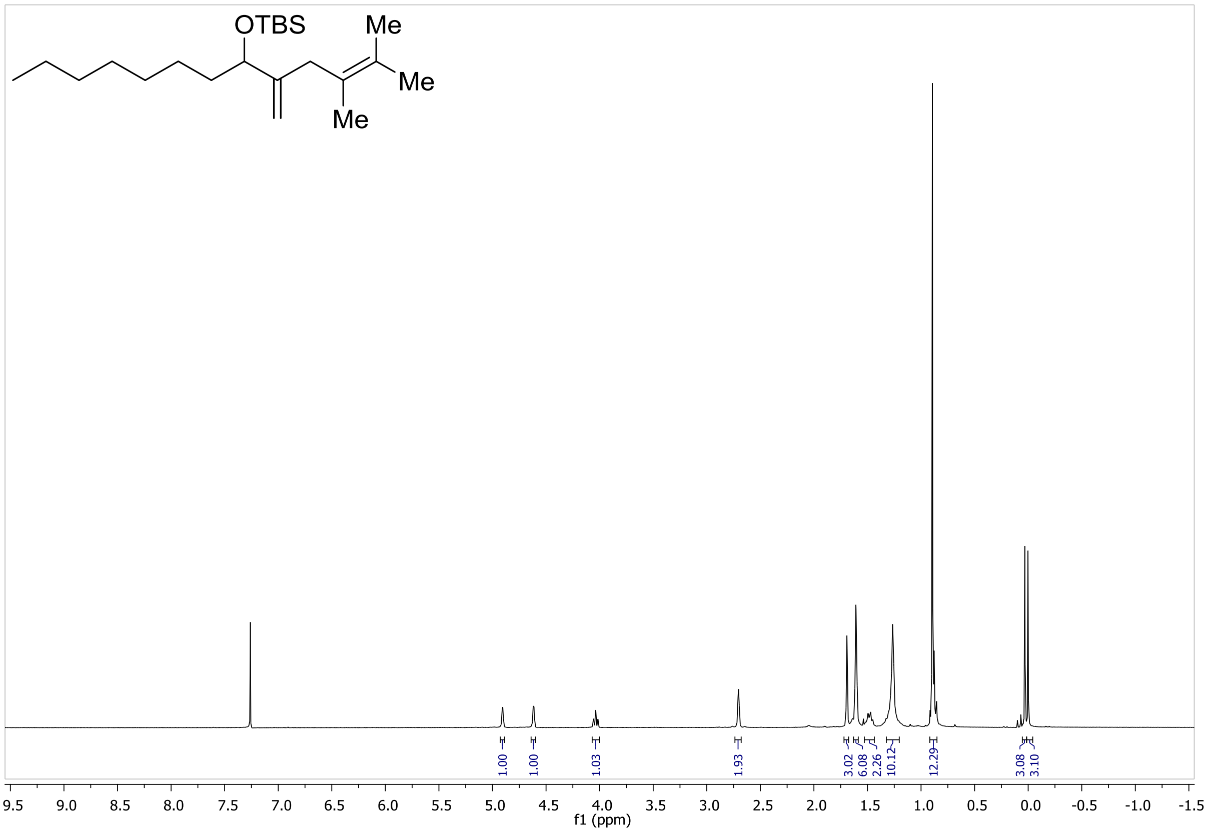

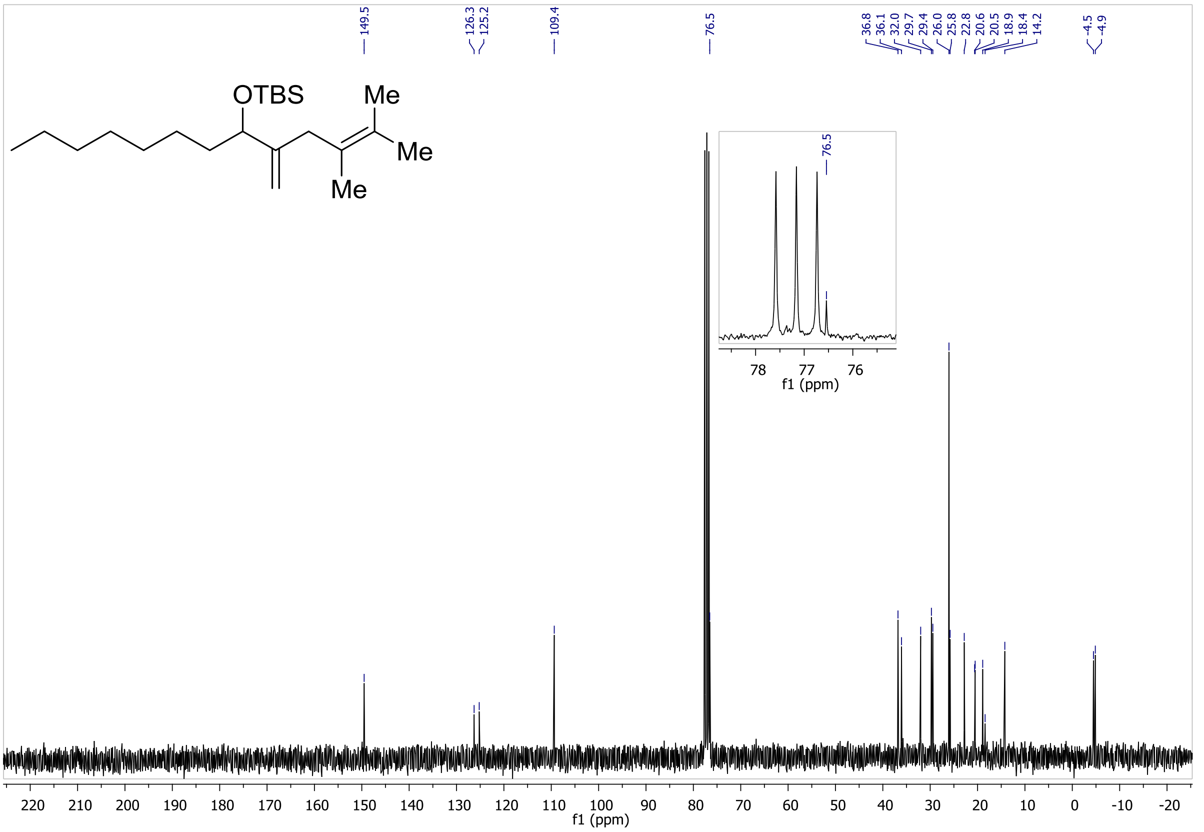

2,3-Dimethyl-5-methylenetridec-2-en-6-ol (2p)

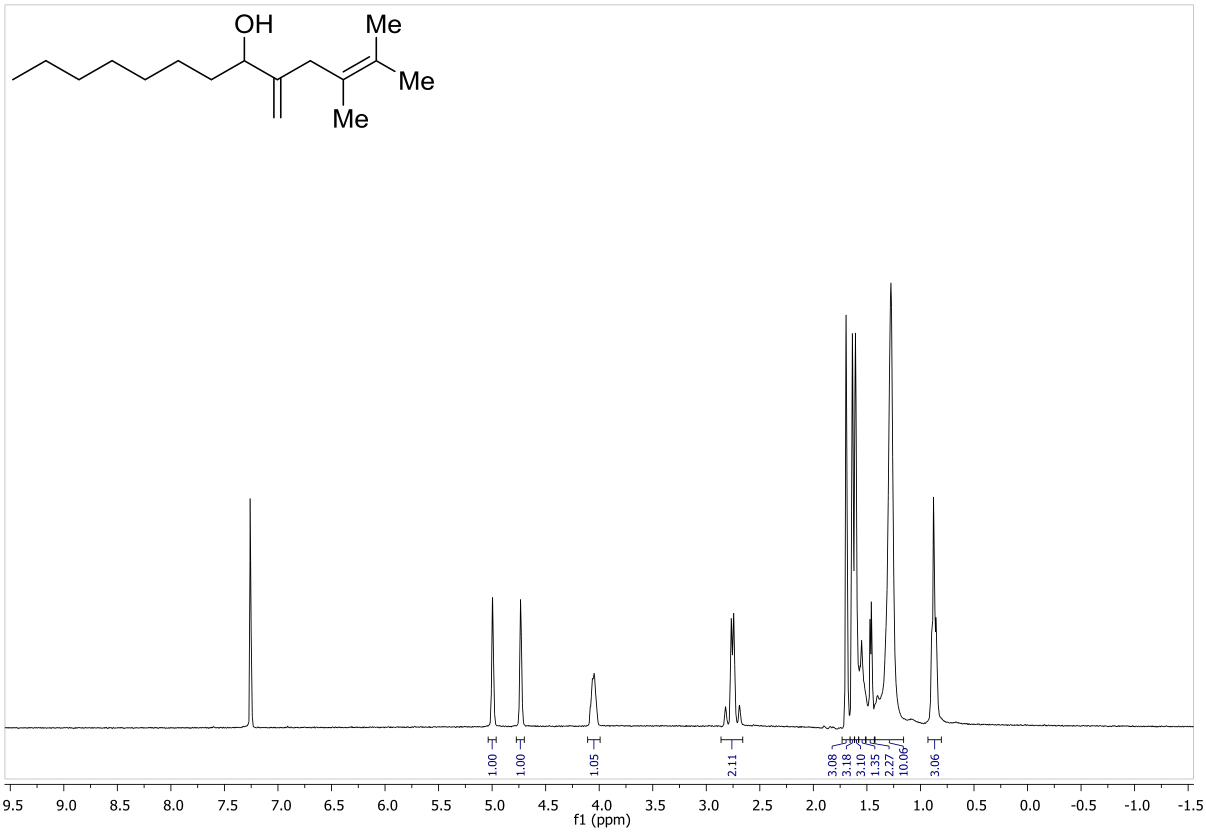

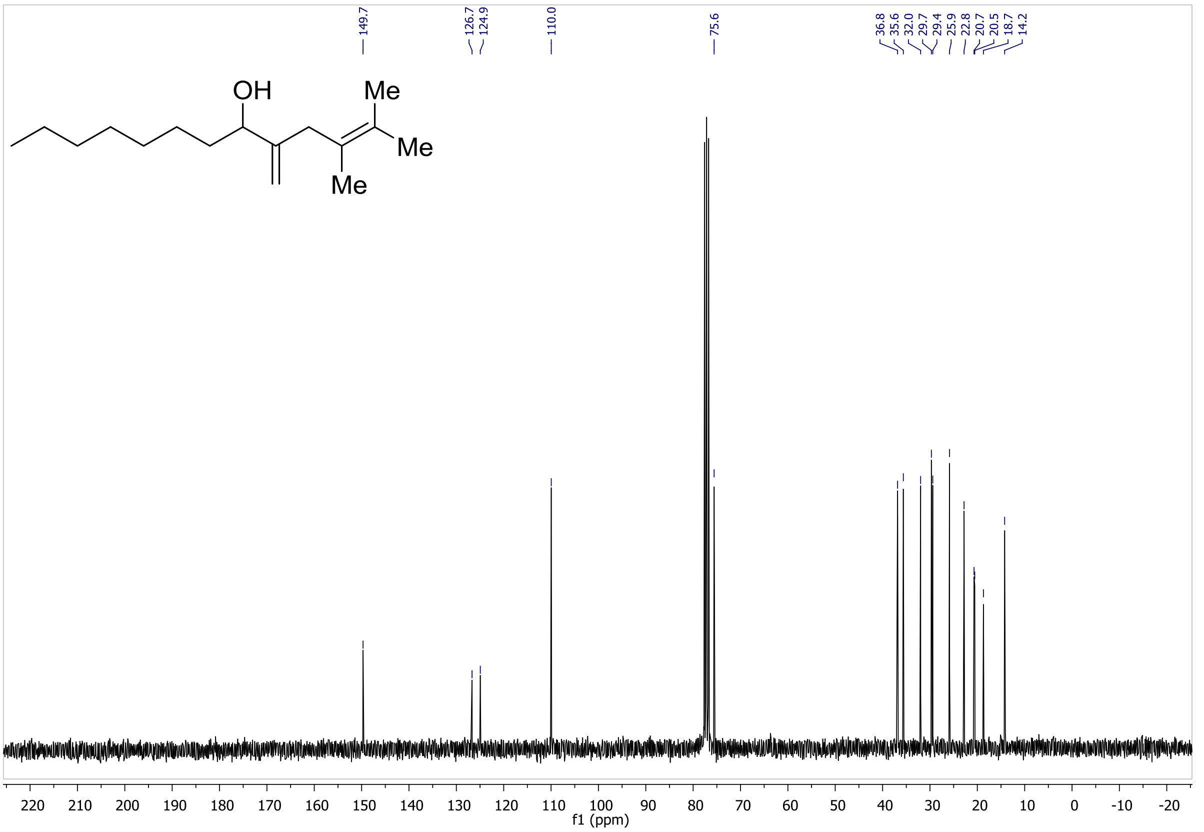

(1-(4-Bromophenyl)-4,5-dimethyl-2-methylenehex-4-enyloxy)(*tert*-butyl)dimethylsilane

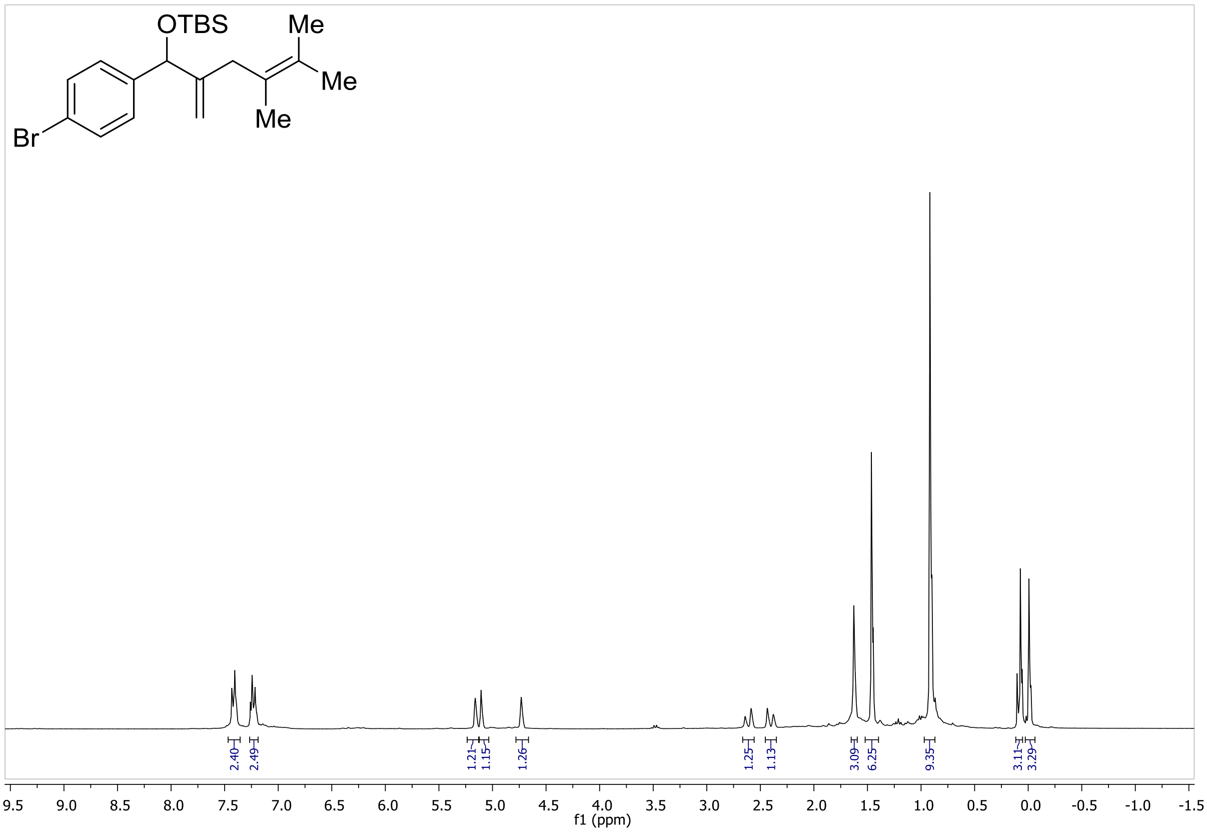

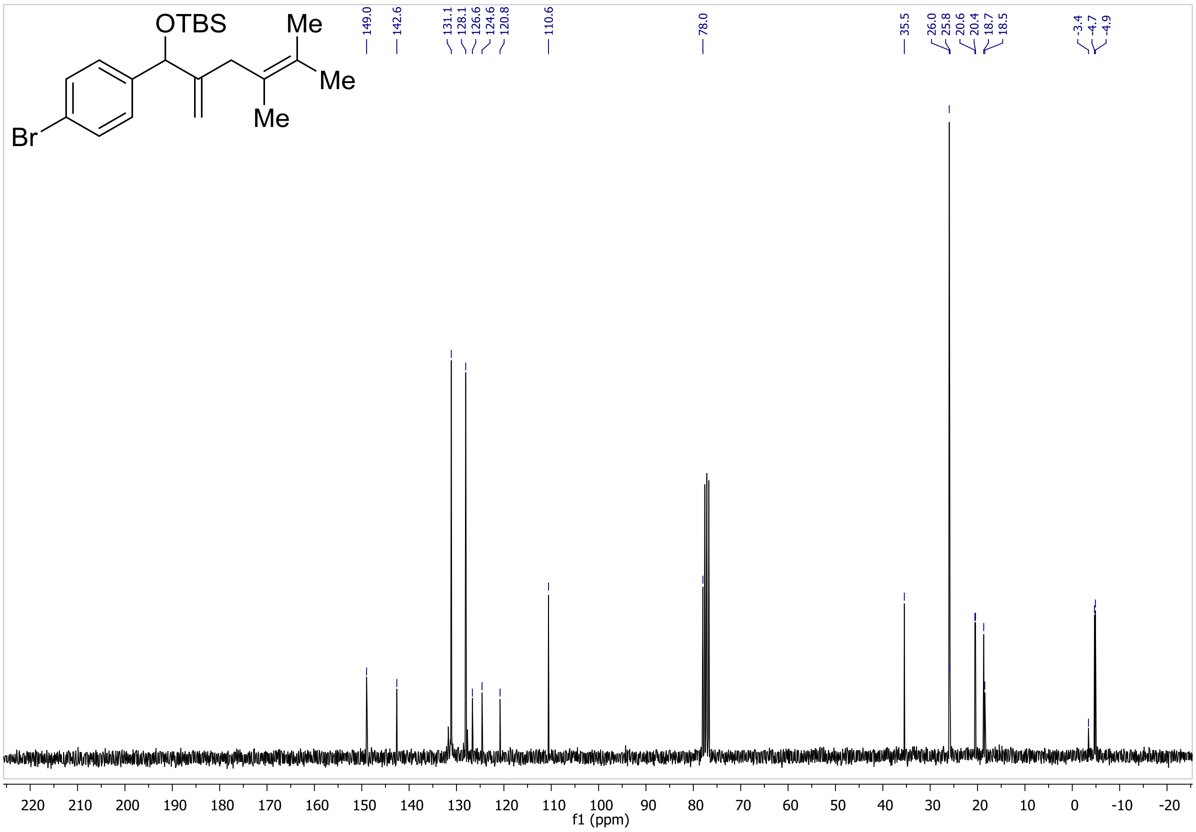

1-(4-Bromophenyl)-4,5-dimethyl-2-methylenehex-4-en-1-ol (2q)

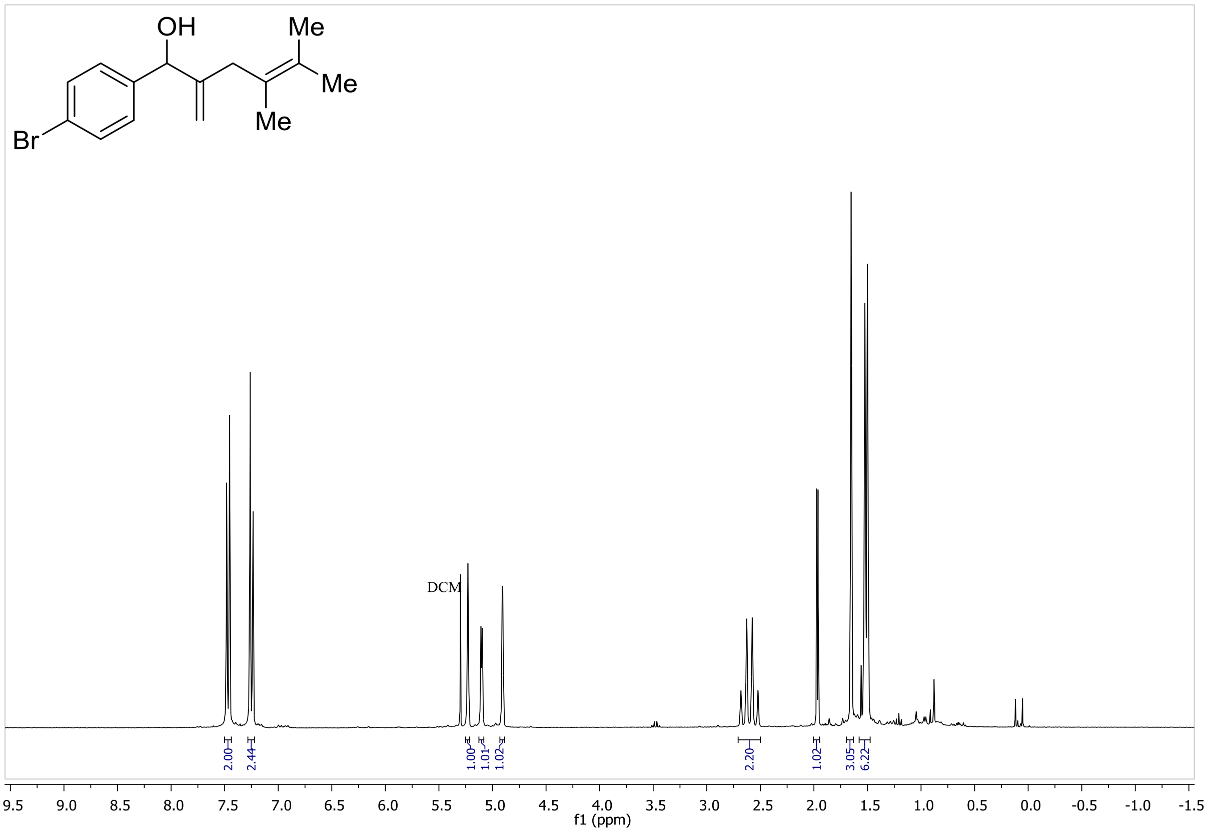

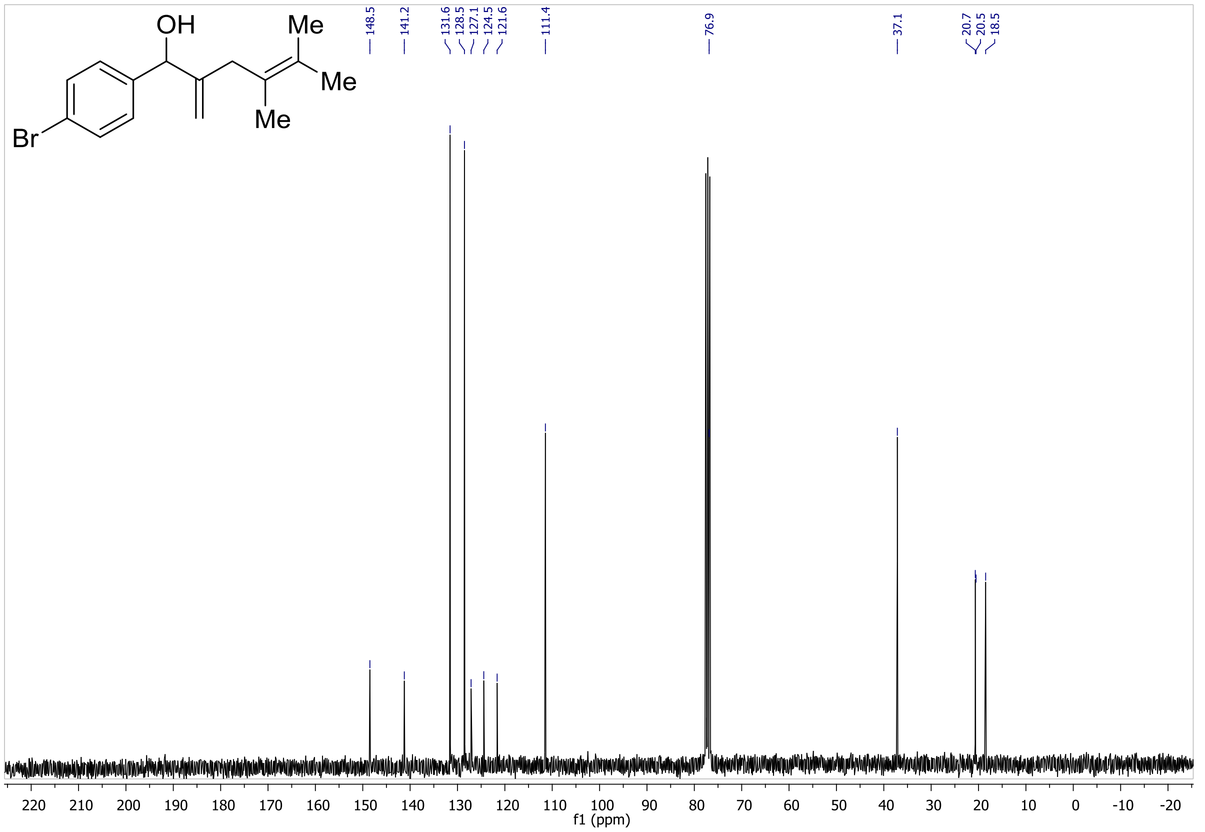

threo-4-Methylene-2-(2-phenyl-1-(phenylselanyl)ethyl)tetrahydrofuran (3a)

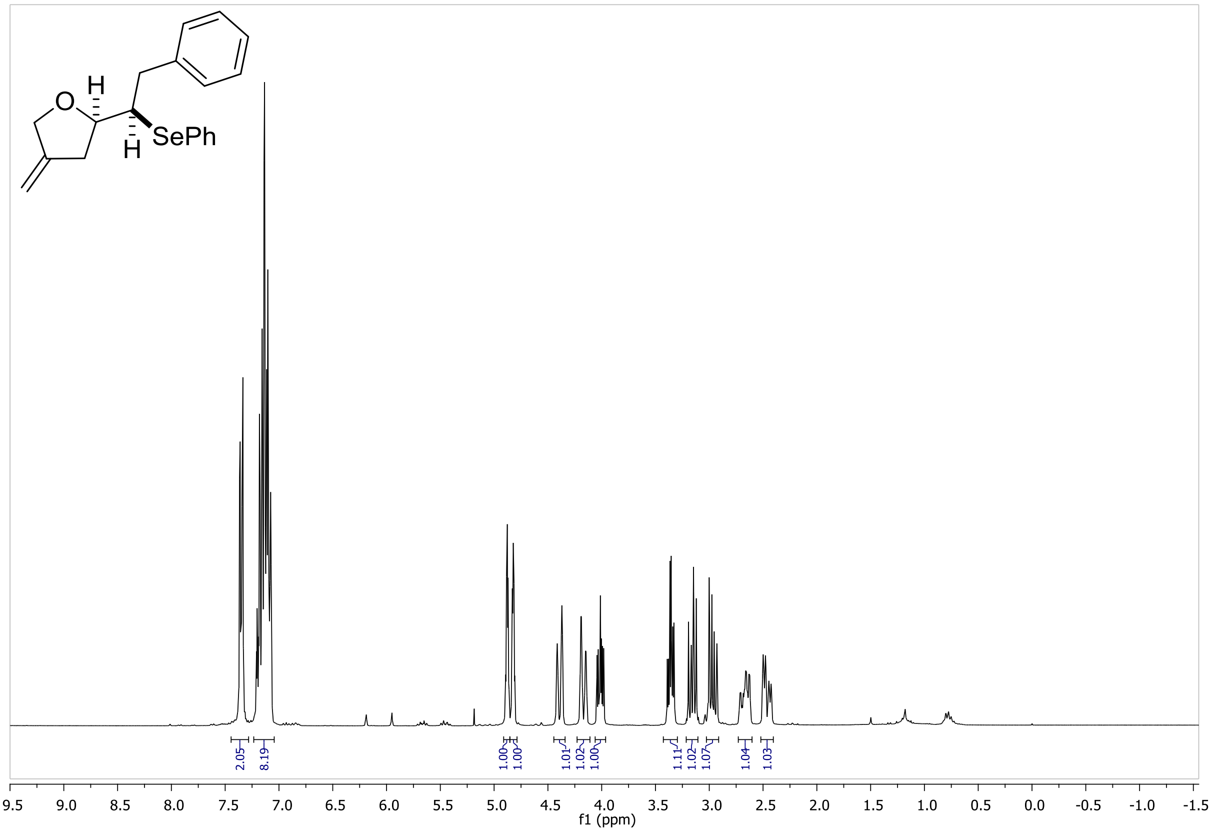

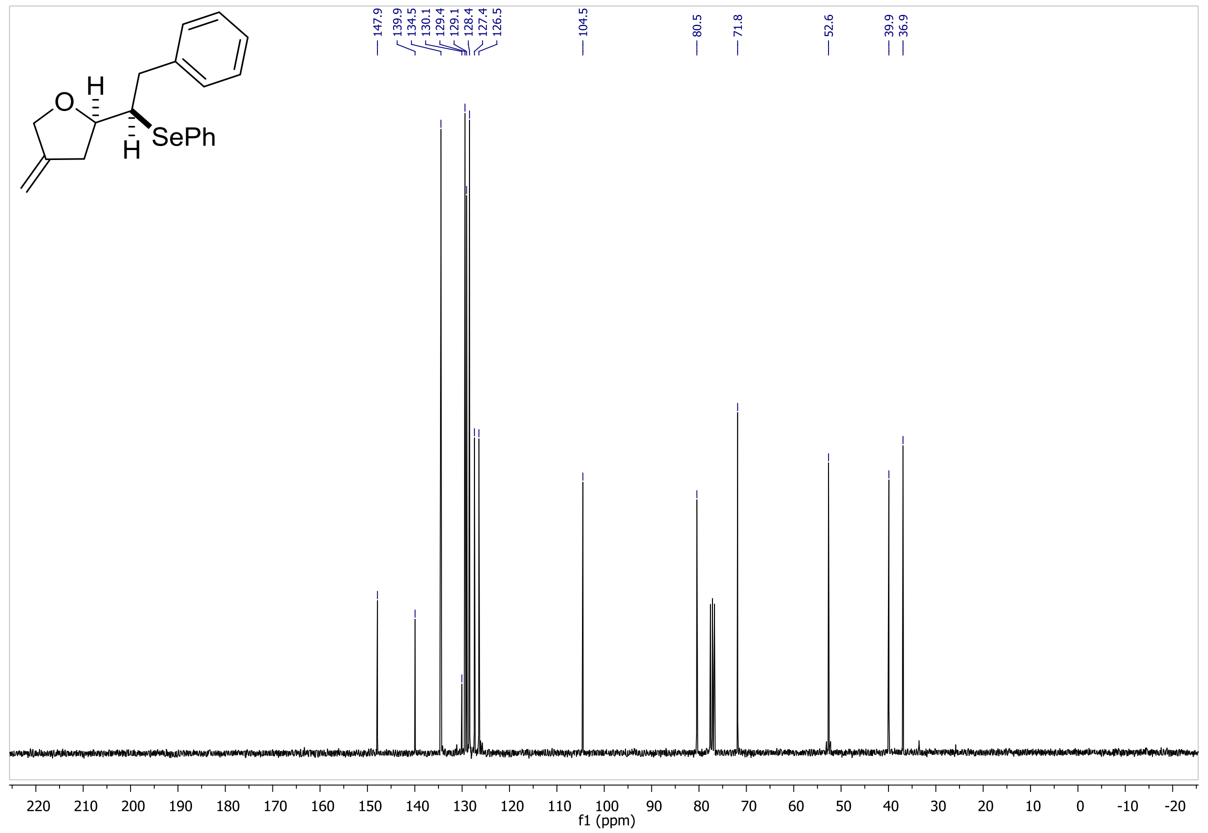

***threo*-2-(2-(4-Bromophenyl)-1-(phenylselanyl)ethyl)-4-methylenetetrahydrofuran (3b)**

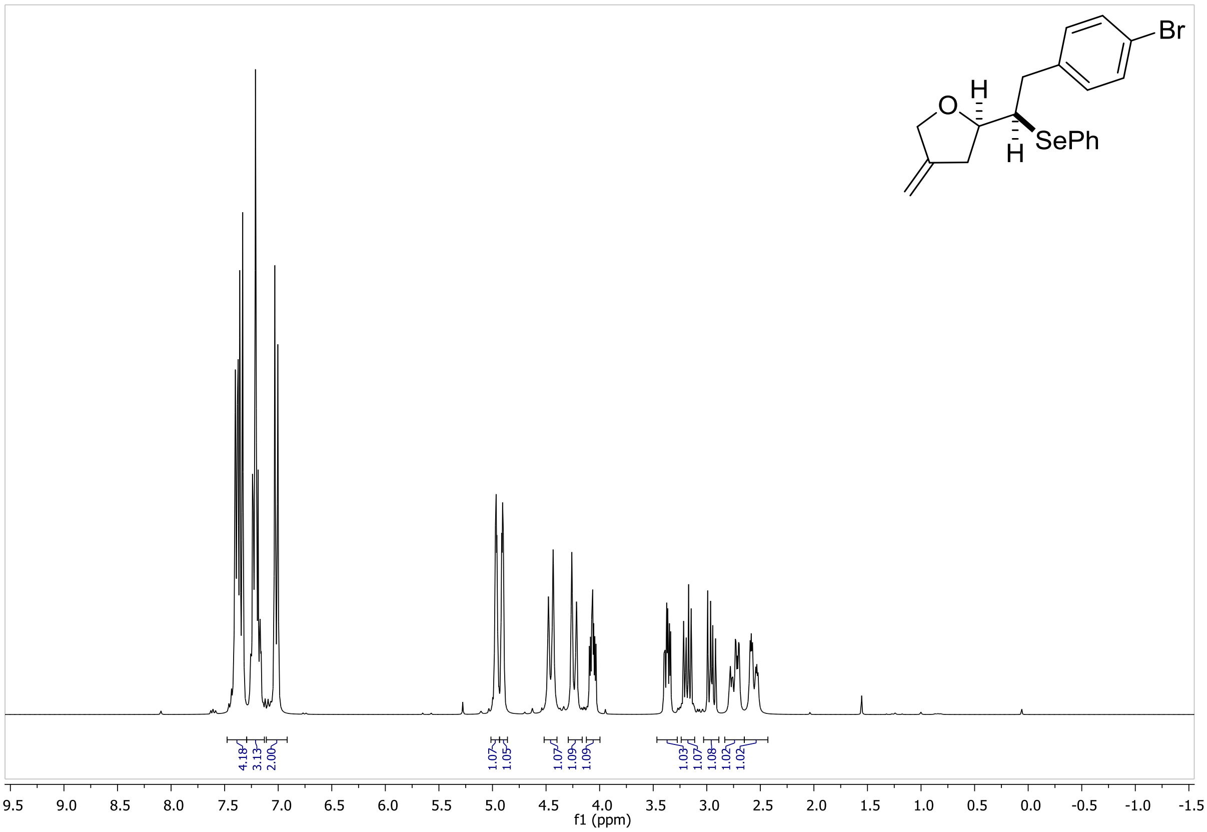

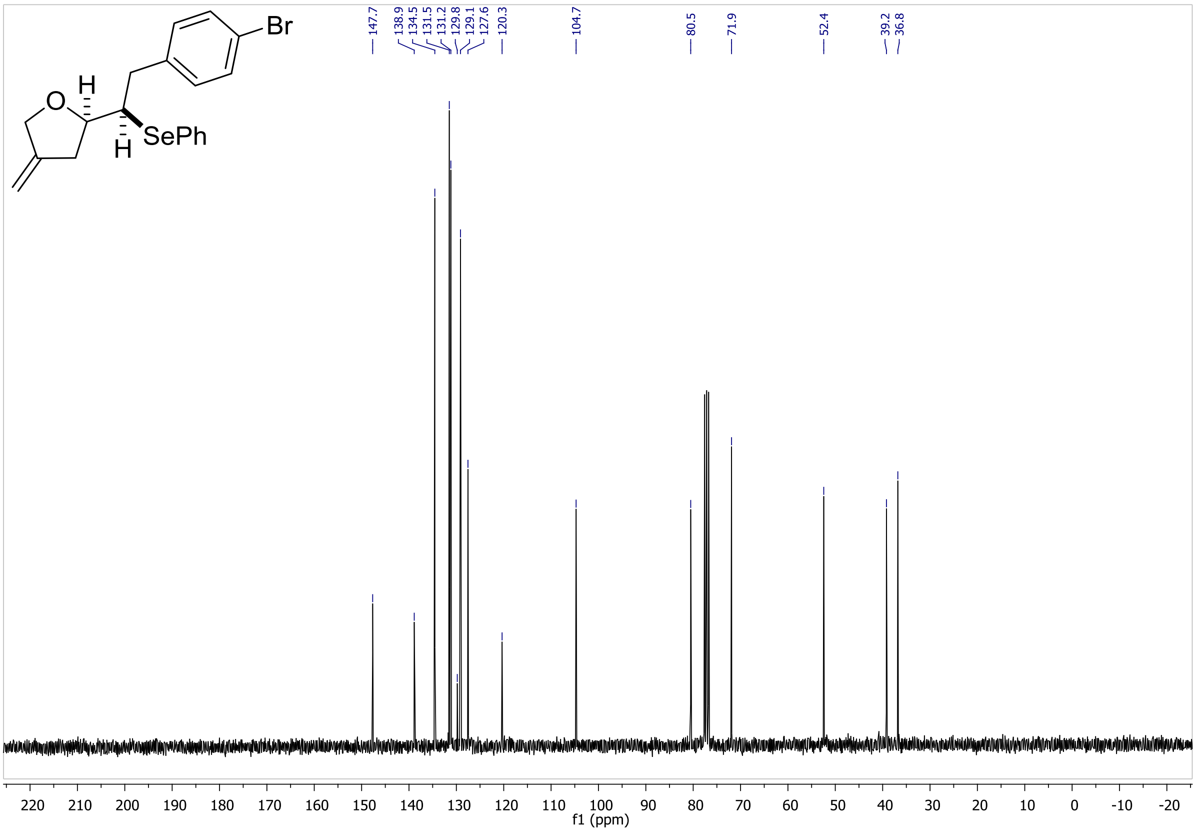

***threo*-Methyl-3-(2-(4-methylenetetrahydrofuran-2-yl)-2-(phenylselanyl)ethyl)benzoate (3d)**

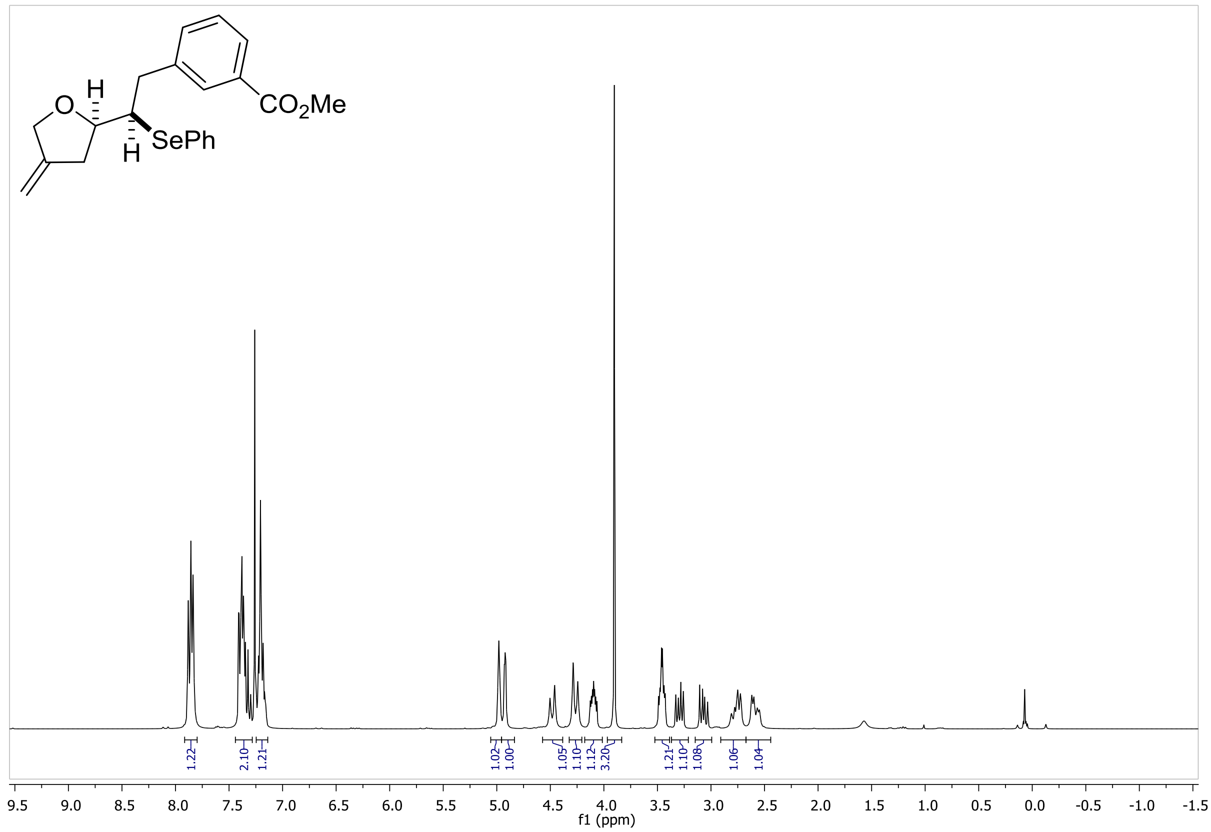

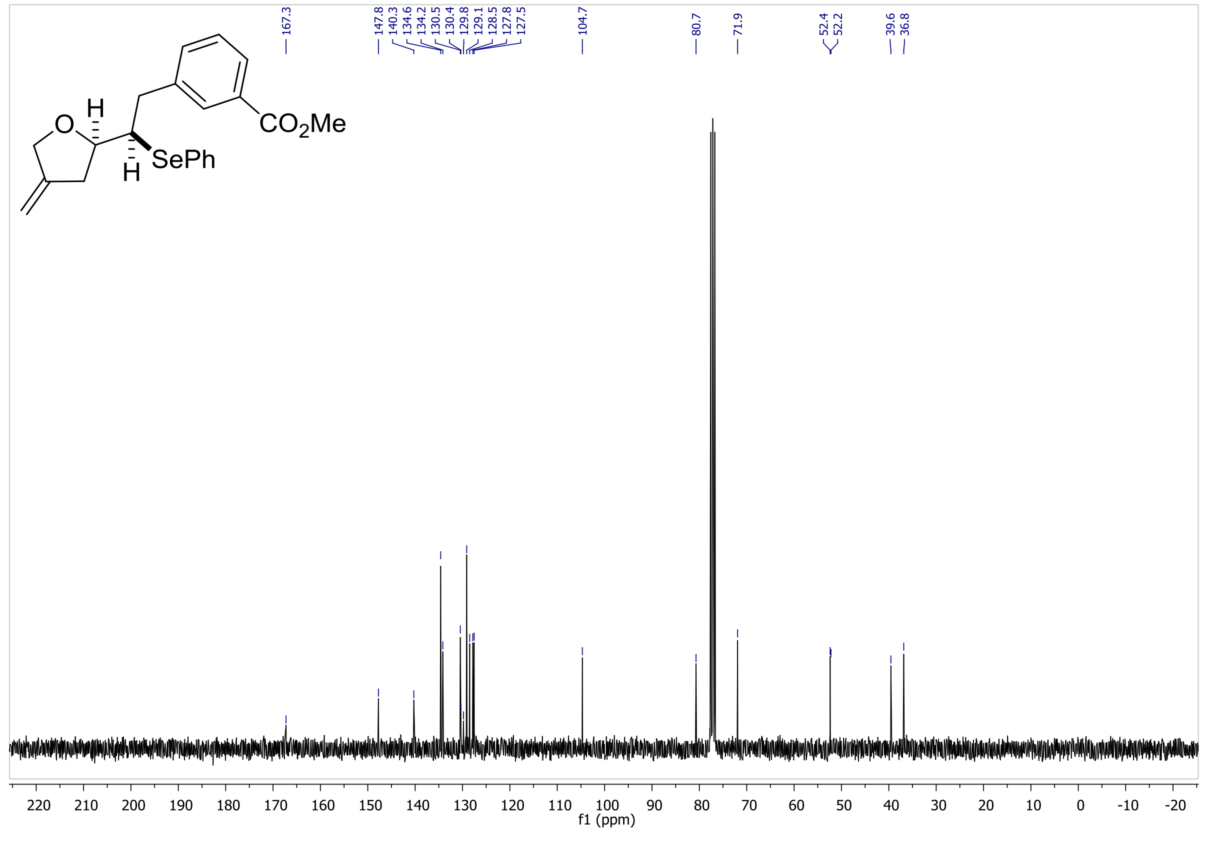

threo-2-(2-(4-Methoxyphenyl)-1-(phenylselanyl)ethyl)-4-methylenetetrahydrofuran (3f)

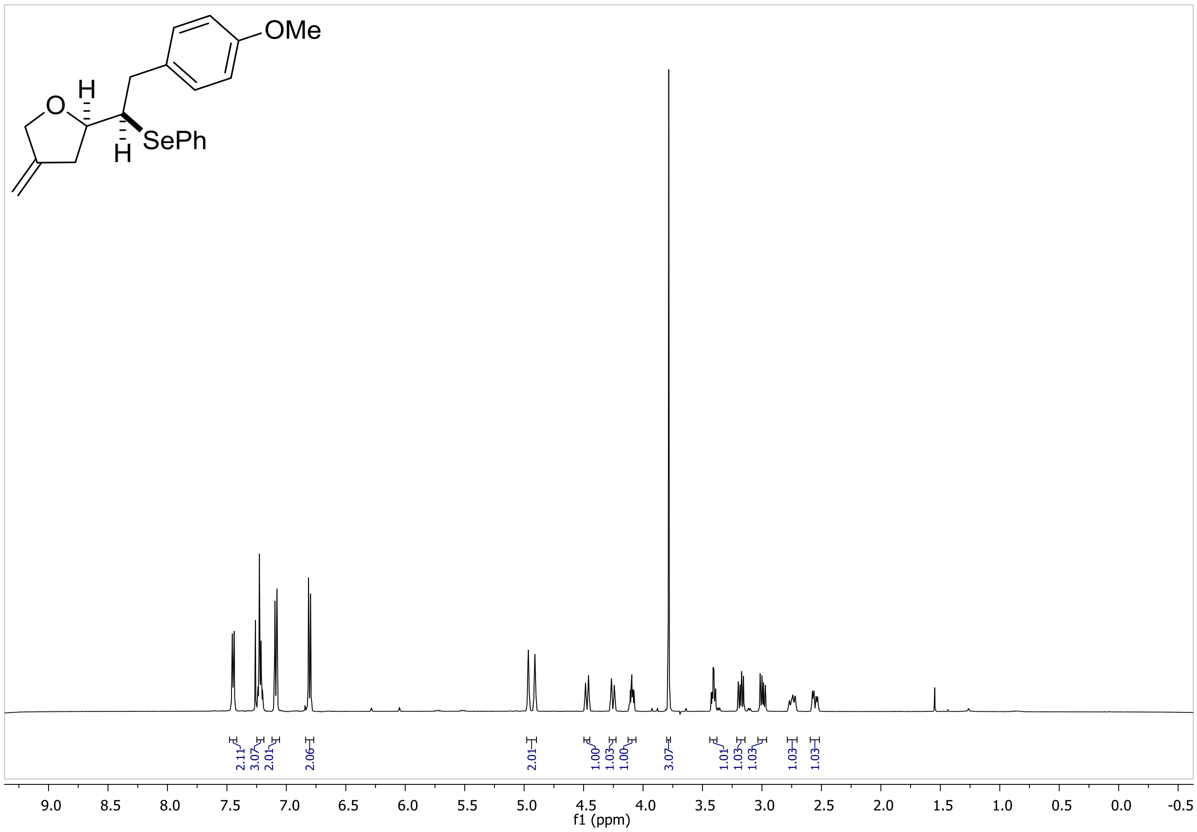

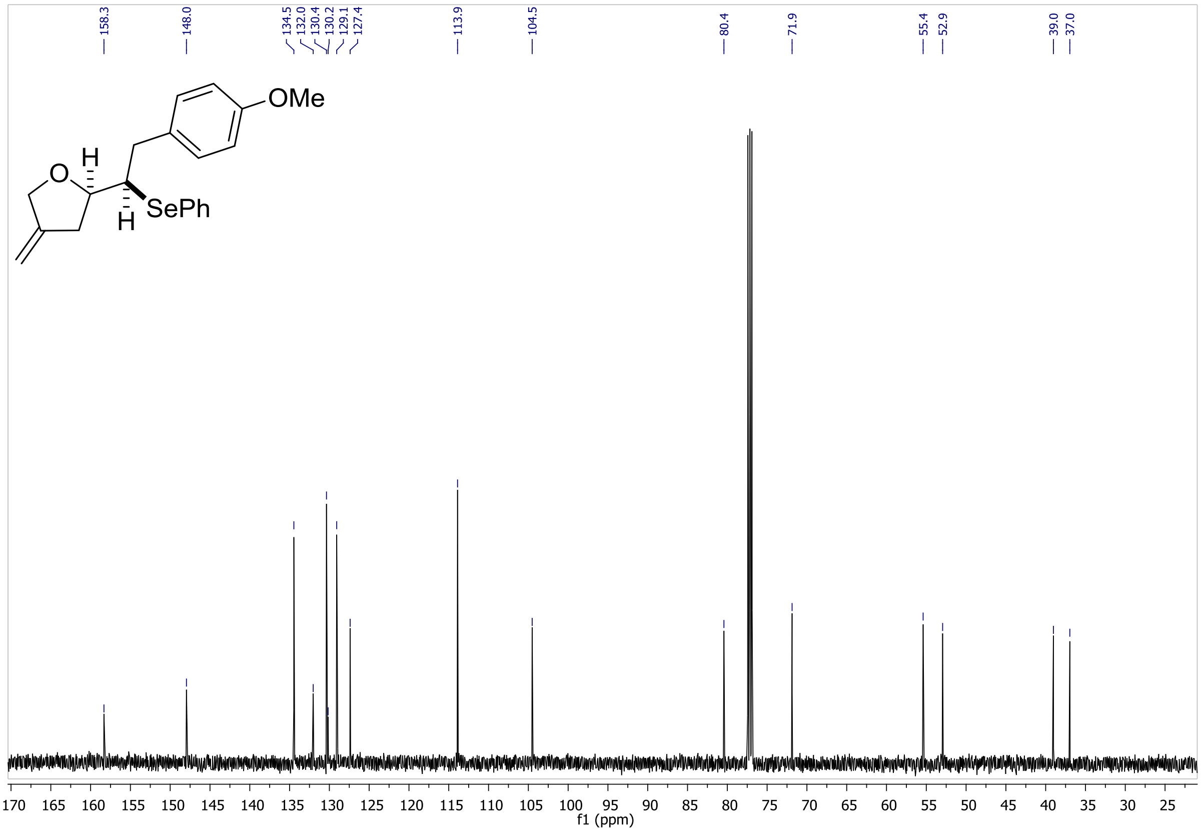

***threo*-4-Methylene-2-(1-(phenylselanyl)-2-(2,4,6-trimethoxyphenyl)ethyl)tetrahydrofuran (3g)**

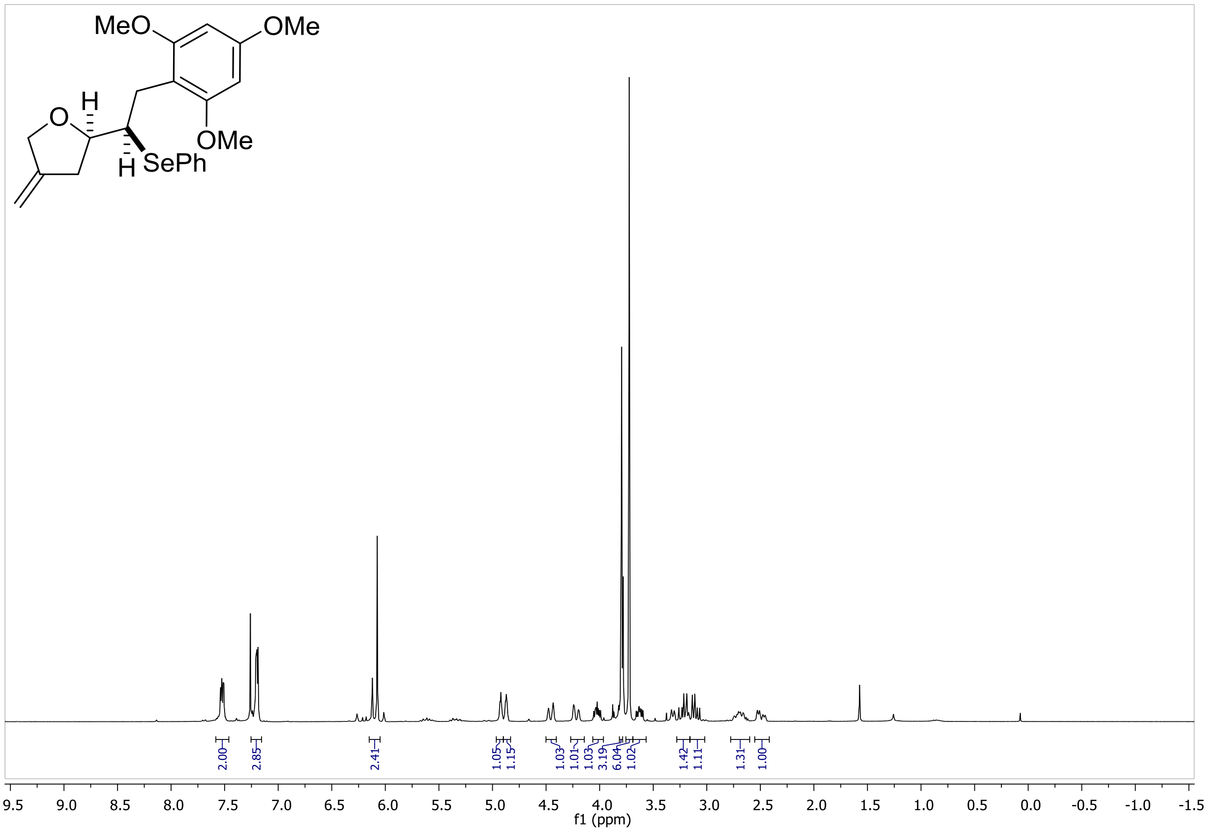

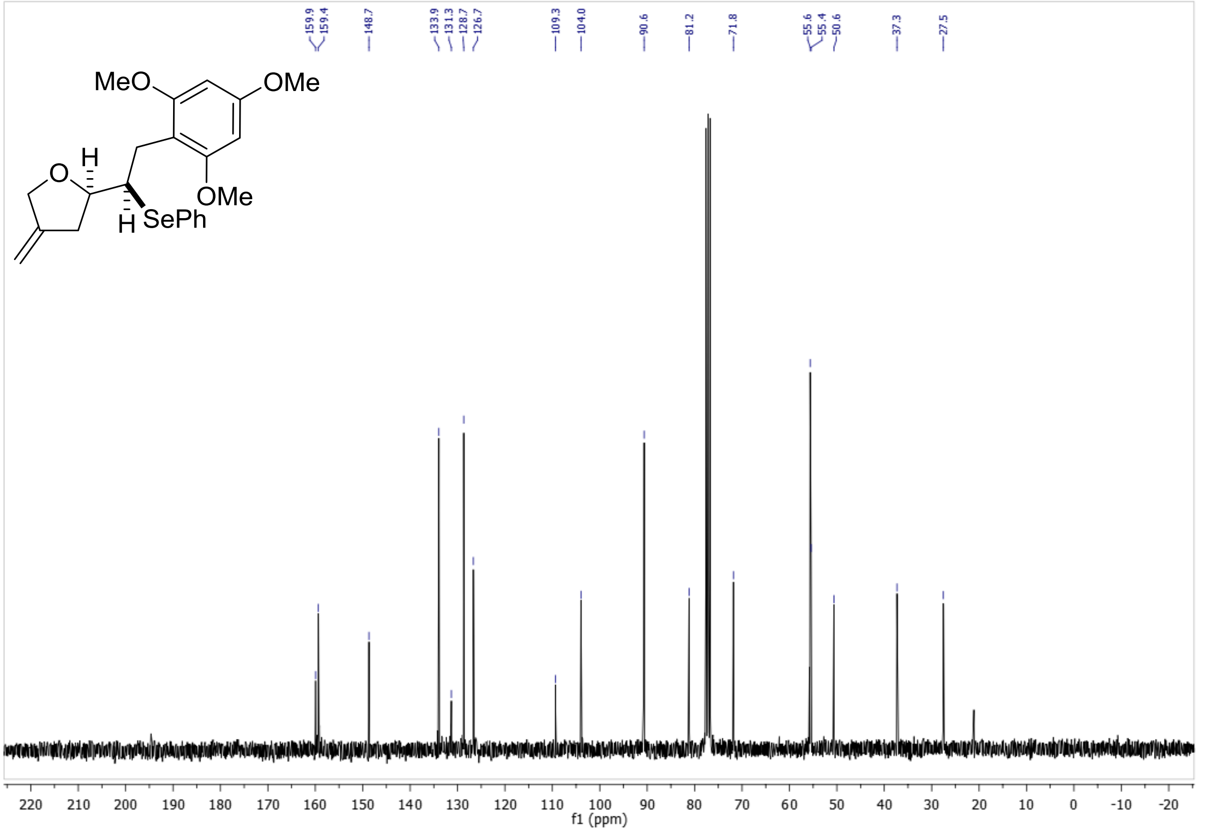

***threo*-3-(2-(4-Methylenetetrahydrofuran-2-yl)-2-(phenylselanyl)ethyl)furan (3j)**

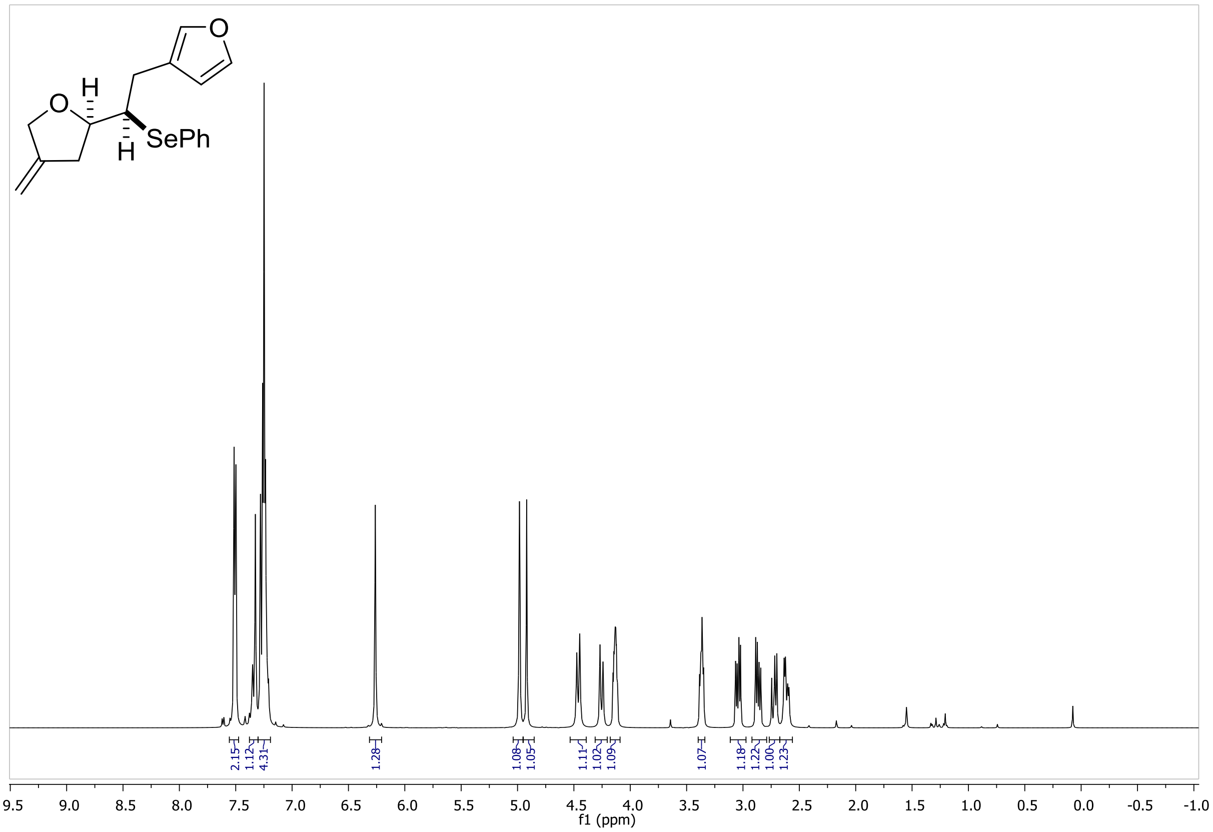

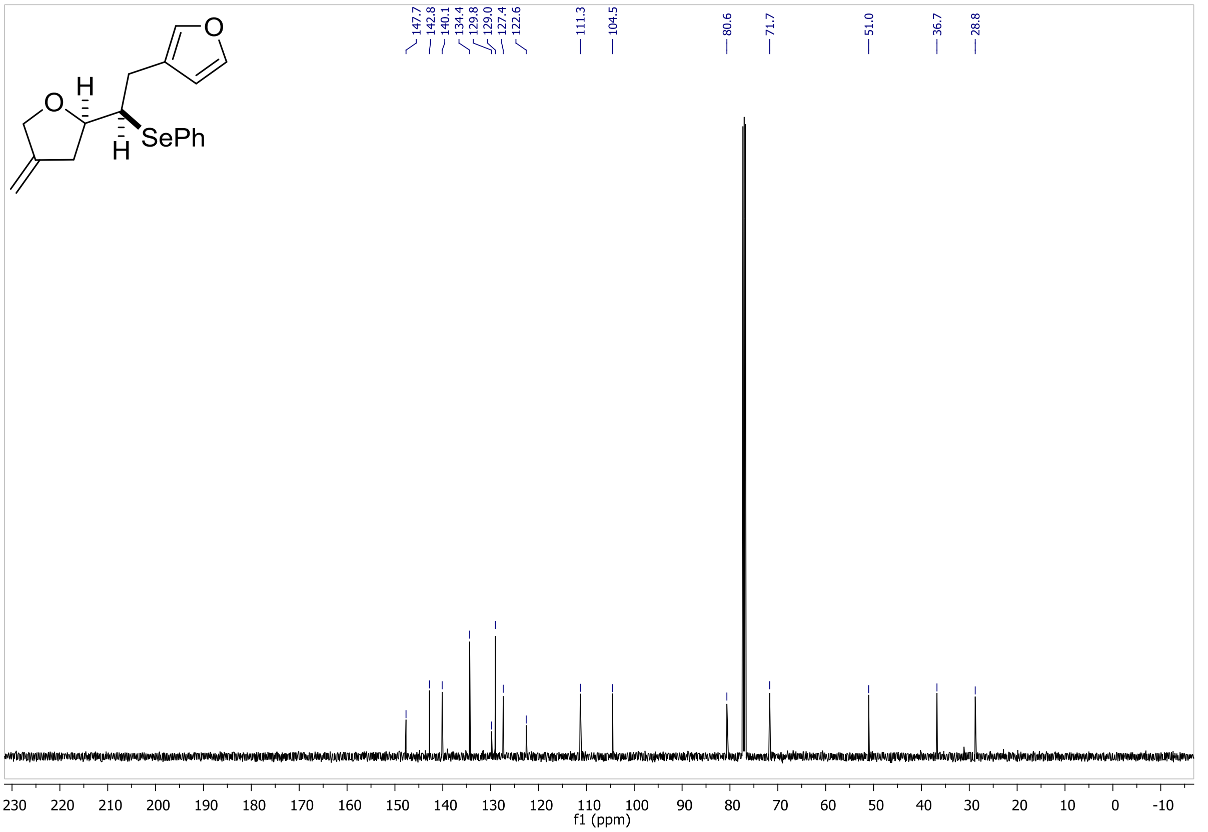

threo-4-Methylene-2-(1-(phenylselanyl)-2-(thiophen-2-yl)ethyl)tetrahydrofuran (3k)

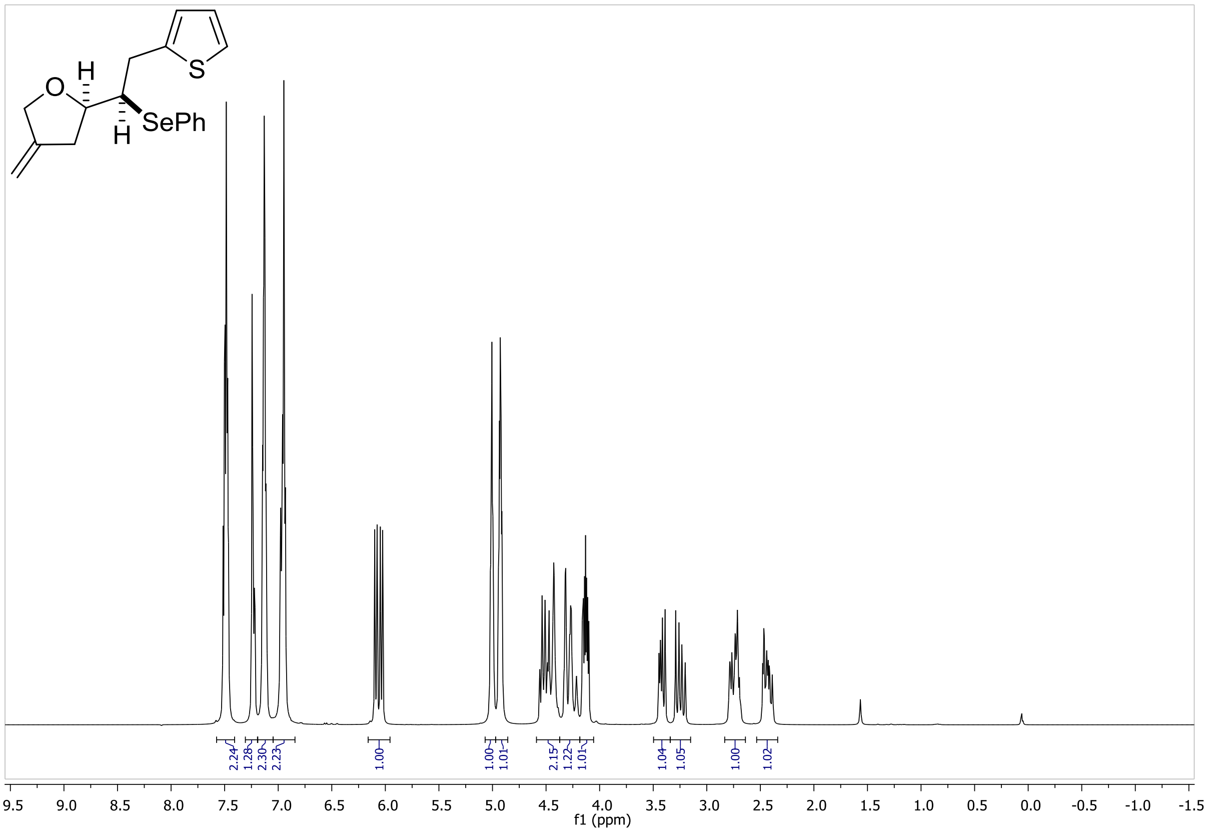

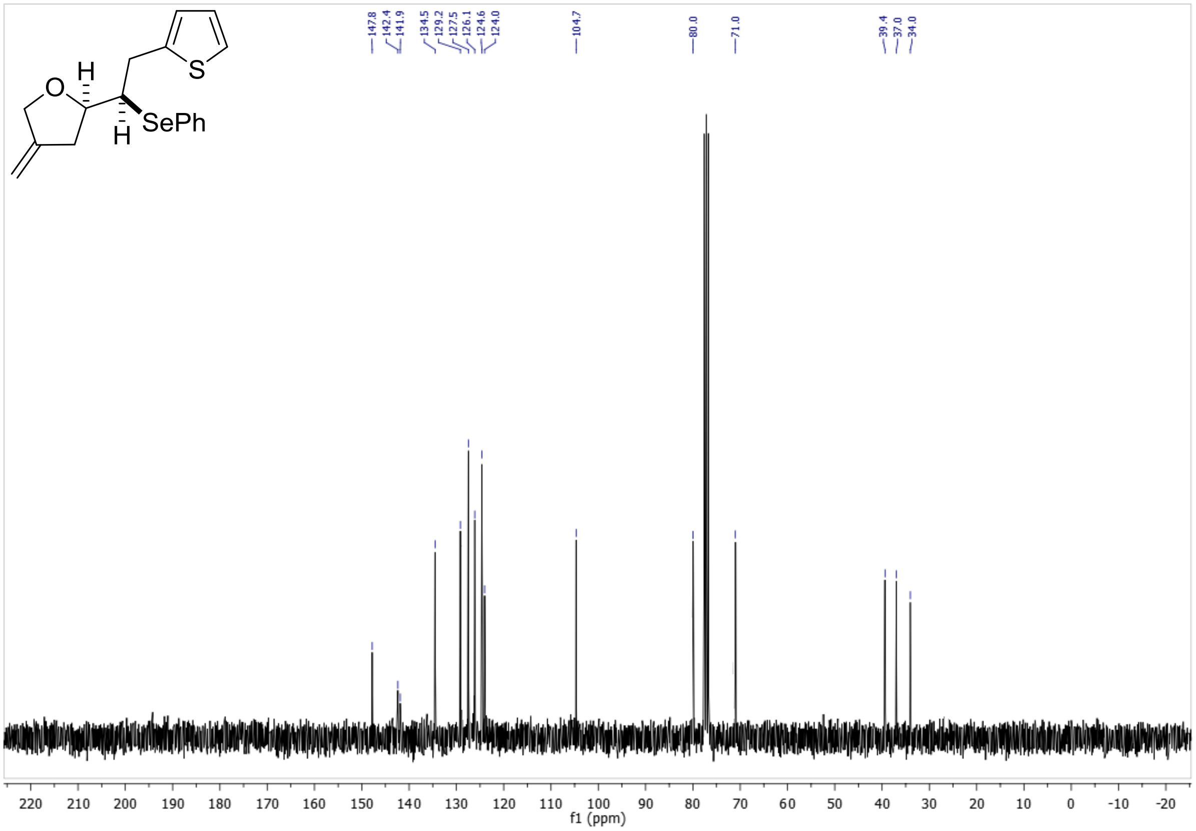

threo-4-Methylene-2-(1-(phenylselanyl)ethyl)tetrahydrofuran (3j)

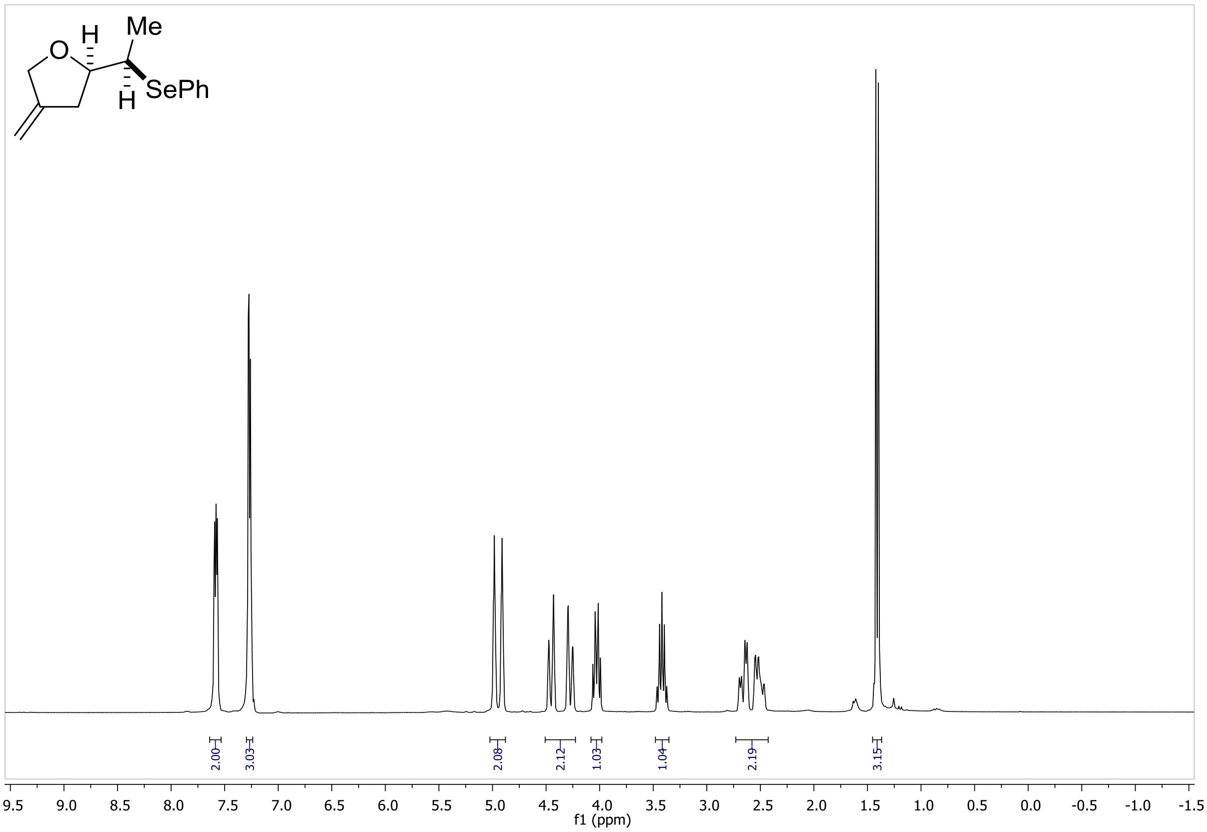

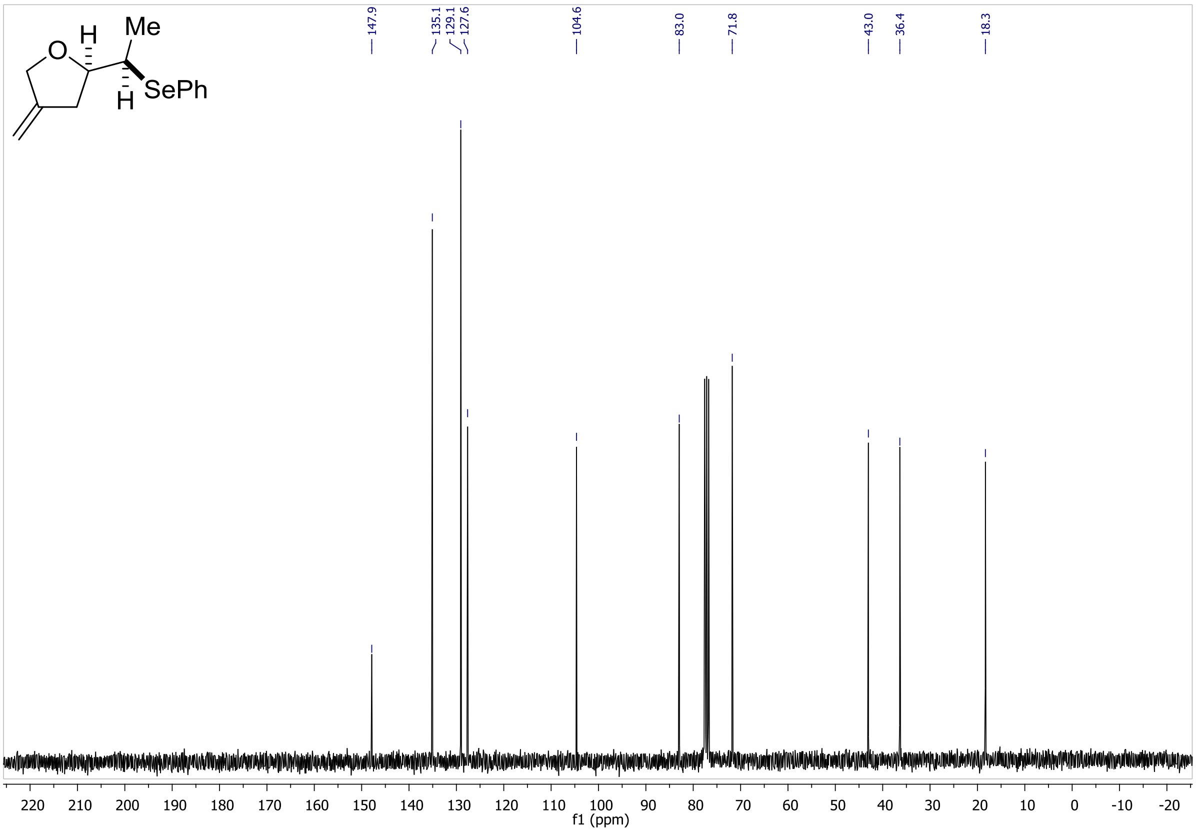

2-Methyl-4-methylene-2-(2-(phenylselanyl)propan-2-yl)tetrahydrofuran (3m)

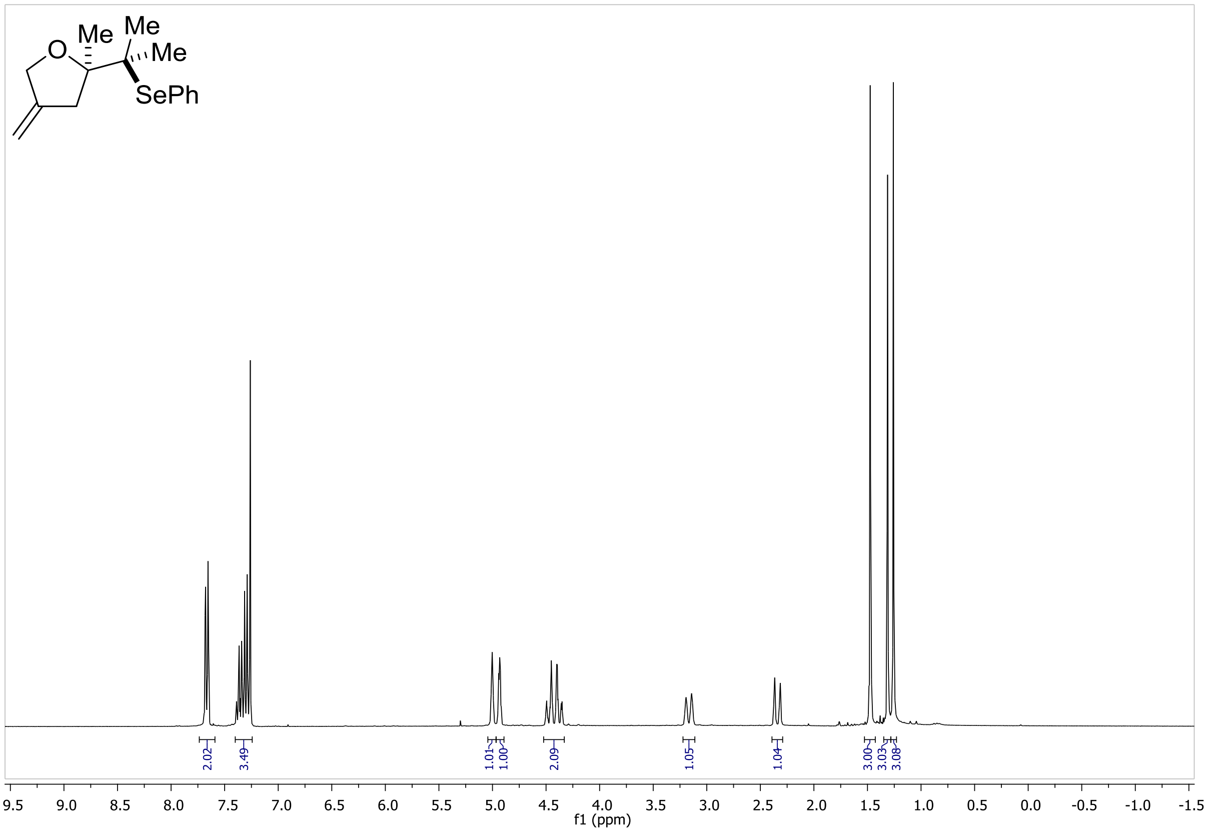

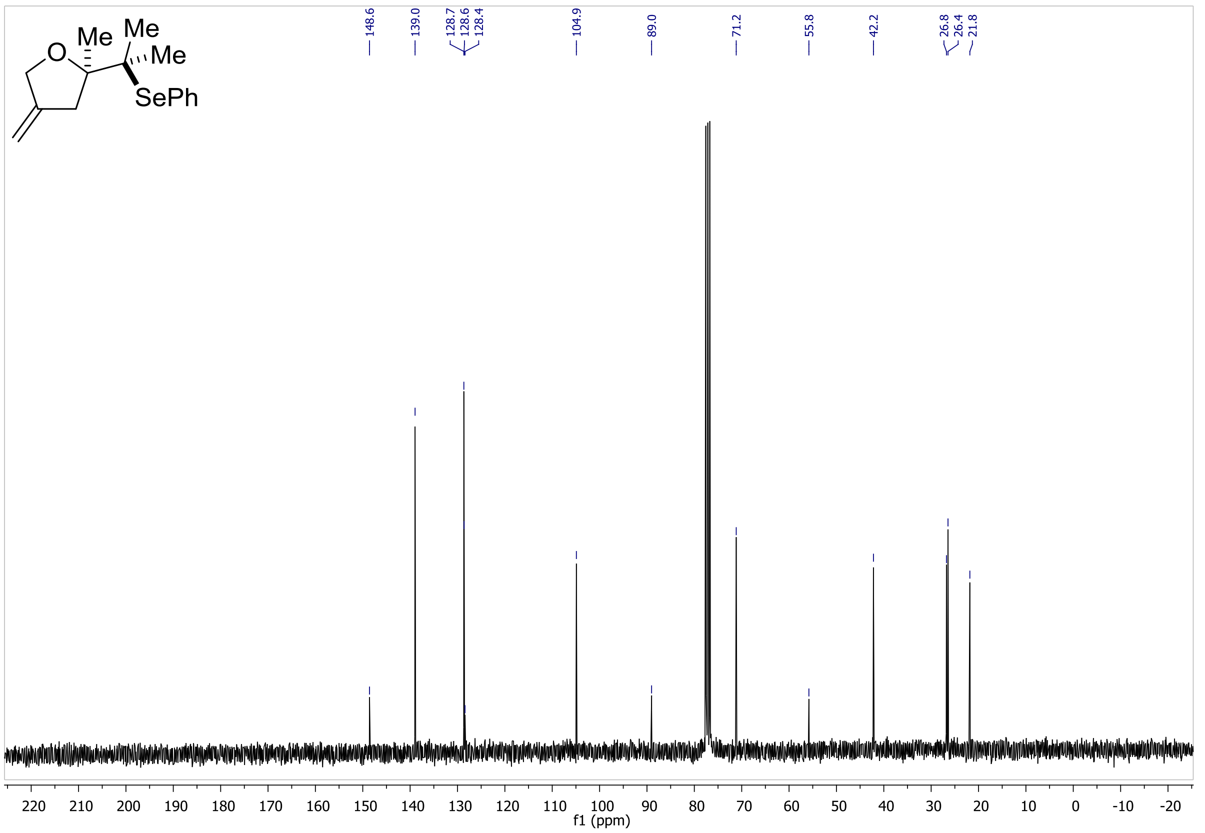

2,2,3-Trimethyl-5-methylene-3-(phenylselanyl)tetrahydro-2*H*-pyran (4m)

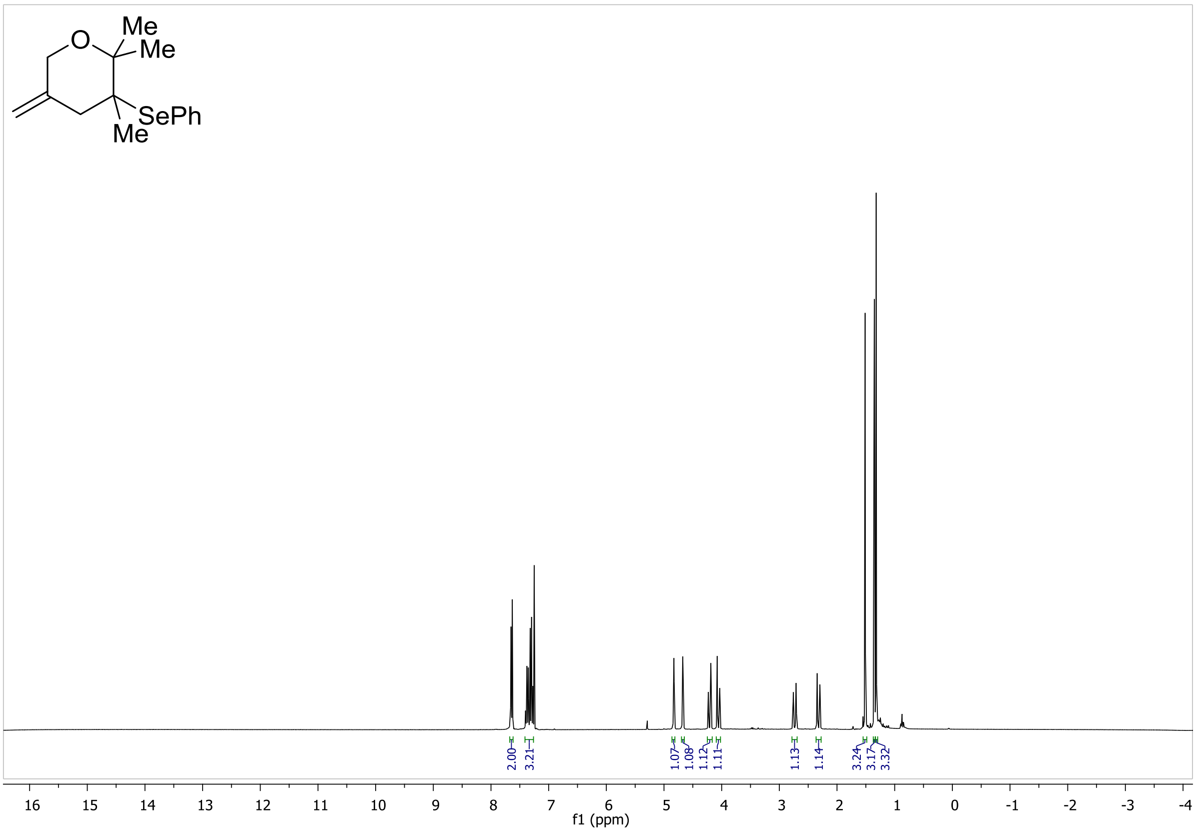

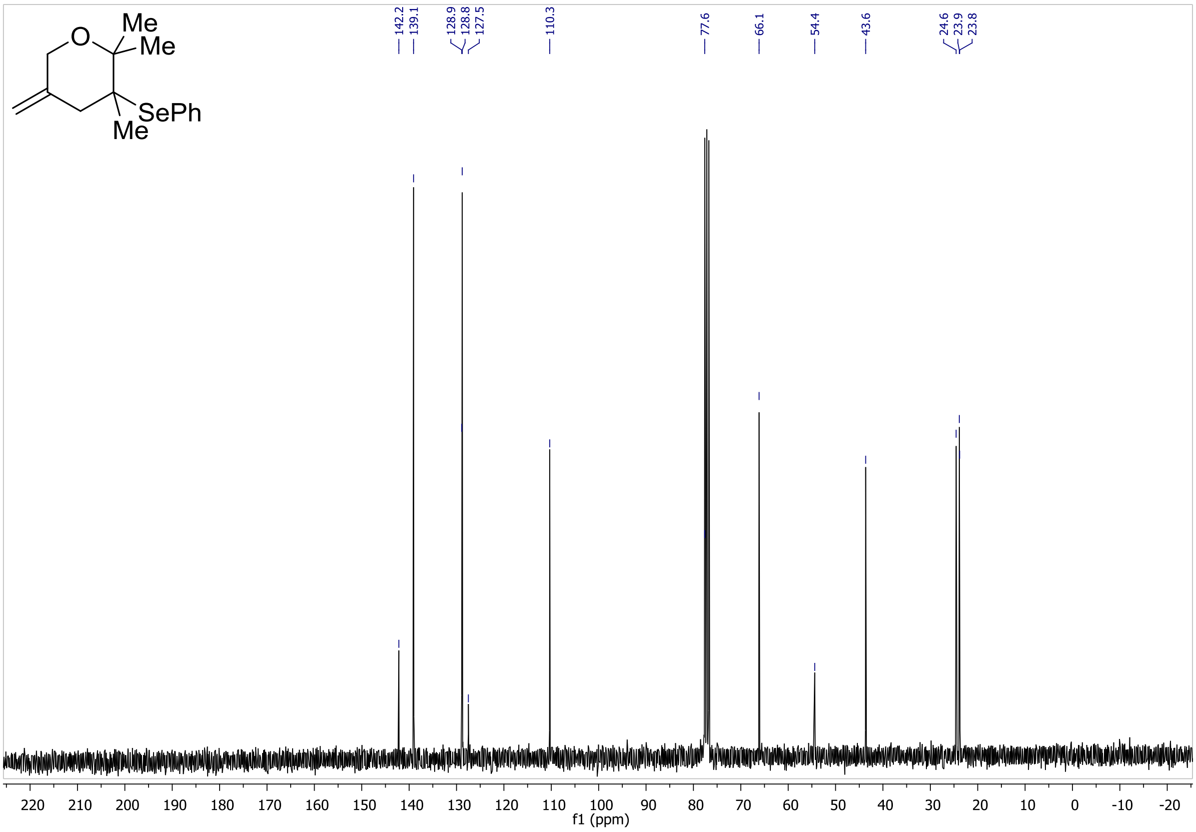

2,2-Dimethyl-5-methylene-3-(phenylselanyl)tetrahydro-2*H*-pyran (4n)

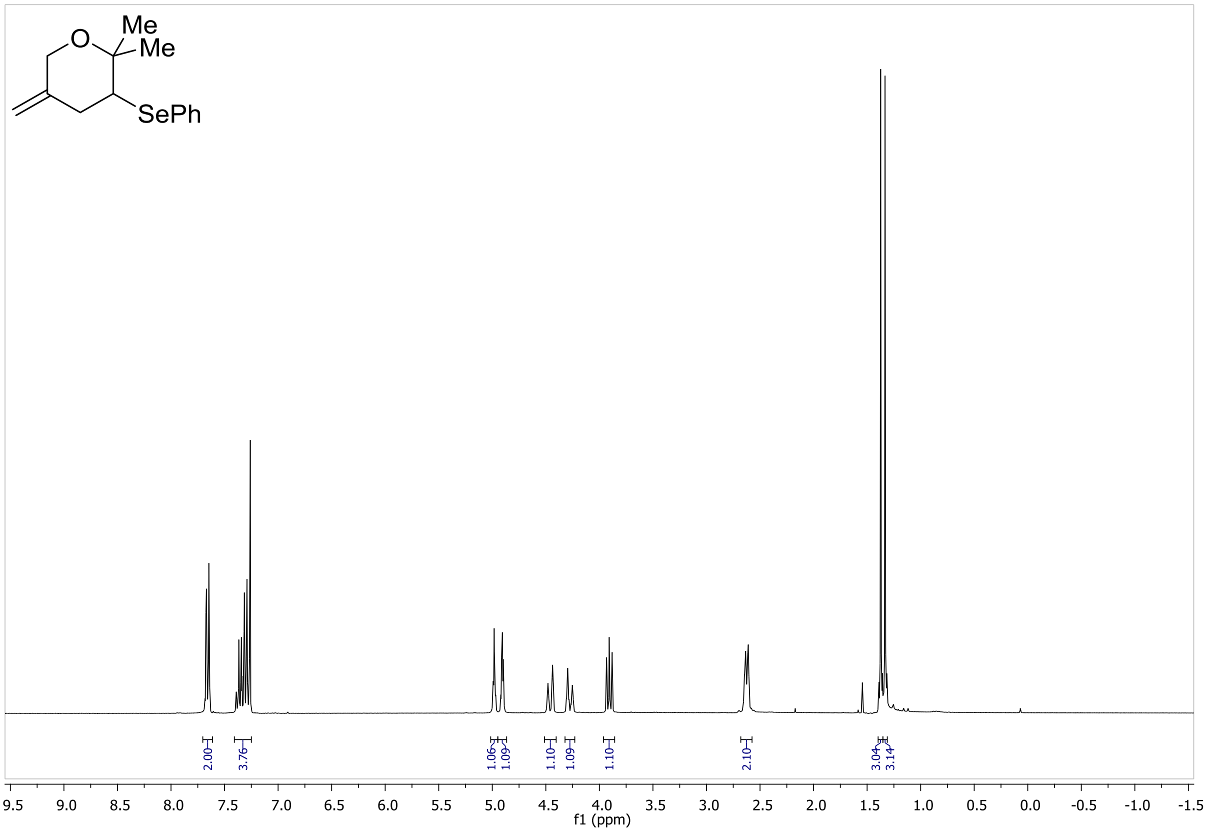

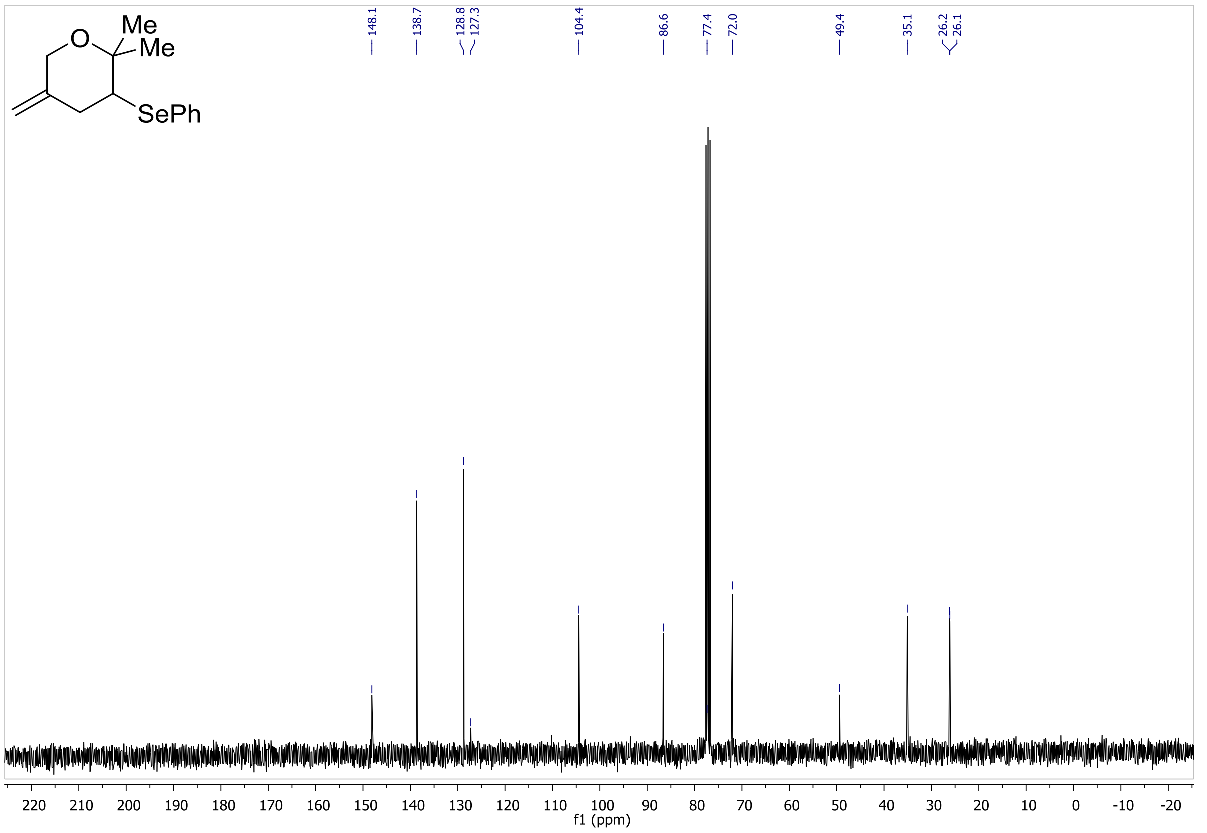

6-Heptyl-2,2,3-trimethyl-5-methylene-3-(phenylselanyl)tetrahydro-2*H*-pyran (4p)

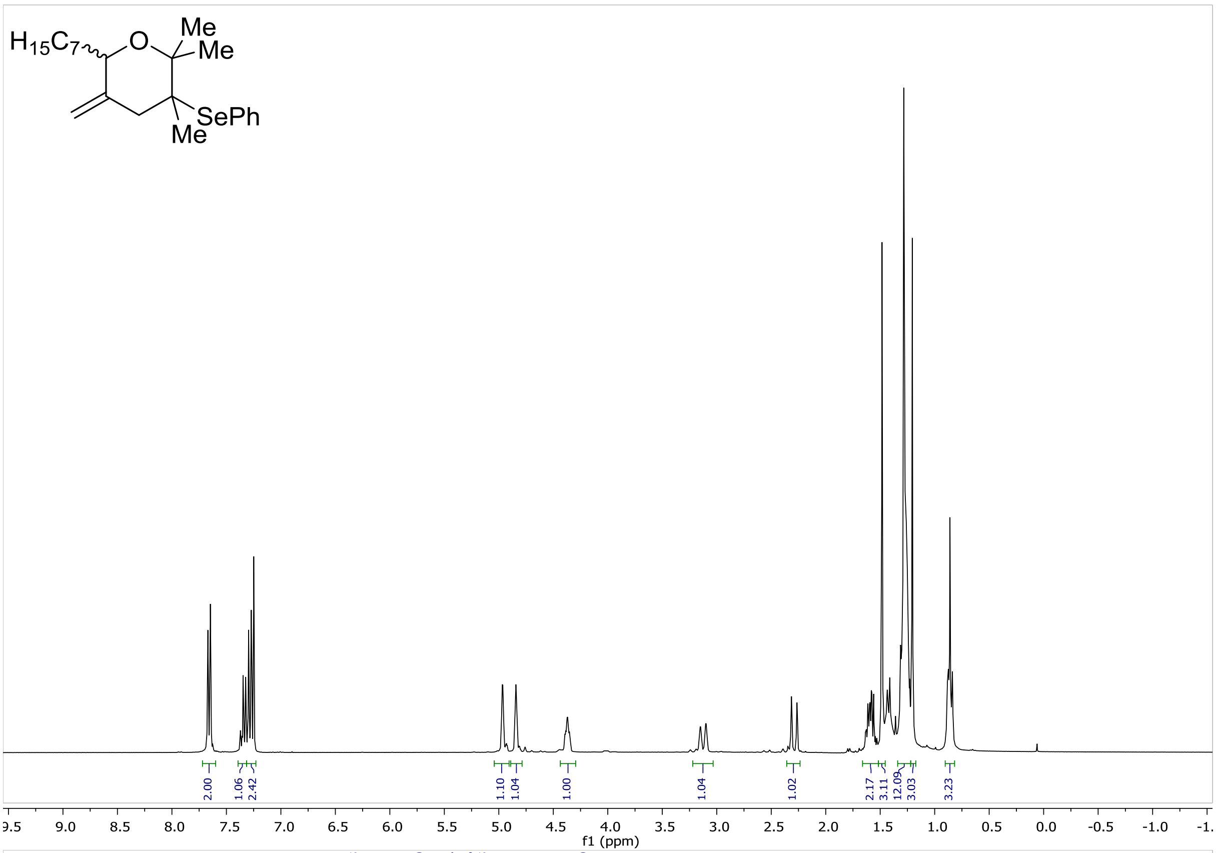

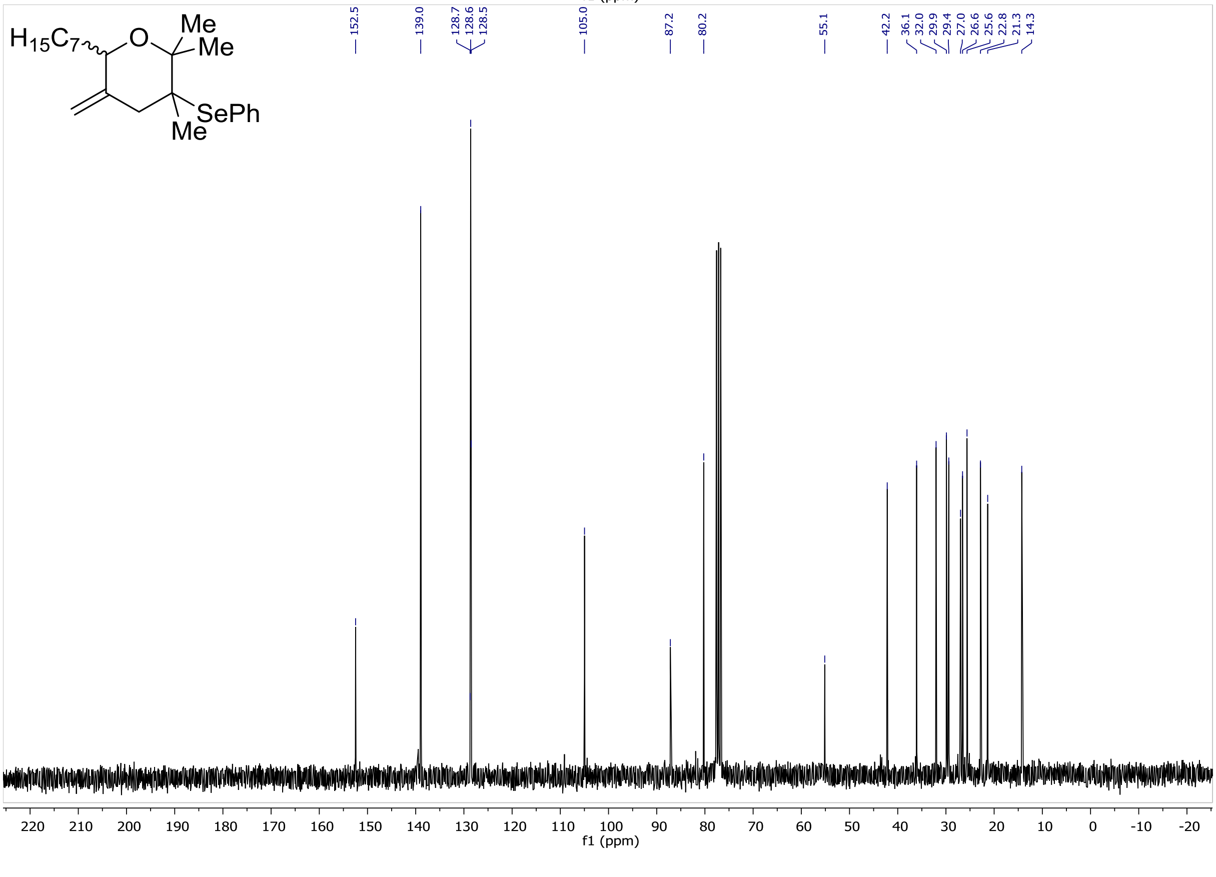

6-(4-Bromophenyl)-2,2,3-trimethyl-5-methylene-3-(phenylselanyl)tetrahydro-2*H*-pyran (4q)

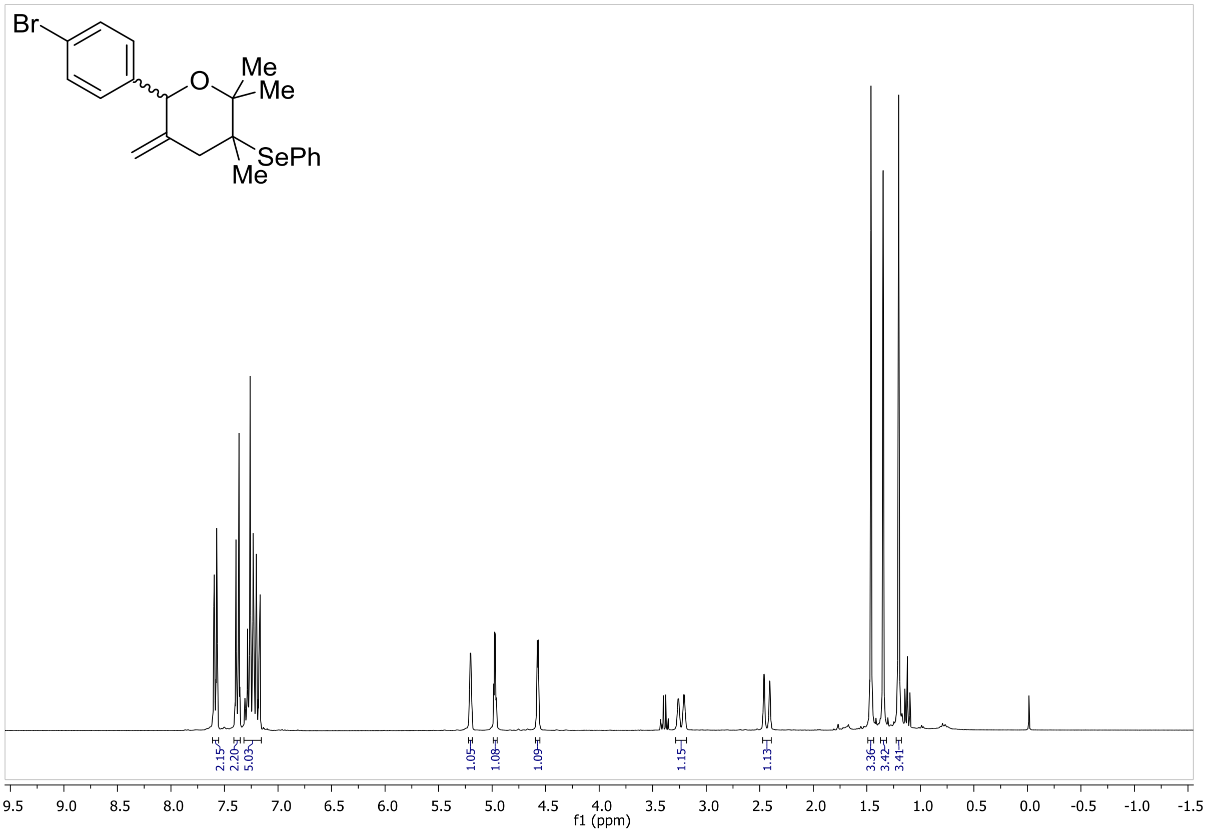

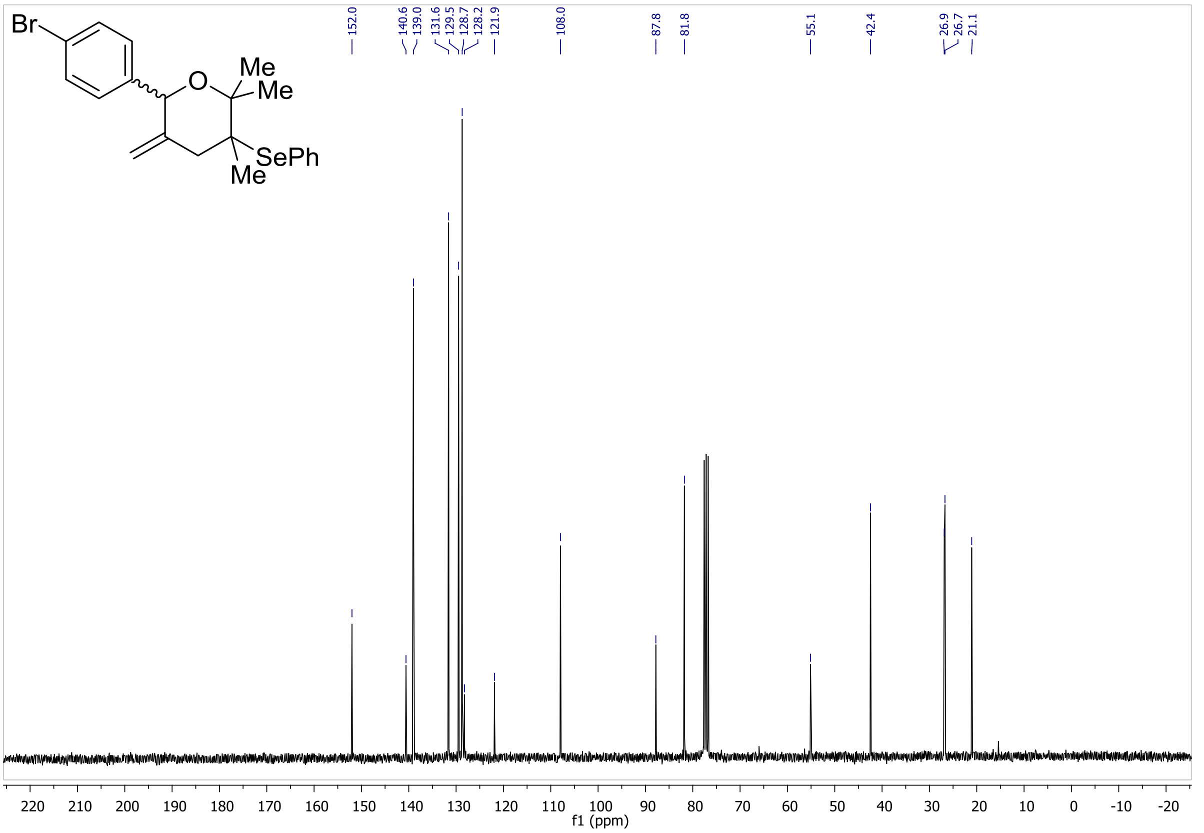

***threo*-2-(1-Iodo-2-phenylethyl)-4-methylenetetrahydrofuran (5a)**

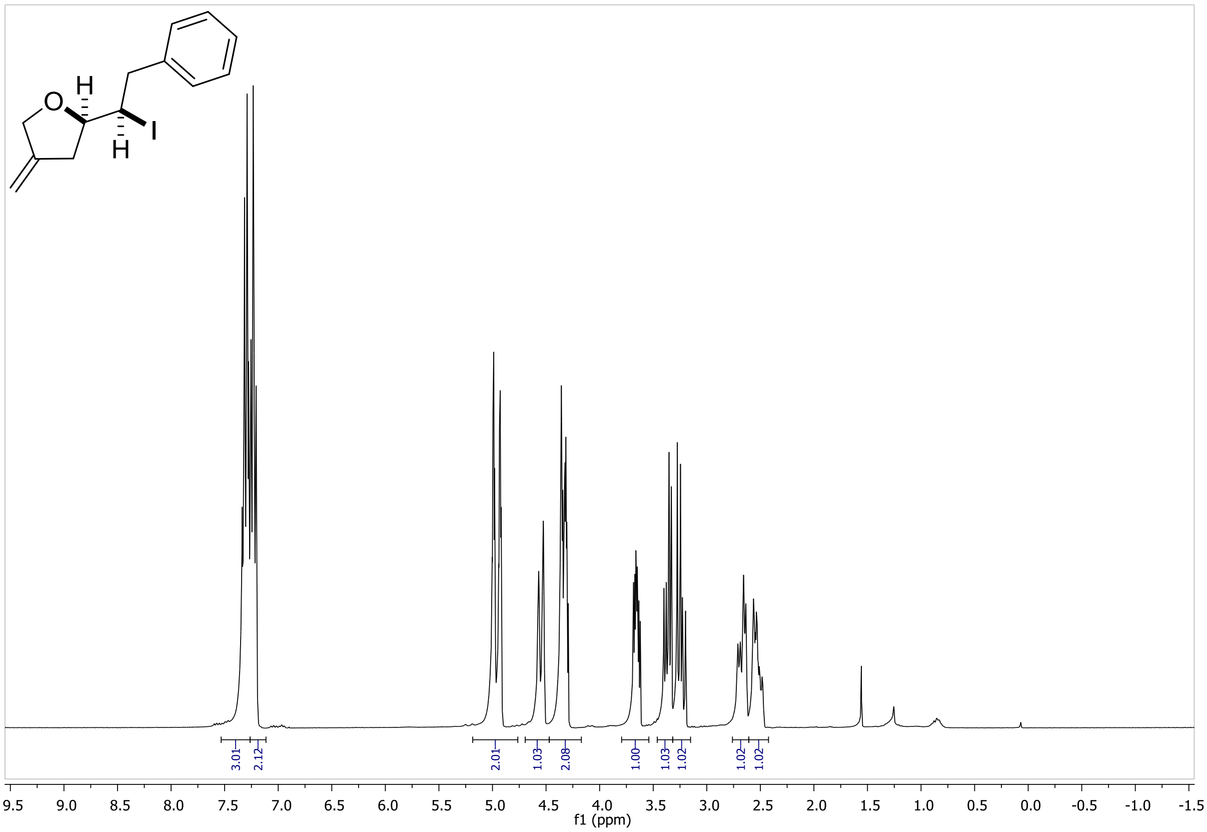

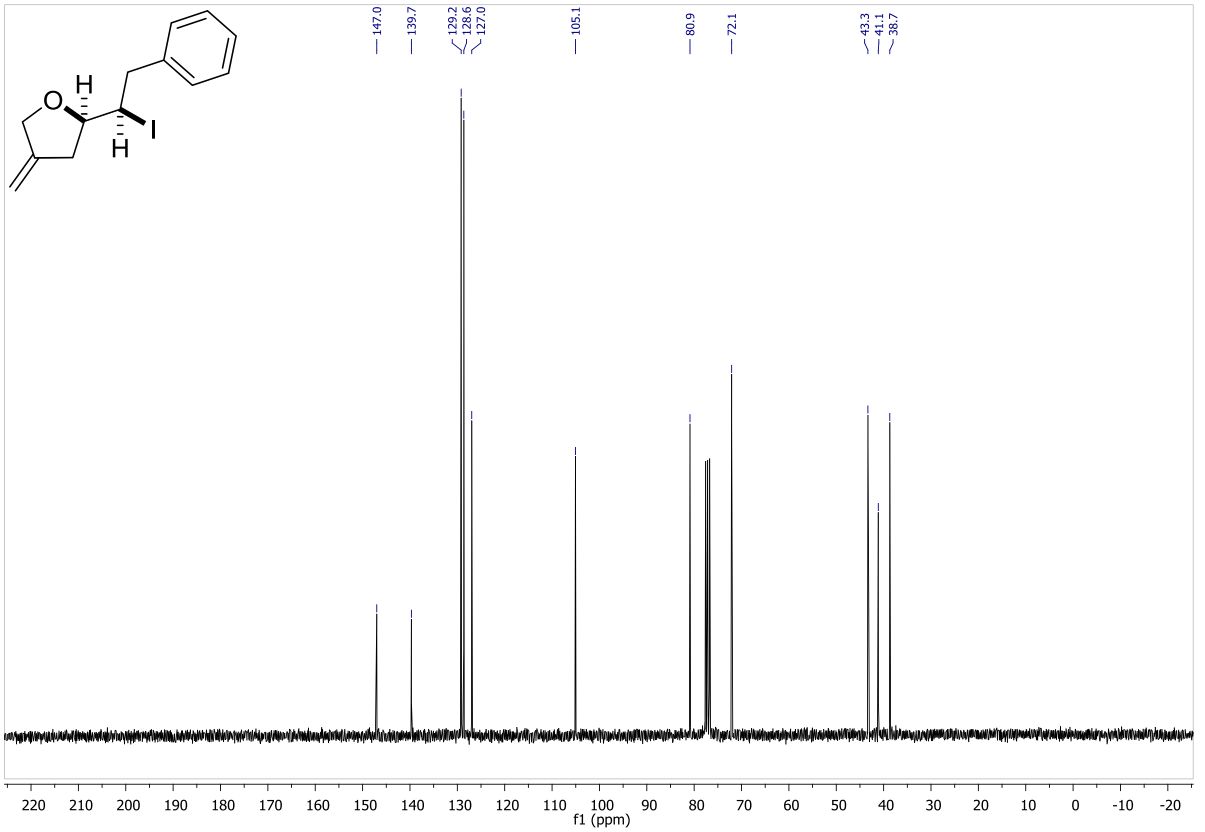

***threreo*-2-(2-(4-Bromophenyl)-1-iodoethyl)-4-methylenetetrahydrofuran (5b)**

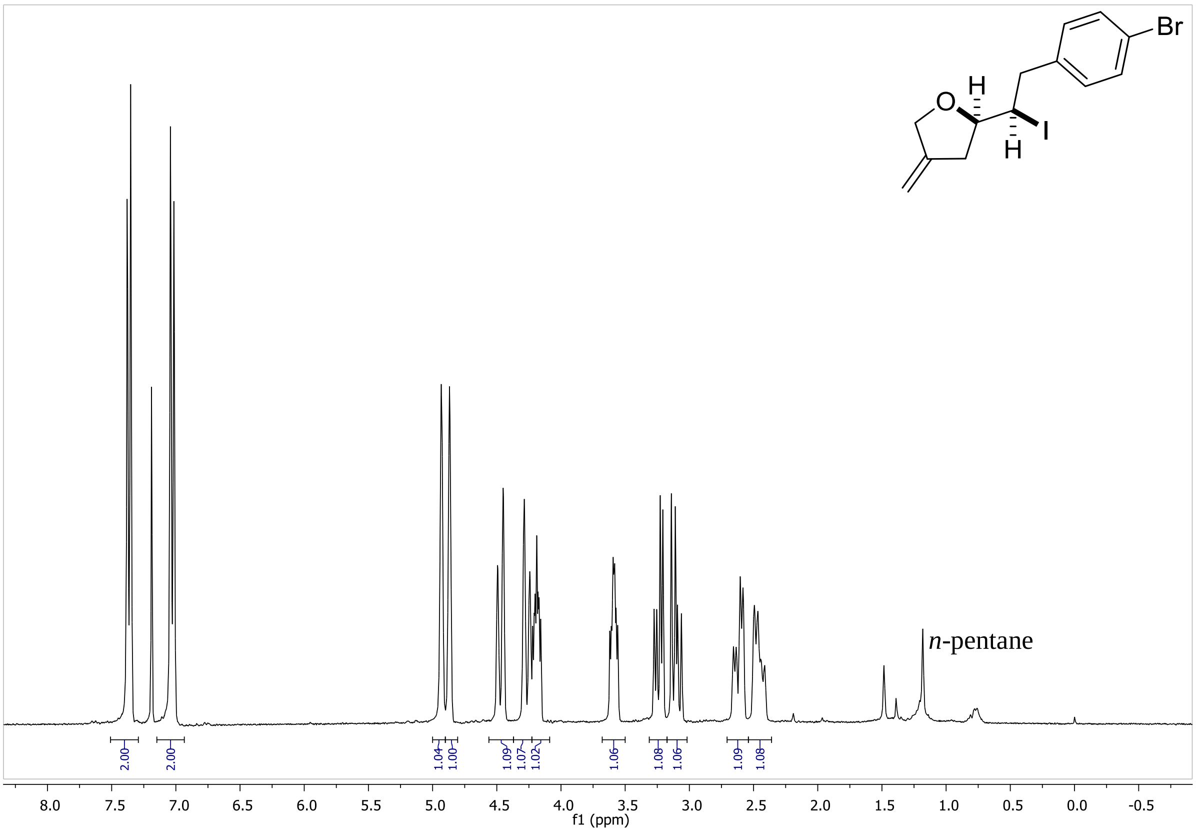

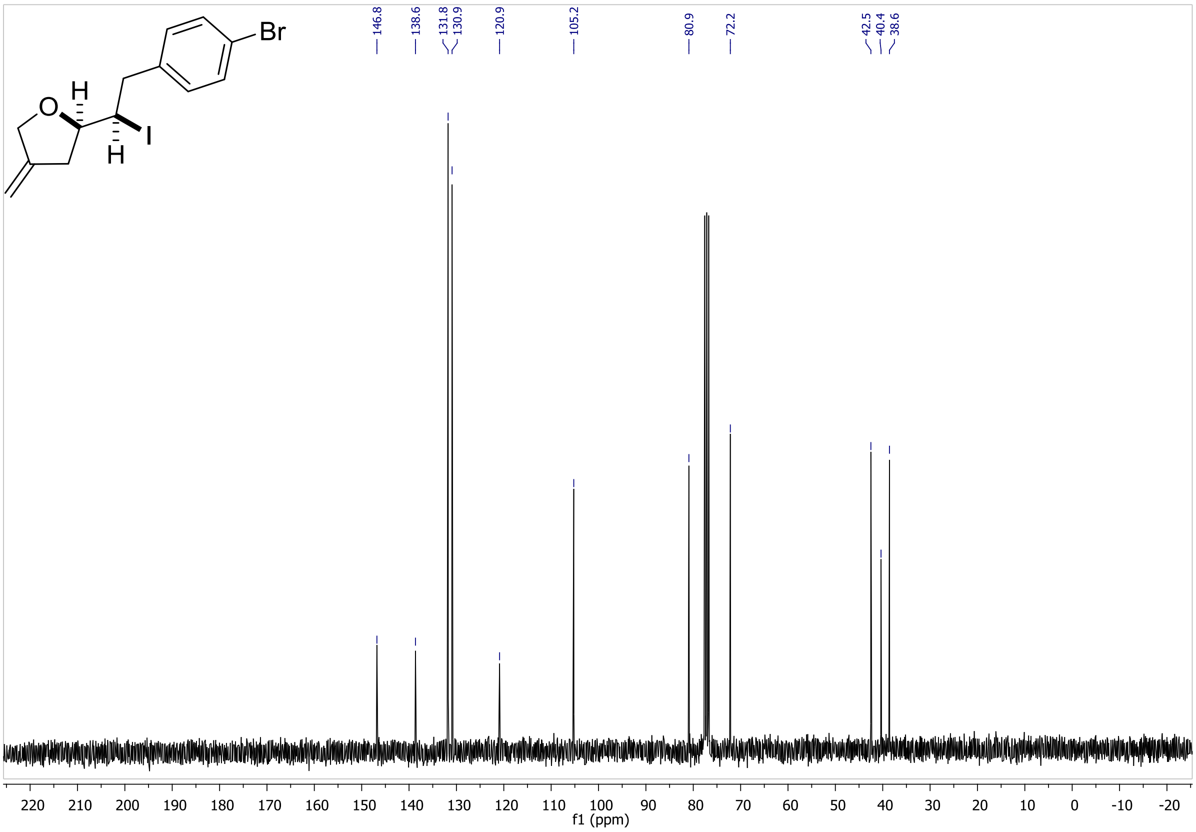

***threo*-2-(2-(2-Chlorophenyl)-1-iodoethyl)-4-methylenetetrahydrofuran (5c)**

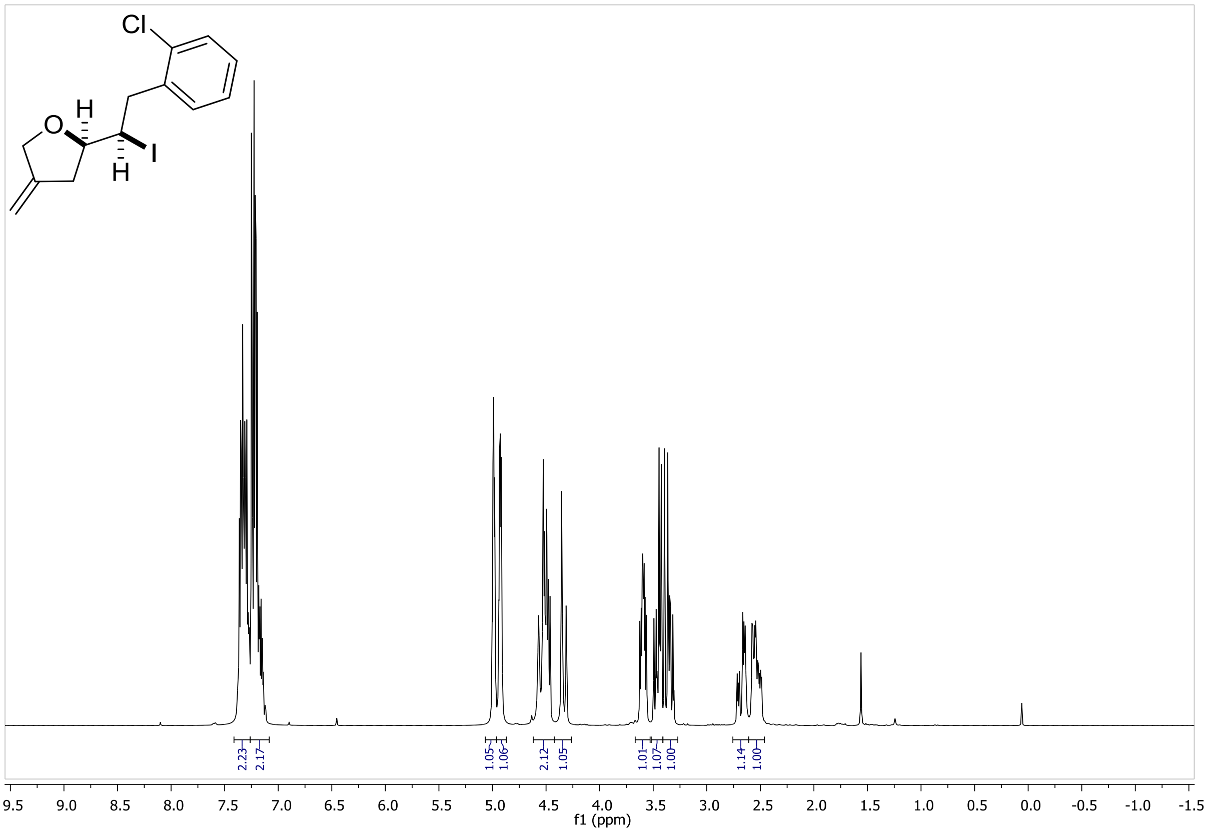

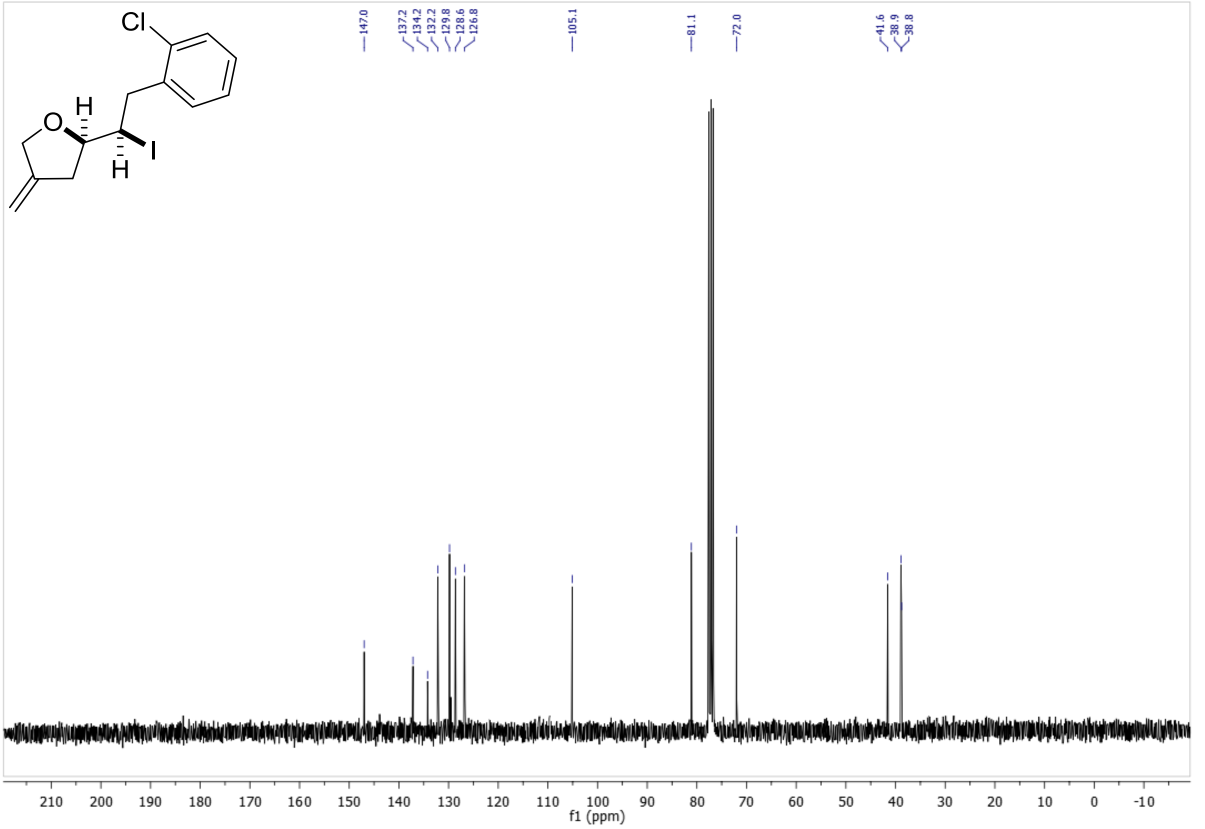

***threreo*-Methyl 3-(2-iodo-2-(4-methylenetetrahydrofuran-2-yl)ethyl)benzoate (5d)**

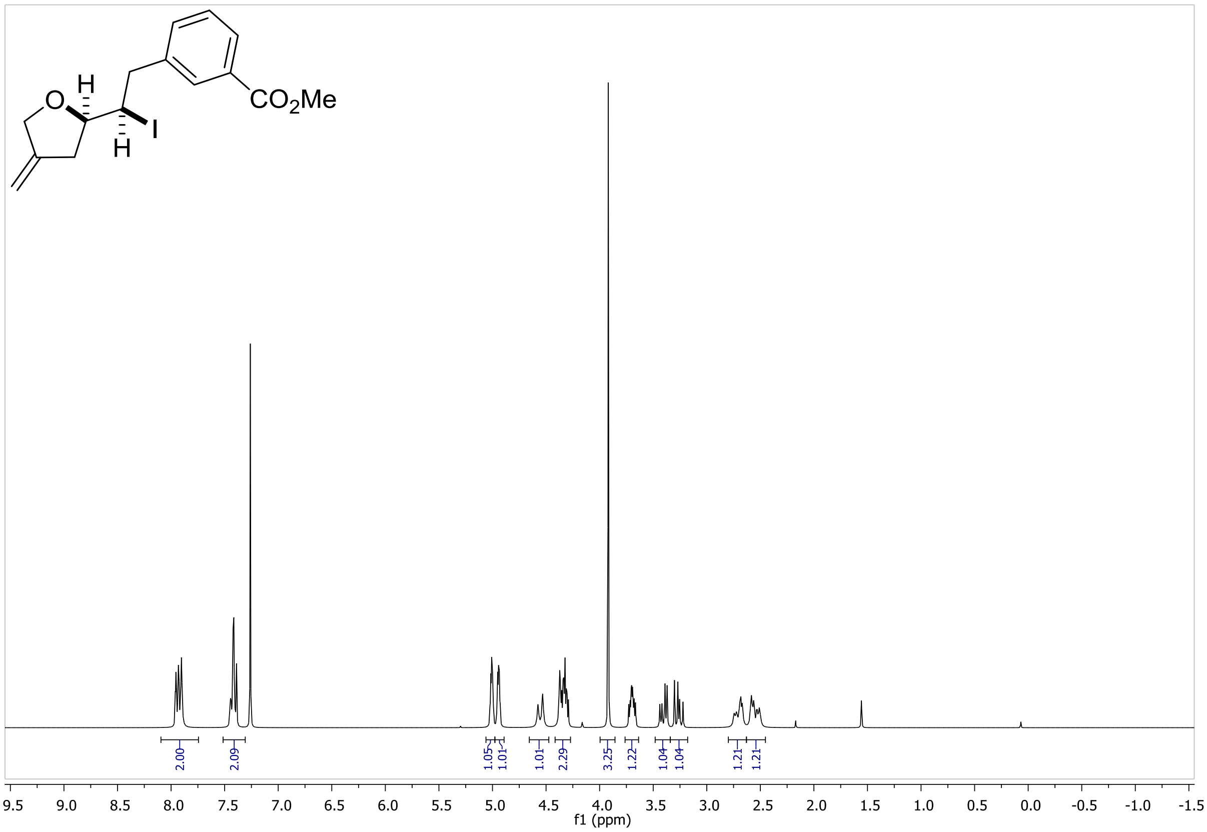

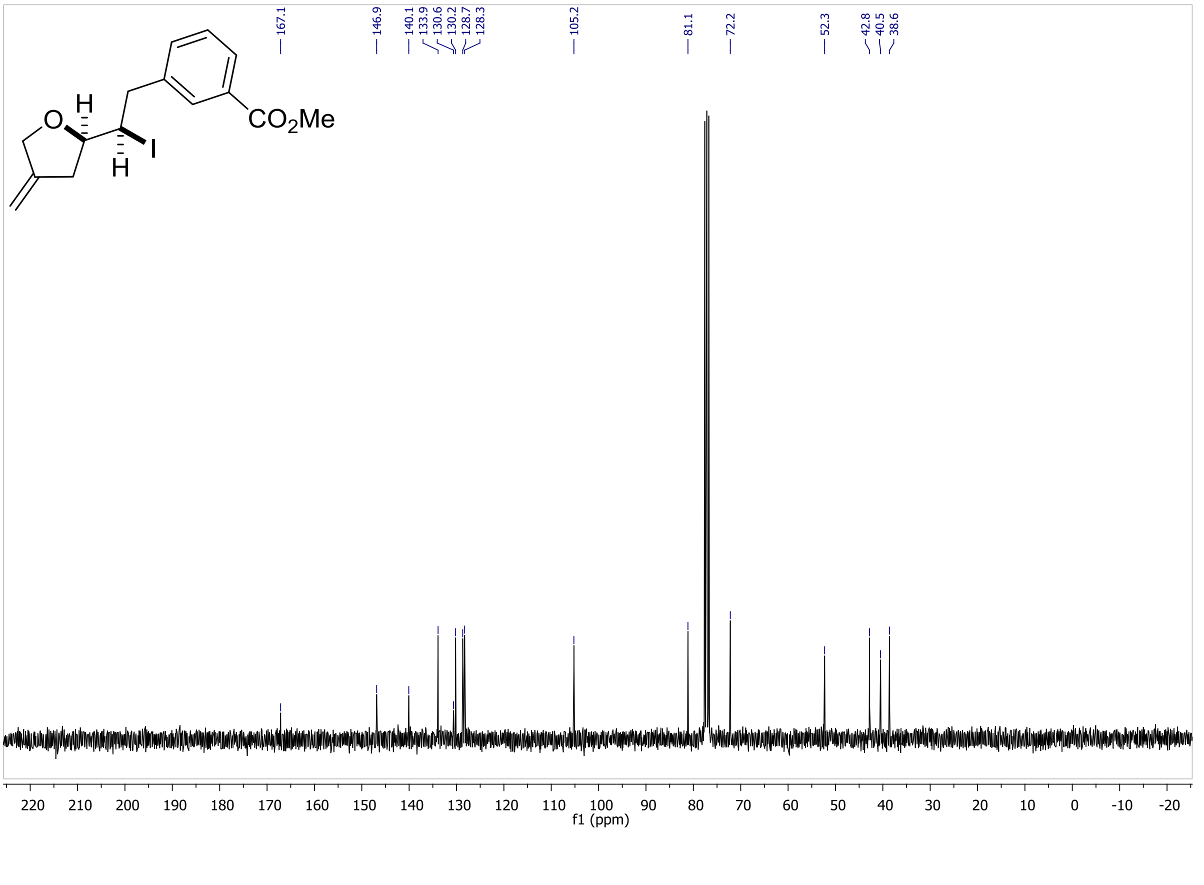

***threo*-2-(1-Iodo-2-(4-(trifluoromethyl)phenyl)ethyl)-4-methylenetetrahydrofuran (5e)**

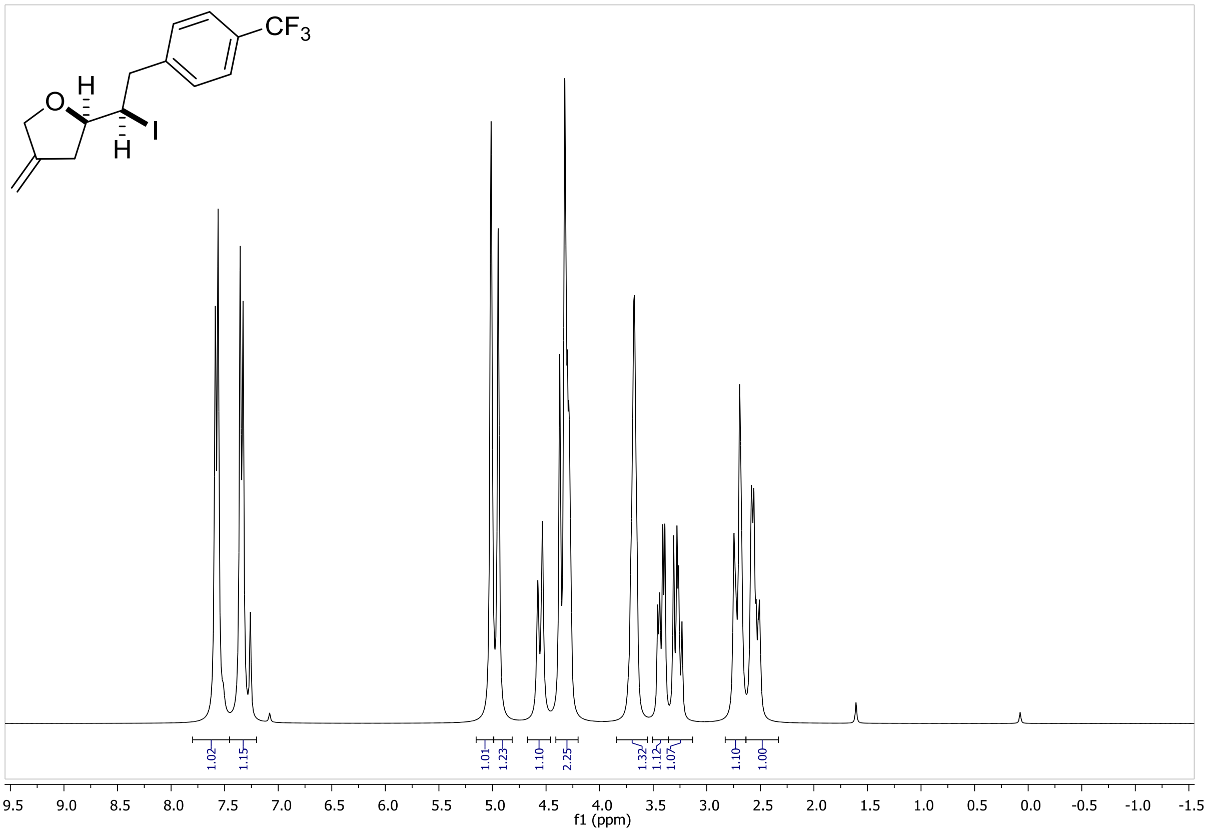

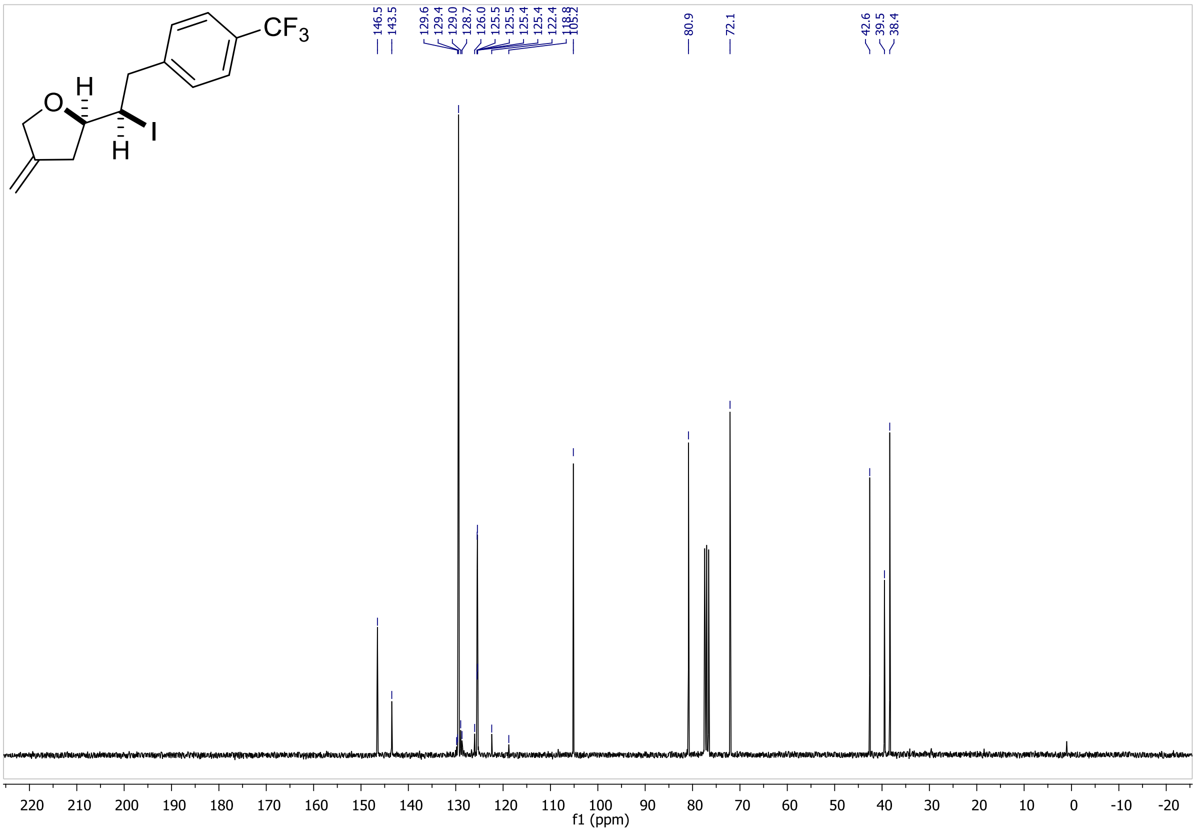

***threo*-2-(1-Iodo-2-(4-methoxyphenyl)ethyl)-4-methylenetetrahydrofuran (5f)**

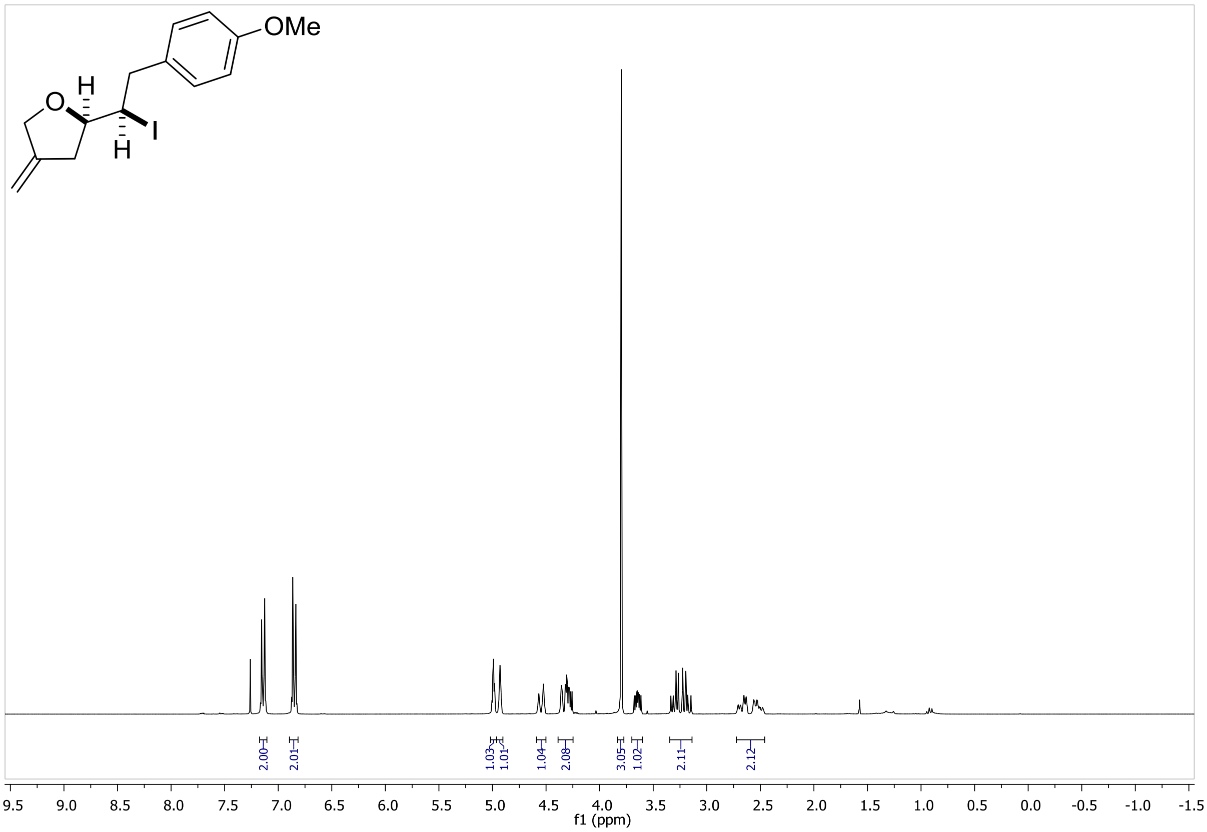

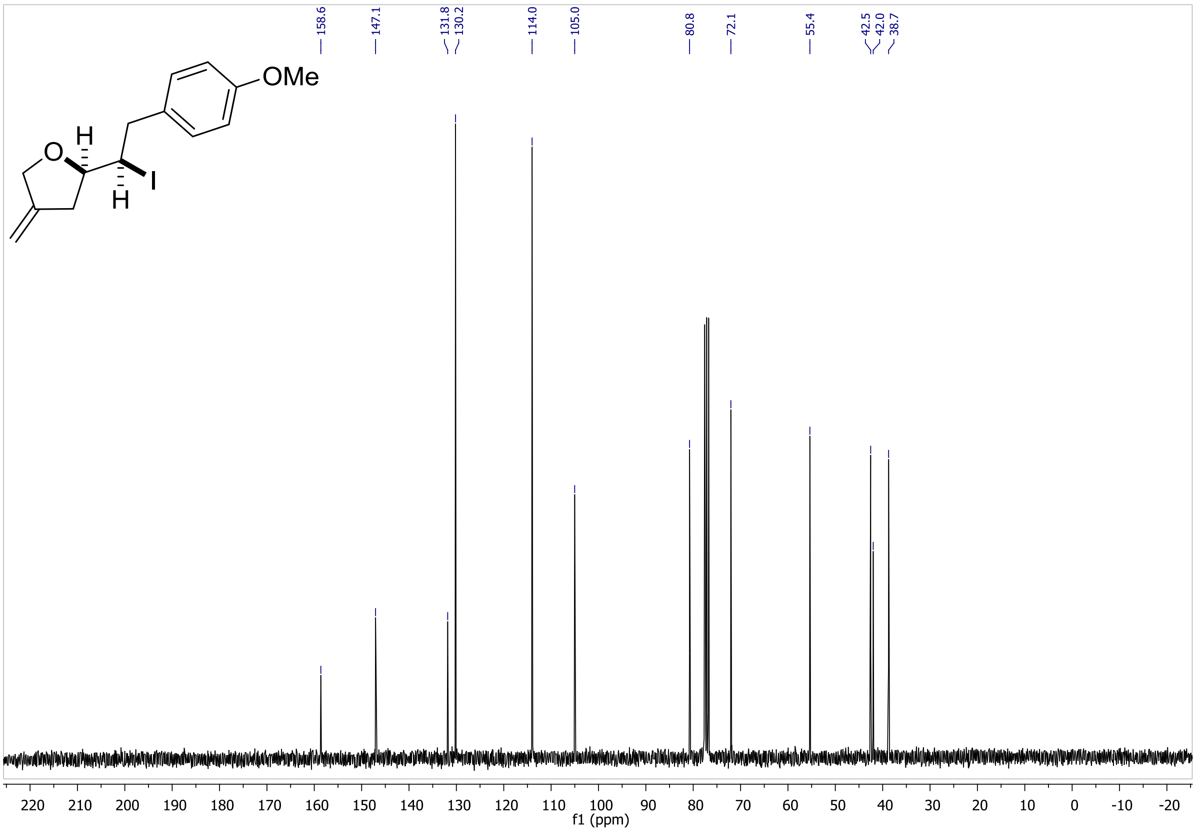

threo-2-(1-Iodo-2-(2,4,6-trimethoxyphenyl)ethyl)-4-methylenetetrahydrofuran (5g)

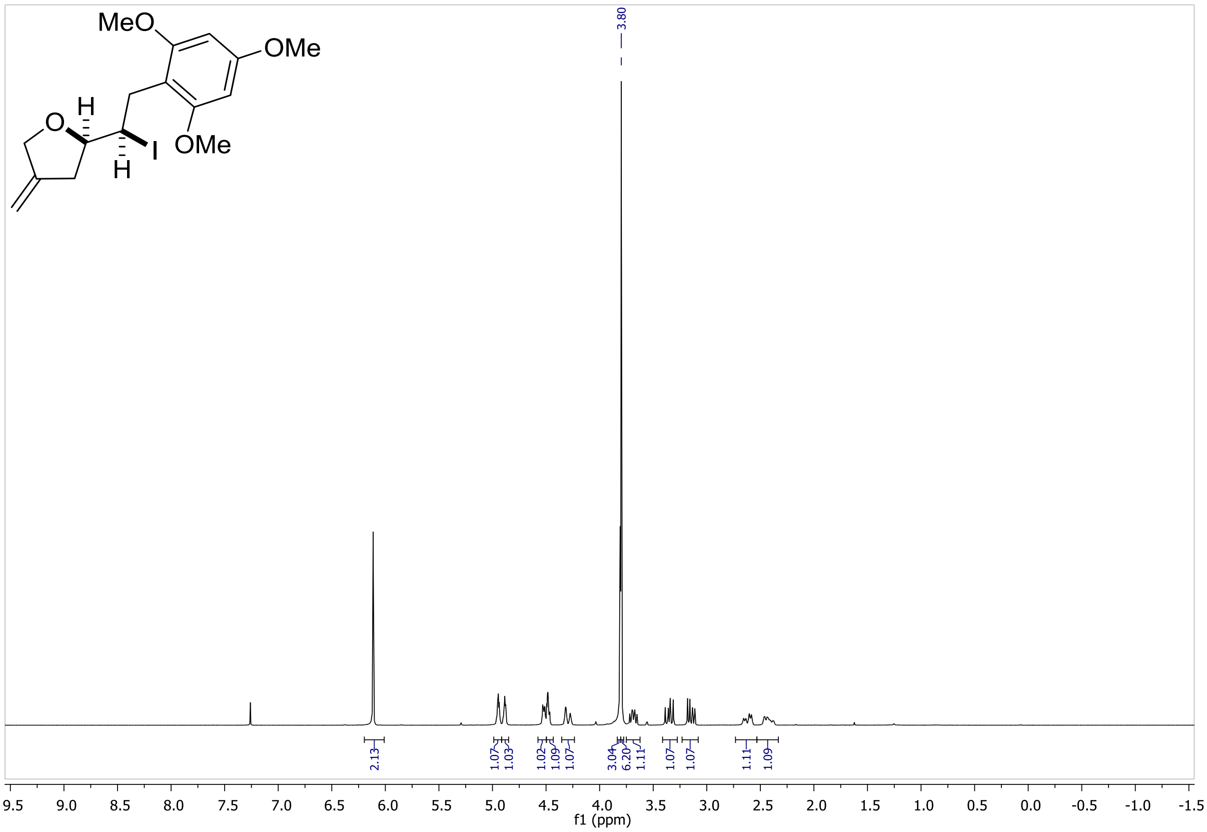

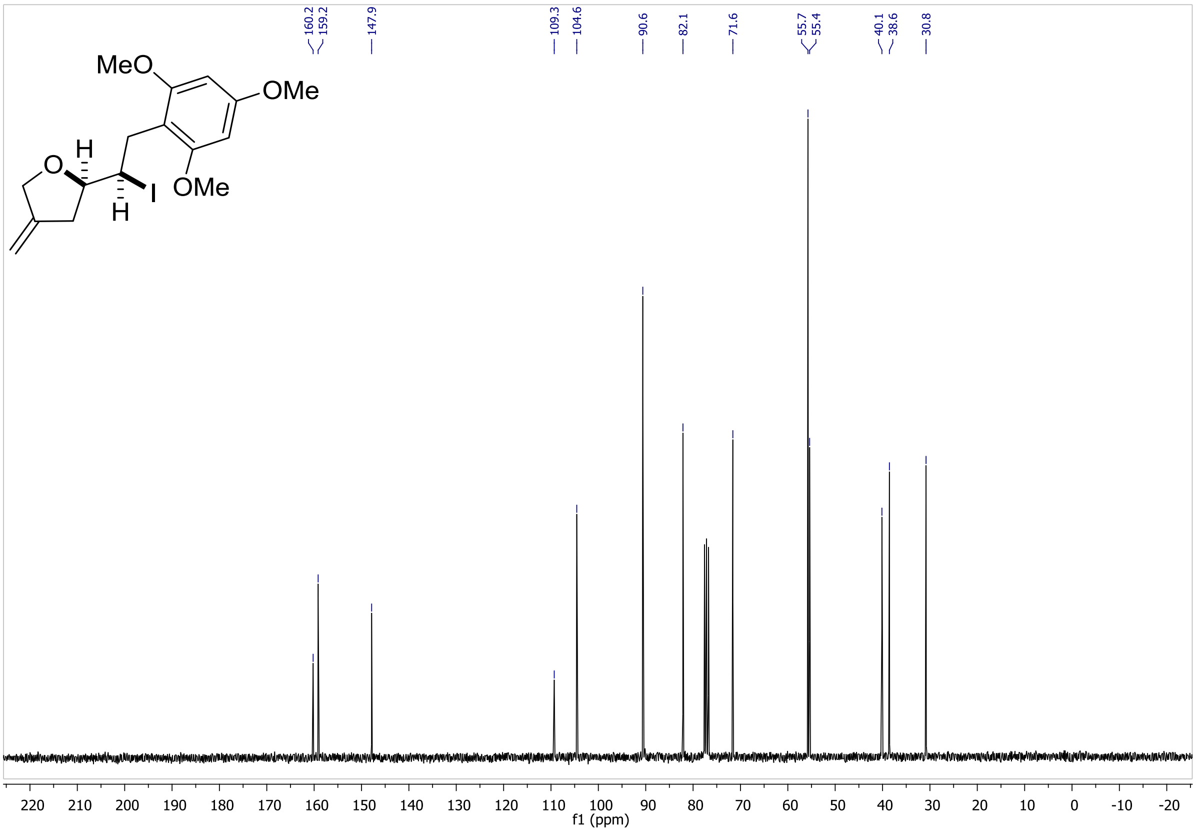

threo-2-(2-(3,4-Dimethoxyphenyl)-1-iodoethyl)-4-methylenetetrahydrofuran (5h)

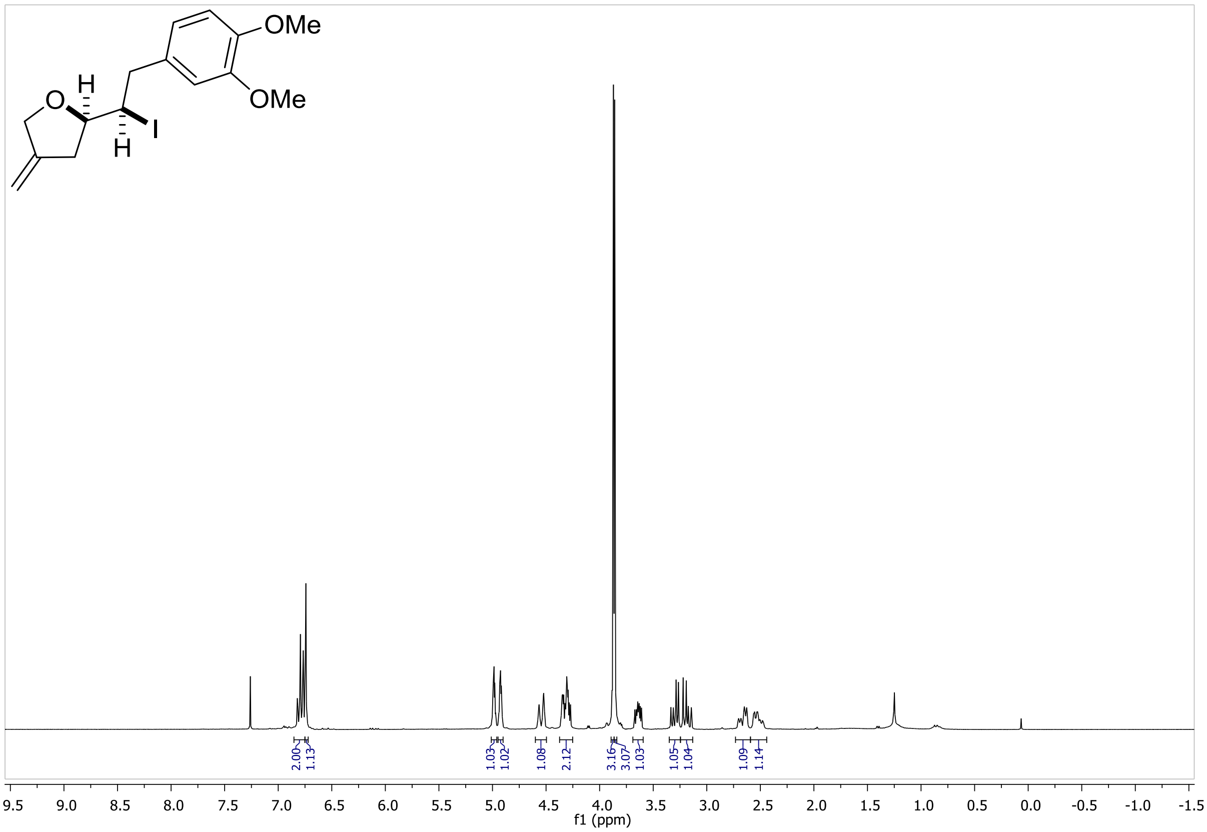

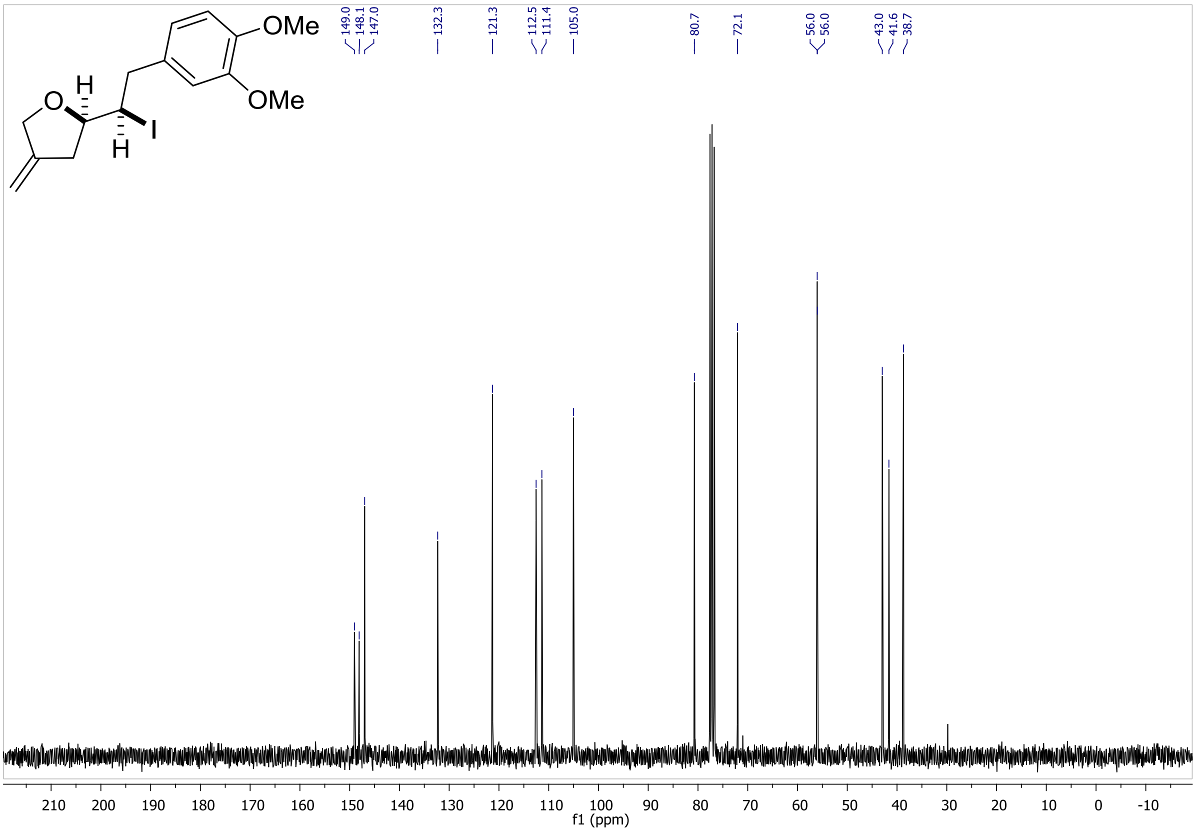

***threo*-2-(2-(2-Bromo-4,5-dimethoxyphenyl)-1-iodoethyl)-4-methylenetetrahydrofuran (5i)**

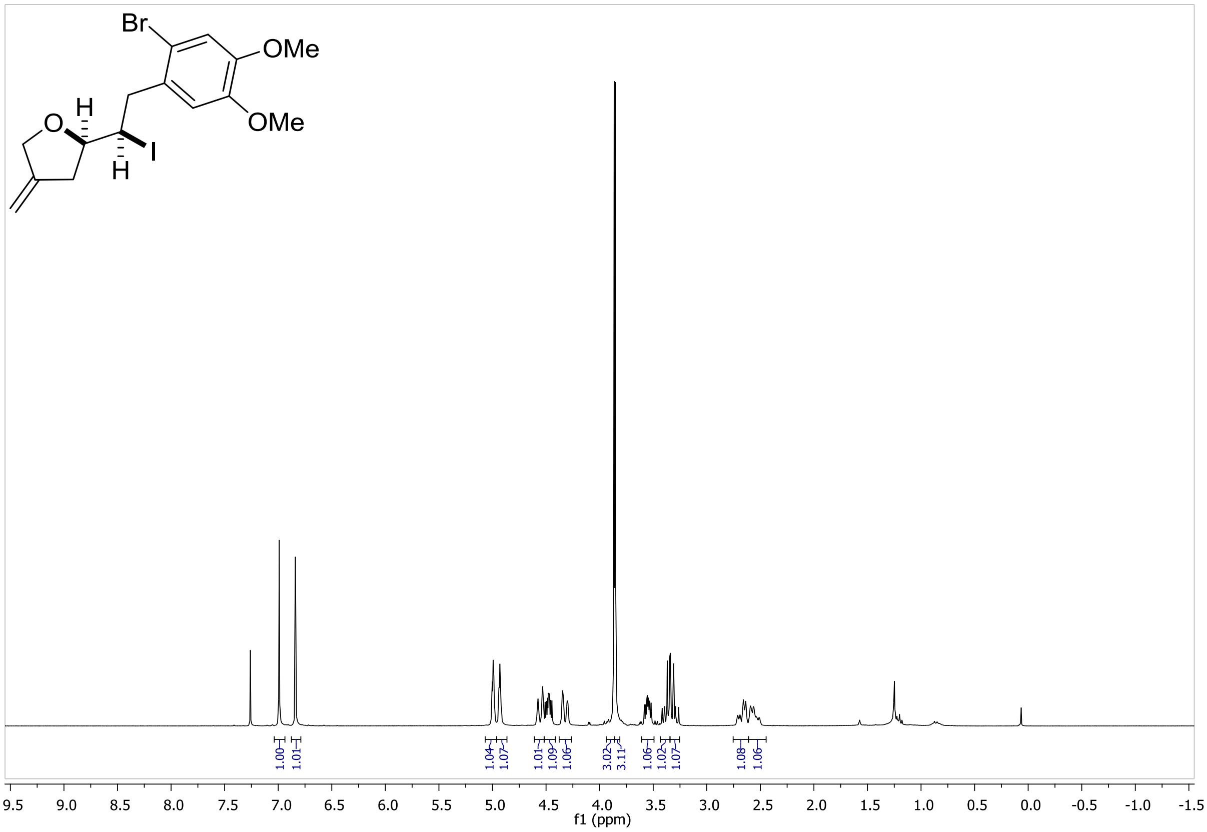

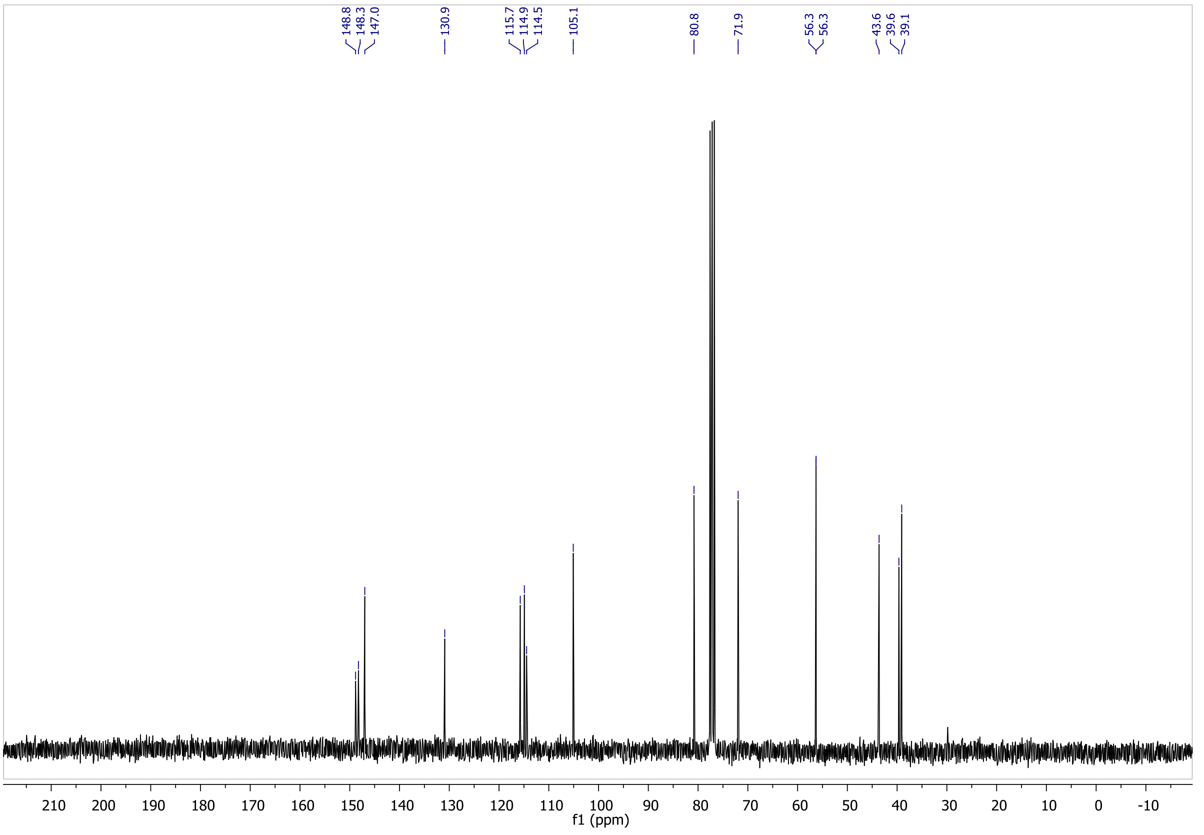

threo-3-(2-Iodo-2-(4-methylenetetrahydrofuran-2-yl)ethyl)furan (5j)

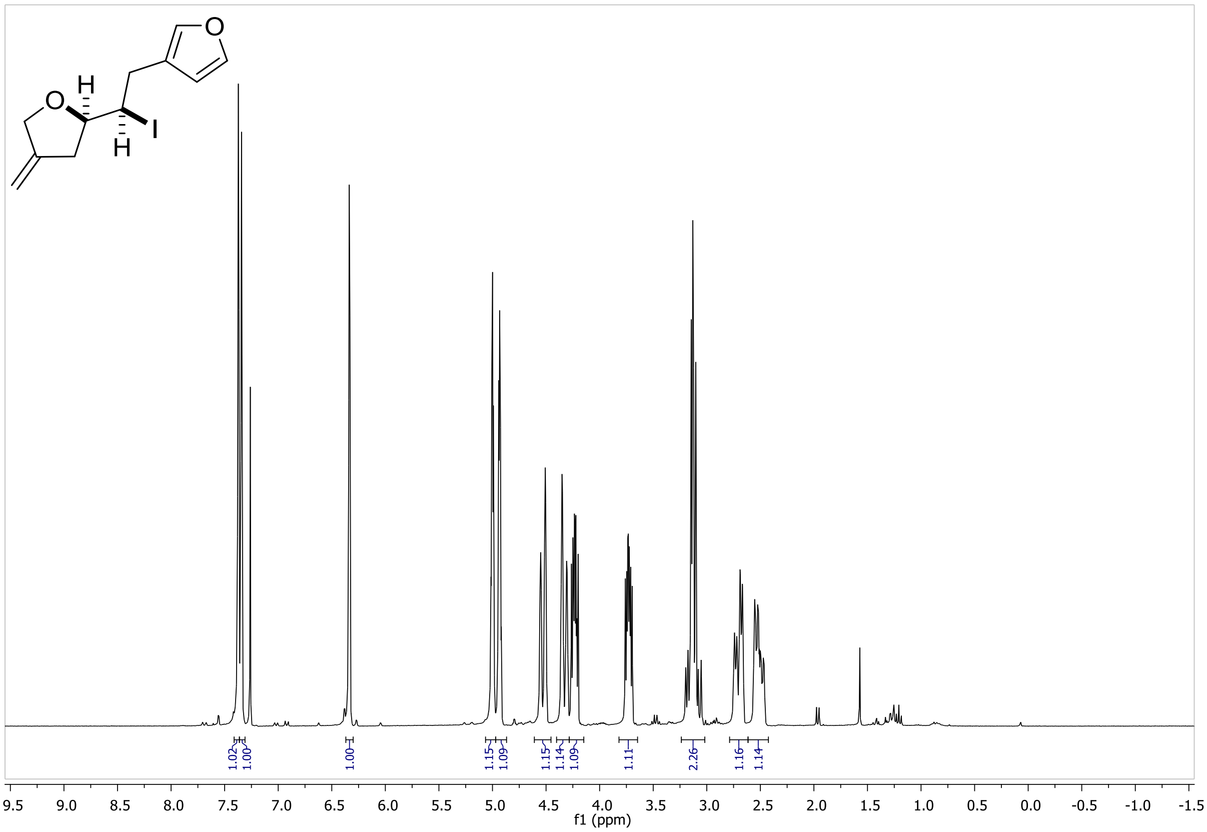

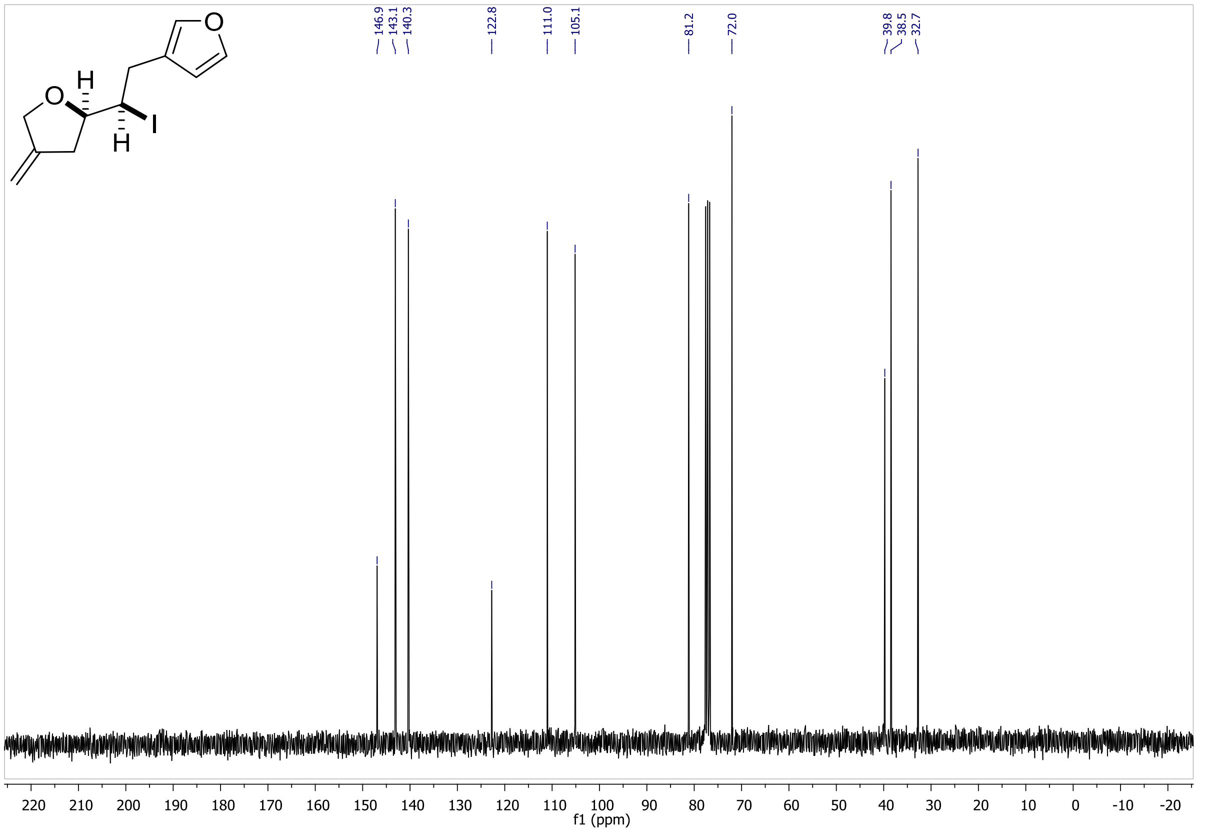

***threo*-2-(1-Iodo-2-(thiophen-2-yl)ethyl)-4-methylenetetrahydrofuran (5k)**

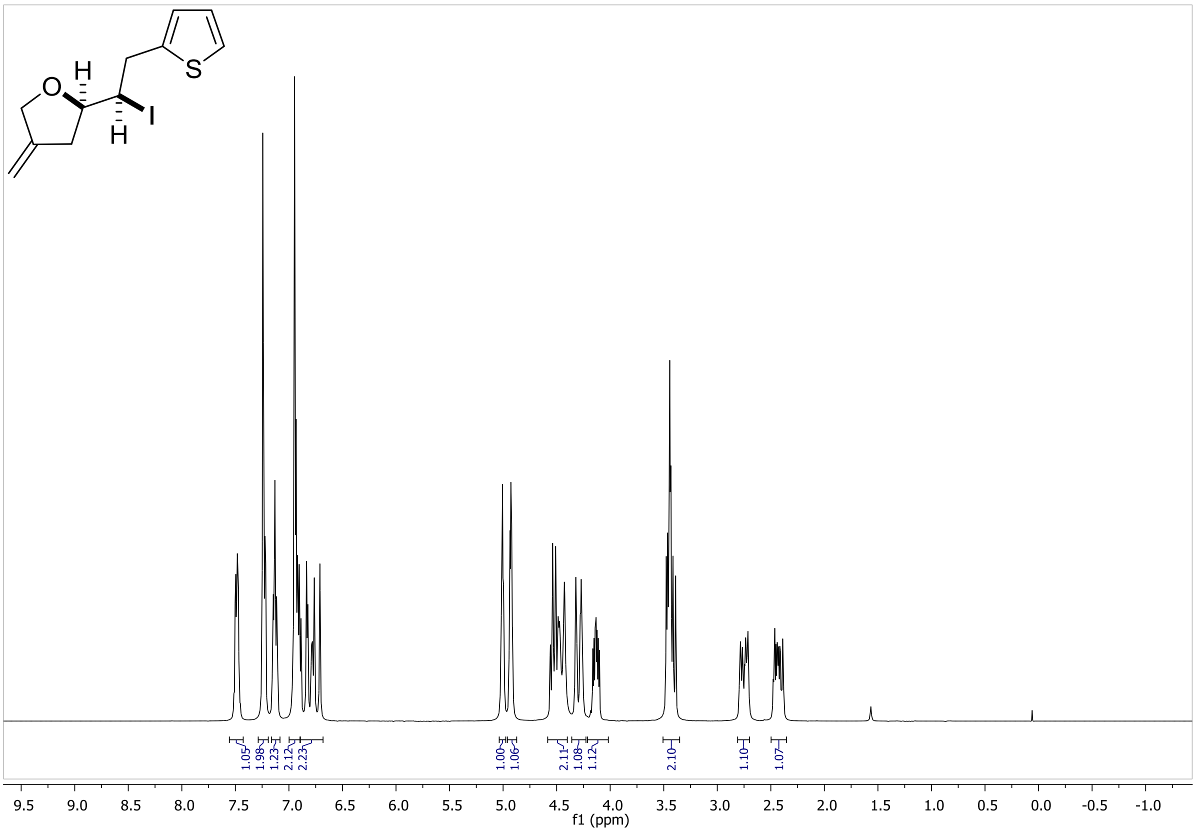

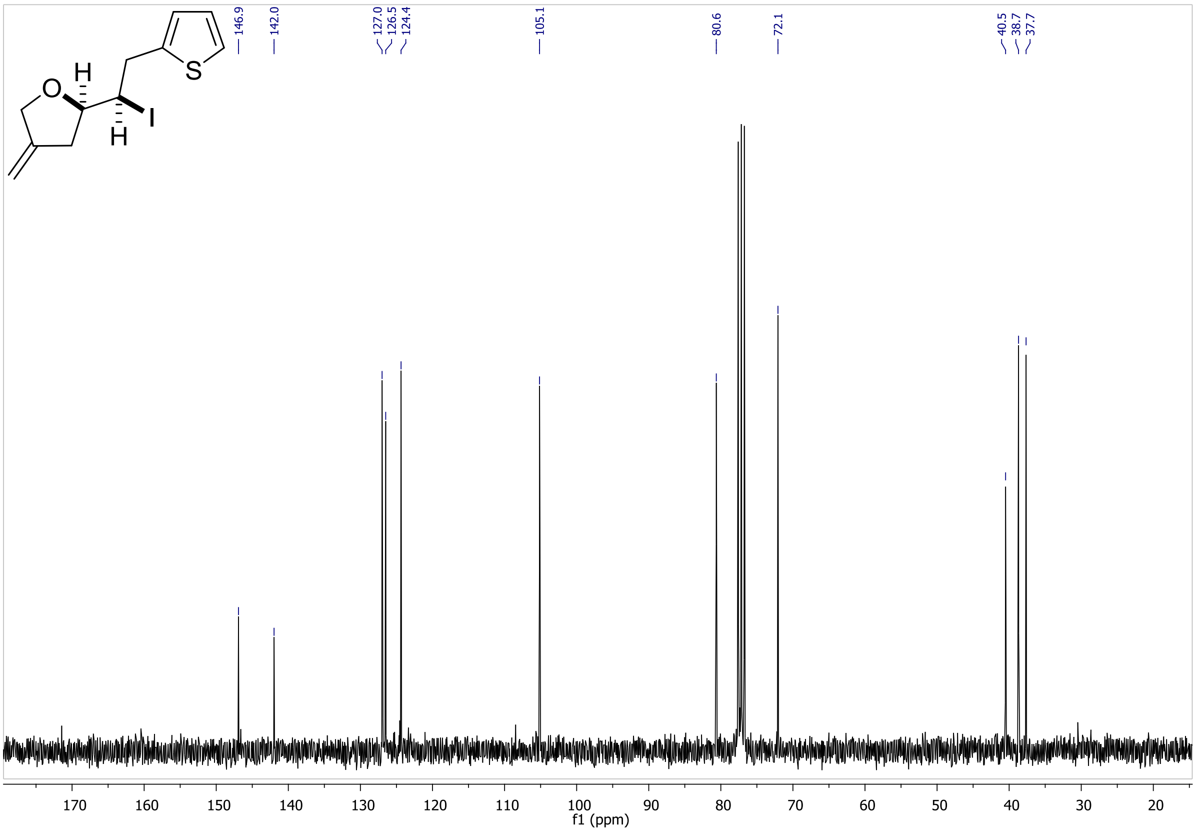

Supplement: File 1 — Experimental details and detailed spectroscopic data. [file Beilstein_J_Org_Chem-11-174-s001.pdf]
